# Careless responding detection revisited: Accuracy of direct and indirect measures

**DOI:** 10.3758/s13428-024-02484-3

**Published:** 2024-08-15

**Authors:** Philippe Goldammer, Peter Lucas Stöckli, Yannik Andrea Escher, Hubert Annen, Klaus Jonas, John Antonakis

**Affiliations:** 1 Military Academy at ETH Zurich, Birmensdorf, Switzerland; 2 Leuphana University Lüneburg, Lüneburg, Germany; 3 University of Zurich, Zurich, Switzerland; 4 University of Lausanne, Lausanne, Switzerland

**Keywords:** Careless responding, Careless responding detection, Infrequency items, Bogus items, Indirect methods

## Abstract

To screen for careless responding, researchers have a choice between several direct measures (i.e., bogus items, requiring the respondent to choose a specific answer) and indirect measures (i.e., unobtrusive post hoc indices). Given the dearth of research in the area, we examined how well direct and indirect indices perform relative to each other. In five experimental studies, we investigated whether the detection rates of the measures are affected by contextual factors: severity of the careless response pattern, type of item keying, and type of item presentation. We fully controlled the information environment by experimentally inducing careless response sets under a variety of contextual conditions. In Studies 1 and 2, participants rated the personality of an actor that presented himself in a 5-min-long videotaped speech. In Studies 3, 4, and 5, participants had to rate their own personality across two measurements. With the exception of maximum longstring, intra-individual response variability, and individual contribution to model misfit, all examined indirect indices performed better than chance in most of the examined conditions. Moreover, indirect indices had detection rates as good as and, in many cases, better than the detection rates of direct measures. We therefore encourage researchers to use indirect indices, especially within-person consistency indices, instead of direct measures.

**Supplementary Information:**

The online version contains supplementary material available at 10.3758/s13428-024-02484-3.

Questionnaire measures are convenient and are used regularly to assess psychological phenomena. However, credible results can be obtained by the survey instrument only if respondents have answered the questions attentively. Unfortunately, it is rare that all respondents will be attentive, especially if their motivation to participate is low (Goldammer et al., 2020). Respondents often rush through the questionnaire without paying attention to item content or instructions (Goldammer et al., 2020; Meade & Craig, 2012). That kind of response behavior has been commonly labeled insufficient effort responding (Huang et al., 2012) or careless responding (Meade & Craig, 2012).

The consequences of undetected careless responding can be severe. Simulations suggest that even small proportions, 10% (Hong et al., 2020; Woods, 2006) or even 5% (Credé, 2010) of careless respondents in the data, can bias results; moreover, bias increases with the rate of careless respondents (Hong et al., 2020). In addition, careless responding has a biasing effect on a range of estimates, including item covariances (Credé, 2010; Goldammer et al., 2020), item means (Goldammer et al., 2020), reliability estimates (Hong et al., 2020; Huang et al., 2012), factor loadings (Kam & Meyer, 2015; Meade & Craig, 2012), and the testing of construct dimensionality (Arias et al., 2020; Goldammer et al., 2020; Woods, 2006). As a consequence, careless responding detection research has gained traction, and several methods for detecting careless responding have been proposed (for an overview, see Curran, 2016). How good are the methods?

A common strategy for detecting careless responding is the use of bogus screening items, such as “I have never used a computer” (Huang, et al., 2015, p. 303), or instructed response items (e.g., “Answer with ‘Disagree’ for this item”; Curran & Hauser, 2019; Edwards, 2019). However, the utility of these explicit or direct measures is questionable for at least two reasons. They may confuse careful respondents and undermine the spirit of cooperation between researchers and respondents (Edwards, 2019; Ward & Meade, 2022). Moreover, respondents could legitimately justify selecting an infrequent response option or not selecting the instructed response option (Curran & Hauser, 2019), which may explain why some studies reported a high rate of participants falsely classified as careless responders (Huang et al., 2012; Niessen et al., 2016).

A better approach might therefore be the use of indirect or unobtrusive measures. Indirect indices make use of a respondent’s deviant or unlikely response pattern. Indirect careless response indices are therefore not salient to respondents, and their use does not alter the questionnaire. Several indirect indices have been proposed for careless responding detection, such as average response time per item (Huang et al., 2012), intra-individual response variability (Marjanovic et al., 2015), and standardized log-likelihood (Niessen et al., 2016).

How is careless responding studied? Many of the measures were developed in an ad hoc manner, and the use of experiments—where the information environment is fully controlled—is unfortunately rare (e.g., Goldammer et al., 2020; Huang et al., 2012; Niessen et al., 2016). In addition, it is unclear for many indices whether their detection performance depends on contextual factors, such as severity of the careless response pattern (i.e., full vs. partial careless responding), type of item keying (i.e., bidirectionally keyed items vs. unidirectionally keyed items only), and type of item presentation (one item per page vs. several items per page [matrix presentation]).

This paper has three aims. In five experiments, we examine (a) how 14 indirect careless responding detection indices perform compared to each other, (b) how the indirect indices perform (individually and jointly) compared to direct careless responding measures (i.e., bogus items and infrequency items), and (c) to what extent the detection performance of the indices is affected by the contextual factors mentioned above. By shedding light on these issues, we provide researchers with guidelines for detecting careless respondents.

In the following, we describe the theoretical basis of the 14 indirect indices that we examined (for an overview, see Table 1). The indices were selected either because they have already been successfully applied for careless responding detection or because they were effective in previous studies for detecting other types of aberrant responding (e.g., faking, cheating). These measures can be grouped into three broad categories: measures of response time, measures of response invariability, and measures of response inconsistency.

**Table 1:** Overview of the 14 examined indirect careless response indices

Index	Source	Screening direction	Experimental evidence for utility	Simulation evidence for utility
**Measure of response time**				
Time	Huang et al. (2012)	Lower values	Huang et al. (2012)	-
**Measures of response invariability**				
Longstring	Johnson (2005)	Higher values	Niessen et al. (2016)	Hong et al. (2020)
IRV	Marjanovic et al. (2015)	Lower values	-	Hong et al. (2020)
**Measures of response inconsistency**				
*Within-person inconsistency*				
Synonyms	Meade and Craig (2012)	Lower values	Goldammer et al. (2020)	Meade and Craig (2012)
Antonyms	Goldberg (2000)	Higher values	Goldammer et al. (2020)	Meade and Craig (2012)
PR	Jackson (1976)	Lower values	Goldammer et al. (2020)	Meade and Craig (2012)
*Inconsistency with the sample norm*				
MD	Mahalanobis (1936)	Higher values	Niessen et al. (2016)	Meade and Craig (2012)
*r*_pbis_	Donlon and Fischer (1968)	Lower values	-	Karabatsos (2003)
*Model-based inconsistency:* *Nonparametric person-fit indices*				
Gnormed	Molenaar (1991)	Higher values	Niessen et al. (2016)	Wind and Wang (2022)
H_t_	Mokken (1971)	Lower values	-	Wind and Wang (2022)
*Model-based inconsistency:* *Parametric IRT-based person-fit indices*				
lz	Drasgow et al. (1985)	Lower values	Niessen et al. (2016)	Hong et al. (2020)
Infit MSE	Wright and Stone (1979)	Higher values	-	Karabatsos (2003)
Outfit MSE	Wright and Stone (1979)	Higher values	-	Karabatsos (2003)
*Model-based inconsistency:* *Parametric covariance structure-based person-fit index*				
INDCHI	Reise and Widaman (1999)	Higher values	Goldammer et al. (2023)	-

Time = average response time per item; Longstring = maximum longstring; IRV = intra-individual response variability; Synonyms = psychometric synonyms; Antonyms = psychometric antonyms; PR = personal reliability; MD = Mahalanobis distance measure; *r*_pbis_ = person-total correlation coefficient; Gnormed = normed Guttman error index for polytomous items; H_t_ = person scalability index; lz = standardized log-likelihood index for polytomous items; Infit MSE = Rasch model-based infit mean squared error; Outfit MSE = Rasch model-based outfit mean squared error; INDCHI = individual contribution to model misfit

## Measures of response time

If respondents are not well motivated to complete a questionnaire, it is likely that they speed up their response rate (i.e., to get through it as quickly as possible). The response time for the total survey and the page-specific response time are measures that may reflect speeded-up response behavior. If the questionnaire is conducted via an online survey tool (e.g., Qualtrics), these measures can usually be extracted automatically. In previous experimental studies, response time measures were found to be very effective in differentiating between careful and careless respondents (Goldammer et al., 2020; Huang et al., 2012; Niessen et al., 2016). We therefore expect careless respondents to complete the total survey or specific parts of a survey much faster than careful respondents (Goldammer et al., 2020; Huang et al., 2012; Niessen et al., 2016).

## Measures of response invariability

To minimize their effort while completing the questionnaire, careless respondents may also use more efficient but content-irrelevant response strategies (i.e., irrelevant to the item content). Using the same response option (e.g., “agree”) or the same adjacent response options (e.g., “agree” and “strongly agree”) over a row of consecutive items would be two such effort-saving response strategies. For instance, these strategies go along with the least cursor or pencil movement needed to complete the questionnaire (Ward & Meade, 2022). Two indices have been commonly used to detect this invariant careless response patterns, maximum longstring index (Johnson, 2005; Meade & Craig, 2012) and intra-individual response variability (Dunn et al., 2018; Marjanovic et al., 2015). Except for Goldammer et al. (2020), previous experimental studies (Huang et al., 2012; Niessen et al., 2016) and simulation studies (Hong et al., 2020; Wind & Wang, 2022) have supported the utility of these invariability indices in detecting careless respondents. Compared to careful respondents, careless respondents might therefore produce response sets with longer strings of identical responses and sets in which the variability of responses is reduced.

## Measures of response inconsistency

Careless respondents answering items irrespective of their content may result in three different low-probability response patterns. First, the pattern will be *inconsistent within itself* (items of similar content are answered differently). Psychometric antonyms (Goldberg, 2000, cited in Johnson, 2005, p. 111), psychometric synonyms (Meade & Craig, 2012, p. 443), and personal reliability (Curran, 2016; DeSimone et al., 2015; Jackson, 1976) reflect this within-person inconsistency. Second, the pattern is *inconsistent with the normative response pattern* (the sample norm of careful respondents). The Mahalanobis distance (Mahalanobis, 1936) and the person-total/personal-biserial correlation coefficient (*r*_pbis_; Donlon & Fischer, 1968) reflect this norm-based inconsistency. Third, the pattern is *inconsistent with the measurement model*. Depending on the measurement model chosen, different types of indices may be calculated that reflect different aspects of this model-based inconsistency. In this paper, we highlight three types of model-based inconsistency indices: (a) nonparametric person-fit statistics such as the normed Guttman error index for polytomous items (Emons, 2008; Molenaar, 1991) and the person or transposed scalability index (Mokken, 1971; Sijtsma, 1986), (b) parametric item response theory (IRT)-based person-fit statistics such as the standardized log-likelihood for polytomous items (Drasgow et al., 1985) and the infit and outfit mean squared error statistics (Wright & Stone, 1979), and (c) a covariance structure-based person-fit index—the individual contribution to the model misfit or χ^2^ (Reise & Widaman, 1999).

### Within-person inconsistency

Careful respondents are expected to choose similar response options when rating items with similar content and to choose opposite response options when rating items with opposite content. Thus, response vectors of synonym pairs should correlate positively, and it follows that response vectors of antonym pairs should correlate negatively. This principle is used for calculating the psychometric synonym index and the psychometric antonym index (DeSimone et al., 2015). For this purpose, the within-person correlation is calculated between the response vectors of strongly correlated synonym or antonym pairs (e.g., greater than |.60| [Meade & Craig, 2012, pp. 442–443]). Optimally, such items pairs are obtained from the correlation matrix of careful respondents. Inconsistency in a respondent’s response pattern is indicated by low values on the synonym index or high values on the antonym index (DeSimone et al., 2015). Previous experimental studies (Goldammer et al., 2020; Huang et al., 2012) and simulation studies (Hong et al., 2020; Meade & Craig, 2012) support the utility of these two indices in detecting careless responding. Compared to careful respondents, we therefore expect careless respondents to have lower values on the synonym index and higher values on the antonym index (e.g., Goldammer et al., 2020).

Careful respondents are also expected to choose similar response options when they rate items of the same unidimensional scale. Accordingly, a score that is based on half the scale items (e.g., even items) should correlate positively with a score based on the rest of the items. This principle is applied when calculating personal reliability, which is also referred to as even–odd consistency (Curran, 2016; DeSimone et al., 2015; Jackson, 1976). If a questionnaire includes at least three scales, personal reliability can be obtained by calculating the within-person correlation between the two vectors of even–odd scale score halves (DeSimone et al., 2015). Inconsistency in a respondent’s response pattern is indicated by a low personal reliability score (DeSimone et al., 2015). In previous experimental work (Goldammer et al., 2020; Huang et al., 2012; Niessen et al., 2016) and a simulation (Meade & Craig, 2012), this index was very effective in classifying respondents. We therefore expect careless respondents to have a lower personal reliability score than that of careful respondents (e.g., Goldammer et al., 2020).

### Inconsistency with the sample norm

Typically, the Mahalanobis distance (MD) has been used in regression analyses for detecting multivariate outliers (see Tabachnick & Fidell, 2007, pp. 73–77). A small distance value indicates that a respondent’s response pattern is closely in line with that of the sample norm; a large distance value indicates that a respondent’s response pattern deviates substantially from the sample norm (Goldammer et al., 2020). In previous experimental (Goldammer et al., 2020; Huang et al., 2012; Niessen et al., 2016) and simulation studies (Hong et al., 2020; Meade & Craig, 2012; Wind & Wang, 2022), the MD was very effective in detecting careless responding. We therefore expect careless respondents to have larger MD values than those of careful respondents.

Another measure, the person-total/personal-biserial correlation coefficient (*r*_pbis_), is comparable to an item-total or item-rest correlation in the context of a scale reliability analysis (Curran, 2016, pp. 12–13). This measure captures whether response patterns of individuals correlate positively with the response pattern of the sample norm. Low or even negative correlation coefficients are a point of concern. Thus, if the normative response pattern is set by careful respondents, we expect careless respondents to have produced with their content-irrelevant responses a response pattern that correlates only minimally with that of the norm (Goldammer et al., 2020); a simulation study shows likewise (Karabatsos, 2003).

#### Model-based inconsistency: Nonparametric person-fit indices

The idea behind Guttman errors is that respondents should answer test items in accordance with their total score (e.g., Meijer et al., 2016; Niessen et al., 2016). In the context of polytomous items, respondents produce a Guttman error if they take an unpopular item step after not taking a more popular item step previously (Niessen et al., 2016, pp. 10–11). Thus, it is very likely that careless respondents produce Gutman errors with their content-irrelevant response pattern. Initial support for the utility of Gnormed (i.e., the normed and polytomous version of the Guttman error index) in detecting careless responding was found in an experimental study (Niessen et al., 2016) and two simulation studies (Karabatsos, 2003; Wind & Wang, 2022). We therefore expect careless respondents to have larger Gnormed values than those of careful respondents.

The person or transposed scalability index (H_t_) represents an extension of the conventional Guttman error index and can be interpreted as the ratio of the frequencies of observed to expected Guttman errors (Wind & Wang, 2022). Previous simulation studies (Karabatsos, 2003; Wind & Wang, 2022) suggested the utility of the H_t_ in detecting careless responding. Compared to careful respondents, we therefore expect careless respondents to have poorer scalability and thus smaller H_t_ values.

### Model-based inconsistency: Parametric IRT-based person-fit indices

Compared to nonparametric person-fit statistics, which are only calculated from the respondents’ scored responses, parametric person-fit statistics require the estimation of a measurement model to be calculated (Karabatsos, 2003). In the case of an IRT model, the respondent’s latent trait level or theta is one of the core parameters and the basis for IRT-based person-fit statistics (Karabatsos, 2003), such as the standardized log-likelihood for polytomous items (lz), and the infit and outfit mean squared error (MSE) statistics. In general, such parametric IRT-based person-fit indices quantify to what extent a respondent’s response pattern is in line with the response pattern that would be expected under the person’s latent trait level (Glas & Khalid, 2017). Whereas consistently responding respondents are expected to respond to items according to their latent trait level, inconsistently responding respondents are expected to provide a response pattern that is very unlikely under the person’s latent trait level.

In the case of the lz, a misfit between the observed and expected response pattern is indicated by large negative values (Reise & Widaman, 1999, p. 6). An experimental study (Niessen et al., 2016) and simulation studies (Hong et al., 2020; Karabatsos, 2003) found support for the utility of this index in detecting careless responding. We therefore expect careless respondents to have lower lz values than those of careful respondents.

The Rasch model (a specific IRT model)-based infit and outfit MSE statistics represent two other indices that indicate deviations between the observed response pattern and the one that would be expected under the person’s latent trait level (Wright & Stone, 1979). Both these error indices range from 0 to infinity, and large positive infit and outfit MSE values are generally taken as indication of a poor fit between the observed and expected response pattern (Linacre, 2002). Because previous simulation studies (Jones et al., 2023; Karabatsos, 2003) suggested the utility of these two indices in detecting careless responding, we expect careless respondents to have larger infit and outfit MSE values than those of careful respondents.

### Model-based inconsistency: Parametric covariance structure-based person-fit index

In contrast to parametric IRT-based person-fit indices, the individual contribution to the model misfit or χ2 (INDCHI) is based on the comparison of two covariance structure models—the saturated model and the substantive factor model (Reise & Widaman, 1999). This results in a statistic, INDCHI, that reflects the individual contribution to the overall factor model misfit (Reise & Widaman, 1999). Respondents with a response pattern that is rather unlikely under the estimated factor model will have larger INDCHI values than respondents that have produced a response pattern that is consistent with the estimated factor model (Reise & Widaman, 1999). Because this index performed well in detecting participants that were instructed to fake-bad (Goldammer et al., 2023), we expect this index to also have utility in detecting careless responding. Thus, we assume that careless respondents will have larger INDCHI values than those of careful respondents.

## Indirect careless responding measures: Unknowns

As the review of the 14 indices shows, there are several promising indirect indices available for detecting careless responding in data sets. However, researchers still have little guidance about the relative effectiveness of different indirect careless responding indices. Even though some previous experimental studies have examined the utility of careless response indices, the analyses of these studies were limited to a selected group of indices (e.g., Goldammer et al. (2020) examined seven indices, Huang et al. (2012) examined four indices, and Niessen et al. (2016) examined six indices). By examining a selection of 14 indirect careless response indices, we provide a more informative picture of the indices’ relative performance. Our first research question was the following:

- ***Research question 1***: How accurate are the 14 indirect indices designed to detect careless responding compared to each other?

Even less is known about how indirect indices perform in comparison to a direct careless response measure. For instance, experimental research has not examined how the intra-individual response variability (IRV), psychometric synonyms and antonyms, *r*_pbis_, H_t_, infit and outfit MSE, and INDCHI indices perform compared to a direct measure (e.g., bogus and infrequency items) in detecting careless responding. We therefore investigated the following research question:

- ***Research question 2***: How accurately do these 14 indirect indices detect careless responding compared to a direct careless response measure?

If several of these indirect indices turn out to be effective in detecting careless responding and increase classification accuracy beyond a direct careless responding measure, it may be fruitful to examine to what extent a selected group of indirect indices, as a set, can outperform a direct careless responding measure. We therefore addressed the following research question:

- ***Research question 3***: How accurately does a set of effective indirect indices detect careless responding compared to a direct measure?

In addition—and most importantly regarding our contribution—little is known on how the detection performance of the careless response indices is affected by contextual factors, such as the severity of the careless response pattern (i.e., partial vs. full careless responding), the type of item keying (i.e., bidirectionally keyed items vs. unidirectionally keyed items only), and the type of item presentation (one item per page vs. several items per page [matrix presentation]). Even though some simulation studies (Hong et al., 2020; Meade & Craig, 2012) partially examined the effects of contextual factors on the detection rate of careless response indices, the generalizability of such simulation findings may be limited, because computer-generated careless response data may not fully reflect human-generated careless response data. Niessen et al. (2016), for instance, found detection rates of indirect indices to be remarkably lower when human-generated careless response data were used, compared to when computer-generated random data were used; simulated careless response data may lead to detection rates that are too optimistic. Thus, examining the following three research questions with human-generated careless response data may bring us novel insights:

- ***Research question 4***: How does the severity of the careless response pattern (i.e., partial vs. careless responding) affect the detection rate of careless response indices?
- ***Research question 5***: How does the type of item keying (i.e., bidirectionally keyed items vs. unidirectionally keyed items only) affect the detection rate of careless response indices?
- ***Research question 6***: How does the type of item presentation (one item per page vs. several items per page [as matrix]) affect the detection rate of careless response indices?

To examine the six research questions, we conducted five studies in which the detection accuracy of the indices was examined based on experimentally induced careless response sets. In Studies 1 and 2, we used a between-subject factor design in which participants had to rate the personality of an actor that presented himself in a 5-min-long videotaped speech. In Studies 3, 4, and 5, we used a within- and between-subject factor design in which participants rated their own personality across two measurements. Notably, and in contrast to previous experimental studies on careless responding detection (Goldammer et al., 2020; Huang et al., 2012; Niessen et al., 2016), each of our five studies was designed in such a way that we could conduct a true test of the effect of the response instructions on participants’ response behavior (e.g., by comparing the ratings of the experimental groups with that of a norm group).

## Study 1

In Study 1, we addressed research questions 1 to 4.

### Method

#### Participants

The participants in Study 1 were 357 German-speaking conscripts doing military service in the fall of 2021 in three randomly drawn basic training camps. The participants in this sample were on average 20.33 years old (*SD* = 1.31) and predominantly men (*n* = 355, 99.4%). The educational level of the participants in the study was as follows: More than a third (*n* = 123, 34.5%) had completed upper secondary school, and the majority (*n* = 220, 61.6%) had completed a certified apprenticeship. Only a minority of the participants (*n* = 14, 3.9%) indicated nine years of compulsory schooling as their highest educational level.

#### Experimental conditions and survey arrangement

The data were gathered platoon-wise. After providing the members of each platoon with a general introduction to the study, we randomly assigned them to one of three experimental conditions—the careful responding condition (*n* = 119) or one of the two careless responding conditions (i.e., partially careless—careless responding to the last third of the questionnaire items [*n* = 120]; fully careless—careless responding to all questionnaire items [*n* = 118]). Civilian instructors, who were randomly assigned to one of the three conditions, then led the three subgroups into separate labs. All participants were then told that they would take part in an experiment that was about how to best identify careless responding in survey data and that after completing the survey they would all receive 10 Swiss francs (about US $10) as compensation for their efforts.

Participants were further told that they would first have to attentively watch a 5-min-long videotaped self-presentation by a non-commissioned officer (NCO) candidate and would then have to complete a questionnaire (i.e., Big-Five Inventory [BFI-2]; Soto & John, 2017) about that NCO candidate. The self-presentation was played by a non-professional actor and was designed such that he would receive high scores on the dimensions extraversion, conscientiousness, agreeableness, and open-mindedness, and low scores on the dimension negative emotionality (see supplements for video and translated script). After watching the video, participants in the careful responding group were asked to complete the questionnaire on the NCO candidate accurately and honestly (i.e., they were told to simply give their honest, personal view of the candidate); participants in the partially careless responding condition were asked to complete the first survey part (i.e., 66% of the items) accurately and honestly and the second survey part (i.e., 33% of the items) with the following attitude: They should show no great interest in completing the rest of the survey. Accordingly, they were to get the survey over with as quickly as possible, without being caught by our careless responding detection method. Participants in the fully careless responding condition were asked to complete the full questionnaire with the following attitude: They should respond as if they had no great interest in completing the survey. Accordingly, they were to get the survey over with as quickly as possible, without being caught by our careless responding detection methods. After receiving the instructions, the participants started completing the online questionnaire, beginning with three sociodemographic questions. They then completed the main part of the questionnaire, in which the order of the items was randomized and each item was displayed on a single web page.

#### Substantive measures

The participants completed the German translation (Danner et al., 2019) of the BFI-2 (Soto & John, 2017) to rate the NCO candidate in the video. The BFI-2 is composed of the five trait scales extraversion (α_Careful_ = 0.78, α_Partially careless_ = 0.49, α_Fully careless_ = 0.62), agreeableness (α_Careful_ = 0.88, α_Partially careless_ = 0.65, α_Fully careless_ = 0.73), conscientiousness (α_Careful_ = 0.86, α_Partially careless_ = 0.64, α_Fully careless_ = 0.77), negative emotionality (α_Careful_ = 0.80, α_Partially careless_ = 0.51, α_Fully careless_ = 0.71), and open-mindedness (α_Careful_ = 0.80, α_Partially careless_ = 0.39, α_Fully careless_ = 0.56). All 60 bidirectionally keyed items (i.e., 30 positively and 30 negatively worded items) of the BFI-2 were rated on a Likert scale ranging from 1 = *completely disagree* to 6 = *completely agree.*

#### Direct careless responding measure

We used three bogus items as a direct careless responding measure. The two items “I have been to every country in the world” and “I have never brushed my teeth” were taken from Meade and Craig (2012, p. 441), and the item “I have never used a computer” was taken from Huang et al., (2015, p. 303). We chose these three bogus items because they turned out to be the only items for which participants in the two studies by Curran and Hauser (2019, p. 4) could not find valid justification when selecting infrequent response options. In addition, using three bogus items per 60 substantial items seemed reasonable to give this direct measure a fair chance to perform well (see Huang et al., 2015, p. 303). These three bogus items were embedded in the regular questionnaire and randomly displayed. As with the substantial items, respondents rated the three bogus items on a Likert scale from 1 = *completely disagree* to 6 = *completely agree*. For the main analyses, an average score was computed across the three items (α_Careful_ = 0.43, α_Partially careless_ = 0.34, α_Fully careless_ = 0.71). If a respondent’s response was missing, the average score was based on the remaining non-missing responses. However, this average score could not be computed for one respondent in the careful responding group, because this respondent did not answer any of the bogus items.

#### Indirect careless responding measures

We calculated the 14 indirect indices from our representative selection. For computing these indirect indices, only the 60 items of the BFI-2 were used. Detailed information on how these indices were computed is provided in the supplementary material.

#### Careless responding criterion and analytical procedure

The indices’ classification accuracy of careless and careful responders was our outcome variable in Study 1. We therefore plotted for every index (and selected combinations of indices) a receiver operating characteristic (ROC) curve (e.g., Swets, 1986) and examined the corresponding area under the curve (AUC). We used the tie-corrected nonparametric method (i.e., trapezoidal approximation) with bootstrap standard errors (i.e., 1000 replications) for estimating the AUCs and its standard errors. All these analyses were performed in Stata (StataCorp, 2021).

### Results

#### Manipulation check

To examine whether our manipulation worked, we compared the scores of the five substantive scales and three indirect careless response indices (i.e., average response time per item, IRV, resampled personal reliability [RPR]) of the experimental groups with those of a norm group. The comparisons revealed that our manipulation was successful (see supplementary material and Table S1). Thus, we proceeded with the main analyses.

#### Descriptive statistics

The condition-specific correlation matrices as well as the condition-specific means and standard deviations of the substantive measures and careless responding indices are reported in the supplementary material (see Tables S2–S4).

#### Comparisons between the indirect indices

The omnibus test indicated a significant inequality between the AUCs for detecting partially careless respondents (*F*[13, 4.9*10^9^] = 18.28, *p* < .001) and between the AUCs for detecting fully careless respondents (*F*[13, 2.3*10^10^] = 25.91, *p* < .001). These global inequalities were then further explored through Bonferroni-corrected pairwise comparisons.

For detecting partially careless respondents (i.e., Careless 33%), the following indices turned out to be effective (i.e., the confidence interval of their AUC did not include 0.5): RPR (i.e., repeated calculation across several random splits of scale halves), MD, lz, psychometric synonyms, Gnormed, outfit MSE, infit MSE, *r*_pbis_, H_t_, INDCHI, psychometric antonyms, and average response time per item (see Table S5 for AUCs). Of these 12 effective indices, the RPR and the MD tended to be the best—they significantly outperformed some of the other effective indices in terms of AUC. In contrast, maximum longstring and IRV did not perform well in detecting partially careless respondents. The accuracy of these indices was not better than chance (i.e., the confidence interval of their AUC included 0.5).

For detecting fully careless respondents, all 14 examined indices turned out to be effective (see Table S6). Again, the RPR tended to be the best-performing index. It outperformed more than half the indices in terms of AUC.

Lastly, we addressed research question 4 and compared each index’s performance in detecting partially careless respondents with its performance in detecting fully careless respondents. For each index, the condition-specific univariate logit estimates (i.e., estimate for Careless 33% and estimate for Careless 100%) were therefore stacked into one model, simultaneously estimated, and tested for equality by using seemingly unrelated estimation (i.e., SUEST; Weesie, 1999). The following seven indices performed equally well in detecting partially and fully careless responding according to the Bonferroni-corrected critical χ^2^ or *F*-value of 8.49 (i.e., α = 0.05/14): H_t_ (χ^2^[1] = 0.03), RPR(χ^2^[1] = 0.44), psychometric synonyms (χ^2^[1] = 1.05), INDCHI (χ^2^[1] = 1.71), *r*_pbis_ (χ^2^[1] = 2.79), MD (χ^2^[1] = 3.64), and psychometric antonyms (*F*[1, 1.7*10^6^] = 4.72).

In contrast, the following indices performed better in detecting one specific careless responding form (i.e., partially vs. fully careless responding). For one, Gnormed (χ^2^[1] = 9.11), lz (χ^2^[1] = 10.91), infit MSE (χ^2^[1] = 12.51), and outfit MSE (χ^2^[1] = 13.18) were found to better detect partially careless responding than fully careless responding. For another, the average response time per item (χ^2^[1] = 12.11) and the IRV (χ^2^[1] = 18.63) were found to better detect fully careless responding than partially careless responding. Even though the Bonferroni-corrected critical χ^2^ value was not passed in the case of maximum longstring (χ^2^[1] = 7.23), it nevertheless seems legitimate to also claim that this index better detects fully careless responding than partially careless responding, especially when taking into account the condition-specific AUCs and their confidence intervals (see Tables S5 and S6). Whereas the 95% confidence interval of the AUC in the partially careless responding condition included 0.5, that of the AUC in the fully careless responding condition did not.

#### Comparisons between the average score on bogus items and indirect indices

We then compared the detection performance of each effective indirect index with that of the average score on the three bogus items (see Table 2). For detecting partially careless respondents, the Bonferroni-corrected pairwise comparisons revealed the following results: Whereas one half of the examined indices (i.e., RPR, MD, lz, psychometric synonyms, Gnormed, outfit MSE, infit MSE) performed significantly better than the average score on the bogus items, the other half (i.e., *r*_pbis_, H_t_, INDCHI, psychometric antonyms, average response time per item) performed as well as the average score on the bogus items. For detecting fully careless respondents, the Bonferroni-corrected pairwise comparisons revealed the following results: Whereas the RPR and the average response time per item performed significantly better than the average score on the bogus items, the majority of indices (i.e., MD, lz, psychometric synonyms, Gnormed, *r*_pbis_, H_t_, INDCHI, psychometric antonyms, outfit MSE, infit MSE, maximum longstring) were as accurate as the average score on the bogus items. Only the IRV turned out to have a significantly lower detection accuracy than the average score on the bogus items. In addition, we also examined the indices’ incremental validity beyond the average score of bogus items. These estimates are reported in the supplementary material (see Table S7).

**Table 2:** Pairwise comparisons between the average score on bogus items and indirect indices regarding the detection of partially and fully careless responding in Study 1

Index	Careless 33% ^a^		Careless 100% ^b^	
	AUC_[95% CI]_	*F*(1, > 10^6^)	AUC_[95% CI]_	*F*(1, > 10^6^)
Bogus (reference)	.66_[.59, .73]_		.76_[.70, .82]_	
RPR	.86_[.81, .91]_	37.38	.88_[.84, .93]_	14.22
MD	.84_[.80, .89]_	26.53	.79_[.73, .84]_	0.43
lz	.81_[.76, .86]_	15.91	.71_[.64, .77]_	1.56
Synonyms	.81_[.75, .86]_	17.08	.83_[.78, .89]_	3.57
Gnormed	.81_[.75, .86]_	14.73	.71_[.64, .77]_	1.49
Outfit MSE	.80_[.75, .86]_	13.01	.66_[.59, .73]_	5.17
Infit MSE	.79_[.74, .85]_	11.47	.65_[.59, .72]_	5.43
*r*_pbis_	.78_[.72, .84]_	8.64	.85_[.80, .90]_	6.52
H_t_	.77_[.71, .83]_	7.31	.79_[.73, .85]_	0.58
INDCHI	.72_[.65, .78]_	1.89	.67_[.60, .74]_	4.27
Antonyms	.71_[.64, .78]_	1.40	.81_[.76, .87]_	1.92
Time	.68_[.61, .75]_	0.19	.87_[.83, .92]_	10.22
Longstring	-	-	.64_[.57, .71]_	7.63
IRV	-	-	.62_[.54, .69]_	9.79

Only indirect indices that turned out to be effective in detecting partially or fully careless responding were examined. The average score on the bogus items could not be computed for one respondent in the careful responding group, because the respondent did not answer any of the bogus items. In addition, the antonym index could not be computed for one respondent in the partially careless responding group (i.e., Careless 33%) and for two respondents in the fully careless responding group (i.e., Careless 100%), because one of the antonym vectors had no variance. Thus, all estimates and test statistics are based on 100 multiply imputed data sets. All indices were coded such that higher scores were more indicative of careless responding. The Bonferroni-corrected critical *F*-value for each of the 26 comparisons was 9.62 (i.e., 12 pairwise comparisons in the partially careless responding condition, 14 pairwise comparisons in the fully careless responding condition); if this value is exceeded, the AUC of bogus items and that of the indirect index can be considered significantly different from each other. AUC = area under the receiver operating characteristic curve; Time = average response time per item; Longstring = maximum longstring; IRV = intra-individual response variability; Synonyms = psychometric synonyms; Antonyms = psychometric antonyms; RPR = resampled personal reliability; MD = Mahalanobis distance measure for which the calculation was based on subsamples; *r*_pbis_ = person-total correlation coefficient for which the calculation was based on subsamples; Gnormed = global (i.e., the average of the scale-specific scores was computed) normed Guttman error index for polytomous items; H_t_ = global (i.e., the average of the scale-specific scores was computed) person scalability index; lz = global (i.e., the average of the scale-specific scores was computed) standardized log-likelihood index for polytomous items; Infit MSE = global (i.e., the average of the scale-specific statistics was computed) Rasch model-based infit mean squared error; Outfit MSE = global (i.e., the average of the scale-specific statistics was computed) Rasch model-based outfit mean squared error; INDCHI = global (i.e., the average of the scale-specific scores was computed) individual contribution to model misfit for which the calculation of the two person-specific model log-likelihood values was based on subsamples

^a^ A subsample that contained careful respondents and respondents that had partially careless responding was used to determine the classification accuracy (*n* = 239). ^b^ A subsample that contained careful respondents and respondents that had fully careless responding (*n* = 237) was used to determine the classification accuracy

#### Comparisons between the average score of bogus items and sets of indirect indices

Lastly, we compared the performance of three sets with that of the average score on the three bogus items. In the first set, all effective indirect indices were used in combination. In the second set, the three most accurate indices were used in combination. In the third set, the three indices that needed the fewest code lines until the final index was obtained (see supplementary Table S8) were used in combination. For these comparisons, we used linear multiple imputation predictions of logit models as input for the ROC analyses.

Most notably, each of the three examined sets performed significantly better than the average score on the three bogus in terms of AUC, no matter which form of careless responding was detected (see Table 3). In addition, some of the sets even outperformed the average score on bogus items in terms of sensitivity at low-false positive rates (see Table 3). For instance, at a false-positive rate of 5%, the set in which all effective indirect indices were used in combination allowed a detection rate of 69% of the partially careless respondents, whereas the average score on bogus items only detected 35%. This difference in sensitivity at a false-positive rate of 5% also turned out to be significant for fully detected careless respondents. Whereas the set of all indirect indices detected 73% of the fully careless respondents, the average score on the bogus items detected only 47%. These analyses showed that—independently of the careless responding pattern—the combined use of indirect indices leads to significantly better identification of careless responding participants than frequently used bogus items do.

**Table 3:** Pairwise comparisons between the average score on bogus items and sets of indirect indices regarding the detection of partially and fully careless responding in Study 1

	AUC comparison		Sen95 comparison ^c^		Sen99 comparison ^d^	
Index	AUC_[95% CI]_	χ^2^(1)	Sen95	z	Sen99	z
Careless 33% ^a^						
Bogus (reference)	.66_[.59, .73]_		.35		.06	
Set 1 (all effective indirect indices)	.90_[.85, .94]_	48.59	.69	− 3.92	.46	− 2.53
Set 2 (RPR, MD, lz)	.88_[.83, .92]_	41.12	.58	− 2.40	.11	− 0.28
Set 3 (Time, MD, *r*_pbis_)	.86_[.81, .91]_	31.71	.49	− 1.41	.39	− 3.72
Careless 100% ^b^						
Bogus (reference)	.76_[.70, .82]_		.47		.16	
Set 1 (all effective indirect indices)	.95_[.92, .98]_	40.76	.73	− 3.44	.66	− 5.54
Set 2 (RPR, time, *r*_pbis_)	.94_[.91, .97]_	36.12	.73	− 2.97	.53	− 2.14
Set 3 (Time, IRV, longstring)	.89_[.85, .93]_	13.34	.63	− 1.88	.42	− 2.32

Only indirect indices that turned out to be effective in detecting careless respondents were examined. The average score on the bogus items could not be computed for one respondent in the careful responding group, because this respondent did not answer any of the bogus items. In addition, the antonym index could not be computed for one respondent in the partially careless responding group (i.e., Careless 33%) and for two respondents in the fully careless responding group (i.e., Careless 100%), because one of the antonym vectors had no variance. We used linear multiple imputation predictions (across 100 imputations) of logit models as input for the ROC analyses. All indices were coded such that higher scores were more indicative of faking. Set 1 included all effective indirect indices. Set 2 included the three most accurate indices. Set 3 included the three easiest-to-compute indices (i.e., lowest number of code lines required). The Bonferroni-corrected critical χ^2^ value for each of the six AUC comparisons was 6.96 (i.e., three pairwise comparisons in the partially careless responding condition, three pairwise comparisons in the fully careless responding condition). If this value is exceeded, the two AUCs can be considered significantly different from each other. The Bonferroni-corrected critical *z*-value for each of the 12 sensitivity comparisons was 2.87 (i.e., six pairwise comparisons in the partially careless responding condition, six pairwise comparisons in the fully careless responding condition). If this *z*-value is exceeded, the sensitivity of the reference and the sensitivity of the comparison can be considered significantly different from each other. The standard error of the difference between the sensitivities was determined through bootstrapping with 1000 replications. AUC = area under the receiver operating characteristic curve; Time = average response time per item; Longstring = maximum longstring; IRV = intra-individual response variability; RPR = resampled personal reliability; MD = Mahalanobis distance measure for which the calculation was based on subsamples; *r*_pbis_ = person-total correlation coefficient for which the calculation was based on subsamples; lz = global (i.e., the average of the scale-specific scores was computed) standardized log-likelihood index for polytomous items

^a^ A subsample that contained careful respondents and respondents that had partially careless responding was used to determine the classification accuracy (*n* = 239). ^b^ A subsample that contained careful respondents and respondents that had fully careless responding (*n* = 237) was used to determine the classification accuracy. ^c^ Sensitivity of the index set at specificity level of 95% (i.e., false-positive rate of 5%). ^d^ Sensitivity of the index set at specificity level of 99% (i.e., false-positive rate of 1%)

## Study 2

Study 1 revealed that indirect indices are effective in detecting different types of careless responding and may even outperform direct measures (i.e., bogus items). Study 2 aimed to replicate these findings in another sample and with a different set of item keying, and thus helped us to gain further insights regarding research questions 1 to 4. In contrast to Study 1, in which respondents were asked to rate the NCO in the video with a set of bidirectionally keyed items (i.e., items of the same unidimensional construct were positively and negatively worded/keyed), the respondents in Study 2 were asked to rate the NCO with a set of unidirectionally keyed items (i.e., all items of same unidimensional construct were scored in the same direction). This design allowed us to compare the indices’ detection performance across Study 1 and 2, and thus to examine research question 5 as well.

### Method

#### Participants

The participants in Study 2 were 341 German-speaking conscripts doing military service in the fall of 2021 in three randomly drawn basic training camps. The participants in this sample were on average 20.15 years old (*SD* = 1.49) and predominantly men (*n* = 328, 96.2%). The educational level of the participants in the study was as follows: Most of the participants had either completed upper secondary school (*n* = 166, 48.7%) or completed a certified apprenticeship (*n* = 168, 49.3%). Only seven participants (2.0%) indicated the nine years of compulsory schooling as their highest educational level.

#### Experimental conditions and survey arrangement

The experimental conditions and the survey arrangement were identical to those in Study 1. The participants in Study 2 were randomly assigned to one of three experimental conditions— the careful responding condition (*n* = 112), or one of the two careless responding conditions (i.e., partially careless—careless responding to the last third of the questionnaire items [*n* = 114], fully careless—careless responding to all questionnaire items [*n* = 115]).

#### Substantive measures

To rate the NCO candidate in the video, the participants completed a selection of unidirectionally keyed items (i.e., all items of the same construct were keyed in the same direction) of the German adaptation (IPIP-5F-30F-R1; Iller et al., 2020) of the International Personality Item Pool (IPIP; Goldberg et al., 2006). In the complete version of the IPIP-5F-30F-R1, each of the five dimensions extraversion, agreeableness, conscientiousness, neuroticism, and openness to experience is measured with six facets, and each facet is measured with six items. To build a questionnaire of unidirectionally keyed items for Study 2 that was comparable to the questionnaire that we used in Study 1 in terms of content and length, we proceeded as follows: First, we selected from each of the five dimensions those three facets that most closely matched those of the BFI-2. Second, we selected from each of these 15 facets those four items that we thought could be most easily assessed by the participants after the video presentation. Finally, this selection resulted in a questionnaire of 60 unidirectionally keyed items (see supplementary Table S9), each of which had to be rated on a Likert scale ranging from 1 = *completely disagree* to 6 = *completely agree*. The condition-specific Cronbach’s alpha for the five personality dimensions was as follows: Extraversion (α_Careful_ = 0.69, α_Partially careless_ = 0.52, α_Fully careless_ = 0.56), agreeableness (α_Careful_ = 0.78, α_Partially careless_ = 0.64, α_Fully careless_ = 0.77), conscientiousness (α_Careful_ = 0.77, α_Partially careless_ = 0.62, α_Fully careless_ = 0.72), neuroticism (α_Careful_ = 0.84, α_Partially careless_ = 0.64, α_Fully careless_ = 0.76), and openness to experience (α_Careful_ = 0.76, α_Partially careless_ = 0.53, α_Fully careless_ = 0.62). These consistency values were somewhat lower than those reported by Iller et al. (2020); of course, this result makes sense given that alpha depends on the number of items in the scale, and we used fewer items (Carmines & Zeller, 1979).

#### Direct careless responding measure

We used the same three bogus items as in Study 1. Like the substantive measure, these three items were rated on a Likert scale that ranged from 1 = *completely disagree* to 6 = *completely agree*. For the main analyses, an average score was computed across the three items (α_Careful_ = 0.28, α_Partially careless_ = 0.09, α_Fully careless_ = 0.63). If a respondent’s response was missing, the average score was based on the remaining non-missing responses.

#### Indirect careless responding measures

We used the same indirect careless responding indices as in Study 1. For computing these indirect indices, only the 60 items of the IPIP-5F-30F-R1 were used. Detailed information on how these indices were computed is provided in the supplementary material.

#### Careless responding criterion and analytical procedure

We used the same criterion and analytical procedure as in Study 1.

### Results

#### Manipulation check

To examine whether our manipulation worked, we compared the scores of the five substantive scales and three indirect careless response indices (i.e., average response time per item, IRV, RPR) of the experimental groups with those of a norm group. The comparisons revealed that our manipulation was successful (see supplementary material and Table S10). Thus, we proceeded with the main analyses.

#### Descriptive statistics

The condition-specific correlation matrices as well as the condition-specific means and standard deviations of the substantive measures and careless responding indices are reported in the supplementary material (see Tables S11–S13).

#### Comparisons between the indirect indices

The omnibus test indicated significant inequality between the AUCs for detecting partially careless respondents (*F*[13, 6.5*10^8^] = 11.70, *p* < .001) and between the AUCs for detecting fully careless respondents (*F*[13, 7.0*10^8^] = 26.49, *p* < .001). These global inequalities were then further explored through Bonferroni-corrected pairwise comparisons.

For detecting partially careless respondents (i.e., Careless 33%), the following 11 indices performed better than chance (i.e., the confidence interval of their AUC did not include 0.5): lz, Gnormed, MD, RPR, Outfit MSE, psychometric synonyms, *r*_pbis_, H_t_, infit MSE, INDCHI, and psychometric antonyms (see Table S14). In contrast, average response per item, maximum longstring, and IRV did not perform well in detecting partially careless respondents, and they were outperformed by all other indices. Whereas average response time per item and maximum longstring did not perform better than chance (i.e., the confidence interval of their AUC included 0.5), IRV even screened in the wrong direction (i.e., it was more indicative for careful responding).

For detecting fully careless respondents, the following 12 indices performed better than chance (i.e., the confidence interval of their AUC did not include 0.5): *r*_pbis_, average response time per item, RPR, H_t_, psychometric synonyms, psychometric antonyms, INDCHI, MD, lz, Gnormed, outfit MSE, and infit MSE (see Table S15). Among these effective indices, *r*_pbis_, average response time per item, RPR, and H_t_ tended to be the best, as they significantly outperformed indices with mediocre performance (i.e., MD, lz, Gnormed, outfit MSE, infit MSE). In contrast, maximum longstring and IRV were the only indices that turned out to be ineffective in detecting fully careless responding (i.e., the confidence interval of their AUC included 0.5).

Lastly, we compared each index’s performance in detecting partially careless respondents with its performance in detecting fully careless respondents. For each index, the condition-specific univariate logit estimates (i.e., estimate for Careless 33% and estimate for Careless 100%) were therefore stacked into one model, simultaneously estimated, and tested for equality by using seemingly unrelated estimation (i.e., SUEST; Weesie, 1999). The following nine indices were equally effective (or ineffective in the case of the maximum longstring) in detecting partially and fully careless responding according to the Bonferroni-corrected critical χ^2^ or *F*-value of 8.49 (i.e., α = 0.05/14): RPR(χ^2^[1] = 0.13), INDCHI (χ^2^[1] = 0.59), MD (χ^2^[1] = 2.46), H_t_ (χ^2^[1] = 3.05), psychometric synonyms (χ^2^[1] = 3.49), *r*_pbis_ (χ^2^[1] = 3.83), longstring (χ^2^[1] = 4.64), psychometric antonyms (*F*[1, 292′368.4] = 4.69), and Gnormed (χ^2^[1] = 7.46).

In contrast, the following indices performed better in detecting one specific careless responding form. For one, infit MSE (χ^2^[1] = 8.73), outfit MSE (χ^2^[1] = 9.77), and lz (χ^2^[1] = 9.93) were found to better detect partially careless responding than fully careless responding. For another, average response time per item (χ^2^[1] = 38.59) and IRV (χ^2^[1] = 11.45) were found to better detect fully careless responding than partially careless responding. In the case of IRV, however, it needs to be mentioned that this difference in accuracy did not really reflect an improvement in classification accuracy. It mainly occurred because IRV did not perform that badly (i.e., being as good as chance) when fully careless responding should be detected and not as badly as it did (i.e., being more indicative for careful responding) when partially careless responding should be detected.

#### Comparisons between the average score on bogus items and indirect indices

We then compared the detection performance of each effective indirect index with that of the average score of the three bogus items (see Table 4). For detecting partially careless respondents, the Bonferroni-corrected pairwise comparisons revealed the following results: Whereas INDCHI and psychometric antonyms turned out to be as accurate as the average score on the bogus items, the majority of the examined indices (i.e., lz, Gnormed, MD, RPR, outfit MSE, psychometric synonyms, *r*_pbis_, H_t_, infit MSE) performed significantly better. For detecting fully careless respondents, the Bonferroni-corrected pairwise comparisons revealed the following results: The majority of examined indirect indices (i.e., MD, RPR, psychometric synonyms, *r*_pbis_, H_t_, INDCHI, psychometric antonyms, average response time per item) were as accurate as the average score on the bogus items. Only lz, Gnormed, outfit MSE, and infit MSE had a significantly lower detection accuracy. In addition, we also examined the indices’ incremental validity beyond the average score on bogus items. These estimates are reported in the supplementary material (see Table S16).

**Table 4:** Pairwise comparisons between the average score on bogus items and indirect indices regarding the detection of partially and fully careless responding in Study 2

	Careless 33% ^a^		Careless 100% ^b^	
Index	AUC_[95% CI]_	*F*(1, > 10^6^)	AUC_[95% CI]_	*F*(1, > 10^6^)
Bogus (reference)	.62_[.55, .69]_		.83_[.78, .88]_	
lz	.81_[.75, .86]_	19.52	.70_[.63, .77]_	11.01
Gnormed	.78_[.72, .84]_	15.67	.69_[.62, .76]_	13.02
MD	.77_[.72, .83]_	13.12	.73_[.67, .80]_	6.83
RPR	.77_[.71, .83]_	14.57	.84_[.79, .89]_	0.05
Outfit MSE	.76_[.70, .82]_	11.60	.64_[.57, .72]_	19.91
Synonyms	.76_[.70, .82]_	9.98	.82_[.77, .88]_	0.06
*r*_pbis_	.76_[.69, .82]_	11.34	.86_[.81, .91]_	0.80
H_t_	.76_[.69, .82]_	10.66	.84_[.78, .89]_	0.03
Infit MSE	.75_[.69, .81]_	9.82	.64_[.56, .71]_	20.83
INDCHI	.74_[.68, .81]_	7.93	.78_[.72, .84]_	1.96
Antonyms	.73_[.66, .80]_	6.38	.82_[.76, .87]_	0.19
Time	-	-	.85_[.80, .90]_	0.40

Only indirect indices that turned out to be effective in detecting partially or fully careless responding were examined. Because the antonym index could not be computed for two respondents in the partially careless responding group (i.e., Careless 33%) and for two respondents in the fully careless responding group (i.e., Careless 100%), all reported estimates and test statistics are based on 100 multiply imputed data sets. All indices were coded such that higher scores were more indicative of careless responding. The Bonferroni-corrected critical *F*-value for each of the 23 comparisons was 9.40 (i.e., 11 pairwise comparisons in the partially careless responding condition, 12 pairwise comparisons in the fully careless responding condition); if this value is exceeded, the AUC of bogus items and that of the indirect index can be considered significantly different from each other. AUC = area under the receiver operating characteristic curve; Time = average response time per item; Synonyms = psychometric synonyms; Antonyms = psychometric antonyms; RPR = resampled personal reliability; MD = Mahalanobis distance measure for which the calculation was based on subsamples; *r*_pbis_ = person-total correlation coefficient for which the calculation was based on subsamples; Gnormed = global (i.e., the average of the scale-specific scores was computed) normed Guttman error index for polytomous items; H_t_ = global (i.e., the average of the scale-specific scores was computed) person scalability index; lz = global (i.e., the average of the scale-specific scores was computed) standardized log-likelihood index for polytomous items; Infit MSE = global (i.e., the average of the scale-specific statistics was computed) Rasch model-based infit mean squared error; Outfit MSE = global (i.e., the average of the scale-specific statistics was computed) Rasch model-based outfit mean squared error; INDCHI = global (i.e., the average of the scale-specific scores was computed) individual contribution to model misfit for which the calculation of the two person-specific model log-likelihood values was based on subsamples

^a^ A subsample that contained careful respondents and respondents that had partially careless responding was used to determine the classification accuracy (*n* = 226). ^b^ A subsample that contained careful respondents and respondents that had fully careless responding (*n* = 227) was used to determine the classification accuracy

#### Comparisons between the average score of bogus items and sets of indirect indices

We then compared the performance of three sets of indirect indices with that of the average score on the three bogus items. In the first set, all effective indirect indices were used in combination. In the second set, the three most accurate indices were used in combination. In the third set, the three indices that needed the fewest code lines until the final index was obtained (see supplementary Table S8) were used in combination. For these comparisons, we used linear multiple imputation predictions of logit models as input for the ROC analyses.

For detecting partially careless respondents, all examined sets turned out to have a significantly larger AUC than the average score on bogus items (see Table 5). In addition, the set in which all effective indices were combined also outperformed the average score on bogus items in terms of sensitivity at a false-positive rate of 5%. Whereas this set of indirect indices detected 51% of the partially careless respondents, the average score on bogus items only detected 12% (see Table 5). For detecting fully careless responding, the first and second set turned out to have a significantly larger AUC than the average score on bogus items.

**Table 5:** Pairwise comparisons between the average score on bogus items and sets of indirect indices regarding the detection of partially and fully careless responding in Study 2

	AUC comparison		Sen95 comparison ^c^		Sen99 comparison ^d^	
Index	AUC_[95% CI]_	χ^2^(1)	Sen95	z	Sen99	z
Careless 33% ^a^						
Bogus (reference)	.62_[.55, .69]_		.12		.05	
Set 1 (all effective indirect indices)	.86_[.82, .91]_	37.81	.51	− 4.35	.25	− 1.87
Set 2 (lz, Gnormed, MD)	.81_[.76, .87]_	20.08	.38	− 2.34	.21	− 2.53
Set 3 (MD, *r*_pbis_, lz)	.82_[.76, .88]_	24.74	.30	− 1.66	.11	− 0.71
Careless 100% ^b^						
Bogus (reference)	.83_[.78, .88]_		.47		.39	
Set 1 (all effective indirect indices)	.93_[.91, .97]_	15.79	.72	− 2.74	.52	− 1.34
Set 2 (*r*_pbis_, Time, RPR)	.90_[.86, .94]_	7.07	.64	− 2.48	.43	− 0.44
Set 3 (Time, MD, *r*_pbis_)	.90_[.86, .94]_	6.25	.57	− 1.23	.38	0.09

Only indirect indices that turned out to be effective in detecting careless respondents were examined. The average score on the bogus items could not be computed for one respondent in the careful responding group, because this respondent did not answer any of the bogus items. In addition, the antonym index could not be computed for two respondents in the partially careless responding group (i.e., Careless 33%) and for two respondents in the fully careless responding group (i.e., Careless 100%), because one of the antonym vectors had no variance. As input for the ROC analyses, we used linear multiple imputation predictions (across 100 imputations) of logit models. All indices were coded such that higher scores were more indicative of faking. Set 1 included all effective indirect indices. Set 2 included the three most accurate indices. Set 3 included the three easiest-to-compute indices (i.e., lowest number of code lines required). The Bonferroni-corrected critical χ^2^ value for each of the six AUC comparisons was 6.96 (i.e., three pairwise comparisons in the partially careless responding condition, three pairwise comparisons in the fully careless responding condition); if this value is exceeded, the two AUCs can be considered significantly different from each other. The Bonferroni-corrected critical *z*-value for each of the 12 sensitivity comparisons was 2.87 (i.e., six pairwise comparisons in the partially careless responding condition, six pairwise comparisons in the fully careless responding condition); if this *z*-value is exceeded, the sensitivity of the reference and the sensitivity of the comparison can be considered significantly different from each other. The standard error of the difference between the sensitivities was determined through bootstrapping with 1000 replications. AUC = area under the receiver operating characteristic curve; Time = average response time per item; RPR = resampled personal reliability; MD = Mahalanobis distance measure for which the calculation was based on subsamples; *r*_pbis_ = person-total correlation coefficient for which the calculation was based on subsamples; Gnormed = global (i.e., the average of the scale-specific scores was computed) normed Guttman error index for polytomous items; lz = global (i.e., the average of the scale-specific scores was computed) standardized log-likelihood index for polytomous items

^a^ A subsample that contained careful respondents and respondents that had partially careless responding was used to determine the classification accuracy (*n* = 226). ^b^ A subsample that contained careful respondents and respondents that had fully careless responding (*n* = 227) was used to determine the classification accuracy. ^c^ Sensitivity of the index set at specificity level of 95% (i.e., false-positive rate of 5%). ^d^ Sensitivity of the index set at specificity level of 99% (i.e., false-positive rate of 1%)

#### Detection performance of indices at bidirectionally and unidirectionally keyed items

We then addressed research question 5 and compared the indices’ performance in Study 1 (in which items of same unidimensional construct were positively and negatively keyed) with their performance in Study 2 (in which all items of same unidimensional construct were scored in the same direction). We therefore appended the data of Study 2 to the data of Study 1 and used the *by* option of the *roccomp* command in Stata.

However, none of the pairwise AUC comparisons for detecting partially and fully careless respondents reached the Bonferroni-corrected critical value of 9.88 (i.e., α = 0.05/30). Nevertheless, it seemed legitimate to claim that three indices functioned differently across the two studies, especially if the confidence intervals of the indices’ AUCs were used for making the judgment. For instance, when partially careless responding should be detected, the average response time per item performed significantly better than chance in Study 1 and did not do so in Study 2 (see AUCs reported in Table S5 and Table S14, respectively). For another, when fully careless responding should be detected, the maximum longstring and the IRV performed significantly better than chance in Study 1 and did not do so in Study 2 (see AUCs reported in Table S6 and Table S15, respectively).

## Study 3

In Study 3, the manipulation turned out to be unsuccessful and thus prevented us from conducting the main analyses. For transparency reasons, and to inform researchers interested in conducting studies on careless responding in online populations like MTurk, we report the methods of Study 3 and the results of the manipulation check in the supplementary material (including Table S17).

## Study 4

Studies 1 and 2 showed that indirect indices are effective in detecting different types of careless responding and even outperform direct measures (i.e., bogus items). Study 4 aimed to replicate these findings with a sample of Prolific workers and with a shift in the rating perspective (i.e., respondents were asked to rate their own personality). In addition, we used a different direct careless responding measure—the eight items of the infrequency scale by Huang et al. (2015). Thus, Study 4 allowed us to address research questions 1 to 4 and to examine how the comparison between direct and indirect careless responding measures turns out if a validated bogus item scale is used that contains more than twice as many bogus items as in Studies 1 and 2.

### Methods

#### Participants

The data for Study 4 were gathered in January 2024. The participants were Prolific workers who had Prolific accounts in the United States or Great Britain and an approval rate of at least 98%. On the Prolific website and the starting web page of the online survey, Prolific workers were informed that the study would examine the impact of careless (i.e., unmotivated) responding on the quality of personality test results. For this purpose, potential participants would have to complete our personality questionnaire twice. In the first session, all participants would be asked to complete the survey accurately. In the second session, however, some participants would be asked to imitate careless responding and would be instructed to complete the survey or parts thereof according to our specific “careless responding” instructions, whereas others would be instructed to complete the questionnaire accurately. Lastly, we announced that we would pay each participant £3 for participating in our study.

A total of *N* = 481 Prolific workers were recruited for Study 4. The participants in this sample were on average 44.30 years old (*SD* = 13.97) and half of them were women (*n* = 249, 51.8%). Almost two thirds of the participants (*n* = 292, 60.7%) had a bachelor’s or higher degree, and 40% of them had an associate’s degree or a lower education level (*n* = 189, 39.3%). Approximately three quarters of the participants were currently employed (*n* = 347, 72.1%).

#### Experimental conditions

At the first measurement, which also included the four sociodemographic variables, all participants were instructed to respond to the questionnaire items as they applied to them. They were further informed that there were no right or wrong answers and that they just had to respond to each item as honestly as possible.

Immediately after the participants had completed the first measurement, they completed the same questionnaire again. At this second measurement, however, they were randomly assigned to one of the four experimental conditions—either to one of the two careful responding conditions (*n*_Careful unincentivized_ = 120; *n*_Careful incentivized_ = 121)^1^ or to one of the two careless responding conditions (i.e., partially careless—careless responding to the last third of the questionnaire items [*n*_Partially careless_ = 118], fully careless—careless responding to all questionnaire items [*n*_Fully careless_ = 122]).

Participants assigned to the *unincentivized* careful responding condition were again instructed to respond to the questionnaire items as they applied to them and as honestly as possible. Participants assigned to the *incentivized* careful responding condition received the same instruction as participants in the unincentivized careful responding condition. However, they were additionally told that we acknowledged that answering the same questions with the same instruction may be a less attractive task, which is why they would receive a bonus of £1 as compensation, so that they would nevertheless pay full attention when completing the second measurement. In contrast, participants in the *partially careless* responding condition were asked to complete the first survey part (i.e., 66% of the items) accurately and honestly and the second survey part (i.e., 33% of the items) with the following instruction: They should pretend that they were not really interested in the survey and just wanted to get the job done as quickly as possible. Thus, they should deliberately respond without effort to every item of the remaining questionnaire. They were further informed that there would be no risk of any penalty and that we would pay them in any case. Participants in the *fully careless* responding condition received the following instruction: They should pretend that they were not really interested in the survey and just wanted to get this job done as quickly as possible. Thus, they should deliberately respond without effort to every item of the questionnaire. They were further informed that there would be no risk of any penalty and that we would pay them in any case.

#### Substantive measures

At both measurements, the participants completed the Big Five Inventory–2 (BFI-2; Soto & John, 2017), which contains the five trait scales extraversion (α_Careful unincentivized_ = 0.87_t1_/0.88_t2_, α_Careful incentivized_ = 0.88_t1_/0.89_t2_, α_Partially careless_ = 0.89_t1_/0.63_t2_, α_Fully careless_ = 0.86_t1_/NA_t2_), agreeableness (α_Careful unincentivized_ = 0.86_t1_/0.87_t2_, α_Careful incentivized_ = 0.83_t1_/0.84_t2_, α_Partially careless_ = 0.81_t1_/0.70_t2_, α_Fully careless_ = 0.88_t1_/0.46_t2_), conscientiousness (α_Careful unincentivized_ = 0.91_t1_/0.91_t2_, α_Careful incentivized_ = 0.90_t1_/0.92_t2_, α_Partially careless_ = 0.91_t1_/0.72_t2_, α_Fully careless_ = 0.91_t1_/0.62_t2_), negative emotionality (α_Careful unincentivized_ = 0.94_t1_/0.94_t2_, α_Careful incentivized_ = 0.94_t1_/0.94_t2_, α_Partially careless_ = 0.91_t1_/0.77_t2_, α_Fully careless_ = 0.90_t1_/0.27_t2_), and open-mindedness (α_Careful unincentivized_ = 0.87_t1_/0.89_t2_, α_Careful incentivized_ = 0.89_t1_/0.91_t2_, α_Partially careless_ = 0.89_t1_/0.77_t2_, α_Fully careless_ = 0.92_t1_/0.29_t2_). The respondents rated all 60 bidirectionally keyed items (i.e., 30 positively and 30 negatively worded items) of the BFI-2 on a Likert scale ranging from 1 = *completely disagree* to 6 = *completely agree.*

#### Direct careless responding measure

We used Huang et al.’s (2015) infrequency scale as a direct careless responding measure. The eight bogus items of this scale were embedded in the regular questionnaire and randomly displayed. As with the substantial items, respondents rated the eight bogus items on a Likert scale from 1 = *completely disagree* to 6 = *completely agree*. For the main analyses, an average score was computed across the eight items (α_Careful unincentivized_ = 0.88_t1_/0.97_t2_, α_Careful incentivized_ = 0.90_t1_/0.92_t2_, α_Partially careless_ = 0.68_t1_/0.53_t2_, α_Fully careless_ = 0.78_t1_/0.88_t2_). If a respondent’s response was missing, the average score was based on the remaining non-missing responses. However, this average score could not be computed for one respondent in the fully careful responding group at the second measurement, because they did not answer any of the bogus items.

#### Indirect careless responding measures

We used the same indirect careless responding indices as in Studies 1 and 2. For computing these indirect indices, only the 60 items of the BFI-2 were used. Detailed information on how these indices were computed is provided in the supplementary material.

#### Careless responding criterion and analytical procedure

The within-subject design of Study 4 allowed us to examine whether the direct and indirect careless response indices correctly distinguished between ratings that were given by careless responders (i.e., rating at t2) and ratings that were given by careful responders (i.e., rating at t1). Accordingly, only participants that were assigned to the partially or fully careless responding condition at the second measurement were used for examining the indices’ detection accuracy.^2^ As in the previous studies, we plotted for every index (and selected combinations of indices) an ROC curve (e.g., Swets, 1986) and examined the corresponding AUC. We used the tie-corrected nonparametric method (i.e., trapezoidal approximation) with bootstrap standard errors (i.e., 1000 replications) for estimating the AUCs and standard errors. Because the data for all ROC analyses were arranged in long format, the standard errors were additionally adjusted for clustering. All these analyses were performed in Stata 18 (StataCorp, 2021).

### Results

#### Manipulation check

To examine whether the respondents followed our response instructions, we compared the t1–t2 mean differences of the five substantive scales and of three indirect careless response indices (i.e., average response time per item, IRV, RPR) across the four response conditions. The comparisons revealed that our manipulation was successful (see supplementary material and Table S18). Thus, we proceeded with the main analyses.

#### Descriptive statistics

The condition-specific correlation matrices at the first and second measurement as well as the condition-specific means and standard deviations at the first and second measurement of the substantive measures and careless responding indices are reported in the supplementary material (see Tables S19–S26).

#### Comparisons between the indirect indices

The omnibus test indicated significant inequality between the AUCs for detecting partially careless responding (χ^2^[13] = 966.47, *p* < .001) and between the AUCs for detecting fully careless responding (*F*[13, 35,601] = 43.29, *p* < .001). These global inequalities were then further explored through Bonferroni-corrected pairwise comparisons.

For detecting partially careless responding (i.e., Careless 33%), the following 12 indices turned out to be effective (i.e., the confidence interval of their AUC did not include 0.5): MD, psychometric synonyms, RPR, psychometric antonyms, Gnormed, *r*_pbis_, H_t_, average response time per item, outfit MSE, lz, infit MSE, and maximum longstring (see Table S27 for AUCs). Of these effective indices, the MD and the three within-person consistency indices (psychometric synonyms, psychometric antonyms, and RPR) tended to be the best: They significantly outperformed at least five of the other eight effective indices in terms of AUC. In contrast, INDCHI and IRV did not perform well in detecting partially careless responding. The accuracy of these indices was not better than chance (i.e., the confidence interval of their AUC included 0.5).

For detecting fully careless responding, all examined indices except INDCHI turned out to be effective (see Table S28). Of the 13 effective indices, psychometric synonyms, *r*_pbis_, and RPR tended to be the best-performing indices. They significantly outperformed seven of the other ten effective indices in terms of AUC.

Lastly, we addressed research question 4 and compared each index’s performance in detecting partially careless respondents with its performance in detecting fully careless respondents. For each index, the condition-specific univariate logit estimates (i.e., estimate for Careless 33% and estimate for Careless 100%) were therefore stacked into one model, simultaneously estimated, and tested for equality by using seemingly unrelated estimation (SUEST; Weesie, 1999). Although we observed a tendency for several indices to perform better when fully instead of partially careless responding had to be detected, the Bonferroni-corrected critical value (i.e., a χ^2^ of 8.49 or a *F*-value of 8.56; α = 0.05/14) was only reached in the case of the IRV. This index was significantly better in detecting fully careless responding than partially careless responding (χ^2^[1] = 11.04). The remaining 13 indices were equally effective (or ineffective in the case of INDCHI) in detecting partially and fully careless responding according to the Bonferroni-corrected critical value: INDCHI (χ^2^[1] = 0.04), RPR(χ^2^[1] = 0.06), Gnormed (χ^2^[1] = 0.37), H_t_ (χ^2^[1] = 0.38), psychometric antonyms (*F*[1, 554] = 0.62), psychometric synonyms (*F*[1, 128,642.2] = 0.93), maximum longstring (χ^2^[1] = 2.12), MD (χ^2^[1] = 2.61), outfit MSE (χ^2^[1] = 3.86), lz (χ^2^[1] = 4.80), *r*_pbis_ (*F*[1, 3.9*10^7^] = 5.01), infit MSE (χ^2^[1] = 5.51), and average response time per item (χ^2^[1] = 8.04).

#### Comparisons between the average score of bogus items and indirect indices

We then compared the detection performance of each effective indirect index with that of the average score of the eight bogus items (see Table 6). For detecting partially careless responding, the Bonferroni-corrected pairwise comparisons revealed the following results: The MD and the three within-person consistency indices (psychometric synonyms, psychometric antonyms, RPR) turned out to be as accurate as the average score of the bogus items, but the majority of the examined indices (i.e., Gnormed, *r*_pbis_, H_t_, average response time per item, outfit MSE, lz, infit MSE, maximum longstring) performed significantly worse. For detecting fully careless responding, the Bonferroni-corrected pairwise comparisons revealed the following results: Psychometric synonyms, *r*_pbis_, and psychometric antonyms turned out to be as accurate as the average score of bogus items, but the majority of examined indirect indices (i.e., MD, RPR, Gnormed, H_t_, average response time per item, outfit MSE, lz, infit MSE, maximum longstring, and IRV) performed significantly worse. In addition, we also examined the indices’ incremental validity beyond the average score on bogus items. These estimates are reported in the supplementary material (see Table S29).

**Table 6:** Pairwise comparisons between the average score on bogus items and indirect indices regarding the detection of partially and fully careless responding in Study 4

Index	Careless 33%^a^		Careless 100%^b^	
	AUC_[95% CI]_	χ^2^(1)	AUC_[95% CI]_	*F*(1, > 262)
Bogus (reference)	.89_[.85, .93]_		.98_[.96, .998]_	
MD	.88_[.84, .91]_	0.16	.86_[.81, .91]_	22.99
Synonyms	.85_[.80, .89]_	2.24	.94_[.91, .98]_	8.22
RPR	.84_[.80, .89]_	3.17	.93_[.90, .97]_	13.69
Antonyms	.80_[.76, .85]_	9.14	.90_[.84, .96]_	8.14
Gnormed	.78_[.74, .83]_	12.08	.83_[.78, .88]_	26.55
*r*_pbis_	.77_[.73, .81]_	19.09	.94_[.91, .97]_	8.75
H_t_	.73_[.68, .79]_	21.03	.70_[.65, .76]_	89.74
Time	.69_[.65, .74]_	49.94	.91_[.88, .95]_	13.10
Outfit MSE	.59_[.53, .64]_	76.31	.69_[.63, .76]_	69.05
lz	.58_[.52, .63]_	82.60	.72_[.65, .78]_	65.32
Infit MSE	.58_[.53, .63]_	80.58	.69_[.62, .75]_	72.05
Longstring	.58_[.52, .64]_	77.77	.70_[.64, .76]_	87.32
IRV	-	-	.60_[.53, .66]_	119.88

Only indirect indices that turned out to be effective in detecting partially or fully careless responding were examined. Because four indices (synonyms, antonyms, *r*_pbis_, average score of bogus items) could not be computed for every participant of the fully careless responding condition, all reported estimates and test statistics for the fully careless responding condition are based on 100 multiply imputed data sets. All indices were coded such that higher scores were more indicative of careless responding. For the 25 comparisons (i.e., 12 pairwise comparisons in the partially careless responding condition, 13 pairwise comparisons in the fully careless responding condition), we used a Bonferroni-corrected critical *F* and χ^2^ value of 9.74; this critical value was obtained by using an *F*-distribution with one degree of freedom for the numerator and 262 degrees of freedom for the denominator and a probability value of 1 − (.05/25); if the critical value of 9.74 is exceeded, the AUC of bogus items and that of the indirect index can be considered significantly different from each other. AUC = area under the receiver operating characteristic curve; Time = average response time per item; Longstring = maximum longstring; IRV = intra-individual response variability; Synonyms = psychometric synonyms; Antonyms = psychometric antonyms; RPR = resampled personal reliability; MD = Mahalanobis distance measure for which the calculation was based on subsamples; *r*_pbis_ = person-total correlation coefficient for which the calculation was based on subsamples; Gnormed = global (i.e., the average of the scale-specific scores was computed) normed Guttman error index for polytomous items; H_t_ = global (i.e., the average of the scale-specific scores was computed) person scalability index; lz = global (i.e., the average of the scale-specific scores was computed) standardized log-likelihood index for polytomous items; Infit MSE = global (i.e., the average of the scale-specific statistics was computed) Rasch model-based infit mean squared error; Outfit MSE = global (i.e., the average of the scale-specific statistics was computed) Rasch model-based outfit mean squared error

^a^ The ratings of the first and second measurement of the partially careless responding condition (*n* = 118) were used to determine the classification accuracy. ^b^ The ratings of the first and second measurement of the fully careless responding condition (*n* = 122) were used to determine the classification accuracy

#### Comparisons between the average score of bogus items and sets of indirect indices

We then compared the performance of three sets of indirect indices with that of the average score of the eight bogus items. In the first set, all effective indirect indices were used in combination. In the second set, the three most accurate indices were used in combination. In the third set, the three indices that needed the fewest code lines until the final index was obtained (see supplementary Table S8) were used in combination. We used linear predictions of logit models as input for the ROC analyses in the partially careless responding condition and linear multiple imputation predictions of logit models (across 72 to 100 imputations because convergence was not achieved for estimating logit models in some imputation samples) as input for the ROC analyses in the fully careless responding condition.

For detecting partially careless responding, the analyses revealed the following: Although the second and third set were as accurate as the average score of bogus items, the first set performed significantly better than the average score of bogus items in terms of AUC and sensitivity at a false-positive rate of 1% (see Table 7). The difference in sensitivity at a false-positive rate of 1% between the first set and average score of bogus items was impressive. Moreover, the first set detected every partially careless respondent; however, the average score of bogus items only detected 17% of them.

**Table 7:** Pairwise comparisons between the average score on bogus items and sets of indirect indices regarding the detection of partially and fully careless responding in Study 4

	AUC comparison		Sen95 comparison ^c^		Sen99 comparison ^d^	
Index	AUC_[95% CI]_	χ^2^(1)	Sen95	z	Sen99	z
Careless 33% ^a^						
Bogus (reference)	.89_[.85, .93]_		.58		.17	
Set 1 (all effective indirect indices)	1_[1, 1]_	31.29	1	− 2.82	1	− 7.45
Set 2 (MD, Synonyms, RPR)	.91_[.87, .94]_	0.73	.53	0.34	.47	− 2.56
Set 3 (Time, Longstring, MD)	.90_[.86, .94]_	0.24	.68	− 0.61	.48	− 2.39
Careless 100% ^b^						
Bogus (reference)	.98_[.96, .998]_		.95		.85	
Set 1 (all effective indirect indices)	1_[1, 1]_	4.75	1	− 1.78	1	− 3.74
Set 2 (Synonyms, *r*_pbis_, RPR)	.96_[.93, .99]_	6.68	.93	0.50	.92	− 0.28
Set 3 (Time, IRV, Longstring)	.93_[.89, .96]_	10.23	.75	4.20	.70	1.75

Only indirect indices that turned out to be effective in detecting careless responding were examined. We used linear predictions of logit models as input for the ROC analyses in the partially careless responding condition and linear multiple imputation predictions (across 72 to 100 imputations [convergence was not achieved for estimating logit models in some imputation samples]) of logit models as input for the ROC analyses in the fully careless responding condition. All indices were coded such that higher scores were more indicative of faking. Set 1 included all effective indirect indices. Set 2 included the three most accurate indices. Set 3 included the three easiest-to-compute indices (i.e., lowest number of code lines required). The Bonferroni-corrected critical χ^2^ value for each of the six AUC comparisons was 6.96 (i.e., three pairwise comparisons in the partially careless responding condition, three pairwise comparisons in the fully careless responding condition); if this value is exceeded, the two AUCs can be considered significantly different from each other. The Bonferroni-corrected critical *z*-value for each of the 12 sensitivity comparisons was 2.87 (i.e., six pairwise comparisons in the partially careless responding condition, six pairwise comparisons in the fully careless responding condition); if this *z*-value is exceeded, the sensitivity of the reference and the sensitivity of the comparison can be considered significantly different from each other. The standard error of the difference between the sensitivities was determined through bootstrapping with 1000 replications. AUC = area under the receiver operating characteristic curve; Time = average response time per item; Longstring = maximum longstring; IRV = intra-individual response variability; Synonyms = psychometric synonyms; RPR = resampled personal reliability; MD = Mahalanobis distance measure for which the calculation was based on subsamples; *r*_pbis_ = person-total correlation coefficient for which the calculation was based on subsamples

^a^ The ratings of the first and second measurement of the partially careless responding condition (*n* = 118) were used to determine the classification accuracy. ^b^ The ratings of the first and second measurement of the fully careless responding condition (*n* = 122) were used to determine the classification accuracy. ^c^ Sensitivity of the index set at specificity level of 95% (i.e., false-positive rate of 5%). ^d^ Sensitivity of the index set at specificity level of 99% (i.e., false-positive rate of 1%)

For detecting fully careless responding, the analyses revealed the following results (see Table 7): First, the second set turned out to be as accurate as the average score of bogus items in terms of AUC and sensitivity. Second, even though the third set reached high levels of AUC (0.93) and sensitivity (0.75 at a false-positive rate of 5% and 0.70 at a false-positive rate of 1%), it performed significantly worse than the average score of bogus items, which reached an almost perfect detection accuracy. Lastly, only the first set outperformed the average score of bogus items. As for detecting partially careless responding, the first set reached perfect detection accuracy and performed significantly better than the average score of bogus items in terms of sensitivity at a false-positive rate of 1%. Whereas the first set detected every fully careless respondent, the average score of bogus items detected “only” 85% of them.

## Study 5

Studies 1, 2, and 4 showed that indirect indices were effective in detecting different types of careless responding and may even outperform different sets of bogus items, and thus one popular type of direct careless responding measure. In Study 5 we aimed to replicate these findings with another sample of Prolific workers and another popular direct careless responding measure: instructed response items. Thus, Study 5 allowed us to address research questions 1 to 4 and to examine how the comparison between direct and indirect careless responding measures turns out if an index of eight instructed response items is used as a direct careless responding measure instead of an average score of bogus items.

### Methods

#### Participants

The data for Study 5 were gathered in January 2024. The participants were Prolific workers who had Prolific accounts in the United States or Great Britain and an approval rate of at least 98%. In addition, they were excluded from participation if they were already participating in Study 4. On the Prolific website and the starting web page of the online survey, Prolific workers were informed that the study would examine the impact of careless (unmotivated) responding on the quality of personality test results. For this purpose, potential participants would have to complete our personality questionnaire twice. In the first session, all participants would be asked to complete the survey accurately. In the second session, some participants would be asked to imitate careless responding and would be instructed to complete the survey or parts thereof according to our specific “careless responding” instructions, while others would be instructed to complete the questionnaire accurately. Lastly, we announced that we would pay every participant £3 for participating in our study.

A total of *N* = 481 Prolific workers were recruited for Study 5. The participants in this sample were on average 42.40 years old (*SD* = 13.76), and almost half of them were women (*n* = 225, 46.8%). More than half of the participants (*n* = 279, 58.0%) had a bachelor’s or higher degree, and a bit more than 40% of them had an associate’s degree or a lower education level (*n* = 202, 42.0%). Approximately three quarters of the participants were currently employed (*n* = 363, 75.5%).

#### Experimental conditions

The experimental conditions in Study 5 were identical to those in Study 4. As in Study 4, the participants in Study 5 were randomly assigned to one of the four experimental conditions—either to one of the two careful responding conditions (*n*_Careful unincentivized_ = 120; *n*_Careful incentivized_ = 120) or to one of the two careless responding conditions (i.e., partially careless—careless responding to the last third of the questionnaire items [*n*_Partially careless_ = 120], fully careless—careless responding to all questionnaire items [*n*_Fully careless_ = 121]).

#### Substantive measures

At both measurements, the participants completed the BFI-2 (Soto & John, 2017) which contains the five trait scales extraversion (α_Careful unincentivized_ = 0.86_t1_/0.87_t2_, α_Careful incentivized_ = 0.88_t1_/0.90_t2_, α_Partially careless_ = 0.85_t1_/0.73_t2_, α_Fully careless_ = 0.90_t1_/0.08_t2_), agreeableness (α_Careful unincentivized_ = 0.88_t1_/0.93_t2_, α_Careful incentivized_ = 0.86_t1_/0.89_t2_, α_Partially careless_ = 0.84_t1_/0.67_t2_, α_Fully careless_ = 0.85_t1_/0.39_t2_), conscientiousness (α_Careful unincentivized_ = 0.89_t1_/0.92_t2_, α_Careful incentivized_ = 0.90_t1_/0.92_t2_, α_Partially careless_ = 0.91_t1_/0.78_t2_, α_Fully careless_ = 0.91_t1_/0.41_t2_), negative emotionality (α_Careful unincentivized_ = 0.94_t1_/0.94_t2_, α_Careful incentivized_ = 0.94_t1_/0.94_t2_, α_Partially careless_ = 0.93_t1_/0.79_t2_, α_Fully careless_ = 0.94_t1_/0.33_t2_), and open-mindedness (α_Careful unincentivized_ = 0.86_t1_/0.88_t2_, α_Careful incentivized_ = 0.90_t1_/0.91_t2_, α_Partially careless_ = 0.90_t1_/0.79_t2_, α_Fully careless_ = 0.87_t1_/0.08_t2_). The respondents rated all 60 bidirectionally keyed items (i.e., 30 positively and 30 negatively worded items) of the BFI-2 on a Likert scale ranging from 1 = *completely disagree* to 6 = *completely agree.*

#### Direct careless responding measure

We used eight instructed response items as a direct careless responding measure (e.g., Meade & Craig, 2012). These eight instructed response items were embedded in the regular questionnaire and randomly displayed. For each of the eight items, the respondents were instructed to select one particular response option on a six-point Likert scale from 1 = *completely disagree* to 6 = *completely agree*. If the instructed response option was chosen, the participant’s response was coded as 0; if uninstructed response option was chosen, the participant’s response was coded as 1. For main analyses, an average score was computed across the eight items (α_Careful unincentivized_ = NA_t1_/NA_t2_, α_Careful incentivized_ = NA_t1_/0.01_t2_, α_Partially careless_ = 0.01_t1_/0.26_t2_, α_Fully careless_ = 0.45_t1_/0.60_t2_).^3^ If a respondent’s response was missing, the average score was based on the remaining non-missing responses.

#### Indirect careless responding measures

We used the same indirect careless responding indices as in Studies 1, 2, and 4. For computing these indirect indices, only the 60 items of the BFI-2 were used. Detailed information on how these indices were computed is provided in the supplementary material.

#### Careless responding criterion and analytical procedure

We used the same criterion and analysis procedure as in Study 4.

### Results

#### Manipulation check

To examine whether the respondents followed our response instructions, we compared the t1–t2 mean differences of the five substantive scales and of three indirect careless response indices (i.e., average response time per item, IRV, RPR) across the four response conditions. The comparisons revealed that our manipulation was successful (see supplementary material and Table S30). Thus, we proceeded with the main analyses.

#### Descriptive statistics

The condition-specific correlation matrices at the first and second measurement as well as the condition-specific means and standard deviations at the first and second measurement of the substantive measures and careless responding indices are reported in the supplementary material (see Tables S31–S38).

#### Comparisons between the indirect indices

The omnibus test indicated significant inequality between the AUCs for detecting partially careless responding (χ^2^[13] = 931.49, *p* < .001) and between the AUCs for detecting fully careless responding (*F*[13, 99′423.2] = 39.95, *p* < .001). These global inequalities were then further explored through Bonferroni-corrected pairwise comparisons.

For detecting partially careless responding (i.e., Careless 33%), the following indices turned out to be effective (i.e., the confidence interval of their AUC did not include 0.5): psychometric synonyms, MD, RPR, psychometric antonyms, Gnormed, H_t_, *r*_pbis_, average response time per item, and maximum longstring (see Table S39 for AUCs). Of these nine effective indices, psychometric synonyms, MD, and RPR tended to be the best: They significantly outperformed four of the other nine effective indices in terms of AUC. In contrast, the following five indices did not perform well in detecting partially careless responding: IRV, infit MSE, outfit MSE, lz, and INDCHI. The accuracy of these indices was not better than chance (i.e., the confidence interval of their AUC included 0.5).

For detecting fully careless responding, all examined indices turned out to be effective (see Table S40). Among the 14 effective indices, the three within-person consistency indices (psychometric synonyms, psychometric antonyms, and RPR), *r*_pbis_, average response time per item, and MD tended to perform best: They significantly outperformed seven to eight other effective indices in terms of AUC.

Lastly, we addressed research question 4 and compared each index’s performance in detecting partially careless respondents with its performance in detecting fully careless respondents. For each index, the condition-specific univariate logit estimates (i.e., estimate for Careless 33% and estimate for Careless 100%) were therefore stacked into one model, simultaneously estimated, and tested for equality by using seemingly unrelated estimation (SUEST; Weesie, 1999). The following seven indices were equally effective in detecting partially and fully careless responding according to the Bonferroni-corrected critical χ^2^ of 8.49 or *F*-value of 8.53 (i.e., α = 0.05/14): H_t_ (χ^2^[1] = 0.08), psychometric synonyms (*F*[1, 7′974.6] = 0.35), MD (χ^2^[1] = 0.53), RPR(χ^2^[1] = 0.91), Gnormed (χ^2^[1] = 2.20), maximum longstring (χ^2^[1] = 3.16), and psychometric antonyms (*F*[1, 1096.8] = 5.98). In contrast, the following seven indices were more effective when fully instead of partially careless responding had to be detected: INDCHI (χ^2^[1] = 9.37), infit MSE (χ^2^[1] = 11.70), outfit MSE (χ^2^[1] = 12.47), IRV (χ^2^[1] = 13.40), average response time per item (χ^2^[1] = 13.48), lz (χ^2^[1] = 18.17), and *r*_pbis_ (*F*[1, 1.7*10^6^] = 18.84).

#### Comparisons between the average score of instructed response items and indirect indices

We then compared the detection performance of each effective indirect index with that of the average score of the eight instructed response items (see Table 8). For detecting partially careless responding, the Bonferroni-corrected pairwise comparisons revealed the following results: Of the nine effective indirect indices, eight of them performed as accurately as the average score of the instructed response items. The only exception was maximum longstring, which was significantly less accurate than the average score of instructed response items.

**Table 8:** Pairwise comparisons between the average score of instructed response items and indirect indices regarding the detection of partially and fully careless responding in Study 5

	Careless 33% ^a^		Careless 100% ^b^	
Index	AUC_[95% CI]_	χ^2^(1)	AUC_[95% CI]_	*F*(1, > 2083.9)
Instructed responses (reference)	.75_[.71, .80]_		.84_[.79, .88]_	
Synonyms	.82_[.77, .87]_	5.60	.90_[.86, .95]_	5.13
MD	.81_[.77, .86]_	4.25	.88_[.83, .92]_	2.06
RPR	.81_[.76, .85]_	3.34	.93_[.90, .96]_	14.94
Antonyms	.74_[.70, .80]_	0.03	.90_[.85, .95]_	4.35
Gnormed	.72_[.68, .77]_	0.91	.84_[.79, .89]_	0.00
H_t_	.71_[.66, .77]_	1.12	.73_[.68, .79]_	8.77
*r*_pbis_	.70_[.66, .74]_	2.76	.91_[.88, .95]_	7.48
Time	.68_[.64, .72]_	5.72	.92_[.88, .95]_	10.68
Longstring	.59_[.54, .65]_	17.83	.69_[.63, .75]_	17.61
IRV	-	-	.66_[.60, .72]_	21.96
Infit MSE	-	-	.66_[.60, .73]_	20.64
Outfit MSE	-	-	.67_[.60, .74]_	19.54
lz	-	-	.71_[.65, .79]_	12.58
INDCHI	-	-	.64_[.57, .71]_	21.55

Only indirect indices that turned out to be effective in detecting partially or fully careless responding were examined. Because three indices (synonyms, antonyms, *r*_pbis_) could not be computed for every participant of the fully careless responding condition, all reported estimates and test statistics for the fully careless responding condition are based on 100 multiply imputed data sets. All indices were coded such that higher scores were more indicative of careless responding. For the 22 comparisons (i.e., nine pairwise comparisons in the partially careless responding condition, 13 pairwise comparisons in the fully careless responding condition), we used a Bonferroni-corrected critical *F* and χ^2^ value of 9.34; this critical value was obtained by using an *F*-distribution with one degree of freedom for the numerator and 2083.9 degrees of freedom for the denominator and a probability value of 1 − (.05/25); if the critical value of 9.34 is exceeded, the AUC of instructed response items and that of the indirect index can be considered significantly different from each other. AUC = area under the receiver operating characteristic curve; Time = average response time per item; Longstring = maximum longstring; IRV = intra-individual response variability; Synonyms = psychometric synonyms; Antonyms = psychometric antonyms; RPR = resampled personal reliability; MD = Mahalanobis distance measure for which the calculation was based on subsamples; *r*_pbis_ = person-total correlation coefficient for which the calculation was based on subsamples; Gnormed = global (i.e., the average of the scale-specific scores was computed) normed Guttman error index for polytomous items; H_t_ = global (i.e., the average of the scale-specific scores was computed) person scalability index; lz = global (i.e., the average of the scale-specific scores was computed) standardized log-likelihood index for polytomous items; Infit MSE = global (i.e., the average of the scale-specific statistics was computed) Rasch model-based infit mean squared error; Outfit MSE = global (i.e., the average of the scale-specific statistics was computed) Rasch model-based outfit mean squared error; INDCHI = global (i.e., the average of the scale-specific scores was computed) individual contribution to model misfit for which the calculation of the two person-specific model log-likelihood values was based on subsamples

^a^ The ratings of the first and second measurement of the partially careless responding condition (*n* = 120) were used to determine the classification accuracy. ^b^ The ratings of the first and second measurement of the fully careless responding condition (*n* = 121) were used to determine the classification accuracy

For detecting fully careless responding, the Bonferroni-corrected pairwise comparisons revealed the following: First, two indices performed significantly better than the average score of instructed response items—RPR and average response time per item. Second, a group of six indices (psychometric synonyms, psychometric antonyms, MD, Gnormed, H_t_, *r*_pbis_) turned out to be as accurate as the average score of the instructed response items. Third, a group of another six indices (maximum longstring, IRV, infit MSE, outfit MSE, lz, INDCHI) performed significantly worse than the average score of instructed response items. In addition, we also examined the indices’ incremental validity beyond the average score of instructed response items. These estimates are reported in the supplementary material (see Table S41).

#### Comparisons between the average score of instructed response items and sets of indirect indices

We then compared the performance of three sets of indirect indices with that of the average score of the eight instructed response items (see Table 9). In the first set, all effective indirect indices were used in combination. In the second set, the three most accurate indices were used in combination. In the third set, the three indices that needed the fewest code lines until the final index was obtained (see supplementary Table S8) were used in combination. We used linear predictions of logit models as input for the ROC analyses in the partially careless responding condition and linear multiple imputation predictions of logit models (across 69 to 100 imputations because convergence was not achieved for estimating logit models in some imputation samples) as input for the ROC analyses in the fully careless responding condition.

**Table 9:** Pairwise comparisons between the average score of instructed response items and sets of indirect indices regarding the detection of partially and fully careless responding in Study 5

	AUC comparison		Sen95 comparison ^c^		Sen99 comparison ^d^	
Index	AUC_[95% CI]_	χ^2^(1)	Sen95	z	Sen99	z
Careless 33% ^a^						
Instructed responses (reference)	.75_[.71, .80]_		.54		.35	
Set 1 (all effective indirect indices)	.90_[.86, .93]_	29.13	.61	− 0.68	.38	− 0.29
Set 2 (Synonyms, MD, RPR)	.86_[.81, .90]_	12.95	.39	1.36	.32	0.43
Set 3 (Time, Longstring, MD)	.84_[.80, .88]_	11.18	.38	1.46	.13	2.35
Careless 100% ^b^						
Instructed responses (reference)	.84_[.79, .88]_		.69		.58	
Set 1 (all effective indirect indices)	1_[1, 1]_	61.45	1	− 7.29	1	− 7.95
Set 2 (RPR, Time, *r*_pbis_)	.96_[.94, .99]_	31.87	.89	− 4.13	.88	− 5.31
Set 3 (Time, IRV, Longstring)	.94_[.91, .97]_	22.29	.83	− 2.77	.70	− 1.39

Only indirect indices that turned out to be effective in detecting careless responding were examined. We used linear predictions of logit models as input for the ROC analyses in the partially careless responding condition and linear multiple imputation predictions (across 69 to 100 imputations [convergence was not achieved for estimating logit models in some imputation samples]) of logit models as input for the ROC analyses in the fully careless responding condition. All indices were coded such that higher scores were more indicative of faking. Set 1 included all effective indirect indices. Set 2 included the three most accurate indices. Set 3 included the three easiest-to-compute indices (i.e., lowest number of code lines required). The Bonferroni-corrected critical χ^2^ value for each of the six AUC comparisons was 6.96 (i.e., three pairwise comparisons in the partially careless responding condition, three pairwise comparisons in the fully careless responding condition); if this value is exceeded, the two AUCs can be considered significantly different from each other. The Bonferroni-corrected critical *z*-value for each of the 12 sensitivity comparisons was 2.87 (i.e., six pairwise comparisons in the partially careless responding condition, six pairwise comparisons in the fully careless responding condition); if this *z*-value is exceeded, the sensitivity of the reference and the sensitivity of the comparison can be considered significantly different from each other. The standard error of the difference between the sensitivities was determined through bootstrapping with 1000 replications. AUC = area under the receiver operating characteristic curve; Time = average response time per item; Longstring = maximum longstring; IRV = intra-individual response variability; Synonyms = psychometric synonyms; RPR = resampled personal reliability; MD = Mahalanobis distance measure for which the calculation was based on subsamples; *r*_pbis_ = person-total correlation coefficient for which the calculation was based on subsamples

^a^ The ratings of the first and second measurement of the partially careless responding condition (*n* = 120) were used to determine the classification accuracy. ^b^ The ratings of the first and second measurement of the fully careless responding condition (*n* = 121) were used to determine the classification accuracy. ^c^ Sensitivity of the index set at specificity level of 95% (i.e., false-positive rate of 5%). ^d^ Sensitivity of the index set at specificity level of 99% (i.e., false-positive rate of 1%)

When detecting partially careless responding, all sets of indirect indices outperformed the average score of instructed response items in terms of AUC. However, none of the three sets outperformed the average score of instructed response items at the two sensitivity levels.

For detecting fully careless responding, the analyses revealed the following results: The first and second set outperformed the average score of instructed response items in terms of AUC and the two sensitivity levels; however, the third set “only” outperformed the average score of instructed response items in terms of AUC. For us, the most impressive finding of these analyses was the difference in sensitivity at a false-positive rate of 1% between the first set and average score of instructed response items. Whereas the first set detected every fully careless respondent, the average score of instructed response items “only” detected 59% of them.

## General discussion

The results of our studies should provide researchers with some useful insights on how to select and combine indirect indices for detecting careless responding in their data. Our key findings and recommendations on the further use of the 14 indices examined here are summarized in Table 10. In the following sections, the performance of each indirect index in our studies is discussed in detail.

**Table 10:** Summary of findings on the effectiveness of the indirect indices and recommendations for further use of the indirect indices

	Does the index perform better than chance?				Performance compared to direct CR measure				Contextual robustness		Code lines	Norm required?	Recommendation
Index	Study 1	Study 2	Study 4	Study 5	Study 1	Study 2	Study 4	Study 5	Affected by severity?	Affected by item keying?			
RPR	Yes	Yes	Yes	Yes	Better	B_PC_/E_FC_	E_PC_/W_FC_	E_PC_/B_FC_	No	No	228	No	Use
Synonyms	Yes	Yes	Yes	Yes	B_PC_/E_FC_	B_PC_/E_FC_	Equal	Equal	No	No	237	Recommended	Use
Antonyms	Yes	Yes	Yes	Yes	Equal	Equal	Equal	Equal	No	No	237	Recommended	Use
MD	Yes	Yes	Yes	Yes	B_PC_/E_FC_	B_PC_/E_FC_	E_PC_/W_FC_	Equal	No	No	58	Recommended	Use
*r*_pbis_	Yes	Yes	Yes	Yes	Equal	B_PC_/E_FC_	W_PC_/E_FC_	Equal	Likely better in detecting FC	No	87	Recommended	Use
Gnormed	Yes	Yes	Yes	Yes	B_PC_/E_FC_	B_PC_/W_FC_	Worse	Equal	Likely better in detecting PC	No	124	No	Use
H_t_	Yes	Yes	Yes	Yes	Equal	B_PC_/E_FC_	Worse	Equal	No	No	124	No	Use
Time	Yes	No_PC_/Yes_FC_	Yes	Yes	E_PC_/B_FC_	W_PC_/E_FC_	Worse	E_PC_/B_FC_	Yes, better in detecting FC	Yes, partially ineffective for UK scales	1	No	Use with caution
lz	Yes	Yes	Yes	No_PC_/Yes_FC_	B_PC_/E_FC_	B_PC_/W_FC_	Worse	Worse	Inconclusive	No	124	No	Use with caution
Outfit MSE	Yes	Yes	Yes	No_PC_/Yes_FC_	B_PC_/E_FC_	B_PC_/W_FC_	Worse	Worse	Inconclusive	No	177	No	Use with caution
Infit MSE	Yes	Yes	Yes	No_PC_/Yes_FC_	B_PC_/E_FC_	B_PC_/W_FC_	Worse	Worse	Inconclusive	No	177	No	Use with caution
INDCHI	Yes	Yes	No	No_PC_/Yes_FC_	Equal	Equal	Worse	Worse	Likely better in detecting FC	No	958	Recommended	Do not use
Longstring	No_PC_/Yes_FC_	No	Yes	Yes	W_PC_/E_FC_	Worse	Worse	Worse	Likely better in detecting FC	Yes, ineffective for UK scales	44	No	Do not use
IRV	No_PC_/Yes_FC_	No	No_PC_/Yes_FC_	No_PC_/Yes_FC_	Worse	Worse	Worse	Worse	Yes, better for detecting FC	Yes, ineffective for UK scales	1	No	Do not use

CR = careless responding; severity = severity of careless responding pattern, either partially or fully careless responding; item keying = keying of presented items, either bidirectionally keyed (i.e., positive and negatively worded items were used for the same construct) or unidirectionally keyed items only (i.e., all items of the same construct were keyed in the same direction); code lines = number of code lines needed to obtain final index; norm required = computation of the index requires a normative sample; PC = partially careless responding; FC = fully careless responding; B_PC_ = index performs better than the direct careless responding measure in detecting partially careless responding; B_FC_ = index performs better than the direct careless responding measure in detecting fully careless responding; E_PC_ = index is equally effective as the direct careless responding measure in detecting partially careless responding; E_FC_ = index is equally effective as the direct careless responding measure in detecting fully careless responding; W_PC_ = index performs worse than the direct careless responding measure in detecting partially careless responding; W_FC_ = index performs worse than the direct careless responding measure in detecting fully careless responding; UK = unidirectionally keyed scales (i.e., all items of the same construct were keyed in the same direction); Time = average response time per item; Longstring = maximum longstring; IRV = intra-individual response variability; Synonyms = psychometric synonyms; Antonyms = psychometric antonyms; RPR = resampled personal reliability; MD = Mahalanobis distance measure for which the calculation was based on subsamples; *r*_pbis_ = person-total correlation coefficient for which the calculation was based on subsamples; Gnormed = global (i.e., the average of the scale-specific scores was computed) normed Guttman error index for polytomous items; H_t_ = global (i.e., the average of the scale-specific scores was computed) person scalability index; lz = global (i.e., the average of the scale-specific scores was computed) standardized log-likelihood index for polytomous items; Infit MSE = global (i.e., the average of the scale-specific statistics was computed) Rasch model-based infit mean squared error; Outfit MSE = global (i.e., the average of the scale-specific statistics was computed) Rasch model-based outfit mean squared error; INDCHI = global (i.e., the average of the scale-specific scores was computed) individual contribution to model misfit for which the calculation of the two person-specific model log-likelihood values was based on subsamples

## Measures of response time

Given the high detection rates reported in previous studies (Goldammer et al., 2020; Huang et al., 2012; Niessen et al., 2016), the average response time per item showed a less satisfying performance across our studies than we expected. On the positive side, this index turned out to perform better than chance in detecting careless respondents in most examined conditions. However, it was affected by both contextual factors (see Table 10): The average response time per item performed worse when partial instead of full careless responding should be detected and worse when unidirectionally instead of bidirectionally keyed scales were used. In addition, its performance compared with the direct careless responding measures may also be considered only mediocre (see Table 10). A potential reason for the mediocre performance of the response time measure may be the fact that we used an aggregated measure that was based on raw time scores. This kind of response time measure was easily calculated and applied but may have also been less accurate than other calculation methods. For instance, item-level response time measures might have been better for modeling the process of speeding up that careless responders are expected to show. In addition, the biasing effects of extreme response times in the raw time score (e.g., very slow response times for a few items) may have been minimized by winsorizing and/or standardizing the raw time scores within each person. To sum up, we recommend caution when using an averaged response time measure that is based on raw scores for careless responding detection.

## Measures of response invariability

Contrary to previous studies (e.g., Huang et al., 2012; Niessen et al., 2016)—but in accordance with our expectations—the performance of maximum longstring and IRV was unsatisfactory across our studies: first, because these two indices were always among the least accurate of the 14 examined indices, and second, because their performance was clearly affected by both contextual factors (i.e., severity of careless responding, type of item keying). For instance, maximum longstring and IRV tended to perform worse or were even completely ineffective when partially instead of fully careless responding needed to be detected (see Table 10). Additionally, these indices were ineffective in the context of unidirectionally keyed scales and only performed marginally better than chance in in the context of bidirectionally keyed scales. Third, their detection rate turned out to be worse than that of the direct careless responding measures in almost all examined conditions.

The weak performance of the maximum longstring and the IRV was not surprising, given the results of Goldammer et al. (2020) regarding unidirectionally keyed scales. Moreover, an invariant careless response pattern is rather obvious and easy to spot. It might be employed by respondents who do not care about being identified as careless responders (Ward & Meade, 2022), and being identified as careless responder was something that participants in Study 1 and 2 were supposed to explicitly avoid. Considering this poor performance, we therefore do not recommend using the maximum longstring and the IRV for detecting careless responding.

## Measures of within-person inconsistency

In line with our expectation and results of previous studies (Goldammer et al., 2020; Huang et al., 2012; Meade & Craig, 2012), the three within-person consistency indices (i.e., RPR, psychometric synonyms, psychometric antonyms) performed remarkably well and did better than chance in all examined conditions. Their detection rates were not affected by the contextual factors that we examined, and they performed comparably to—and in some cases even better than—the direct careless responding measures (see Table 10). Thus, all three within-person consistency indices can be used for careless responding detection, but we recommend using at least one of them—optimally the RPR, which tended to be the best-performing within-person consistency index.

Nevertheless, it is important to keep in mind that even the within-person consistency indices only perform well if certain preconditions are given. For instance, a sufficient number of eligible pairs (strongly correlated item pairs in the case of psychometric synonyms and antonyms, and pairs of scale halves in the case of the RPR) needs to be found prior to calculation—at least three, but the more the better. In addition, it is important to note that we used the RPR for our analyses, which is based on the repeated calculation of the personal reliability across several randomly rearranged sets of scale halves, not the conventional personal reliability or even–odd consistency in which just one set of scale halves is used (e.g., halves of even- or odd-numbered items). As our supplementary analyses showed, only when we calculated the RPR could we be sure to have obtained a personal reliability index with the highest possible detection accuracy.

## Measures that reflect inconsistency with the sample norm

In line with our expectation and the results of previous studies (Goldammer et al., 2020; Karabatsos, 2003), the MD and *r*_pbis_ performed well: first, because they detected careless responding better than chance in all examined conditions; second, because their detection rates were mostly unaffected by the contextual factors that we examined; and lastly, because they performed comparably to—and in some cases even better than—the direct careless responding measures. Thus, both the MD and *r*_pbis_ can be used for careless responding detection, and we recommend using at least one of them.

Nevertheless, it is important to keep in mind that even these two indices only perform well if certain preconditions are given. For instance, the normative response pattern must be set by careful respondents; otherwise these indices might be less effective or even detect the wrong targets (i.e., careful respondents; see Curran, 2016). This was also the reason we calculated the MD and *r*_pbis_ on the basis of subsamples (see Goldammer et al., 2023), where the norm was always set in maximal favor for the careful responding group. The importance of the norm setting was also partially illustrated in the case of the MD (see supplementary material). For this index, the detection accuracy of careless respondents tended to be higher if the calculation was based on subsamples than when the index was calculated at once in the total sample.

## Measures that reflect model-based inconsistency

Within the group of indices that reflect a model-based inconsistency (i.e., H_t_, Gnormed, lz, infit MSE, outfit MSE, INDCHI), the two nonparametric person-fit indices Gnormed and H_t_ performed best. These two indices detected careless responding better than chance in all examined conditions, had detection rates that were mostly unaffected by the examined contextual factors, and performed comparably to the direct careless responding measures in many conditions (see Table 10). The good performance of these two indices in our studies is in line with the findings of Wind and Wang (2022). Thus, both Gnormed and H_t_ can be used for careless responding detection, and we recommend using at least one of them.

In the internal ranking of indices that reflect a model-based inconsistency, Gnormed and H_t_ are followed by the three parametric IRT-based person-fit indices lz, outfit MSE, and infit MSE. These three indices had somewhat lower detection rates than Gnormed and H_t_ and did not perform better than chance in any of the examined conditions. Furthermore, lz, outfit MSE, and infit MSE mostly performed worse than the direct careless responding measures and tended to perform inconsistently across the two levels of careless responding severity that we examined—partially versus fully careless responding. The mediocre performance of lz, outfit MSE, and infit MSE stands in contrast to Jones et al.’s findings (2023) of notably higher detection rates for these indices.^4^ This result underlines the need for further experimental and or simulation studies that examine in greater detail under what conditions these three parametric IRT-based person-fit indices are more or less effective in detecting careless responding. Until more is known, we recommend caution when using lz, outfit MSE, and infit MSE for careless responding detection.

In our internal ranking of indices that reflect a model-based inconsistency, the parametric covariance structure-based person-fit index INDCHI placed last. This index had somewhat lower detection rates than the three parametric IRT-based person-fit indices and did not perform better than chance in more than one third of the conditions. Furthermore, INDCHI performed worse than the direct careless responding measures in half of the conditions and tended to be less effective for partially careless responding. In view of this rather low performance, the calculation effort (i.e., 958 code lines in our case, see Table 10) seems hardly justifiable. We therefore do not recommend using INDCHI for detecting careless responding.

## The benefits of using indirect indices as sets

Human-generated careless responding may manifest in different forms, which is why different types of indices have been proposed for detection (Ward & Meade, 2022). Using them as a set in which each of the included indices is sensitive to one specific form of careless responding may therefore help to increase overall detection accuracy beyond the accuracy that can be achieved when using each index individually (see Goldammer et al., 2020). Our studies illustrate the benefit of using indirect indices as a set. For instance, compared to the RPR (i.e., the most accurate index in Study 1 according to the AUC), which detected 53% of the partially careless respondents at a false-positive rate of 5%, the set in which all effective indirect indices were used together detected 69%, and thus 16% more. The increase in sensitivity tended to be even more pronounced if a false-positive rate of only 1% was considered as acceptable. Whereas the RPR in Study 1 detected 14% of the partially careless respondents at a false-positive rate of 1%, the set in which all effective indirect indices were used together detected 46%, and thus 32% more. An even more impressive example in this regard was observed in Study 4. Compared to MD (i.e., the most accurate index in Study 4 according to the AUC), which detected 53% of the partially careless respondents at a false-positive rate of 5%, the set in which all effective indirect indices were used together had a perfect detection rate (i.e., 100%) and thus detected 47% more. As in Study 1, the increase in sensitivity tended to be even more pronounced if a false-positive rate of only 1% was considered as acceptable. Whereas the MD in Study 4 detected 36% of the partially careless respondents at a false-positive rate of 1%, the set in which all effective indirect indices were used together had again a perfect detection rate of 100% and thus detected 64% more. Based on these findings, we therefore generally recommend using several indirect indices for careless responding detection—optimally, indices with different screening principles. For instance, if wanted to use a set of three indices for our screening, RPR, MD, and Gnormed would be a reasonable choice based on our results.

## On the performance of direct careless responding measures

Our results also have several implications for the further use of direct careless responding measures. First, direct responding measures such as bogus items may only perform well if a sufficient number of items is used. For instance, compared to the average score of the three bogus items that we used in Studies 1 and 2, which had mostly only mediocre detection rates (i.e., with AUCs that ranged from 0.62 to 0.83), the average score of the eight bogus items that we used in Study 4 had remarkably high detection rates (i.e., with AUCs that ranged from 0.89 to 0.98). These findings illustrate that previous recommendations regarding the number bogus items that should be included in the survey (i.e., using no more than one bogus item per 50–100 regular items, up to the maximum number of three [Meade & Craig, 2012, p. 452], or using three bogus items for a 70-item questionnaire [Huang et al., 2015, p. 303]) are no longer tenable. Thus, if researchers wish to use bogus items for detecting careless responding, we recommend using at least eight items, such as the eight items of the infrequency scale (Huang et al., 2015). However, the additional inclusion of eight bogus items may not be desirable in every research context. For instance, whereas the inclusion of eight bogus items may not be a problem in the case of longer surveys (e.g., questionnaires with 120 items and more), it may be one in the case of shorter surveys (e.g., questionnaires that have fewer than 60 items), because it substantially lengthens the survey and may annoy respondents (due to imbalance between bogus and regular test items) and thus additionally increase the risk of careless responding.

Second, the average score of the eight bogus items that we used in Study 4 tended to perform better than the average score of the eight instructed response items that we used in Study 5. This finding stands in contrast to the recommendation of Meade and Craig (2012, p. 254), who advised using instructed response items rather than bogus items due to their clearer interpretation. However, it is in line with the finding of Curran and Hauser (2019) that there were better detection rates for bogus than for instructed response items. This underlines the need for future studies that examine in greater detail under what conditions instructed response items are more or less effective in detecting careless responding. Until more is known, we recommend being cautious when using instructed response items for careless responding detection.

Lastly, despite the strict dichotomy between direct and indirect careless responding indices that we outlined throughout the paper, the supplementary material (see Tables S7, S16, S29, S41) also illustrates options for effectively combining these two types of indices. For instance, using indirect indices in addition to direct measures tended to increase the overall detection accuracy when only a few bogus items (i.e., three) were used as direct measure, when instructed response items were used as direct measure, and when partially careless responding needed to be detected. Under these conditions, the three within-person consistency indices (i.e., RPR, psychometric synonyms, psychometric antonyms), the two sample norm-based indices (i.e., MD, *r*_pbis_), the two nonparametric person-fit indices (i.e., Gnormed, H_t_), and average response time per item tended to increase the detection accuracy most consistently across our studies.

## Limitations and future research directions

Our set of studies has several limitations that need to be mentioned. First, the generalizability of our findings may be limited because of the specific careless response set instruction that our participants received. With the careless responding instructions in our studies, we wanted to provoke a response behavior that most closely matched the real-world phenomena of careless responding. Nevertheless, it was a hypothetical scenario, and it can be plausibly argued that the provoked behavior only partially reflects naturally occurring careless responding. Future studies should therefore examine the detection accuracy of indirect indices under different—optimally non-hypothetical—response set instructions.

Second, the experimental approach with human-generated careless response patterns only allowed us to examine the indices’ effectiveness across a small set of contextual factors (i.e., severity of careless responding [partial vs. full]) and item keying [bidirectionally keyed scales vs. unidirectionally keyed scales]) with a finite number of samples. However, there are other contextual factors that may have an impact on the indices’ detection accuracy (e.g., type of item presentation, length of survey, number of response options, sample size, percentage of careless responders in the sample). In addition, our results indicated that the type of reference rating (i.e., either careful ratings of the same subjects at t1 [i.e., within-subject perspective] or careful ratings of different subjects at t2 [i.e., between-subject perspective]) might have an impact on the detection of some indices as well (i.e., H_t_, average response time per item, lz, outfit MSE, onfit MSE; see footnote 2). Therefore, future studies should extend our work and examine the performance of individual indices and sets of indices under further contextual conditions. Obviously, our experimental approach with human-generated careless response patterns becomes less attractive or even unfeasible with an increasing number of design factors and in contexts where the effect of sampling variability should be examined as well. In such instances, running a simulation study is the far better option, despite the potential limitation that simulated careless protocols may only partially reflect human-generated careless response protocols.

Lastly, it may be argued that we only examined a selection of indirect indices that is non-exhaustive. Indeed, there are other promising indirect indices or analytical approaches that we did not examine. For instance, change-point analyses and their derived indices have proven to be very useful in detecting the exact location in the questionnaire where a responder has shifted from careful to careless responding (e.g., Yu & Cheng, 2019, 2022). This kind of knowledge would give researchers more efficient options in handling the data of careless responders. For instance, instead of discarding the whole protocol, items beyond such a change-point could be simply treated as missing values, while the rest of the responses (those given at the beginning of the questionnaire, before the change-point) would be used for the main analyses (Edwards, 2019; Goldammer et al., 2020). Future studies should therefore examine the utility of alternative indirect indices in detecting careless responding, especially the issue of how indices derived from change-point analyses may supplement our tested set of indirect indices.

## Conclusion

A common strategy for detecting careless responding is the use of screening items, such as infrequency (bogus) items or instructed response items. However, these explicit or direct measures lengthen the questionnaire and may undermine the spirit of cooperation between researchers and respondents. As our results suggest, a better approach for assessing respondents’ careless responding may therefore be the use of indirect indices. First, indirect indices cannot be detected by the test-taker. Second, their use does not require changes to the regular questionnaire. Third, their use results in comparable and, in many cases, even better detection rates than the use of direct careless responding measures. We therefore encourage researchers to use indirect indices, such as within-person consistency indices, instead of direct measures when they aim to detect careless responding in their data.

## Supplementary Information

Below is the link to the electronic supplementary material.

Supplementary file1 (DOCX 461 KB)

## Data Availability

All data, analysis code, and research materials not already provided in the supplements are available under the following link: https://polybox.ethz.ch/index.php/s/88QNfcknKt9JHQm

## Footnotes

1 In Studies 4 and 5, we used an unincentivized and incentivized control group, because we feared that respondents who had to do the same task twice (i.e., complete the same questionnaire twice, honestly and accurately) would lose motivation at the second measurement and start to respond carelessly, which would have undermined any between- and within condition comparisons. However, the manipulation checks in Studies 4 and 5 revealed no differences between participants in the unincentivized and incentivized careful responding group (see “Manipulation check” sections in Studies 4 and 5 above).

2 We also examined whether the type of reference rating (either careful ratings of the same subjects at t1 [i.e., within-subject perspective] or careful ratings of different subjects at t2 [i.e., between-subject perspective]) had an effect on the detection accuracy of direct and indirect indices in Studies 4 and 5 (see Table S42). For the majority of the indices, the detection accuracies (i.e., AUC) changed only marginally across the two types of references ratings. However, five indices tended to perform differently. Whereas the H_t_ and the average response time per item tended to perform better when the careful ratings of the same subjects at t1 were used as a reference, the three parametric IRT-based person-fit indices lz, outfit MSE and infit MSE tended to perform better when the careful ratings of different subjects at t2 were used as a reference. Implications of this finding are elaborated in the Discussion section.

3 Because several instructed response items had no variance, the Cronbach’s alpha values could not be computed for all conditions.

4 Jones et al. (2023) used outfit and infit MSE statistics that were based on a bidirectional interpretation (i.e., their indices treated model underfit as well model overfit as misfit). To obtain their bidirectional outfit and infit MSE indices, they used the squared deviation from 1. We, on the other hand, used outfit and infit MSE statistics that were based on a unidirectional interpretation (i.e., our indices only treated model underfit as misfit). Thus, in the case of our outfit and infit MSE indices, only larger values were indicative of misfit. Naturally, this raised the question of whether the mediocre performance of the outfit and infit MSE in our studies only occurred because of the different calculation method. We therefore ran supplementary analyses and examined whether outfit and infit MSE indices that were based on a bidirectional interpretation (i.e., indices for which the squared deviation from 1 was used for calculation) would be better in detecting careless responding than outfit and infit MSE indices that were based on a unidirectional interpretation. However, in all of our studies, the bidirectional outfit and infit MSE statistics were always only as good as chance and thus worse than the unidirectional outfit and infit MSE statistics reported in our studies (which always performed better than chance). Thus, the different calculation method does not explain the mediocre performance of the outfit and infit MSE indices in our studies. This in turn underlines the need for future studies that examine under what conditions a unidirectional or a bidirectional MSE statistics interpretation is more advantageous.

## Declarations

### Transparency

The studies’ designs, hypotheses, and analyses were not preregistered.
