# Supplementary material for: Careless responding detection revisited: Accuracy of direct and indirect measures
Source: Behav Res Methods. 2024 Aug 15;56(8):8422–49. doi: 10.3758/s13428-024-02484-3 (PMC11525390; doi:10.3758/s13428-024-02484-3)
Supplement: Supplementary file 1 — Supplementary file1 (DOCX 461 KB) [file 13428_2024_2484_MOESM1_ESM.docx]

# Supplemental Material for

# Careless Responding Detection Revisited: Accuracy of Direct and Indirect Measures

# Script of Self-Presentation Video Used in Study 1 and 2

The video can be viewed via the following link:

<https://share.dma.swiss/s/6gp4BqHRCJ2LoJK>

The following password is needed to play the video:

NCO-Candidate-2024

## Instructions Regarding the Communication Behaviors of the Actor

The actor was instructed to hold steady, pleasant eye contact with the audience. Further, he was instructed to show vivid but authentic facial expressions and gestures that support the statements of his speech. He was instructed to speak fluently, with targeted voice modulations for decisive speech topics. In addition, he was also instructed to pause briefly at decisive moments of his speech.

## Content of the Short Presentation

Ladies and gentlemen, my name is Cédric Lauper, and I am delighted to have been given the opportunity to apply for the position of a professional Non-commissioned Officer (NCO)—I have arrived at the assessment center for prospective professional NCOs (ACABU) suitably motivated.

I have structured my short presentation as follows: First of all, I will talk about my civilian and military career and, in this context, also show why I am so keen to become a professional non-commissioned officer. In the second part of my presentation, I will then comment on the topic assigned to me, ‘comradeship.’ I would like to talk about both the positive and negative aspects of comradeship and illustrate them with examples from my practical service.

As far as my civilian career is concerned: I am 24 years old and come from Bern, where I live with my wife and our 2-year-old daughter. Besides carnival, *Fasnacht* as we call it, and football, tinkering with cars is one of my main hobbies and, having done an apprenticeship as a car mechanic, I have even been able to make this my profession. After completing my apprenticeship, I found a job at Garage Schmocker, a Mitsubishi dealership in Ostermundigen, where I continued to learn a lot, and I still work there. I really enjoy working in such a professional environment. For me, for example, it is very satisfying when you can work as a team to solve a problem that you don’t come across every day. Everyone in the team has their own strengths. For example, with my eye for detail and my creative suggestions for solutions, I have often been able to improve the basic approach that has been developed. If everyone pulls together in this way, it ultimately leads to a result that more than meets the customer’s wishes.

However, after four years at Garage Schmocker, I have reached a point where I want a new challenge. I have decided to pursue a career as a professional non-commissioned officer, provided I pass the ACABU. This would not be a decision that is anyway critical of Garage Schmocker but instead a positive decision for the Swiss Armed Forces. This is because in my short career in the Swiss Army so far, I have had many positive experiences.

But let’s go back to the beginning and talk about my military career so far: I went to recruit school in Thun; specifically, I went to Maintenance School 50 at the time, i.e., today’s Maintenance School 43. I trained as a maintenance specialist for wheeled vehicles, primarily as a tank mechanic. It goes without saying that we cursed every now and then when a tank driver failed to keep his Leopard under control and we were left to clear up the mess. Having said that, we were always satisfied after the work was done. It was impressive to see how a having good team means that these problems can be solved quickly.

Even though I didn’t like everything about the military, I was more than happy to train as a unit sergeant. I like working as a sergeant major. My lightbulb moment, however, came during my practical service as a sergeant major. One of the instructors dropped out due to illness, and because of my professional background I was allowed to replace him for a week. I really appreciated the way the recruits listened to me with such motivation. It was great to see how the recruits progressed and respected and appreciated me, not least because of my expertise. That was a formative experience, and I want to experience more of that in the future. That is the main reason why I want to become a professional NCO. I want to take on leadership responsibility and pass something on to young people.

I firmly believe that I have what it takes to become a successful professional NCO. I have the expertise, and the experience I have had as a leader in the armed forces so far also gives me confidence.

But now to the topic I was assigned to speak on: I am supposed to give my views on ‘comradeship.’ It is enormously important in the army. By comradeship, I mean that we are always there for each other and support each other as much as possible. If, for example, a comrade has a problem and is therefore less able to perform, it is up to the other comrades to compensate for this. They can help him by talking to him and trying to talk about his difficulties and maybe look for possible solutions. Or they can help him by taking over parts of his work. In my opinion, this is what is truly important for the military. If we don’t all pull together, it doesn’t work. So it doesn’t work without comradeship.

I would like to share two examples from my own experience. During my training as a sergeant, a comrade was in a very bad way for a while because his girlfriend wanted to break up with him. She had trouble with him wanting to pursue a military career. A colleague and I talked to him a lot. We chose not to do other things during our free time or simply to have a laugh when we went out, and instead spent most of the time listening to our comrade. That is what comradeship is all about, putting aside your own interests for the sake of others. We discussed a lot of things with him and then also took over a few of his tasks so that he could spend some time with his girlfriend during that period. We covered for him when he couldn’t show up for a briefing because he was on the phone to her. Fortunately, they were able to sort out their relationship problems and they are still together to this day. The same colleague once helped me a lot when I was not doing so well after a death in the family. He also took on tasks that were actually my responsibility, no questions asked. Being there for each other like that is also part of comradeship.

Nevertheless, comradeship can also have its negative sides. For example, we had a close comrade at recruit school who was struggling with alcohol problems. We knew what was going on; he was always very open with us about it. We repaid this trust by covering for him whenever he wasn’t really up to it or didn’t show up for a training exercise. But looking back, we might have been better comrades to him if we hadn’t covered for him. His drinking got worse, and one day he had to be rushed to hospital. With certain problems, comradeship reaches its limits, and you have to have the courage to report someone for their own good.

To sum up, I would say that comradeship can have its limits, especially when you support a comrade with the best of intentions but it ends up being dangerous for his health, or when it is used as a pretext to exclude others. But of course, comradeship is indispensable in the armed forces. The excellent comradeship that I experienced has left its mark on me. And not least for that reason, I hope to pass the ACABU and experience and contribute to our comradeship on a daily basis as a career NCO.

# Computation of Indirect Careless Responding Indices in Study 1

For computing the indirect indices in Study 1, we used only the 60 items from the Big-Five Inventory-2 (BFI-2; Soto & John, 2017). Negatively worded items were inverted prior to the computation of personal reliability, normed Guttman errors, personal scalability, standardized log-likelihood, infit and outfit mean square error (MSE) statistics, and individual contribution to the model misfit. For the computation of all other indices, the raw item scoring was used.

## Measures of Response Time

### Average Time Per Item

The Qualtrics survey tool that we used for the Study 1 allowed us to extract three response time measures for every survey page that was displayed (and all substantive items were displayed on a single web page). ‘First Click’ measures how many milliseconds the page was visible before the respondents clicked the first time (not including the submit button). ‘Last Click’ measures how many milliseconds the page was visible before the respondents clicked the last time (not including the submit button). ‘Page Submit’ measures how many milliseconds the page was visible before the respondent clicked on the submit button. For our analyses, we calculated an average score of the 60 Page Submit response time measures.

## Measures of Response Invariability

### Maximum Longstring

To obtain the maximum long string, we counted for each respondent the maximum number of identical responses in a row. After rearranging the data from wide to long format, we therefore used the community-contributed Stata function *tsspell* (Cox, 2007) and collapsed (using the max option) the variable _seq for each respondent. If a respondent’s response was missing, the maximum longstring was based on the remaining non-missing responses.

### Intra-Individual Response Variability

To obtain the intra-individual response variability (IRV), we computed the within-person standard deviation over the set of 60 substantive items. We therefore used the Stata function *rowsd*. If a respondent’s response was missing, the IRV was based on the remaining non-missing responses.

## Measure of Within-Person Inconsistency

### Psychometric Synonyms

To obtain the psychometric synonym index, we=first searched the item correlation matrix of the careful responding group for correlation coefficients larger than .60 (Meade & Craig, 2012, p. 442-443). However, because no coefficient reached this threshold, we started searching the item correlation matrix of the careful responding group for correlation coefficients larger than .50. Out of 1770 potential pairs, we identified 20 pairs meeting the criterion of being correlated larger than .50. Next, we computed the within-person correlation across these 20 pairs for each respondent. If an item pair had a missing value, the within-person correlation was based on the remaining item pairs with no missing values.

### Psychometric Antonyms

To obtain the psychometric antonym index, we first searched the item correlation matrix of the careful responding group for correlation coefficients lower than -.60 (Meade & Craig, 2012, p. 442-443). However, because no coefficient reached this threshold, we started searching the item correlation matrix of the careful responding group for correlation coefficients lower than -.50. Out of 1770 potential pairs, we identified 8 pairs meeting the criterion of being correlated lower than -.50. Next, we computed the within-person correlation across these 8 pairs for each respondent. If an item pair had a missing value, the within-person correlation was based on the remaining item pairs with no missing values. However, the antonym index could not be computed for one respondent in the partially careless responding group (i.e., Careless 33%) and two respondents in the fully careless responding group (i.e., Careless 100%), because one of their antonym vectors had no variance.

### Resampled Personal Reliability

We calculated the resampled personal reliability (RPR; Curran, 2016) as follows. Compared to the conventional personal reliability or even-odd consistency, in which just one set of scale halves is used (e.g., halves of even or odd numbered items), the RPR is based on the repeated calculation of the personal reliability across several randomly rearranged sets of scale halves. The RPR therefore helps to cancel out random error that may occur when using a specific (even weird) set of scale halve pairings for the reliability calculation (Curran, 2016).

To obtain the RPR we proceeded as follows: After transposing the data, we first used the Stata command *splitsample* with the *balance* option to randomly split the items of each of the 15 facets of the BFI-2 into two halves. In this transposed data, we then used the *collapse* command with the *by* option to obtain two scale half scores for each of the 15 facets. Next, we re-transposed the data and transformed the 15 scores of the first halves and the 15 scores of the second halves into a data set in long format. Based on this data set in long format, we then calculate the within-person correlation between the two scale halves and corrected each of these calculated correlation coefficients for decreased length with the Spearman-Brown formula (Brown, 1910; Spearman, 1910). In the rare cases where this corrected coefficient was less than -1, it was replaced by -1, which ensured that all corrected coefficients lay within the boundaries of -1 and 1. This whole process was repeated for 100 random splits (iterations), and the final RPR was obtained by taking the average of the resulting 100 personal reliabilities.

Across the 100 iterations, the standard deviation of the personal reliabilities within each respondent was on average 0.13 (*SD* = 0.11). This indicated that using different sets of scale half scores resulted in remarkably different personal reliabilities for each respondent. Because of this variability, we also examined how the personal reliability of each iteration performed compared to the RPR in detecting careless respondents. In 67 of the iterations, the personal reliability performed significantly worse than the RPR, and in only 33 of the iterations, the personal reliability performed as well as the RPR (area under the curve [AUC] = .87, *SE* = .02). Accordingly, in none of the iterations did the personal reliability perform better than the RPR. These results clearly illustrate that a more trustworthy and accurate index can be obtained when the calculation of personal reliability is based on several randomly rearranged sets of scale halves.

## Sample Norm-Based Inconsistency

### Mahalanobis Distance and Person-Total Correlation

As suggested by Goldammer et al. (2022), we used subsamples to calculate the Mahalanobis distance (MD; Mahalanobis, 1936) and the *r*_pbis_ (Donlon & Fischer, 1968; Curran, 2016, pp. 12-13). This meant that each respondent in the two careless responding groups was separately merged with the group of careful respondents for the calculation of the indices. The subsample runs resulted in 238 distance and *r*_pbis_ measures for every careful respondent and one distance and *r*_pbis_ measure for every careless respondent. To obtain the final distance and *r*_pbis_ measures for every careful respondent, the 238 values of subsample runs were averaged. The calculation in subsamples (Goldammer et al., 2022) was chosen because the validity of these indices grounds on the assumption that the majority of responses was given honestly (Curran, 2016, p. 13). Thus, calculating the indices at once for the total sample (*n* = 357) could have produced biased indices in our case, because careless respondents formed the majority (*n* = 238) in the sample of Study 1. Because the MD can only be computed if no missing item responses are present, we used the non-parametric imputation method (see Niessen et al., 2016, p. 6) that is available in the *PerFit* package (Tendeiro et al., 2016) in R (R Core Team, 2022) before calculating the distance measure. In the 60 substantive items, 18.5% (*n* = 66) of the respondents had at least one missing item response. However, we expected the effect of the imputation on the consistency of the response patterns to be minimal, because the vast majority of respondents with missing responses (89%) had only one or two item responses missing, with a maximum of 16 missing item responses. In the case of the *r*_pbis_, the remaining non-missing responses were used for calculation if respondents had missing item responses. The Stata command *mahascore* with the options *refmeans* and *compute_incov* was used for the computation of the MD. Based on a transposed data frame, the *r*_pbis_ could be computed with the *alpha* command in Stata and the item-rest correlation coefficients were extracted for further analyses purposes.

To check the utility of the subsample-based index calculations, we compared their performance in detecting careless respondents with the performance of distance/correlation indices that were calculated at once in the total sample. In the case of MD, the subsample-based version (AUC = .82; *SE* = .02) turned out to perform significantly better (χ^2^[1] = 5.19, *p* = .02) than the version in which the MD was calculated at once in the total sample (AUC = .80; *SE* = .02). In the case of the *r*_pbis_, however, the subsample-based version (AUC = .82; *SE* = .03) did not perform better (χ^2^[1] = 0.64, *p* = .42) than the version in which the *r*_pbis_ was calculated at once in the total sample (AUC = .82; *SE* = .03). Thus, the additional effort for calculating the subsample-based versions of MD and *r*_pbis_ paid off in terms of higher detection accuracy, at least in the case of the MD.

## Model-based inconsistency: Nonparametric Person-Fit Indices

### Guttman Error and Scalability Index

To obtain the normed Guttman error index for polytomous items (Gnormed; Emons, 2008; Molenaar, 1991) and the person or transposed scalability index (H_t_; Mokken; 1971; Sijtsma, 1986), we first computed for each of the five domains in BFI-2 domain-specific or scale-specific indices. On this basis, we then calculated a global or multi-test extension of the Gnormed and H_t_ (Drasgow et al., 1991; Niessen et al., 2016, p. 6) by averaging the values of the scale-specific indices. The *PerFit* package (Tendeiro et al., 2016) was used to calculate Gnormed, and the *mokken* package (Van der Ark, 2007) was used to calculate H_t_. For calculating Gnormed, we used a data set in which rows represented observations and columns variables/items. For calculating the H_t_, in contrast, we used a transposed data set in which rows represented variables/items and columns observations. Because the Gnormed and H_t_ cannot be computed if missing item responses are present, we used the parametric and non-parametric imputation method that is available in *PerFit* before calculating the Gnormed and H_t_ (see Niessen et al., 2016, p. 6). In the 60 substantive items, 18.5% (*n* = 66) of the respondents had at least one missing item response. However, we expected the effect of the imputation on consistency of the response patterns to be minimal, because the vast majority of respondents with missing responses (89%) had only one or two item responses missing, with a maximum of 16 missing item responses.

## Model-Based Inconsistency: Parametric IRT Based Person-Fit Indices

### Standardized Log-Likelihood and Infit and Outfit MSE

To obtain the standardized log-likelihood for polytomous items (lz; Drasgow et al., 1985), and the Raschmodel-based infit and outfit mean square error (MSE) statistics (Wright & Stone, 1979), we first computed for each of the five domains in BFI-2 domain-specific or scale-specific indices. On this basis, we then calculated a global or multi-test extension of lz and infit and outfit MSE (Drasgow et al., 1991; Niessen et al., 2016, p. 6) by averaging the values of the scale-specific indices. We estimated graded response models (Samejima, 1969) in the *PerFit* package (Tendeiro et al., 2016) in the case of lz, and Rasch rating scale models (Andrich, 1978) in the *TAM* package (Robitzsch et al., 2020) in the case of infit and outfit MSE statistics. Because lz cannot be computed if missing item responses are present, we used the parametric and non-parametric imputation method that is available in *PerFit* before calculating lz (see Niessen et al., 2016, p. 6). In the 60 substantive items, 18.5% of the respondents (*n* = 66) had at least one missing item response. However, we expected the effect of the imputation on consistency of the response patterns to be minimal, because the vast majority of respondents with missing responses (89%) had only one or two item responses missing, with a maximum of 16 missing item responses. When calculating infit and outfit MSE, in contrast, no imputation was necessary, because we used marginal maximum likelihood in the *TAM* package for estimating the Rasch rating scale models, which allowed us to handle missing values through full information estimation.

## Model-Based Inconsistency: Parametric Covariance-Structure Based Person-Fit Index

### Individual Contribution to Model Misfit

As suggested by Goldammer et al. (2022), we used subsamples to calculate the individual contribution to model misfit (INDCHI; Reise & Widaman, 1999, pp. 7-8). We did so because the second part of formula for calculating the person-specific log-likelihood (i.e., the basis for calculating the INDCHI) is equivalent to the squared Mahalanobis distance (Reise & Widaman, 1999, p. 7), which in turn grounds on the assumption that the majority of responses was given carefully, to be a valid measure for aberrant responding (Curran, 2016, p. 13). Thus, calculating the INDCHI at once for the total sample (*n* = 357), could have produced a biased index in our case, because careless respondents formed the majority (*n* = 238) in the sample of Study 1.

To obtain scale-specific INDCHI indices (for each of the five BFI-2 domains/scales) for each respondent, we first estimated a saturated model and a one-factor model for each scale and extracted the person-specific log-likelihood values of both models. In the subsample runs, we obtained for every careful respondent 238 person-specific log-likelihood values for the saturated model and 238 person-specific log-likelihood values for the one-factor model. For every careless respondent, one person-specific log-likelihood value for the saturated model and one person-specific log-likelihood value for the one-factor model were obtained from the subsample runs. The final two person-specific log-likelihood values for every careful respondent were obtained by averaging the 238 values of each of the two models. Next, the scale-specific INDCHI values could be computed for each respondent by subtracting the person-specific log-likelihood of the saturated model from the person-specific log-likelihood of the substantive factor model and by multiplying this difference by -2. Eventually, we computed a global or multi-test extension of the INDCHI by averaging the values of the scale-specific indices. All models were estimated with robust maximum likelihood estimation (MLR) in Mplus Version 8.8 (Muthén & Muthén, 1998-2017). Missing values were handled through the implemented default when using MLR (i.e., full information maximum likelihood estimation FIML).

To check the utility of the subsample-based index calculation, we compared its performance in detecting careless respondents with the performance of an INDCHI index that was calculated at once in the total sample. Whereas the subsample-based version detected careless respondents significantly better than chance (AUC = .69; *SE* = .03), the version in which the INDCHI was calculated at once in the total sample even turned out to screen in the wrong direction (AUC = .42; *SE* = .03) — it was more indicative for careful responding than for careless responding. Thus, the additional effort for calculating the subsample-based version of the INDCHI allowed us to obtain an index that screened in the correct (expected) direction with significant accuracy.

## Manipulation Check Study 1

To examine whether our manipulation worked we compared the ratings of the experimental groups with the ratings of a norm group. The participants in the norm group were 57 German-speaking prospective professional officers and NCOs who attended basic training courses for prospective officers and NCOs in the fall of 2021 at the military academy and the professional NCO school. They were on average 29.28 years old (*SD* = 4.43) and predominantly men (*n* = 52, 91.2%). Most of them had either completed upper secondary school (*n* = 23, 40.4%) or a certified apprenticeship (*n* = 23, 40.4%). Only a minority of the participants in the norm group (*n* = 11, 19.3%) indicated the nine years of compulsory schooling as their highest educational level. In our instructions, we told them that we had filmed new videos of assessment center exercises to facilitate the training of new assessors in our assessment centers. Thus, they would be helping us with their rating to examine whether the acted NCO candidate in the video is perceived by the viewer as we intended. If so, the videos could be used for assessor trainings and even experimental studies. They were then shown the NCO candidate’s self-presentation and afterwards asked to complete a questionnaire about that NCO candidate (i.e., the 60 BFI-2 items).

As expected, the scale scores of the careless respondents were biased towards the scale midpoint (Goldammer et al., 2020; Huang et al., 2015). Compared to the scale scores of the norm and careful responding group (whose scores did not differ from each other), the fully careless respondents (i.e., Careless 100%) had lower scores on the scales extraversion, agreeableness, and conscientiousness, and a higher score on the scale negative emotionality (see Table S1). In the case of the partially careless responding group (i.e., Careless 33%), the effects were in the expected direction as well; however, only the post-hoc tests for the scale negative emotionality reached the Bonferroni-corrected critical z-value of 3.28.

Next, we examined whether the means of three indirect careless response indices (average response time per item, IRV, RPR) differed between the norm and the three experimental groups. The mean comparisons revealed the expected pattern. Compared to the norm and the careful responding group, the fully careless respondents (i.e., Careless 100%) responded much faster and less consistently (see Table S1). In the case of the partially careless responding group (i.e., Careless 33%), the effects were in the expected direction as well; however, only the post-hoc tests for the RPR reached the Bonferroni-corrected critical z-value of 3.28.

Lastly, we then examined the differences in the correlation of the response vectors. The correlation between the normative response vector and the response vectors of the respondents in the three experimental conditions was as follows: Careful (*M* = .69, *SD* = .21), Careless 33% (*M* = .50, *SD* = .20), Careless 100% (*M* = .32, *SD* = .30). In line with our expectations, the response vectors of careful respondents correlated significantly stronger with the normative response vector than the response vectors of partially careless respondents (i.e., Careless 33%; Estimate_Δcorrelation_ = .18, *SE* = .03, *z* = 6.84, *p* < .001), and fully careless respondents (i.e., Careless 100%; Estimate_Δcorrelation_ = .36, *SE* = .03, *z* = 10.73, *p* < .001). In addition, the response vectors of partially careless respondents correlated significantly stronger with the normative response vector than the response vectors of fully careless respondents (Estimate_Δcorrelation_ = .18, *SE* = .03, *z* = 5.37, *p* < .001). Based on these results, we concluded the manipulation to be successful and therefore proceeded with the main analyses.

# Table S1

*Comparison of Substantive Scale Scores and Selected Indirect Careless Responding Indices between Norm and Experimental Conditions in Study 1*

|  | Response conditions | | | | | | |  |  |
| --- | --- | --- | --- | --- | --- | --- | --- | --- | --- |
|  | Norm |  | Careful |  | Careless 33% |  | Careless 100% |  |  |
| Measure | *M* (*SD*) |  | *M* (*SD*) |  | *M* (*SD*) |  | *M* (*SD*) | χ^2^(3) | Cohen’s *d* |
| Subtantive Scales |  |  |  |  |  |  |  |  |  |
| Extraversion | 4.21_a_ (0.51) |  | 4.35_a_ (0.51) |  | 4.26_a_ (0.45) |  | 3.86_b_ (0.54) | 60.59^*^ | 0.18_PC_/0.94_FC_ |
| Agreeableness | 4.68_a_ (0.44) |  | 4.64_ab_ (0.57) |  | 4.42_b_ (0.55) |  | 3.98_c_ (0.58) | 101.02^*^ | 0.40_PC_/1.15_FC_ |
| Conscientiousness | 4.59_ab_ (0.45) |  | 4.69_a_ (0.48) |  | 4.39_b_ (0.52) |  | 4.01_c_ (0.59) | 104.25^*^ | 0.60_PC_/1.27_FC_ |
| Negative Emotionality | 2.54_a_ (0.44) |  | 2.59_a_ (0.53) |  | 2.80_b_ (0.45) |  | 3.15_c_ (0.58) | 81.71^*^ | -0.42_PC_/-1.00_FC_ |
| Open-Mindedness | 3.63_ab_ (0.45) |  | 3.76_ab_ (0.60) |  | 3.82_ab_ (0.45) |  | 3.53_a_ (0.51) | 24.89^*^ | -0.12_PC_/0.41_FC_ |
| Careless Response Indices |  |  |  |  |  |  |  |  |  |
| Time | 7.33_ab_ (2.99) |  | 7.23_a_ (1.78) |  | 6.18_b_ (1.79) |  | 4.58_c_ (1.73) | 149.82^*^ | 0.59_PC_/1.51_FC_ |
| IRV | 1.24_a_ (0.29) |  | 1.33_ab_ (0.30) |  | 1.39_b_ (0.28) |  | 1.19_a_ (0.35) | 28.62^*^ | -0.23_PC_/0.41_FC_ |
| RPR | 0.81_a_ (0.21) |  | 0.83_a_ (0.18) |  | 0.56_b_ (0.28) |  | 0.40_c_ (0.42) | 159.80^*^ | 1.14_PC_/1.31_FC_ |

*Note.* Means and standard deviations are based on univariate sample statistics for the norm group (*n* = 57), the careful responding condition (*n* = 119), the partially careless responding (i.e., Careless 33%) condition (*n* = 120), and the fully careless responding (i.e., Careless 100%) condition (*n* = 118). In each row, means with different subscripts are significantly different from each other (i.e., α = .025/48, with the critical z-value of 3.28). χ^2^ = χ^2^ values were obtained by conducting Wald tests of parameter constraints that were based on robust full information maximum likelihood estimation (MLR) in Mplus Version 8.8. *d*_PC_ = Cohen’s *d* for the mean comparison between Careless 33% and Careful, *d*_FC_ = Cohen’s *d* for the mean comparison between Careless 100% and Careful.

^*^ Larger than the critical χ^2^(3) value of 12.36 (i.e., α = .05/8), which indicates global inequality between the means.

# Table S2

*Descriptive Statistics of the Substantive Measures and Careless Response Indices for the Careful Responding Group in Study 1*

| Scale | 1. | 2. | 3. | 4. | 5. | 6. | 7. | 8. | 9. | 10. | 11. | 12. | 13. | 14. | 15. | 16. | 17. | 18. | 19. | 20. |
| --- | --- | --- | --- | --- | --- | --- | --- | --- | --- | --- | --- | --- | --- | --- | --- | --- | --- | --- | --- | --- |
| 1. EX | 1 |  |  |  |  |  |  |  |  |  |  |  |  |  |  |  |  |  |  |  |
| 2. AG | .409** | 1 |  |  |  |  |  |  |  |  |  |  |  |  |  |  |  |  |  |  |
| 3. CO | .528** | .605** | 1 |  |  |  |  |  |  |  |  |  |  |  |  |  |  |  |  |  |
| 4. EM | -.629** | -.656** | -.701** | 1 |  |  |  |  |  |  |  |  |  |  |  |  |  |  |  |  |
| 5. OP | .514** | .496** | .407** | -.482** | 1 |  |  |  |  |  |  |  |  |  |  |  |  |  |  |  |
| 6. Bogus | -.109 | .036 | -.164 | .102 | .072 | 1 |  |  |  |  |  |  |  |  |  |  |  |  |  |  |
| 7. Time | -.054 | -.109 | -.055 | .037 | -.073 | -.216* | 1 |  |  |  |  |  |  |  |  |  |  |  |  |  |
| 8. Longstring | -.364** | -.370** | -.296** | .362** | -.166 | .218* | .093 | 1 |  |  |  |  |  |  |  |  |  |  |  |  |
| 9. IRV | .566** | .731** | .789** | -.632** | .382** | -.073 | -.041 | -.382** | 1 |  |  |  |  |  |  |  |  |  |  |  |
| 10. Synonyms | .418** | .432** | .578** | -.483** | .249** | -.161 | -.050 | -.368** | .499** | 1 |  |  |  |  |  |  |  |  |  |  |
| 11. Antonyms | -.407** | -.598** | -.403** | .437** | -.370** | .035 | .057 | .271** | -.421** | -.296** | 1 |  |  |  |  |  |  |  |  |  |
| 12. RPR | .418** | .373** | .505** | -.554** | .215* | -.536** | .053 | -.389** | .341** | .477** | -.428** | 1 |  |  |  |  |  |  |  |  |
| 13. MD | -.174 | -.018 | -.022 | .232* | -.259** | .029 | .080 | -.010 | .341** | -.156 | .195* | -.313** | 1 |  |  |  |  |  |  |  |
| 14. *r*_pbis_ | .638** | .672** | .632** | -.803** | .600** | -.113 | -.086 | -.327** | .404** | .472** | -.576** | .633** | -.479** | 1 |  |  |  |  |  |  |
| 15. Gnormed | .025 | .190* | .276** | .061 | -.088 | .048 | .103 | -.025 | .576** | .040 | .046 | -.164 | .792** | -.275** | 1 |  |  |  |  |  |
| 16. H_t_ | .403** | .402** | .360** | -.464** | .324** | -.065 | -.126 | -.263** | .263** | .405** | -.485** | .418** | -.417** | .636** | -.306** | 1 |  |  |  |  |
| 17. lz | -.018 | -.118 | -.205* | -.076 | .104 | -.037 | -.139 | .010 | -.529** | .053 | -.030 | .186* | -.838** | .292** | -.893** | .325** | 1 |  |  |  |
| 18. Infit MSE | .175 | .327** | .411** | -.092 | .049 | .034 | .084 | -.080 | .703** | .139 | -.074 | -.073 | .755** | -.109 | .964** | -.185* | -.898** | 1 |  |  |
| 19. Outfit MSE | .163 | .312** | .394** | -.075 | .026 | .037 | .078 | -.080 | .693** | .129 | -.061 | -.084 | .776** | -.131 | .962** | -.204* | -.912** | .998** | 1 |  |
| 20. INDCHI | -.273** | -.171 | -.131 | .309** | -.239** | -.116 | .084 | .035 | .064 | -.113 | .170 | -.061 | .379** | -.404** | .421** | -.291** | -.480** | .344** | .356** | 1 |
| *M* | 4.350 | 4.645 | 4.689 | 2.592 | 3.756 | 1.373 | 7.226 | 3.109 | 1.328 | 0.584 | -0.802 | 0.826 | 59.338 | 0.734 | 0.085 | 0.147 | 0.872 | 0.649 | 0.640 | 0.883 |
| *SD* | 0.512 | 0.572 | 0.482 | 0.531 | 0.602 | 0.539 | 1.777 | 0.955 | 0.302 | 0.249 | 0.238 | 0.176 | 14.200 | 0.211 | 0.051 | 0.058 | 0.678 | 0.334 | 0.318 | 0.997 |

*Note.* The careful responding group included 119 participants. All reported Pearson correlation coefficients and means and standard deviations are based on 119 observations except for the estimates that involve the score on bogus items, which are based on 118 observations (because one participant did not answer all bogus items). EX = Extraversion; AG = Agreeableness; CO = Conscientiousness; EM = Negative Emotionality; OP = Open-Mindedness; bogus = average score of the three bogus items; time = average response time per item; longstring = maximum longstring; IRV = intra-individual response variability; synonyms = psychometric synonyms; antonyms = psychometric antonyms; RPR = resampled personal reliability; MD = Mahalanobis distance measure for which the calculation was based on subsamples; *r*_pbis_ = person-total correlation coefficient for which the calculation was based on subsamples; Gnormed = global (i.e., the average of the scale-specific scores was computed) normed Guttman error index for polytomous items; H_t_ = global (i.e., the average of the scale-specific scores was computed) person scalability index; lz = global (i.e., the average of the scale-specific scores was computed) standardized log-likelihood index for polytomous items; infit MSE = global (i.e., the average of the scale-specific statistics was computed) Rasch-model based infit mean square error; outfit MSE = global (i.e., the average of the scale-specific statistics was computed) Rasch-model based outfit mean square error; INDCHI = global (i.e., the average of the scale-specific scores was computed) individual contribution to model misfit for which the calculation of the two person-specific model log-likelihood values was based on subsamples.

* *p* < .05 (two-tailed).

** *p* < .01 (two-tailed).

# Table S3

*Descriptive Statistics of the Substantive Measures and Careless Response Indices for the Partially Careless Responding Group (i.e., Careless 33%) in Study 1*

| Scale | 1. | 2. | 3. | 4. | 5. | 6. | 7. | 8. | 9. | 10. | 11. | 12. | 13. | 14. | 15. | 16. | 17. | 18. | 19. | 20. |
| --- | --- | --- | --- | --- | --- | --- | --- | --- | --- | --- | --- | --- | --- | --- | --- | --- | --- | --- | --- | --- |
| 1. EX | 1 |  |  |  |  |  |  |  |  |  |  |  |  |  |  |  |  |  |  |  |
| 2. AG | .594** | 1 |  |  |  |  |  |  |  |  |  |  |  |  |  |  |  |  |  |  |
| 3. CO | .628** | .631** | 1 |  |  |  |  |  |  |  |  |  |  |  |  |  |  |  |  |  |
| 4. EM | -.450** | -.436** | -.447** | 1 |  |  |  |  |  |  |  |  |  |  |  |  |  |  |  |  |
| 5. OP | .278** | .328** | .266** | -.233* | 1 |  |  |  |  |  |  |  |  |  |  |  |  |  |  |  |
| 6. Bogus | -.232* | -.333** | -.363** | .180* | -.079 | 1 |  |  |  |  |  |  |  |  |  |  |  |  |  |  |
| 7. Time | .049 | .078 | .096 | -.001 | .037 | -.086 | 1 |  |  |  |  |  |  |  |  |  |  |  |  |  |
| 8. Longstring | -.246** | -.287** | -.298** | .067 | -.118 | .016 | -.004 | 1 |  |  |  |  |  |  |  |  |  |  |  |  |
| 9. IRV | .480** | .509** | .484** | -.349** | .134 | .048 | -.023 | -.252** | 1 |  |  |  |  |  |  |  |  |  |  |  |
| 10. Synonyms | .370** | .424** | .429** | -.228* | .038 | -.389** | .007 | -.194* | .108 | 1 |  |  |  |  |  |  |  |  |  |  |
| 11. Antonyms | -.355** | -.547** | -.393** | .189* | -.182* | .180 | -.159 | .191* | -.230* | -.498** | 1 |  |  |  |  |  |  |  |  |  |
| 12. RPR | .460** | .470** | .505** | -.539** | .066 | -.445** | .111 | -.188* | -.041 | .484** | -.347** | 1 |  |  |  |  |  |  |  |  |
| 13. MD | -.058 | -.126 | -.199* | .103 | -.084 | .416** | -.148 | -.002 | .526** | -.456** | .355** | -.552** | 1 |  |  |  |  |  |  |  |
| 14. *r*_pbis_ | .679** | .775** | .804** | -.565** | .356** | -.418** | .098 | -.218* | .213* | .508** | -.527** | .659** | -.444** | 1 |  |  |  |  |  |  |
| 15. Gnormed | .042 | .011 | -.016 | .041 | -.130 | .300** | -.116 | -.056 | .747** | -.289** | .160 | -.525** | .811** | -.325** | 1 |  |  |  |  |  |
| 16. H_t_ | .187* | .389** | .324** | -.109 | .088 | -.286** | .084 | -.024 | -.004 | .436** | -.530** | .405** | -.513** | .536** | -.392** | 1 |  |  |  |  |
| 17. lz | .006 | .040 | .039 | -.094 | .139 | -.333** | .105 | .058 | -.703** | .339** | -.183* | .577** | -.827** | .354** | -.959** | .408** | 1 |  |  |  |
| 18. Infit MSE | .161 | .130 | .108 | -.060 | -.065 | .245** | -.118 | -.082 | .811** | -.233* | .103 | -.426** | .777** | -.194* | .984** | -.333** | -.932** | 1 |  |  |
| 19. Outfit MSE | .133 | .095 | .087 | -.035 | -.056 | .280** | -.124 | -.096 | .798** | -.276** | .127 | -.465** | .809** | -.223* | .981** | -.370** | -.957** | .989** | 1 |  |
| 20. INDCHI | -.231* | -.216* | -.203* | .203* | -.190* | .297** | -.123 | -.032 | .449** | -.251** | .233* | -.593** | .586** | -.446** | .772** | -.339** | -.823** | .707** | .747** | 1 |
| *M* | 4.263 | 4.420 | 4.387 | 2.802 | 3.819 | 1.853 | 6.181 | 3.300 | 1.394 | 0.268 | -0.559 | 0.563 | 80.338 | 0.565 | 0.170 | 0.094 | -0.324 | 1.175 | 1.170 | 2.901 |
| *SD* | 0.454 | 0.553 | 0.517 | 0.453 | 0.448 | 0.910 | 1.794 | 1.105 | 0.275 | 0.287 | 0.384 | 0.275 | 14.552 | 0.223 | 0.092 | 0.051 | 1.213 | 0.597 | 0.570 | 3.593 |

*Note.* The partially careless responding group included 120 participants. All reported Pearson correlation coefficients and means and standard deviations are based on 120 observations except for the estimates that involve the antonym index, which are based on 119 observations (because the antonym vectors of one participant had no variance). EX = Extraversion; AG = Agreeableness; CO = Conscientiousness; EM = Negative Emotionality; OP = Open-Mindedness; bogus = average score of the three bogus items; time = average response time per item; longstring = maximum longstring; IRV = intra-individual response variability; synonyms = psychometric synonyms; antonyms = psychometric antonyms; RPR = resampled personal reliability; MD = Mahalanobis distance measure for which the calculation was based on subsamples; *r*_pbis_ = person-total correlation coefficient for which the calculation was based on subsamples; Gnormed = global (i.e., the average of the scale-specific scores was computed) normed Guttman error index for polytomous items; H_t_ = global (i.e., the average of the scale-specific scores was computed) person scalability index; lz = global (i.e., the average of the scale-specific scores was computed) standardized log-likelihood index for polytomous items; infit MSE = global (i.e., the average of the scale-specific statistics was computed) Rasch model-based infit mean square error; outfit MSE = global (i.e., the average of the scale-specific statistics was computed) Rasch model-based outfit mean square error; INDCHI = global (i.e., the average of the scale-specific scores was computed) individual contribution to model misfit for which the calculation of the two person-specific model log-likelihood values was based on subsamples.

* *p* < .05 (two-tailed).

** *p* < .01 (two-tailed).

# Table S4

*Descriptive Statistics of the Substantive Measures and Careless Response Indices for the Fully Careless Responding Group (i.e., Careless 100%) in Study 1*

| Scale | 1. | 2. | 3. | 4. | 5. | 6. | 7. | 8. | 9. | 1. | 11. | 12. | 13. | 14. | 15. | 16. | 17. | 18. | 19. | 2. |
| --- | --- | --- | --- | --- | --- | --- | --- | --- | --- | --- | --- | --- | --- | --- | --- | --- | --- | --- | --- | --- |
| 1. EX | 1 |  |  |  |  |  |  |  |  |  |  |  |  |  |  |  |  |  |  |  |
| 2. AG | .594** | 1 |  |  |  |  |  |  |  |  |  |  |  |  |  |  |  |  |  |  |
| 3. CO | .628** | .631** | 1 |  |  |  |  |  |  |  |  |  |  |  |  |  |  |  |  |  |
| 4. EM | -.450** | -.436** | -.447** | 1 |  |  |  |  |  |  |  |  |  |  |  |  |  |  |  |  |
| 5. OP | .278** | .328** | .266** | -.233* | 1 |  |  |  |  |  |  |  |  |  |  |  |  |  |  |  |
| 6. Bogus | -.232* | -.333** | -.363** | .180* | -.079 | 1 |  |  |  |  |  |  |  |  |  |  |  |  |  |  |
| 7. Time | .049 | .078 | .096 | -.001 | .037 | -.086 | 1 |  |  |  |  |  |  |  |  |  |  |  |  |  |
| 8. Longstring | -.246** | -.287** | -.298** | .067 | -.118 | .016 | -.004 | 1 |  |  |  |  |  |  |  |  |  |  |  |  |
| 9. IRV | .480** | .509** | .484** | -.349** | .134 | .048 | -.023 | -.252** | 1 |  |  |  |  |  |  |  |  |  |  |  |
| 10. Synonyms | .370** | .424** | .429** | -.228* | .038 | -.389** | .007 | -.194* | .108 | 1 |  |  |  |  |  |  |  |  |  |  |
| 11. Antonyms | -.355** | -.547** | -.393** | .189* | -.182* | .180 | -.159 | .191* | -.230* | -.498** | 1 |  |  |  |  |  |  |  |  |  |
| 12. RPR | .460** | .470** | .505** | -.539** | .066 | -.445** | .111 | -.188* | -.041 | .484** | -.347** | 1 |  |  |  |  |  |  |  |  |
| 13. MD | -.058 | -.126 | -.199* | .103 | -.084 | .416** | -.148 | -.002 | .526** | -.456** | .355** | -.552** | 1 |  |  |  |  |  |  |  |
| 14. *r*_pbis_ | .679** | .775** | .804** | -.565** | .356** | -.418** | .098 | -.218* | .213* | .508** | -.527** | .659** | -.444** | 1 |  |  |  |  |  |  |
| 15. Gnormed | .042 | .011 | -.016 | .041 | -.130 | .300** | -.116 | -.056 | .747** | -.289** | .160 | -.525** | .811** | -.325** | 1 |  |  |  |  |  |
| 16. H_t_ | .187* | .389** | .324** | -.109 | .088 | -.286** | .084 | -.024 | -.004 | .436** | -.530** | .405** | -.513** | .536** | -.392** | 1 |  |  |  |  |
| 17. lz | .006 | .040 | .039 | -.094 | .139 | -.333** | .105 | .058 | -.703** | .339** | -.183* | .577** | -.827** | .354** | -.959** | .408** | 1 |  |  |  |
| 18. Infit MSE | .161 | .130 | .108 | -.060 | -.065 | .245** | -.118 | -.082 | .811** | -.233* | .103 | -.426** | .777** | -.194* | .984** | -.333** | -.932** | 1 |  |  |
| 19. Outfit MSE | .133 | .095 | .087 | -.035 | -.056 | .280** | -.124 | -.096 | .798** | -.276** | .127 | -.465** | .809** | -.223* | .981** | -.370** | -.957** | .989** | 1 |  |
| 20. INDCHI | -.231* | -.216* | -.203* | .203* | -.190* | .297** | -.123 | -.032 | .449** | -.251** | .233* | -.593** | .586** | -.446** | .772** | -.339** | -.823** | .707** | .747** | 1 |
| *M* | 4.263 | 4.420 | 4.387 | 2.802 | 3.819 | 1.853 | 6.181 | 3.300 | 1.394 | 0.268 | -0.559 | 0.563 | 80.338 | 0.565 | 0.170 | 0.094 | -0.324 | 1.175 | 1.170 | 2.901 |
| *SD* | 0.454 | 0.553 | 0.517 | 0.453 | 0.448 | 0.910 | 1.794 | 1.105 | 0.275 | 0.287 | 0.384 | 0.275 | 14.552 | 0.223 | 0.092 | 0.051 | 1.213 | 0.597 | 0.570 | 3.593 |

*Note.* The fully careless responding group included 118 participants. All reported Pearson correlation coefficients and means and standard deviations are based on 118 observations except for the estimates that involve the antonym index, which are based on 116 observations (because the antonym vectors of two participants had no variance). EX = Extraversion; AG = Agreeableness; CO = Conscientiousness; EM = Negative Emotionality; OP = Open-Mindedness; bogus = average score of the three bogus items; time = average response time per item; longstring = maximum longstring; IRV = intra-individual response variability; synonyms = psychometric synonyms; antonyms = psychometric antonyms; RPR = resampled personal reliability; MD = Mahalanobis distance measure for which the calculation was based on subsamples; *r*_pbis_ = person-total correlation coefficient for which the calculation was based on subsamples; Gnormed = global (i.e., the average of the scale-specific scores was computed) normed Guttman error index for polytomous items; H_t_ = global (i.e., the average of the scale-specific scores was computed) person scalability index; lz = global (i.e., the average of the scale-specific scores was computed) standardized log-likelihood index for polytomous items; infit MSE = global (i.e., the average of the scale-specific statistics was computed) Rasch model-based infit mean square error; outfit MSE = global (i.e., the average of the scale-specific statistics was computed) Rasch model-based outfit mean square error; INDCHI = global (i.e., the average of the scale-specific scores was computed) individual contribution to model misfit for which the calculation of the two person-specific model log-likelihood values was based on subsamples.

* *p* < .05 (two-tailed).

** *p* < .01 (two-tailed).

# Table S5

*Ordered Accuracies of the 14 Indirect Indices in Detecting Partially Careless (i.e., Careless 33%)* *Responding and Corresponding Pairwise Comparison Results in Study 1*

| Index | AUC_[95% CI]_ | Sen95 ^a^ | Sen99 ^b^ | 1. | 2. | 3. | 4. | 5. | 6. | 7. | 8. | 9. | 10. | 11. | 12. | 13. |
| --- | --- | --- | --- | --- | --- | --- | --- | --- | --- | --- | --- | --- | --- | --- | --- | --- |
| 1. RPR | .86_[.81, .91]_ | .53 | .14 |  |  |  |  |  |  |  |  |  |  |  |  |  |
| 2. MD | .84_[.80, .89]_ | .48 | .33 | 0.25 |  |  |  |  |  |  |  |  |  |  |  |  |
| 3. lz | .81_[.76, .86]_ | .46 | .23 | 2.60 | 5.75 |  |  |  |  |  |  |  |  |  |  |  |
| 4. Synonyms | .81_[.75, .86]_ | .23 | .06 | 3.93 | 1.39 | 0.00 |  |  |  |  |  |  |  |  |  |  |
| 5. Gnormed | .81_[.75, .86]_ | .39 | .24 | 2.82 | 7.27 | 0.18 | 0.00 |  |  |  |  |  |  |  |  |  |
| 7. Outfit MSE | .80_[.75, .86]_ | .43 | .22 | 3.02 | 7.33 | 0.91 | 0.05 | 0.74 |  |  |  |  |  |  |  |  |
| 8. Infit MSE | .79_[.74, .85]_ | .41 | .21 | 3.66 | 9.03 | 2.39 | 0.17 | 3.14 | 8.88 |  |  |  |  |  |  |  |
| 8. *r*_pbis_ | .78_[.72, .84]_ | .07 | .04 | 12.28 | 5.87 | 0.91 | 0.90 | 0.64 | 0.31 | 0.12 |  |  |  |  |  |  |
| 9. H_t_ | .77_[.71, .83]_ | .11 | .02 | 9.58 | 7.02 | 1.68 | 1.64 | 1.38 | 0.83 | 0.48 | 0.23 |  |  |  |  |  |
| 10. INDCHI | .72_[.65, .78]_ | .37 | .27 | 14.96 | 16.70 | 9.82 | 5.77 | 7.72 | 6.12 | 4.95 | 3.30 | 2.00 |  |  |  |  |
| 11. Antonyms | .71_[.65, .77]_ | .25 | .07 | 26.78 | 14.75 | 6.87 | 9.94 | 5.94 | 4.63 | 3.77 | 5.55 | 3.35 | 0.04 |  |  |  |
| 12. Time | .68_[.61, .75]_ | .18 | .05 | 18.56 | 16.78 | 8.99 | 8.41 | 8.40 | 7.70 | 6.68 | 4.88 | 3.88 | 0.62 | 0.42 |  |  |
| 13. Longstring | .54_[.47, .61]_ | .09 | .02 | 65.71 | 47.21 | 35.35 | 44.53 | 32.70 | 30.37 | 28.29 | 32.08 | 26.60 | 13.97 | 15.03 | 6.24 |  |
| 14. IRV | .43_[.36, .50]_ | .03 | .01 | 130.02 | 70.11 | 48.01 | 96.96 | 44.60 | 40.59 | 37.98 | 76.85 | 58.45 | 30.19 | 52.23 | 24.10 | 6.90 |

*Note.* A subsample from Study 1 including only participants in the careful responding condition and the partially responding condition was used to determine the classification accuracy (*n* = 239). Because the antonym index could not be computed for one respondent in the partially careless responding group (i.e., Careless 33%), all reported estimates and test statistics are based on 100 multiply imputed data sets. All indices were coded such that higher scores were more indicative of careless responding. Values in the matrix part represent the *F*-values for the pairwise comparisons with one degree of freedom for the numerator and degrees of freedom that ranged from 4.5*10^6^ to ∞ for the denominator. With the most conservative denominator value of 4.5*10^6^, the Bonferroni-corrected critical *F*-value for each of the 91 comparisons was 11.94; If this value is exceeded, the two AUCs can be considered significantly different from each other, and the corresponding cells in the matrix part are shown in gray. AUC = area under the receiver operating characteristic curve; Time = average response time per item; Longstring = maximum longstring; IRV = intra-individual response variability; Synonyms = psychometric synonyms; Antonyms = psychometric antonyms; RPR = resampled personal reliability; MD = Mahalanobis distance measure for which the calculation was based on subsamples; *r*_pbis_ = person-total correlation coefficient for which the calculation was based on subsamples; Gnormed = global (i.e., the average of the scale-specific scores was computed) normed Guttman error index for polytomous items; H_t_ = global (i.e., the average of the scale-specific scores was computed) person scalability index; lz = global (i.e., the average of the scale-specific scores was computed) standardized log-likelihood index for polytomous items; Infit MSE = global (i.e., the average of the scale-specific statistics was computed) Rasch model-based infit mean square error; Outfit MSE = global (i.e., the average of the scale-specific statistics was computed) Rasch model-based outfit mean square error; INDCHI = global (i.e., the average of the scale-specific scores was computed) individual contribution to model misfit for which the calculation of the two person-specific model log-likelihood values was based on subsamples.

^a^ Sensitivity of the index at specificity level of 95% (i.e., false-positive rate of 5%). ^b^ Sensitivity of the index at specificity level of 99% (i.e., false-positive rate of 1%).

# Table S6

*Ordered Accuracies of the 14 Indirect Indices in Detecting Fully Careless (i.e., Careless 100%)* *Responding and Corresponding Pairwise Comparison Results in Study 1*

| Index | AUC_[95% CI]_ | Sen95 ^a^ | Sen99 ^b^ | 1. | 2. | 3. | 4. | 5. | 6. | 7. | 8. | 9. | 10. | 11. | 12. | 13. |
| --- | --- | --- | --- | --- | --- | --- | --- | --- | --- | --- | --- | --- | --- | --- | --- | --- |
| 1. RPR | .88_[.84, .93]_ | .61 | .31 |  |  |  |  |  |  |  |  |  |  |  |  |  |
| 2. Time | .87_[.83, .92]_ | .55 | .37 | 0.16 |  |  |  |  |  |  |  |  |  |  |  |  |
| 3. *r*_pbis_ | .85_[.80, .90]_ | .33 | .25 | 3.73 | 0.31 |  |  |  |  |  |  |  |  |  |  |  |
| 4. Synonyms | .83_[.78, .89]_ | .21 | .04 | 4.01 | 1.18 | 0.48 |  |  |  |  |  |  |  |  |  |  |
| 5. Antonyms | .81_[.76, .87]_ | .40 | .16 | 8.97 | 2.69 | 3.33 | 0.41 |  |  |  |  |  |  |  |  |  |
| 6. H_t_ | .79_[.73, .85]_ | .36 | .08 | 16.38 | 4.90 | 8.64 | 1.95 | 0.94 |  |  |  |  |  |  |  |  |
| 7. MD | .79_[.73, .84]_ | .41 | .31 | 12.68 | 5.40 | 6.22 | 1.75 | 0.74 | 0.00 |  |  |  |  |  |  |  |
| 8. Gnormed | .71_[.64, .77]_ | .29 | .19 | 26.62 | 17.24 | 18.02 | 9.52 | 8.05 | 5.49 | 24.88 |  |  |  |  |  |  |
| 9. lz | .71_[.64, .77]_ | .36 | .24 | 26.24 | 17.49 | 17.13 | 9.99 | 7.83 | 5.06 | 20.78 | 0.00 |  |  |  |  |  |
| 10. INDCHI | .67_[.60, .74]_ | .31 | .26 | 32.76 | 23.65 | 27.85 | 15.24 | 14.29 | 9.08 | 12.25 | 1.13 | 1.20 |  |  |  |  |
| 11. Outfit MSE | .66_[.59, .73]_ | .27 | .15 | 34.81 | 27.42 | 24.54 | 16.04 | 14.10 | 11.01 | 39.17 | 29.47 | 21.08 | 0.17 |  |  |  |
| 12. Infit MSE | .65_[.59, .72]_ | .26 | .14 | 35.15 | 27.98 | 24.90 | 16.39 | 14.41 | 11.32 | 39.75 | 30.73 | 21.31 | 0.24 | 1.68 |  |  |
| 13. Longstring | .64_[.57, .71]_ | .21 | .04 | 43.48 | 30.41 | 31.45 | 22.28 | 18.67 | 14.60 | 10.64 | 1.78 | 1.75 | 0.39 | 0.10 | 0.07 |  |
| 14. IRV | .62_[.54, .69]_ | .18 | .10 | 52.71 | 36.51 | 39.83 | 31.62 | 26.81 | 16.06 | 10.30 | 2.21 | 2.18 | 0.99 | 0.41 | 0.35 | 0.42 |

*Note.* A subsample from Study 1 including only participants in the careful responding condition, and the fully responding condition was used to determine the classification accuracy (*n* = 237). Because the antonym index could not be computed for two respondents in the fully careless responding group (i.e., Careless 100%), all reported estimates and test statistics are based on 100 multiply imputed data sets. All indices were coded such that higher scores were more indicative of careless responding. Values in the matrix part represent the *F*-values for the pairwise comparisons with one degree of freedom for the numerator and degrees of freedom that ranged from 1.6*10^7^ to ∞ for the denominator. With the most conservative denominator value of 1.6*10^7^, the Bonferroni-corrected critical *F*-value for each of the 91 comparisons was 11.94; if this value is exceeded, the two AUCs can be considered significantly different from each other, and the corresponding cells in the matrix part are shown in gray. AUC = area under the receiver operating characteristic curve; Time = average response time per item; Longstring = maximum longstring; IRV = intra-individual response variability; Synonyms = psychometric synonyms; Antonyms = psychometric antonyms; RPR = resampled personal reliability; MD = Mahalanobis distance measure for which the calculation was based on subsamples; *r*_pbis_ = person-total correlation coefficient for which the calculation was based on subsamples; Gnormed = global (i.e., the average of the scale-specific scores was computed) normed Guttman error index for polytomous items; H_t_ = global (i.e., the average of the scale-specific scores was computed) person scalability index; lz = global (i.e., the average of the scale-specific scores was computed) standardized log-likelihood index for polytomous items; Infit MSE = global (i.e., the average of the scale-specific statistics was computed) Rasch model-based infit mean square error; Outfit MSE = global (i.e., the average of the scale-specific statistics was computed) Rasch model based-outfit mean square error; INDCHI = global (i.e., the average of the scale-specific scores was computed) individual contribution to model misfit for which the calculation of the two person-specific model log-likelihood values was based on subsamples.

^a^ Sensitivity of the index at specificity level of 95% (i.e., false-positive rate of 5%). ^b^ Sensitivity of the index at specificity level of 99% (i.e., false-positive rate of 1%).

# Table S7

*Incremental Validity Beyond Average Score on Bogus Items of Each Indirect Index in Study 1*

|  | Careless 33% ^a^ | |  | Careless 100% ^b^ | |
| --- | --- | --- | --- | --- | --- |
| Index | AUC_[95% CI]_ | χ^2^(1) |  | AUC_[95% CI]_ | χ^2^(1) |
| Bogus (reference) | .66_[.59, .73]_ |  |  | .76_[.70, .82]_ |  |
| Bogus + RPR | .86_[.81, .91]_ | 38.30 |  | .90_[.86, .94]_ | 29.11 |
| Bogus + MD | .85_[.80, .90]_ | 33.29 |  | .85_[.80, .90]_ | 11.22 |
| Bogus + lz | .82_[.77, .87]_ | 23.73 |  | .81_[.75, .86]_ | 4.96 |
| Bogus + Synonyms | .81_[.76, .87]_ | 22.77 |  | .88_[.83, .92]_ | 19.31 |
| Bogus + Gnormed | .81_[.76, .87]_ | 21.35 |  | .81_[.75, .86]_ | 5.38 |
| Bogus + Outfit MSE | .81_[.76, .87]_ | 21.01 |  | .79_[.73, .85]_ | 2.67 |
| Bogus + Infit MSE | .81_[.75, .86]_ | 19.59 |  | .79_[.73, .84]_ | 2.59 |
| Bogus + *r*_pbis_ | .76_[.70, .82]_ | 12.91 |  | .87_[.83, .91]_ | 18.68 |
| Bogus + H_t_ | .78_[.73, .84]_ | 16.60 |  | .83_[.78, .88]_ | 8.27 |
| Bogus + INDCHI | .75_[.69, .81]_ | 8.43 |  | .78_[.72, .84]_ | 1.58 |
| Bogus + Antonyms | .74_[.67, .80]_ | 8.25 |  | .85_[.80, .90]_ | 14.52 |
| Bogus + Time | .74_[.68, .80]_ | 11.20 |  | .89_[.85, .94]_ | 22.96 |
| Bogus + Longstring | - | - |  | .79_[.73, .85]_ | 5.63 |
| Bogus + IRV | - | - |  | .79_[.73, .85]_ | 3.97 |

*Note*. Only indirect indices that turned out to be effective in detecting partially or fully careless responding were examined. The average score on the bogus items could not be computed for one respondent in the careful responding group, because this respondent did not answer any of the bogus items. In addition, the antonym index could not be computed for one respondent in the partially careless responding group (i.e., Careless 33%) and for two respondents in the fully careless responding group (i.e., Careless 100%), because one of the antonym vectors had no variance. Thus, we used linear multiple imputation predictions (across 100 imputations) of logit models as input for the ROC analyses. All indices were coded such that higher scores were more indicative of careless responding. The Bonferroni-corrected critical χ^2^ value for each of the 26 comparisons was 9.62 (i.e., 12 pairwise comparisons in the partially careless responding condition, 14 pairwise comparisons in the fully careless responding condition). If this value is exceeded, the AUCs can be considered significantly different from each other. AUC = area under the receiver operating characteristic curve; Time = average response time per item; Longstring = maximum longstring; IRV = intra-individual response variability; Synonyms = psychometric synonyms; antonyms = psychometric antonyms; RPR = resampled personal reliability; MD = Mahalanobis distance measure for which the calculation was based on subsamples; *r*_pbis_ = person-total correlation coefficient for which the calculation was based on subsamples; Gnormed = global (i.e., the average of the scale-specific scores was computed) normed Guttman error index for polytomous items; H_t_ = global (i.e., the average of the scale-specific scores was computed) person scalability index; lz = global (i.e., the average of the scale-specific scores was computed) standardized log-likelihood index for polytomous items; infit MSE = global (i.e., the average of the scale-specific statistics was computed) Rasch model-based infit mean square error; outfit MSE = global (i.e., the average of the scale-specific statistics was computed) Rasch model-based outfit mean square error; INDCHI = global (i.e., the average of the scale-specific scores was computed) individual contribution to model misfit for which the calculation of the two person-specific model log-likelihood values was based on subsamples.

^a^ A subsample that contained careful respondents and respondents that were partially careless responding was used to determine the classification accuracy (*n* = 239). ^b^ A subsample that contained careful respondents and respondents that were fully careless responding (*n* = 237) was used to determine the classification accuracy.

# Table S8

*Number of Code Lines Needed to Obtain the Final Indirect Index*

| Index | Number of code lines in syntax file |
| --- | --- |
| Time | 1 |
| IRV | 1 |
| Longstring | 44 |
| MD | 58 |
| *r*_pbis_ | 87 |
| Gnormed | 124 |
| H_t_ | 124 |
| Lz | 124 |
| MSE Outfit | 177 |
| MSE Infit | 177 |
| RPR | 228 |
| Synonyms | 237 |
| Antonyms | 237 |
| INDCHI | 958 |

*Note*. Time = average response time per item; Longstring = maximum longstring; IRV = intra-individual response variability; Synonyms = psychometric synonyms; antonyms = psychometric antonyms; RPR = resampled personal reliability; MD = Mahalanobis distance measure for which the calculation was based on subsamples; *r*_pbis_ = person-total correlation coefficient for which the calculation was based on subsamples; Gnormed = global (i.e., the average of the scale-specific scores was computed) normed Guttman error index for polytomous items; H_t_ = global (i.e., the average of the scale-specific scores was computed) person scalability index; lz = global (i.e., the average of the scale-specific scores was computed) standardized log-likelihood index for polytomous items; infit MSE = global (i.e., the average of the scale-specific statistics was computed) Rasch-model based infit mean square error; outfit MSE = global (i.e., the average of the scale-specific statistics was computed) Rasch model-based outfit mean square error; INDCHI = global (i.e., the average of the scale-specific scores was computed) individual contribution to model misfit for which the calculation of the two person-specific model log-likelihood values was based on subsamples.

# Computation of Indirect Careless Responding Indices in Study 2

For computing the indirect indices in Study 2, we only used the selected 60 unidirectionally keyed items of the of the German adaptation (IPIP-5F-30F-R1; Iller et al., 2020) of the International Personality Item Pool (IPIP; Goldberg, 2006). Negatively worded items were inverted prior to the computation of personal reliability, normed Guttman errors, personal scalability, standardized log-likelihood, infit and outfit MSE statistics, and individual contribution to the model misfit. For the computation of all other indices, the raw item scoring was used.

## Measures of Response Time

### Average Time Per Item

The Qualtrics survey tool that we used for the Study 2 allowed us to extract three response time measures for every survey page that was displayed (and all substantive items were displayed on a single web page). ‘First Click’ measures how many milliseconds the page was visible before the respondents clicked the first time (not including the submit button). ‘Last Click’ measures how many milliseconds the page was visible before the respondents clicked the last time (not including the submit button). ‘Page Submit’ measures how many milliseconds the page was visible before the respondent clicked on the submit button. For our analyses, we calculated an average score of the 60 Page Submit response time measures.

## Measures of Response Invariability

### Maximum Longstring

To obtain the maximum longstring, we counted for each respondent the maximum number of identical responses in a row. After rearranging the data from wide to long format, we therefore used the community-contributed Stata function *tsspell* (Cox, 2007) and collapsed (using the max option) the variable _seq for each respondent. If a respondent’s response was missing, the maximum longstring was based on the remaining non-missing responses.

### Intra-Individual Response Variability

To obtain the intra-individual response variability (IRV), we computed the with-person standard deviation over the set of 60 substantive items. We therefore used the Stata function *rowsd*. If a respondent’s response was missing, the IRV was based on the remaining non-missing responses.

## Measure of Within-Person Inconsistency

### Psychometric Synonyms

To obtain the psychometric synonym index, we proceeded as follows. First, we searched the item correlation matrix of the careful responding group for correlation coefficients larger than .60 (Meade & Craig, 2012, p. 442-443) or larger than .50. However, because no coefficient reached this threshold, we started searching the item correlation matrix of the careful responding group for correlation coefficients larger than .45. Out of 1770 potential pairs, we identified 18 pairs meeting the criterion of being correlated larger than .45. Next, we computed the within-person correlation across these 18 pairs for each respondent. If an item pair had a missing value, the within-person correlation was based on the remaining item pairs with no missing values.

### Psychometric Antonyms

To obtain the psychometric antonym index, we proceeded as follows. First, we searched the item correlation matrix of the careful responding group for correlation coefficients lower than -.60 (Meade & Craig, 2012, p. 442-443) or lower than -.50 or lower than -.45. However, because no coefficient reached these thresholds, we started searching the item correlation matrix of the careful responding group for correlation coefficients lower than -.40. Out of 1770 potential pairs, we identified 7 pairs meeting the criterion of being correlated lower than -.40. Next, we computed the within-person correlation across these 7 pairs for each respondent. If an item pair had a missing value, the within-person correlation was based on the remaining item pairs with no missing values. However, the antonym index could not be computed for two respondents in the partially careless responding group (i.e., Careless 33%) and two respondents in the fully careless responding group (i.e., Careless 100%), because one of their antonym vectors had no variance.

### Resampled Personal Reliability

We calculated resampled personal reliability (RPR; Curran, 2016). Compared to conventional personal reliability or even-odd consistency, in which just one set of scale halves is used (e.g., halves of even or odd numbered items), RPR is based on the repeated calculation of personal reliability across several randomly rearranged sets of scale halves. The RPR therefore helps to cancel out random error, that may occur when using a specific (even weird) set of scale halve pairings for the reliability calculation (Curran, 2016).

To obtain the RPR we proceeded as follows: After transposing the data, we first used the Stata command *splitsample* with the *balance* option to randomly split the selected four items of each of the selected 15 facets of the IPIP-5F-30F-R1 into two halves. In this transposed data, we then used the *collapse* command with the *by* option to obtain two scale half scores for each of the 15 facets. Next, we re-transposed the data and transformed the 15 scores of the first halves and the 15 scores of the second halves into a data set in long format. Based on this data set in long-format, we then calculate the within-person correlation between the two scale halves and corrected each of these calculated correlation coefficients for decreased length with the Spearman-Brown formula (Brown, 1910; Spearman, 1910). In the rare cases where this corrected coefficient was less than -1, it was replaced by -1, which ensured that all corrected coefficients lay within the boundaries of -1 and 1. This whole process was repeated for 100 random splits (iterations) and the final RPR was obtained by taking the average of the resulting 100 personal reliabilities.

Across the 100 iterations, the standard deviation of the personal reliabilities within each respondent was on average 0.13 (*SD* = 0.11). This indicated, that using different sets of scale half scores resulted in remarkably different personal reliabilities for each respondent. Because of this variability, we also examined how the personal reliability of each iteration performed compared to the RPR in detecting careless respondents. In 48 of the iterations, personal reliability performed significantly worse than RPR and in only 52 of the iterations, personal reliability performed as well as the RPR (area under the curve [AUC] = .81, *SE* = .02). Accordingly, in none of the iterations did personal reliability perform better than the RPR. These results clearly illustrate that a more trustworthy and accurate index can be obtained when the calculation of personal reliability is based on several randomly rearranged sets of scale halves.

## Sample Norm-Based Inconsistency

### Mahalanobis Distance and Person-Total Correlation

As suggested by Goldammer et al. (2022), we used subsamples to calculate the Mahalanobis distance (MD; Mahalanobis, 1936) and the *r*_pbis_ (Donlon & Fischer, 1968; Curran, 2016, pp. 12-13). This meant, that each respondent in the two careless responding groups was separately merged with the group of careful respondents for the calculation of the indices. The subsample runs resulted in 229 distance and *r*_pbis_ measures for every careful respondent and one distance and *r*_pbis_ measure for every careless respondent. To obtain the final distance and *r*_pbis_ measures for every careful respondent, the 229 values of subsample runs were averaged. The calculation in subsamples (Goldammer et al., 2022) was chosen because the validity of these indices’ grounds on the assumption that the majority of responses was given honestly (Curran, 2016, p. 13). Thus, calculating the indices at once for the total sample (*n* = 341), could have produced biased indices in our case, because careless respondents formed the majority (*n* = 229) in the sample of Study 2. Because the MD can only be computed if no missing item responses are present, we used the non-parametric imputation method (see Niessen et al., 2016, p. 6) that is available in the *PerFit* package (Tendeiro et al., 2016) in R (R Core Team, 2022) before calculating the distance measure. In the 60 substantive items, 22.3% (*n* = 76) of the respondents had at least one missing item response. However, we expected the effect of the imputation on the consistency of the response patterns to be minimal because the vast majority of respondents with missing responses (86%) had only one or two item missing responses, with a maximum of 24 missing item responses. In the case of the *r*_pbis_, the remaining non-missing responses were used for calculation, if respondents had missing item responses. The Stata command *mahascore* with the options *refmeans* and *compute_incov* was used for the computation of the MD. Based on a transposed data frame, the *r*_pbis_ could be computed with the *alpha* command in Stata and the item-rest correlation coefficients were extracted for further analyses purposes.

To check the utility of the subsample-based index calculations, we compared their performance in detecting careless respondents with the performance of distance/correlation indices that were calculated at once in the total sample. For both indices, however, the subsample-based versions of the indices did not perform better than the indices that were calculated at once in the total sample (MD, AUC_Subsample_ = .75; *SE* = .03, AUC_Total_ = .76; *SE* = .03, χ^2^[1] = 0.88, *p* = .35; *r*_pbis_, AUC_Subsample_ = .81; *SE* = .03, AUC_Total_ = .81; *SE* = .03, χ^2^[1] = 1.09, *p* = .30). Thus, the additional effort for calculating the subsample-based versions of MD and *r*_pbis_ did not pay off in terms of higher detection accuracy.

## Model-Based Inconsistency: Nonparametric Person-Fit Indices

### Guttman Error and Scalability Index

To obtain the normed Guttman error index for polytomous items (Gnormed; Emons, 2008; Molenaar, 1991) and the person or transposed scalability index (H_t_; Mokken; 1971; Sijtsma, 1986), we first computed for each of the five domains in IPIP-5F-30F-R1 domain-specific or scale-specific indices. On this basis, we then calculated a global or multi-test extension of Gnormed and H_t_ (Drasgow et al., 1991; Niessen et al., 2016, p. 6) by averaging the values of the scale-specific indices. The *PerFit* package (Tendeiro et al., 2016) was used to calculate Gnormed, and the *mokken* package (Van der Ark, 2007) was used to calculate H_t_. For calculating Gnormed, we used a data set in which rows represented observations and columns variables/items. For calculating H_t_, in contrast, we used a transposed data set in which rows represented variables/items and columns observations. Because Gnormed and H_t_ cannot be computed if missing item responses are present, we used the parametric and non-parametric imputation method that is available in *PerFit* before calculating Gnormed and H_t_ (see Niessen et al., 2016, p. 6). In the 60 substantive items, 22.3% (*n* = 76) of the respondents had at least one missing item response. However, we expected the effect of the imputation on consistency of the response patterns to be minimal, because the vast majority of respondents with missing responses (86%) had only one or two item responses missing, with a maximum of 24 missing item responses.

## Model-Based Inconsistency: Parametric IRT Based Person-Fit Indices

### Standardized Log-Likelihood and Infit and Outfit MSE

To obtain the standardized log-likelihood for polytomous items (lz; Drasgow et al., 1985), and the Rasch model-based infit and outfit mean square error (MSE) statistics (Wright & Stone, 1979), we first computed for each of the five domains in IPIP-5F-30F-R1 domain-specific or scale-specific indices. On this basis, we then calculated a global or multi-test extension of lz and infit and outfit MSE (Drasgow et al., 1991; Niessen et al., 2016, p. 6) by averaging the values of the scale-specific indices. We estimated graded response models (Samejima, 1969) in the *PerFit* package (Tendeiro et al., 2016) in the case of lz, and Rasch rating scale models (Andrich, 1978) in the *TAM* package (Robitzsch et al., 2020) in the case of infit and outfit MSE statistics. Because lz cannot be computed if missing item responses are present, we used the parametric and non-parametric imputation method that is available in *PerFit* before calculating lz (see Niessen et al., 2016, p. 6). In the 60 substantive items, 22.3% (*n* = 76) of the respondents had at least one missing item response. However, we expected the effect of the imputation on consistency of the response patterns to be minimal, because the vast majority of respondents with missing responses (86%) had only one or two item responses missing, with a maximum of 24 missing item responses. When calculating infit and outfit MSE, in contrast, no imputation was necessary, because we used marginal maximum likelihood in the *TAM* package for estimating the Rasch rating scale models, which allowed us to handle missing values trough full information estimation.

## Model-Based Inconsistency: Parametric Covariance-Structure Based Person-Fit Index

### Individual Contribution to Model Misfit

As suggested by Goldammer et al. (2022), we used subsamples to calculate the individual contribution to model misfit (INDCHI; Reise & Widaman, 1999, pp. 7-8). We did so because the second part of formula for calculating the person-specific log-likelihood (i.e., the basis for calculating the INDCHI) is equivalent to the squared Mahalanobis distance (Reise & Widaman, 1999, p. 7), which in turn grounds on the assumption that the majority of responses was given carefully, to be a valid measure for aberrant responding (Curran, 2016, p. 13). Thus, calculating the INDCHI at once for the total sample (*n* = 341) could have produced a biased index in our case, because careless respondents formed the majority (*n* = 229) in the sample of Study 2.

To obtain scale-specific INDCHI indices for each respondent, we first estimated a saturated model and a one-factor model for each scale and extracted the person-specific log-likelihood values of both models. However, we only calculated the INDCHI for the scales extraversion, agreeableness, conscientiousness, and neuroticism, because the fit of the measurement model (i.e., a one-factor model) of the domain/scale openness to experience was so poor that the model estimation regularly failed to converge (especially in the subsample runs) or could only be achieved with arbitrary additional constraints on loadings.

In the subsample runs, we obtained for every careful respondent 229 person-specific log-likelihood values for the saturated model and 229 person-specific log-likelihood values for the one-factor model. For every careless respondent, one person-specific log-likelihood value for the saturated model and one person-specific log-likelihood value for the one-factor model were obtained from the subsample runs. The final two person-specific log-likelihood values for every careful respondent were obtained by averaging the 229 values of each of the two models. Next, the scale-specific INDCHI values could be computed for each respondent by subtracting the person-specific log-likelihood of the saturated model from the person-specific log-likelihood of the substantive factor model and by multiplying this difference by -2. Finally, we computed a global or multi-test extension of the INDCHI by averaging the values of the scale-specific indices. All models were estimated with robust maximum likelihood estimation (MLR) in Mplus Version 8.8 (Muthén & Muthén, 1998-2017). Missing values were handled through the implemented default when using MLR (i.e., full information maximum likelihood estimation FIML).

To check the utility of the subsample-based index calculation, we compared its performance in detecting careless respondents with the performance of an INDCHI index that was calculated at once in the total sample. Whereas the subsample-based version detected careless respondents significantly better than chance (AUC = .76; *SE* = .03), the version in which the INDCHI was calculated at once in the total sample did not perform better than chance (AUC = .51; *SE* = .03). Thus, the additional effort for calculating the subsample-based version of the INDCHI allowed us to obtain an index with significant detection accuracy, which would have been not the case if we had calculated the INDCHI naïvely (i.e., at once in the total sample).

# Table S9

*List of the 60 Selected Items of the IPIP-5F-30F-R1 Used in Study 2*

| Domain | Facet | German item wording | English item wording (taken from DeepL Translator) |
| --- | --- | --- | --- |
| Extraversion | Sociability | Er mag Grossanlässe. | He likes big events. |
| Extraversion | Sociability | Er hat das Bedürfnis, Teil einer Gruppe zu sein. | He feels the need to be part of a group. |
| Extraversion | Sociability | Er pflegt viele soziale Kontakte. | He maintains many social contacts. |
| Extraversion | Sociability | Er liebt das gesellige Beisammensein mit vielen Menschen. | He likes socializing with many people. |
| Extraversion | Assertiveness | Er leitet gerne andere Personen an. | He likes to lead other people. |
| Extraversion | Assertiveness | Er hat eine hohe Überzeugungskraft. | He has a high power of persuasion. |
| Extraversion | Assertiveness | Es fällt ihm leicht, seinen Anliegen Gehör zu verschaffen. | It is easy for him to make his concerns heard. |
| Extraversion | Assertiveness | Er vertritt seine Meinung mit Nachdruck. | He is emphatic in representing his own opinion. |
| Extraversion | Activity | Er ist immer auf dem Sprung. | He is always on the go. |
| Extraversion | Activity | In seiner Freizeit unternimmt er viel. | He does a lot in his free time. |
| Extraversion | Activity | Er mag es, viele Dinge gleichzeitig zu tun. | He likes to do many things at once. |
| Extraversion | Activity | Er steckt voller Tatendrang. | He is full of drive. |
| Agreeableness | Kindness | Kooperativ zu sein ist ihm wichtiger, als wettbewerbsfähig zu sein. | Being cooperative is more important to him than being competitive. |
| Agreeableness | Kindness | Der Schmerz anderer berührt ihn tief. | The pain of others touches him deeply. |
| Agreeableness | Kindness | Solidarität mit Schwächeren ist für ihn die wichtigste Tugend. | Solidarity with weaker people is the most important virtue for him. |
| Agreeableness | Kindness | Das Wohlergehen aller Menschen liegt ihm sehr am Herzen. | The welfare of all people is very close to his heart. |
| Agreeableness | Altruism | Er legt für alle ein gutes Wort ein. | He puts in a good word for everyone. |
| Agreeableness | Altruism | Er hält anderen den Rücken frei. | He covers the back of others. |
| Agreeableness | Altruism | Damit es anderen gut geht stellt er seine eigenen Anliegen zurück. | In order for others to do well, he puts his own concerns aside. |
| Agreeableness | Altruism | Er sorgt dafür, dass sich andere wohlfühlen. | He makes others feel comfortable. |

(continued)

Table S9 (continued)

| Domain | Facet | German item wording | English item wording (taken from DeepL Translator) |
| --- | --- | --- | --- |
| Agreeableness | Trust | Er hat das feste Vertrauen, dass ihm andere Menschen wohlgesinnt sind. | He has firm faith that other people are benevolent to him. |
| Agreeableness | Trust | Er ist gutgläubig. | He has good faith. |
| Agreeableness | Trust | Er geht immer davon aus, dass andere Menschen gute Absichten verfolgen. | He always assumes that other people have good intentions. |
| Agreeableness | Trust | Er glaubt fest an das Gute im Menschen. | He firmly believes in the goodness of people. |
| Conscientiousness | Orderliness | Er achtet stets darauf, Dinge an ihren Platz zurückzustellen. | He always makes sure to put things back in their place. |
| Conscientiousness | Orderliness | Unordnung stört ihn sehr. | Disorder bothers him a lot. |
| Conscientiousness | Orderliness | Er erlediget Dinge gerne in einer bestimmten Reihenfolge. | He likes to do things in a certain order. |
| Conscientiousness | Orderliness | Er hält systematisch Ordnung. | He keeps order systematically. |
| Conscientiousness | Striving for achievement | Er arbeitet hart. | He works hard. |
| Conscientiousness | Striving for achievement | Er erledigt seine Aufgaben mit hoher Einsatzbereitschaft. | He completes his tasks with a high level of commitment. |
| Conscientiousness | Striving for achievement | Er will die Erwartungen stets übertreffen. | He always wants to exceed expectations. |
| Conscientiousness | Striving for achievement | Ehrgeizige Ziele beflügeln ihn. | Ambitious goals inspire him. |
| Conscientiousness | Sense of duty | Er ist absolut zuverlässig. | He is absolutely reliable. |
| Conscientiousness | Sense of duty | Er handelt streng nach seinen Prinzipien. | He acts strictly according to his principles. |
| Conscientiousness | Sense of duty | Pünktlichkeit ist für ihn das A und O. | Punctuality is the be-all and end-all for him. |
| Conscientiousness | Sense of duty | Er setzt alles daran, seinen Pflichten nachzukommen. | He does everything in his power to fulfill his duties. |
| Neuroticism | Anxiousness | Er macht sich schnell Sorgen. | He worries quickly. |
| Neuroticism | Anxiousness | Er macht sich oft Gedanken über Dinge, die möglicherweise schief laufen könnten. | He often worries about things that might go wrong. |
| Neuroticism | Anxiousness | Er lässt sich von Ereignissen leicht beunruhigen. | He is easily disturbed by events. |
| Neuroticism | Anxiousness | Er ist oft nervös. | He is often nervous. |

(continued)

Table S9 (continued)

| Domain | Facet | German item wording | English item wording (taken from DeepL Translator) |
| --- | --- | --- | --- |
| Neuroticism | Timidity | Ihm fällt es schwer, andere kennenzulernen. | He finds it difficult to get to know others. |
| Neuroticism | Timidity | Von schwierigen sozialen Situationen lässt er sich einschüchtern. | He is intimidated by difficult social situations. |
| Neuroticism | Timidity | Er wird verlegen, wenn sich die Blicke anderer Personen auf ihn richten. | He becomes embarrassed when other people’s eyes are on him. |
| Neuroticism | Vulnerability | Es ist ihm unangenehm, vor anderen zu sprechen. | He is uncomfortable speaking in front of others. |
| Neuroticism | Vulnerability | Unter Druck verliert er rasch seine Ruhe. | He quickly loses his composure under pressure. |
| Neuroticism | Vulnerability | Er hat oft das Gefühl, kritische Situationen nicht meistern zu können. | He often feels that he cannot cope with critical situations. |
| Neuroticism | Vulnerability | Ungünstige Ereignisse werfen ihn leicht aus der Bahn. | Unfavorable events easily throw him off course. |
| Openness to experience | Fantasy | In Stresssituationen wird er von seinen Gefühlen überwältigt. | In stressful situations he is overwhelmed by his feelings. |
| Openness to experience | Fantasy | Er geniesst Reisen in Fantasiewelten. | He enjoys traveling to fantasy worlds. |
| Openness to experience | Fantasy | Er mag es, seinen Gedanken nachzuhängen. | He likes to indulge his thoughts. |
| Openness to experience | Fantasy | Er hat ein ausgesprochen lebhaftes Vorstellungsvermögen. | He has a very vivid imagination. |
| Openness to experience | Actions | Seine Fantasie hilft ihm in vielen Lebenslagen. | His imagination helps him in many situations in life. |
| Openness to experience | Actions | Veränderung ist ihm lieber als Routine. | He prefers change to routine. |
| Openness to experience | Actions | Er sucht die Abwechslung. | He seeks variety. |
| Openness to experience | Actions | Er strebt nach neuen Erfahrungen. | He strives for new experiences. |
| Openness to experience | Ideas | Es ist ihm wichtig, dass Vielfalt sein Leben prägt. | It is important to him that variety shapes his life. |
| Openness to experience | Ideas | Er mag es, komplexe Problemstellungen zu bearbeiten. | He likes to work on complex problems. |
| Openness to experience | Ideas | Er beschäftigt sich mit anspruchsvollen Inhalten. | He deals with challenging content. |
| Openness to experience | Ideas | Erkenntnisse aus fremden Wissensgebieten wecken seine Neugier. | Findings from foreign fields of knowledge arouse his curiosity. |

## Manipulation Check Study 2

As in Study 1, we compared the ratings of the experimental groups with the ratings of a norm group to examine whether our manipulation worked. The participants in the norm group in Study 2 were 56 German-speaking prospective professional officers and NCOs who attended the basic training courses for prospective officers and NCOs in the fall of 2021 at the military academy and the professional NCO school. They were on average 26.27 years old (*SD* = 3.14) and predominantly men (*n* = 54, 96.4%). Most of them had either completed the nine years of compulsory schooling (*n* = 33, 58.9%) or a certified apprenticeship (*n* = 18, 30.1%). Only five participants in the norm group (8.9%) indicated the attest apprenticeship as their highest educational level. In our instruction, we told them that we had filmed new videos of assessment center exercises to facilitate the training of new assessors at our assessment centers. Their rating would help us examine whether the NCO candidate in the video was perceived by the viewer as we intended. If so, the videos could be used for assessor trainings and even experimental studies. They were then shown the NCO candidate’s self-presentation video and afterwards asked to complete a questionnaire about that NCO candidate (i.e., the 60 IPIP-5F-30F-R1 items).

Again, as expected, the scale scores of the fully careless respondents were biased towards the scale midpoint (Goldammer et al., 2020; Huang et al., 2015). Compared to the scale scores of the norm group and careful responding group (whose scores did not differ from each other), the fully careless respondents (i.e., Careless 100%) had lower scores on the extraversion, agreeableness, and conscientiousness scales, and a higher score on the neuroticism scale (see Table S10). Contrary to our expectation, however, the partially careless responding group (i.e., Careless 33%) mostly did not provide scale scores that were significantly different than those of norm and careful responding group (see Table S10).

Next, we examined whether the means of three indirect careless response indices (average response time per item, IRV, RPR) differed between the norm and the three experimental groups. The mean comparisons revealed the expected pattern. Compared to the norm and the careful responding group, the fully careless respondents (i.e., Careless 100%) responded much faster and less consistently (see Table S10). In the case of the partially careless responding group (i.e., Careless 33%), the effects were in the expected direction as well; however, only the post-hoc tests for the RPR reached the Bonferroni-corrected critical z-value of 3.28.

Lastly, we then examined the differences in the correlation of the response vectors. The correlation between the normative response vector and the response vectors of the respondents in the three experimental conditions was as follows: Careful (*M* = .69, *SD* = .21), Careless 33% (*M* = .52, *SD* = .20), Careless 100% (*M* = .30, *SD* = .33). In line with our expectation, the response vectors of careful respondents correlated significantly more strongly with the normative response vector than the response vectors of partially careless respondents (i.e., Careless 33%; Estimate_Δcorrelation_ = .17, *SE* = .03, *z* = 6.07, *p* < .001), and fully careless respondents (i.e., Careless 100%; Estimate_Δcorrelation_ = .39, *SE* = .04, *z* = 10.68, *p* < .001). In addition, the response vectors of partially careless respondents correlated significantly more strongly with the normative response vector than the response vectors of fully careless respondents (Estimate_Δcorrelation_ = .22, *SE* = .04, *z* = 6.16, *p* < .001). Based on these results, the manipulation was successful; we thus proceeded with the main analyses.

# Table S10

*Comparison of Substantive Scale Scores and Selected Indirect Careless Responding Indices Between Norm and Experimental Conditions in Study 2*

|  | Response conditions | | | | | | |  |  |
| --- | --- | --- | --- | --- | --- | --- | --- | --- | --- |
|  | Norm |  | Careful |  | Careless 33% |  | Careless 100% |  |  |
| Measure | *M* (*SD*) |  | *M* (*SD*) |  | *M* (*SD*) |  | *M* (*SD*) | χ^2^(3) | Cohen’s *d* |
| Subtantive Scales |  |  |  |  |  |  |  |  |  |
| Extraversion | 4.22_a_ (0.45) |  | 4.39_a_ (0.42) |  | 4.23_a_ (0.46) |  | 3.95_b_ (0.52) | 49.65^*^ | 0.35_PC_/0.93_FC_ |
| Agreeableness | 4.51_ab_ (0.37) |  | 4.67_a_ (0.46) |  | 4.46_b_ (0.52) |  | 4.07_c_ (0.65) | 65.73^*^ | 0.44_PC_/1.06_FC_ |
| Conscientiousness | 4.33_a_ (0.38) |  | 4.54_a_ (0.44) |  | 4.36_a_ (0.48) |  | 3.99_b_ (0.58) | 62.56^*^ | 0.38_PC_/1.04_FC_ |
| Neuroticism | 2.73_a_ (0.37) |  | 2.59_a_ (0.63) |  | 2.79_a_ (0.53) |  | 3.30_b_ (0.66) | 80.30^*^ | -0.34_PC_/-1.10_FC_ |
| Openness to experience | 3.94_ab_ (0.50) |  | 4.03_b_ (0.54) |  | 3.93_ab_ (0.50) |  | 3.71_a_ (0.54) | 21.20^*^ | 0.20_PC_/0.59_FC_ |
| Careless Response Indices |  |  |  |  |  |  |  |  |  |
| Time | 7.04_a_ (2.10) |  | 6.91_a_ (1.67) |  | 6.80_a_ (1.86) |  | 4.42_b_ (1.75) | 160.65^*^ | 0.06_PC_/1.45_FC_ |
| IRV | 1.05_a_ (0.19) |  | 1.23_b_ (0.25) |  | 1.31_b_ (0.24) |  | 1.19_b_ (0.34) | 60.06^*^ | -0.36_PC_/0.12_FC_ |
| RPR | 0.81_a_ (0.09) |  | 0.78_a_ (0.15) |  | 0.57_b_ (0.29) |  | 0.29_c_ (0.52) | 166.07^*^ | 0.94_PC_/1.29_FC_ |

*Note.* Means and standard deviations are based on univariate sample statistics for the norm group (*n* = 56), the careful responding condition (*n* = 112), the partially careless responding (i.e., Careless 33%) condition (*n* = 114), and the fully careless responding (i.e., Careless 100%) condition (*n* = 115). In each row, means with different subscripts are significantly different from each other (i.e., α = .025/48, with the critical z-value of 3.28). χ^2^ = χ^2^ values were obtained by conducting Wald tests of parameter constraints that were based on robust full information maximum likelihood estimation (MLR) in Mplus Version 8.8. *d*_PC_ = Cohen’s *d* for the mean comparison between Careless 33% and Careful, *d*_FC_ = Cohen’s *d* for the mean comparison between Careless 100% and Careful.

^*^ Larger than the critical χ^2^(3) value of 12.36 (i.e., α = .05/8), which indicates global inequality between the means.

# Table S11

*Descriptive Statistics of the Substantive Measures and Careless Response Indices for the Careful Responding Group in Study 2*

| Scale | 1. | 2. | 3. | 4. | 5. | 6. | 7. | 8. | 9. | 10. | 11. | 12. | 13. | 14. | 15. | 16. | 17. | 18. | 19. | 20. |
| --- | --- | --- | --- | --- | --- | --- | --- | --- | --- | --- | --- | --- | --- | --- | --- | --- | --- | --- | --- | --- |
| 1. EX | 1 |  |  |  |  |  |  |  |  |  |  |  |  |  |  |  |  |  |  |  |
| 2. AG | .461** | 1 |  |  |  |  |  |  |  |  |  |  |  |  |  |  |  |  |  |  |
| 3. CO | .545** | .364** | 1 |  |  |  |  |  |  |  |  |  |  |  |  |  |  |  |  |  |
| 4. EM | -.490** | -.317** | -.178 | 1 |  |  |  |  |  |  |  |  |  |  |  |  |  |  |  |  |
| 5. OP | .540** | .473** | .472** | -.262** | 1 |  |  |  |  |  |  |  |  |  |  |  |  |  |  |  |
| 6. Bogus | -.090 | -.141 | .080 | .323** | -.073 | 1 |  |  |  |  |  |  |  |  |  |  |  |  |  |  |
| 7. Time | -.093 | .028 | -.090 | .087 | -.055 | -.154 | 1 |  |  |  |  |  |  |  |  |  |  |  |  |  |
| 8. Longstring | .121 | -.045 | .094 | .154 | .338** | .072 | -.001 | 1 |  |  |  |  |  |  |  |  |  |  |  |  |
| 9. IRV | .433** | .498** | .381** | -.633** | .178 | -.104 | -.051 | -.264** | 1 |  |  |  |  |  |  |  |  |  |  |  |
| 10. Synonyms | .424** | .294** | .306** | -.530** | .274** | -.103 | -.136 | .014 | .348** | 1 |  |  |  |  |  |  |  |  |  |  |
| 11. Antonyms | -.413** | -.388** | -.119 | .666** | -.306** | .193* | .127 | .015 | -.360** | -.503** | 1 |  |  |  |  |  |  |  |  |  |
| 12. RPR | .468** | .427** | .268** | -.625** | .225* | -.256** | .001 | -.126 | .321** | .691** | -.644** | 1 |  |  |  |  |  |  |  |  |
| 13. MD | -.235* | -.020 | -.079 | .214* | -.224* | .083 | .192* | -.189* | .360** | -.344** | .382** | -.490** | 1 |  |  |  |  |  |  |  |
| 14. *r*_pbis_ | .587** | .451** | .274** | -.816** | .367** | -.346** | -.084 | -.088 | .332** | .561** | -.756** | .742** | -.534** | 1 |  |  |  |  |  |  |
| 15. Gnormed | -.002 | .133 | .125 | -.021 | -.052 | .166 | .059 | -.197* | .674** | -.155 | .185 | -.339** | .783** | -.329** | 1 |  |  |  |  |  |
| 16. H_t_ | .336** | .314** | .052 | -.579** | .169 | -.384** | -.122 | -.153 | .199* | .471** | -.666** | .613** | -.538** | .827** | -.372** | 1 |  |  |  |  |
| 17. lz | .007 | -.133 | -.090 | .011 | .092 | -.131 | -.124 | .260** | -.640** | .198* | -.184 | .337** | -.822** | .331** | -.961** | .403** | 1 |  |  |  |
| 18. Infit MSE | .187* | .298** | .263** | -.177 | .116 | .109 | .042 | -.208* | .786** | -.015 | .060 | -.193* | .701** | -.149 | .963** | -.237* | -.915** | 1 |  |  |
| 19. Outfit MSE | .165 | .270** | .246** | -.145 | .086 | .124 | .056 | -.227* | .767** | -.041 | .093 | -.215* | .719** | -.183 | .970** | -.270** | -.937** | .995** | 1 |  |
| 20. INDCHI | -.071 | -.142 | -.020 | .172 | -.127 | .200* | .254** | -.043 | .115 | -.071 | .416** | -.267** | .356** | -.373** | .408** | -.397** | -.459** | .341** | .377** | 1 |
| *M* | 4.386 | 4.674 | 4.536 | 2.591 | 4.033 | 1.375 | 6.907 | 3.768 | 1.226 | 0.618 | -0.784 | 0.784 | 59.345 | 0.694 | 0.091 | 0.170 | 0.817 | 0.677 | 0.666 | 0.653 |
| *SD* | 0.424 | 0.461 | 0.440 | 0.630 | 0.536 | 0.496 | 1.672 | 1.115 | 0.246 | 0.245 | 0.247 | 0.154 | 13.418 | 0.206 | 0.052 | 0.062 | 0.694 | 0.341 | 0.327 | 1.041 |

*Note.* The careful responding group included 112 participants. All reported Pearson correlation coefficients and means and standard deviations are based on 112 observations. EX = Extraversion; AG = Agreeableness; CO = Conscientiousness; EM = Negative Emotionality; OP = Open-Mindedness; bogus = average score of the three bogus items; time = average response time per item; longstring = maximum longstring; IRV = intra-individual response variability; synonyms = psychometric synonyms; antonyms = psychometric antonyms; RPR = resampled personal reliability; MD = Mahalanobis distance measure for which the calculation was based on subsamples; *r*_pbis_ = person-total correlation coefficient for which the calculation was based on subsamples; Gnormed = global (i.e., the average of the scale-specific scores was computed) normed Guttman error index for polytomous items; H_t_ = global (i.e., the average of the scale-specific scores was computed) person scalability index; lz = global (i.e., the average of the scale-specific scores was computed) standardized log-likelihood index for polytomous items; infit MSE = global (i.e., the average of the scale-specific statistics was computed) Rasch model-based infit mean square error; outfit MSE = global (i.e., the average of the scale-specific statistics was computed) Rasch model-based outfit mean square error; INDCHI = global (i.e., the average of the scale-specific scores was computed) individual contribution to model misfit for which the calculation of the two person-specific model log-likelihood values was based on subsamples.

* *p* < .05 (two-tailed).

** *p* < .01 (two-tailed).

# Table S12

*Descriptive Statistics of the Substantive Measures and Careless Response Indices for the Partially Careless Responding Group (i.e., Careless 33%) in Study 2*

| Scale | 1. | 2. | 3. | 4. | 5. | 6. | 7. | 8. | 9. | 10. | 11. | 12. | 13. | 14. | 15. | 16. | 17. | 18. | 19. | 20. |
| --- | --- | --- | --- | --- | --- | --- | --- | --- | --- | --- | --- | --- | --- | --- | --- | --- | --- | --- | --- | --- |
| 1. EX | 1 |  |  |  |  |  |  |  |  |  |  |  |  |  |  |  |  |  |  |  |
| 2. AG | .426** | 1 |  |  |  |  |  |  |  |  |  |  |  |  |  |  |  |  |  |  |
| 3. CO | .383** | .453** | 1 |  |  |  |  |  |  |  |  |  |  |  |  |  |  |  |  |  |
| 4. EM | -.281** | -.141 | -.295** | 1 |  |  |  |  |  |  |  |  |  |  |  |  |  |  |  |  |
| 5. OP | .383** | .392** | .358** | -.154 | 1 |  |  |  |  |  |  |  |  |  |  |  |  |  |  |  |
| 6. Bogus | -.097 | -.168 | -.238* | .215* | -.166 | 1 |  |  |  |  |  |  |  |  |  |  |  |  |  |  |
| 7. Time | -.046 | .055 | -.037 | -.090 | -.069 | .006 | 1 |  |  |  |  |  |  |  |  |  |  |  |  |  |
| 8. Longstring | .005 | .008 | -.020 | .182 | .150 | -.075 | -.069 | 1 |  |  |  |  |  |  |  |  |  |  |  |  |
| 9. IRV | .122 | .051 | .309** | -.383** | -.017 | .169 | .054 | -.250** | 1 |  |  |  |  |  |  |  |  |  |  |  |
| 10. Synonyms | .245** | .279** | .309** | -.571** | .257** | -.291** | .047 | -.089 | -.002 | 1 |  |  |  |  |  |  |  |  |  |  |
| 11. Antonyms | -.383** | -.530** | -.384** | .435** | -.370** | .325** | -.109 | .037 | .065 | -.473** | 1 |  |  |  |  |  |  |  |  |  |
| 12. RPR | .368** | .479** | .392** | -.628** | .255** | -.384** | .082 | -.039 | .071 | .651** | -.557** | 1 |  |  |  |  |  |  |  |  |
| 13. MD | -.236* | -.330** | -.107 | .219* | -.219* | .379** | -.035 | -.140 | .561** | -.498** | .574** | -.481** | 1 |  |  |  |  |  |  |  |
| 14. *r*_pbis_ | .537** | .579** | .498** | -.720** | .374** | -.371** | .085 | -.029 | .031 | .632** | -.687** | .770** | -.601** | 1 |  |  |  |  |  |  |
| 15. Gnormed | -.174 | -.272** | .011 | .085 | -.174 | .354** | -.053 | -.111 | .798** | -.355** | .388** | -.367** | .801** | -.481** | 1 |  |  |  |  |  |
| 16. H_t_ | .360** | .411** | .236* | -.336** | .260** | -.267** | .125 | -.019 | -.012 | .421** | -.462** | .508** | -.524** | .753** | -.397** | 1 |  |  |  |  |
| 17. lz | .155 | .239* | .015 | -.085 | .175 | -.352** | .018 | .137 | -.791** | .409** | -.381** | .378** | -.829** | .477** | -.969** | .401** | 1 |  |  |  |
| 18. Infit MSE | -.043 | -.128 | .152 | .014 | -.066 | .329** | -.061 | -.123 | .849** | -.300** | .295** | -.278** | .771** | -.356** | .975** | -.320** | -.949** | 1 |  |  |
| 19. Outfit MSE | -.067 | -.140 | .134 | .025 | -.088 | .341** | -.058 | -.120 | .843** | -.317** | .318** | -.291** | .791** | -.380** | .978** | -.345** | -.960** | .996** | 1 |  |
| 20. INDCHI | -.311** | -.370** | -.193* | .075 | -.172 | .403** | .006 | -.039 | .571** | -.257** | .337** | -.294** | .542** | -.443** | .782** | -.282** | -.757** | .711** | .730** | 1 |
| *M* | 4.229 | 4.456 | 4.361 | 2.788 | 3.929 | 1.656 | 6.797 | 3.658 | 1.313 | 0.340 | -0.475 | 0.566 | 73.303 | 0.528 | 0.162 | 0.110 | -0.194 | 1.071 | 1.069 | 2.496 |
| *SD* | 0.462 | 0.520 | 0.484 | 0.531 | 0.498 | 0.723 | 1.864 | 1.211 | 0.245 | 0.318 | 0.437 | 0.286 | 13.171 | 0.206 | 0.086 | 0.066 | 1.029 | 0.505 | 0.490 | 3.267 |

*Note.* The partially careless responding group included 114 participants. All reported Pearson correlation coefficients and means and standard deviations are based on 114 observations except for the estimates that involve the antonym index, which are based on 112 observations (because the antonym vectors of two participants had no variance). EX = Extraversion; AG = Agreeableness; CO = Conscientiousness; EM = Negative Emotionality; OP = Open-Mindedness; bogus = average score of the three bogus items; time = average response time per item; longstring = maximum longstring; IRV = intra-individual response variability; synonyms = psychometric synonyms; antonyms = psychometric antonyms; RPR = resampled personal reliability; MD = Mahalanobis distance measure for which the calculation was based on subsamples; *r*_pbis_ = person-total correlation coefficient for which the calculation was based on subsamples; Gnormed = global (i.e., the average of the scale-specific scores was computed) normed Guttman error index for polytomous items; H_t_ = global (i.e., the average of the scale-specific scores was computed) person scalability index; lz = global (i.e., the average of the scale-specific scores was computed) standardized log-likelihood index for polytomous items; infit MSE = global (i.e., the average of the scale-specific statistics was computed) Rasch model-based infit mean square error; outfit MSE = global (i.e., the average of the scale-specific statistics was computed) Rasch model-based outfit mean square error; INDCHI = global (i.e., the average of the scale-specific scores was computed) individual contribution to model misfit for which the calculation of the two person-specific model log-likelihood values was based on subsamples.

* *p* < .05 (two-tailed).

** *p* < .01 (two-tailed).

# Table S13

*Descriptive Statistics of the Substantive Measures and Careless Response Indices for the Fully Careless Responding Group (i.e., Careless 100%) in Study 2*

| Scale | 1. | 2. | 3. | 4. | 5. | 6. | 7. | 8. | 9. | 10. | 11. | 12. | 13. | 14. | 15. | 16. | 17. | 18. | 19. | 20. |
| --- | --- | --- | --- | --- | --- | --- | --- | --- | --- | --- | --- | --- | --- | --- | --- | --- | --- | --- | --- | --- |
| 1. EX | 1 |  |  |  |  |  |  |  |  |  |  |  |  |  |  |  |  |  |  |  |
| 2. AG | .597** | 1 |  |  |  |  |  |  |  |  |  |  |  |  |  |  |  |  |  |  |
| 3. CO | .575** | .638** | 1 |  |  |  |  |  |  |  |  |  |  |  |  |  |  |  |  |  |
| 4. EM | -.287** | -.433** | -.370** | 1 |  |  |  |  |  |  |  |  |  |  |  |  |  |  |  |  |
| 5. OP | .570** | .380** | .372** | -.164 | 1 |  |  |  |  |  |  |  |  |  |  |  |  |  |  |  |
| 6. Bogus | -.293** | -.434** | -.415** | .575** | -.006 | 1 |  |  |  |  |  |  |  |  |  |  |  |  |  |  |
| 7. Time | .257** | .312** | .227* | -.293** | .036 | -.465** | 1 |  |  |  |  |  |  |  |  |  |  |  |  |  |
| 8. Longstring | .159 | .083 | .092 | .022 | .182 | -.021 | .071 | 1 |  |  |  |  |  |  |  |  |  |  |  |  |
| 9. IRV | -.201* | -.125 | -.190* | -.036 | -.259** | .167 | -.144 | -.430** | 1 |  |  |  |  |  |  |  |  |  |  |  |
| 10. Synonyms | .312** | .374** | .370** | -.341** | .093 | -.287** | .321** | .053 | .073 | 1 |  |  |  |  |  |  |  |  |  |  |
| 11. Antonyms | -.315** | -.353** | -.337** | .343** | -.326** | .342** | -.453** | -.031 | .169 | -.422** | 1 |  |  |  |  |  |  |  |  |  |
| 12. RPR | .373** | .474** | .420** | -.391** | -.015 | -.473** | .499** | .021 | -.071 | .563** | -.418** | 1 |  |  |  |  |  |  |  |  |
| 13. MD | -.388** | -.405** | -.448** | .301** | -.257** | .405** | -.424** | -.391** | .773** | -.371** | .500** | -.469** | 1 |  |  |  |  |  |  |  |
| 14. *r*_pbis_ | .618** | .707** | .682** | -.783** | .374** | -.657** | .486** | .150 | -.309** | .442** | -.484** | .596** | -.658** | 1 |  |  |  |  |  |  |
| 15. Gnormed | -.316** | -.315** | -.379** | .267** | -.212* | .459** | -.382** | -.310** | .857** | -.170 | .372** | -.377** | .877** | -.594** | 1 |  |  |  |  |  |
| 16. H_t_ | .424** | .478** | .490** | -.564** | .204* | -.611** | .496** | .254** | -.308** | .292** | -.391** | .477** | -.594** | .827** | -.597** | 1 |  |  |  |  |
| 17. lz | .296** | .273** | .339** | -.193* | .174 | -.386** | .389** | .346** | -.853** | .223* | -.390** | .411** | -.900** | .537** | -.975** | .545** | 1 |  |  |  |
| 18. Infit MSE | -.216* | -.212* | -.287** | .214* | -.148 | .420** | -.367** | -.307** | .865** | -.134 | .338** | -.332** | .858** | -.517** | .989** | -.546** | -.971** | 1 |  |  |
| 19. Outfit MSE | -.235* | -.231* | -.304** | .224* | -.157 | .433** | -.372** | -.309** | .862** | -.146 | .347** | -.343** | .864** | -.534** | .991** | -.565** | -.972** | .999** | 1 |  |
| 20. INDCHI | -.268** | -.228* | -.295** | .203* | -.047 | .354** | -.350** | -.179 | .664** | -.062 | .331** | -.377** | .671** | -.460** | .847** | -.503** | -.834** | .843** | .847** | 1 |
| *M* | 3.946 | 4.074 | 4.000 | 3.302 | 3.715 | 2.604 | 4.424 | 4.278 | 1.189 | 0.236 | -0.349 | 0.285 | 73.525 | 0.301 | 0.165 | 0.069 | -0.177 | 1.000 | 1.004 | 3.282 |
| *SD* | 0.520 | 0.651 | 0.580 | 0.662 | 0.542 | 1.225 | 1.746 | 2.777 | 0.335 | 0.331 | 0.444 | 0.522 | 17.245 | 0.333 | 0.115 | 0.083 | 1.473 | 0.643 | 0.645 | 3.903 |

*Note.* The fully careless responding group included 115 participants. All reported Pearson correlation coefficients and means and standard deviations are based on 115 observations except for the estimates that involve the antonym index, which are based on 113 observations (because the antonym vectors of two participants had no variance). EX = Extraversion; AG = Agreeableness; CO = Conscientiousness; EM = Negative Emotionality; OP = Open-Mindedness; bogus = average score of the three bogus items; time = average response time per item; longstring = maximum longstring; IRV = intra-individual response variability; synonyms = psychometric synonyms; antonyms = psychometric antonyms; RPR = resampled personal reliability; MD = Mahalanobis distance measure for which the calculation was based on subsamples; *r*_pbis_ = person-total correlation coefficient for which the calculation was based on subsamples; Gnormed = global (i.e., the average of the scale-specific scores was computed) normed Guttman error index for polytomous items; H_t_ = global (i.e., the average of the scale-specific scores was computed) person scalability index; lz = global (i.e., the average of the scale-specific scores was computed) standardized log-likelihood index for polytomous items; infit MSE = global (i.e., the average of the scale-specific statistics was computed) Rasch model-based infit mean square error; outfit MSE = global (i.e., the average of the scale-specific statistics was computed) Rasch model-based outfit mean square error; INDCHI = global (i.e., the average of the scale-specific scores was computed) individual contribution to model misfit for which the calculation of the two person-specific model log-likelihood values was based on subsamples.

* *p* < .05 (two-tailed).

** *p* < .01 (two-tailed).

# Table S14

*Ordered Accuracies of the 14 Indirect Indices in Detecting Partially Careless (i.e., Careless 33%)* *Responding and Corresponding Pairwise Comparison Results in Study 2*

| Index | AUC_[95% CI]_ | Sen95 ^a^ | Sen99 ^b^ | 1. | 2. | 3. | 4. | 5. | 6. | 7. | 8. | 9. | 10. | 11. | 12. | 13. |
| --- | --- | --- | --- | --- | --- | --- | --- | --- | --- | --- | --- | --- | --- | --- | --- | --- |
| 1. lz | .81_[.75, .86]_ | .26 | .20 |  |  |  |  |  |  |  |  |  |  |  |  |  |
| 2. Gnormed | .78_[.72, .84]_ | .25 | .19 | 7.36 |  |  |  |  |  |  |  |  |  |  |  |  |
| 3. MD | .77_[.72, .83]_ | .27 | .17 | 3.18 | 0.22 |  |  |  |  |  |  |  |  |  |  |  |
| 4. RPR | .77_[.71, .83]_ | .20 | .13 | 0.91 | 0.08 | 0.00 |  |  |  |  |  |  |  |  |  |  |
| 5. Outfit MSE | .76_[.70, .82]_ | .25 | .15 | 15.47 | 6.68 | 0.30 | 0.07 |  |  |  |  |  |  |  |  |  |
| 6. Synonyms | .76_[.70, .82]_ | .25 | .10 | 1.47 | 0.31 | 0.13 | 0.18 | 0.00 |  |  |  |  |  |  |  |  |
| 7. *r*_pbis_ | .76_[.69, .82]_ | .05 | .01 | 1.82 | 0.45 | 0.29 | 0.44 | 0.01 | 0.02 |  |  |  |  |  |  |  |
| 8. H_t_ | .76_[.69, .82]_ | .09 | .03 | 1.97 | 0.53 | 0.32 | 0.25 | 0.02 | 0.02 | 0.01 |  |  |  |  |  |  |
| 9. Infit MSE | .75_[.69, .81]_ | .21 | .16 | 18.90 | 12.32 | 0.98 | 0.27 | 13.41 | 0.05 | 0.02 | 0.01 |  |  |  |  |  |
| 10. INDCHI | .74_[.68, .81]_ | .35 | .22 | 3.59 | 1.40 | 0.83 | 0.74 | 0.35 | 0.27 | 0.22 | 0.17 | 0.09 |  |  |  |  |
| 11. Antonyms | .73_[.66, .80]_ | .32 | .03 | 4.12 | 2.00 | 2.00 | 2.30 | 0.68 | 1.09 | 1.38 | 0.82 | 0.32 | 0.09 |  |  |  |
| 12. Time | .52_[.45, .60]_ | .11 | .01 | 33.85 | 28.80 | 25.10 | 27.54 | 24.26 | 23.27 | 21.34 | 22.17 | 22.08 | 18.44 | 15.71 |  |  |
| 13. Longstring | .45_[.38, .52]_ | .06 | .01 | 49.35 | 42.86 | 41.01 | 51.57 | 36.19 | 41.67 | 44.29 | 43.91 | 33.56 | 35.09 | 36.16 | 2.65 |  |
| 14. IRV | .40_[.33, .47]_ | .04 | .02 | 46.72 | 39.44 | 44.19 | 78.30 | 32.44 | 66.80 | 69.73 | 59.19 | 29.99 | 41.69 | 53.22 | 5.62 | 1.15 |

*Note.* A subsample from Study 2 including only participants in the careful responding condition, and the partially responding condition was used to determine the classification accuracy (*n* = 226). Because the antonym index could not be computed for two respondents in the partially careless responding group (i.e., Careless 33%), all reported estimates and test statistics are based on 100 multiply imputed data sets. All indices were coded such that higher scores were more indicative of careless responding. Values in the matrix part represent the *F*-values for the pairwise comparisons with one degree of freedom for the numerator and degrees of freedom that ranged from 535184.5 to ∞ for the denominator. With the most conservative denominator value of 535184.5, the Bonferroni-corrected critical *F*-value for each of the 91 comparisons was 11.94; if this value is exceeded, the two AUCs can be considered significantly different from each other, and the corresponding cells in the matrix part are shown in gray. AUC = area under the receiver operating characteristic curve; time = average response time per item; longstring = maximum longstring; IRV = intra-individual response variability; synonyms = psychometric synonyms; antonyms = psychometric antonyms; RPR = resampled personal reliability; MD = Mahalanobis distance measure for which the calculation was based on subsamples; *r*_pbis_ = person-total correlation coefficient for which the calculation was based on subsamples; Gnormed = global (i.e., the average of the scale-specific scores was computed) normed Guttman error index for polytomous items; H_t_ = global (i.e., the average of the scale-specific scores was computed) person scalability index; lz = global (i.e., the average of the scale-specific scores was computed) standardized log-likelihood index for polytomous items; infit MSE = global (i.e., the average of the scale-specific statistics was computed) Rasch model-based infit mean square error; outfit MSE = global (i.e., the average of the scale-specific statistics was computed) Rasch model-based outfit mean square error; INDCHI = global (i.e., the average of the scale-specific scores was computed) individual contribution to model misfit for which the calculation of the two person-specific model log-likelihood values was based on subsamples.

^a^ Sensitivity of the index at specificity level of 95% (i.e., false-positive rate of 5%). ^b^ Sensitivity of the index at specificity level of 99% (i.e., false-positive rate of 1%).

# Table S15

*Ordered Accuracies of the 14 Indirect Indices in Detecting Fully Careless (i.e., Careless 100%)* *Responding and Corresponding Pairwise Comparison Results in Study 2*

| Index | AUC_[95% CI]_ | Sen95 ^a^ | Sen99 ^b^ | 1. | 2. | 3. | 4. | 5. | 6. | 7. | 8. | 9. | 10. | 11. | 12. | 13. |
| --- | --- | --- | --- | --- | --- | --- | --- | --- | --- | --- | --- | --- | --- | --- | --- | --- |
| 1. *r*_pbis_ | .86_[.81, .91]_ | .41 | .20 |  |  |  |  |  |  |  |  |  |  |  |  |  |
| 2. Time | .85_[.80, .90]_ | .54 | .37 | 0.02 |  |  |  |  |  |  |  |  |  |  |  |  |
| 3. RPR | .84_[.79, .89]_ | .45 | .38 | 0.95 | 0.21 |  |  |  |  |  |  |  |  |  |  |  |
| 4. H_t_ | .84_[.78, .89]_ | .35 | .18 | 1.09 | 0.23 | 0.00 |  |  |  |  |  |  |  |  |  |  |
| 5. Synonyms | .82_[.77, .88]_ | .34 | .13 | 2.01 | 0.67 | 0.43 | 0.21 |  |  |  |  |  |  |  |  |  |
| 6. Antonyms | .82_[.76, .87]_ | .49 | .07 | 3.31 | 0.90 | 0.63 | 0.46 | 0.04 |  |  |  |  |  |  |  |  |
| 7. INDCHI | .78_[.72, .84]_ | .45 | .30 | 5.54 | 3.61 | 3.29 | 2.69 | 1.24 | 1.04 |  |  |  |  |  |  |  |
| 8. MD | .73_[.67, .80]_ | .42 | .31 | 17.58 | 10.27 | 12.29 | 11.11 | 6.94 | 7.35 | 2.76 |  |  |  |  |  |  |
| 9. lz | .70_[.63, .77]_ | .36 | .27 | 21.39 | 15.69 | 17.35 | 15.60 | 10.77 | 9.85 | 8.02 | 4.58 |  |  |  |  |  |
| 10. Gnormed | .69_[.62, .76]_ | .32 | .29 | 24.50 | 18.14 | 19.94 | 18.52 | 12.22 | 11.90 | 10.18 | 8.22 | 2.60 |  |  |  |  |
| 11. Outfit MSE | .64_[.57, .72]_ | .27 | .22 | 31.87 | 26.64 | 27.65 | 26.29 | 18.34 | 18.29 | 20.39 | 21.63 | 31.55 | 33.39 |  |  |  |
| 12. Infit MSE | .64_[.56, .71]_ | .26 | .21 | 32.87 | 27.91 | 28.69 | 27.33 | 19.11 | 19.01 | 21.35 | 22.82 | 32.40 | 38.03 | 4.37 |  |  |
| 13. Longstring | .54_[.47, .62]_ | .17 | .02 | 42.21 | 43.17 | 37.89 | 36.75 | 32.46 | 31.19 | 20.36 | 10.10 | 6.10 | 5.25 | 2.51 | 2.23 |  |
| 14. IRV | .53_[.45, .61]_ | .16 | .09 | 49.65 | 41.11 | 43.49 | 38.94 | 42.27 | 36.36 | 18.45 | 9.62 | 5.70 | 4.78 | 2.34 | 2.08 | 0.06 |

*Note.* A subsample from Study 2 including only participants in the careful responding condition, and the partially responding condition was used to determine the classification accuracy (*n* = 227). Because the antonym index could not be computed for two respondents in the fully careless responding group (i.e., Careless 100%), all reported estimates and test statistics are based on 100 multiply imputed data sets. All indices were coded such that higher scores were more indicative of careless responding. Values in the matrix part represent the *F*-values for the pairwise comparisons with one degree of freedom for the numerator and degrees of freedom that ranged from 620572.9 to ∞ for the denominator. With the most conservative denominator value of 620572.9, the Bonferroni-corrected critical *F*-value for each of the 91 comparisons was 11.94; if this value is exceeded, the two AUCs can be considered significantly different from each other, and the corresponding cells in the matrix part are shown in gray. AUC = area under the receiver operating characteristic curve; time = average response time per item; longstring = maximum longstring; IRV = intra-individual response variability; synonyms = psychometric synonyms; antonyms = psychometric antonyms; RPR = resampled personal reliability; MD = Mahalanobis distance measure for which the calculation was based on subsamples; *r*_pbis_ = person-total correlation coefficient for which the calculation was based on subsamples; Gnormed = global (i.e., the average of the scale-specific scores was computed) normed Guttman error index for polytomous items; H_t_ = global (i.e., the average of the scale-specific scores was computed) person scalability index; lz = global (i.e., the average of the scale-specific scores was computed) standardized log-likelihood index for polytomous items; infit MSE = global (i.e., the average of the scale-specific statistics was computed) Rasch model-based infit mean square error; outfit MSE = global (i.e., the average of the scale-specific statistics was computed) Rasch model-based outfit mean square error; INDCHI = global (i.e., the average of the scale-specific scores was computed) individual contribution to model misfit for which the calculation of the two person-specific model log-likelihood values was based on subsamples.

^a^ Sensitivity of the index at specificity level of 95% (i.e., false-positive rate of 5%). ^b^ Sensitivity of the index at specificity level of 99% (i.e., false-positive rate of 1%).

# Table S16

*Incremental Validity Beyond Average Score on Bogus Items of Each Indirect Index in Study 2*

|  | Careless 33% ^a^ | |  | Careless 100% ^b^ | |
| --- | --- | --- | --- | --- | --- |
| Index | AUC_[95% CI]_ | χ^2^(1) |  | AUC_[95% CI]_ | χ^2^(1) |
| Bogus (reference) | .62_[.55, .69]_ |  |  | .83_[.78, .88]_ |  |
| Bogus + lz | .81_[.75, .86]_ | 22.42 |  | .85_[.80, .90]_ | 1.48 |
| Bogus + Gnormed | .78_[.72, .84]_ | 17.08 |  | .85_[.80, .90]_ | 1.93 |
| Bogus + MD | .78_[.73, .84]_ | 17.85 |  | .85_[.80, .90]_ | 2.13 |
| Bogus + RPR | .77_[.71, .83]_ | 17.96 |  | .87_[.82, .92]_ | 5.54 |
| Bogus + Outfit MSE | .77_[.71, .83]_ | 14.65 |  | .84_[.79, .89]_ | 0.87 |
| Bogus + Synonyms | .77_[.70, .83]_ | 16.11 |  | .88_[.83, .92]_ | 6.92 |
| Bogus + *r*_pbis_ | .76_[.70, .82]_ | 17.55 |  | .88_[.83, .92]_ | 7.26 |
| Bogus + H_t_ | .76_[.70, .83]_ | 15.69 |  | .87_[.83, .91]_ | 5.50 |
| Bogus + Infit MSE | .76_[.70, .82]_ | 13.43 |  | .84_[.79, .89]_ | 0.74 |
| Bogus + INDCHI | .74_[.68, .81]_ | 10.36 |  | .87_[.82, .91]_ | 4.94 |
| Bogus + Antonyms | .74_[.68, .81]_ | 11.40 |  | .87_[.82, .92]_ | 5.49 |
| Bogus + Time | - | - |  | .89_[.85, .93]_ | 9.32 |

*Note*. Only indirect indices that turned out be effective in detecting partially or fully careless responding were examined. Because the antonym index could not be computed for two respondents in the partially careless responding group (i.e., Careless 33%) and for two respondents in the fully careless responding group (i.e., Careless 100%), we used linear multiple imputation predictions (across 100 imputations) of logit models as input for the ROC analyses. All indices were coded such that higher scores were more indicative of careless responding. The Bonferroni-corrected critical χ^2^ value for each of the 23 comparisons was 9.40 (i.e., 11 pairwise comparisons in the partially careless responding condition, 12 pairwise comparisons in the fully careless responding condition). If this value is exceeded, the AUC of Bogus items and that of the indirect index can be considered significantly different from each other. AUC = area under the receiver operating characteristic curve; time = average response time per item; Synonyms = psychometric synonyms; antonyms = psychometric antonyms; RPR = resampled personal reliability; MD = Mahalanobis distance measure for which the calculation was based on subsamples; *r*_pbis_ = person-total correlation coefficient for which the calculation was based on subsamples; Gnormed = global (i.e., the average of the scale-specific scores was computed) normed Guttman error index for polytomous items; H_t_ = global (i.e., the average of the scale-specific scores was computed) person scalability index; lz = global (i.e., the average of the scale-specific scores was computed) standardized log-likelihood index for polytomous items; infit MSE = global (i.e., the average of the scale-specific statistics was computed) Rasch model-based infit mean square error; outfit MSE = global (i.e., the average of the scale-specific statistics was computed) Rasch model-based outfit mean square error; INDCHI = global (i.e., the average of the scale-specific scores was computed) individual contribution to model misfit for which the calculation of the two person-specific model log-likelihood values was based on subsamples.

^a^ A subsample that contained careful respondents and respondents that had partially careless responding was used to determine the classification accuracy (*n* = 226). ^b^ A subsample that contained careful respondents and respondents that had fully careless responding (*n* = 227) was used to determine the classification accuracy.

# Study 3

Studies 1 and 2 showed that indirect indices are effective in detecting different types of careless responding and may even outperform direct measures (i.e., bogus items). Study 3 aimed to replicate these findings with a non-military sample of Amazon Mechanical Turk (MTurk) workers and with a shift of the rating perspective (i.e., respondents were asked to rate their own personality) and thus helped us to address research questions 1 to 4. In addition, the type of item presentation was manipulated as well, such that respondents either completed the survey in a format in which each item was displayed on a single webpage or in a format in which several items were displayed together per webpage (i.e., matrix presentation). With this design we compared the indices detection performance across the types of item presentation and thus examined research question 6 as well.

It is important to note, however, that the manipulation turned out to be not successful and thus prevented us from conducting the main analyses in Study 3. For transparency reasons and to inform researchers interested in conducting studies on careless responding in online-populations like MTurk, we report the methods of Study 3 and the results of the manipulation check in the following sections. In addition, we highlight in a study-specific discussion three potential reasons for the failed manipulation and offer recommendations for using the convenience sampling platform MTurk in future (careless responding) research.

## Method of Study 3

### Participants and Procedure

The data for Study 3 was gathered in late fall 2021. The participants were MTurk workers with U.S. MTurk accounts and an approval rate of at least 98%. On the MTurk website and the starting web page of the online survey, MTurk workers were informed that the study would examine the impact of careless (unmotivated) responding on the quality of personality test results. For this purpose, participants would have to complete a personality questionnaire at two time points. The first survey would take place right now and the second one would follow in two weeks. We further informed potential participants that during the study, some participants would be instructed to complete the questionnaire(s) carelessly, whereas others would be instructed to complete the questionnaire(s) accurately. Lastly, we announced that we would pay every participant $1 for completing the first questionnaire and another $3 for completing the second questionnaire.

Of the 811 workers that participated at the t1 measurement, 10 were not contacted for the t2 measurement because they did not provide a correct MTurk worker ID (*n* = 1), or because the same worker ID was used twice (*n* = 9, [i.e., 8 protocols]). Of the 792 MTurk workers that were contacted for the second measurement two weeks later, 608 workers eventually participated. However, 8 participants (i.e., 16 protocols) were excluded because their worker ID was listed twice, and 2 participants were excluded because they provided an incorrect worker ID.

The final sample therefore contained 590 participants that completed the questionnaires at both measurements. The participants in this sample were on average 38.3 years old (*SD* = 11.43) and most of them were men (*n* = 343, 58.1%). The educational level of the participants was rather high: More than three quarters (*n* = 483, 81.9%) had a bachelor’s or higher degree, and only a minority had an associate’s degree or a lower education level (*n* = 107, 18.1%). Three quarters of the participants were employed (*n* = 452, 76.6%) and worked 40 hours per week or more.

### Experimental Conditions and Survey Arrangement

Once an MTurk worker decided to participate at the first measurement (which also included the four sociodemographic questions), they were assigned either to the condition in which each personality item was presented on a single webpage (*n* = 307) or to the condition in which the items were presented as a matrix—seven items per webpage (*n* = 283). In both item presentation conditions, the order of items was randomized. At the first measurement, all participants were instructed to respond to the questionnaire items as they applied to them. They were further informed that there were no right or wrong answers and that they just had to respond to each item as honestly as possible.

At the second measurement two weeks later, participants completed the same questionnaire in the same item presentation format in which they had completed the first questionnaire (again with randomized item order) but were randomly assigned to the careful responding condition (*n*_single item presentation_ = 103; *n*_matrix presentation_ = 94) or to one of the two careless responding conditions (i.e., partially careless—careless responding to the last third of the questionnaire items [*n*_single item presentation_ = 102; *n*_matrix presentation_ = 96], fully careless—careless responding to all questionnaire items [*n*_single item presentation_ = 102; *n*_matrix presentation_ = 93]).

Participants assigned to the careful condition were again instructed to respond to the questionnaire items as they applied to them and as honestly as possible. In contrast, participants in the partially careless responding condition were asked to complete the first survey part (i.e., 66% of the items) accurately and honestly, and the second survey part (i.e., 33% of the items) with the following instruction: They should complete the remaining questionnaire as fast as possible, and they should not pay attention to the item content. They were further told that they would receive a bonus of $1 if they could provide responses that could not be detected as careless or false by our algorithms. Participants in the fully careless responding condition received the following instruction at the beginning of the survey: They should complete the questionnaire as fast as possible, and they should not pay attention to the item content. They were further told that they would receive a bonus of $1 if they could provide responses that could not be detected as being careless of false by our algorithms. The bonus of $1 was paid to all participants in the two careless responding conditions, independent of their response patterns.

### Substantive Measures

The Big Five Inventory-2 (BFI-2; Soto & John, 2017) was used as substantive measure and consisted of the five trait scales extraversion (α_Careful_ = .69_t1_/.71_t2_, α_Partially careless_ = .74_t1_/.73_t2_, α_Fully careless_ = .73_t1_/.74_t2_), agreeableness (α_Careful_ = .81_t1_/.84_t2_, α_Partially careless_ = .78_t1_/.76_t2_, α_Fully careless_ = .83_t1_/.81_t2_), conscientiousness (α_Careful_ = .87_t1_/.88_t2_, α_Partially careless_ = .87_t1_/.86_t2_, α_Fully careless_ = .87_t1_/.86_t2_), negative emotionality (α_Careful_ = .82_t1_/.83_t2_, α_Partially careless_ = .83_t1_/.83_t2_, α_Fully careless_ = .84_t1_/.83_t2_), and open-mindedness (α_Careful_ = .81_t1_/.83_t2_, α_Partially careless_ = .81_t1_/.81_t2_, α_Fully careless_ = .82_t1_/.81_t2_). The respondents rated all 60 bidirectionally keyed items (i.e., 30 positively and 30 negatively worded items) of the BFI-2 on a Likert scale ranging from 1 = *completely disagree* to 6 = *completely agree*.

### Direct Careless Responding Measure

We used the same three bogus items as in Studies 1 and 2. As the substantive measure, these three items were rated on a Likert scale from 1 = *completely disagree* to 6 = *completely agree*. For the main analyses, an average score was computed across the 3 items (α_Careful_ = .90_t1_/.88_t2_, α_Partially careless_ = .90_t1_/.85_t2_, α_Fully careless_ = .90_t1_/.88_t2_). If a respondent’s response was missing, the average score was based on the remaining non-missing responses.

### Indirect Careless Responding Measures

We used the same indirect careless responding indices as in Studies 1 and 2. For computing these indirect indices, only the 60 items of the BFI-2 were used.

### Careless Responding Criterion and Analytical Procedure

The indices’ classification accuracy of careless and careful responders at t2 was the main outcome variable in Study 3. The respondents’ data that we gathered at t1 was only used for the manipulation check. For the main analyses, we used the same analytical procedure as in Studies 1 and 2.

## Manipulation Check Study 3

To check whether our manipulation worked, we first examined whether the change in the substantive measures across the two measurements was significantly different across the response conditions (see Table S17). We therefore estimated a three-group latent change/difference score model (see McArdle & Hamagami, 2001; Newsom, 2015) for each of the five substantive measures in each of the two item-presentation modes. In addition, we also examined the within-person correlation of the t1-t2 item response vectors. If our manipulation worked, there should be a significant interaction of the within- and between-subject factor, indicated by a significant inequality of the Δ*M* values. In other words, whereas we expected the scale scores of respondents in the careful group to have not changed from the first to the second measurement, we expected the scale scores of the respondents in the careless responding groups to have changed towards the scale midpoint (Goldammer et al., 2020; Huang, et al., 2015). In addition, we expected the within-person correlation of the t1-t2 response vectors to be significantly higher for careful respondents than for respondents in the careless responding groups.

In contrast to our expectation, however, the difference in change of the substantive measures from the first to the second measurement turned out to be not significantly different across the response conditions (see Table S17). In line with these results, the within-person correlations of the t1-t2 response vectors was not significantly different across the across the response conditions as well—neither in the single item presentation mode, χ^2^(2) = 0.67, *p* = 0.72 (Estimate_Careful_ = .42, *SE* = .04; Estimate_Partially careless_ = .40, *SE* = .04; Estimate_Fully careless_ = .38, *SE* = .04) nor in the matrix item presentation mode, χ^2^(2) = 0.95, *p* = 0.62 (Estimate_Careful_ = .40, *SE* = .04; Estimate_Partially careless_ = .46, *SE* = .04; Estimate_Fully careless_ = .41, *SE* = .04). Based on these results, we concluded that our manipulation had failed and therefore decided to not proceed with the main analyses.

## Discussion of Study 3

There are at least three potential reasons why the manipulation was not successful; we discuss these reasons to provide pointers for future studies on careless responding with online populations. First, respondents that were instructed to respond carelessly may have answered the items at t2 carefully to produce an unobtrusive response pattern and thus to enhance their chances of getting the bonus of $1. Second, a large proportion of respondents may have just wanted to get their money with the least possible effort and thus rushed through the survey at t2 in a rather careless manner, even though they may have been instructed to respond otherwise (i.e., carefully or partially careless). Lastly, trained survey participants, such MTurk workers, may have thought our study results could have been used to better screen for unmotivated/careless responding in future studies (which is true), which in turn could have made it harder for them in future assignments on MTurk to get easy money by completing surveys in a careless manner. Thus, they purposefully misreported at t1 and/or at t2 to prevent us from developing better screening techniques.

Our finding of the failed manipulation and the explanations that we offer are also in line with the results of studies that recently examined the data quality provided by MTurk workers (e.g., Chandler et al., 2015, Peer et al., 2017). For instance, a large proportion (i.e., 20%-50% depending on the cut-off) of MTurk workers failed the attention check questions in Peer et al. (2017), even though they were paid fairly (i.e., $1 for a task that took 10 min to complete). In addition, Peer et al. (2017) also found MTurk workers to be less naïve than respondents of other comparable convenience sampling platforms (e.g., Prolific Academic or CrowdFlower) when confronted with experimental tasks, which in turn has been shown to reduce effect sizes of experiments (Chandler et al., 2015). Lastly, Peer et al. (2017) also showed that MTurk workers were more dishonest than respondents of other platforms (e.g., Prolific Academic or CrowdFlower), especially when it comes to bonus payments (i.e., they claimed more than they were owed).

Taken together, researchers interested in examining careless responding via the convenience sampling platform MTurk may need to take certain precautionary steps to ensure data quality and effects of sufficient sizes: First, researchers may need to use less conventional or less transparent manipulations that are novel even to experienced test-takers (e.g., giving them a second task or confronting them with a distractor while they are completing the survey). In addition, researchers should consider unexpected/unwanted response behaviors that are provoked by announcing bonus payments. Finally, oversampling may be necessary so that unusable data can be cleaned without fear that the sample is too small for substantive analyses.

# Table S17

*Comparisons of t1-t2 Scale Score Mean Differences Across the Response Conditions in Study 3 Separately Conducted for the Item Presentation Modes*

|  | Careful | | | | | | |  | Careless 33% | | | | | | |  | Careless 100% | | | | | | |  |  |  |
| --- | --- | --- | --- | --- | --- | --- | --- | --- | --- | --- | --- | --- | --- | --- | --- | --- | --- | --- | --- | --- | --- | --- | --- | --- | --- | --- |
|  | t1 | |  | t2 | |  |  |  | t1 | |  | t2 | |  |  |  | t1 | |  | t2 | |  |  |  | Δ*M* comparisons | |
| Scale | *M* | *SD* |  | *M* | *SD* |  | Δ*M* |  | *M* | *SD* |  | *M* | *SD* |  | Δ*M* |  | *M* | *SD* |  | *M* | *SD* |  | Δ*M* |  | χ^2^(2) | |
| Item presentation mode: 1 item per survey page (i.e., as single item) | | | | | | | | | | | | | | | | | | | | | | | | | | |
| Extraversion | 3.68 | 0.59 |  | 3.69 | 0.56 |  | 0.01 |  | 3.64 | 0.64 |  | 3.57 | 0.72 |  | -0.07 |  | 3.72 | 0.72 |  | 3.66 | 0.72 |  | -0.06 |  | 2.78 | |
| Agreeableness | 4.11 | 0.72 |  | 4.13 | 0.74 |  | 0.02 |  | 4.12 | 0.71 |  | 4.14 | 0.65 |  | 0.02 |  | 4.02 | 0.74 |  | 3.92 | 0.70 |  | -0.10 |  | 2.95 | |
| Conscientiousness | 4.31 | 0.81 |  | 4.36 | 0.83 |  | 0.05 |  | 4.26 | 0.87 |  | 4.26 | 0.74 |  | 0.01 |  | 4.20 | 0.84 |  | 4.21 | 0.79 |  | 0.01 |  | 0.63 | |
| Negative Emotionality | 3.00 | 0.70 |  | 2.92 | 0.71 |  | -0.08 |  | 3.16 | 0.71 |  | 3.04 | 0.66 |  | -0.13 |  | 3.14 | 0.81 |  | 3.14 | 0.76 |  | 0.01 |  | 4.98 | |
| Open-Mindedness | 4.00 | 0.73 |  | 4.05 | 0.71 |  | 0.06 |  | 3.86 | 0.70 |  | 3.87 | 0.68 |  | 0.01 |  | 3.97 | 0.78 |  | 3.96 | 0.74 |  | -0.01 |  | 1.46 | |
| Item presentation mode: 7 items per survey page (i.e., as matrix) | | | | | | | | | | | | | | | | | | | | | | | | | | |
| Extraversion | 3.71 | 0.70 |  | 3.72 | 0.73 |  | 0.01 |  | 3.55 | 0.83 |  | 3.55 | 0.82 |  | 0.00 |  | 3.60 | 0.70 |  | 3.68 | 0.69 |  | 0.09 |  | 1.78 | |
| Agreeableness | 4.00 | 0.84 |  | 4.00 | 0.81 |  | 0.00 |  | 4.09 | 0.77 |  | 4.03 | 0.76 |  | -0.06 |  | 4.08 | 0.84 |  | 4.06 | 0.83 |  | -0.02 |  | 1.19 | |
| Conscientiousness | 4.25 | 0.94 |  | 4.27 | 0.90 |  | 0.03 |  | 4.23 | 0.96 |  | 4.15 | 0.99 |  | -0.08 |  | 4.27 | 0.93 |  | 4.31 | 0.93 |  | 0.04 |  | 3.82 | |
| Negative Emotionality | 2.94 | 0.86 |  | 2.99 | 0.85 |  | 0.05 |  | 3.19 | 0.99 |  | 3.19 | 0.98 |  | 0.00 |  | 3.12 | 0.89 |  | 3.07 | 0.92 |  | -0.05 |  | 2.50 | |
| Open-Mindedness | 3.97 | 0.84 |  | 3.98 | 0.85 |  | 0.01 |  | 4.25 | 0.89 |  | 4.22 | 0.86 |  | -0.02 |  | 4.00 | 0.84 |  | 3.93 | 0.84 |  | -0.07 |  | 1.44 | |

*Note.* Means and standard deviations are based on univariate sample statistics for the careful responding condition (*n*_single item presentation_ = 103; *n*_matrix presentation_ = 94), the partially careless responding (i.e., Careless 33%) condition (*n*_single item presentation_ = 102; *n*_matrix presentation_ = 96), and the fully careless responding (i.e., Careless 100%) condition (*n*_single item presentation_ = 102; *n*_matrix presentation_ = 93). Reported Delta estimates may not exactly match reported t1 and t2 values due to rounding error. χ^2^ values were obtained by conducting Wald tests of parameter constraints that were based on robust full information maximum likelihood estimation (MLR) in Mplus Version 8.4. t1 = first measurement, t2 = second measurement two weeks later; Δ*M* = estimated latent difference between t2 and t1.

^*^ Larger than the critical χ^2^(2) value of 10.60 (i.e., α = .05/10), which indicates global inequality between the Δ*M* estimates.

# Computation of Indirect Careless Responding Indices in Studies 4 and 5

For computing the indirect indices in Studies 4 and 5, we used only the 60 items from the Big-Five Inventory-2 (BFI-2; Soto & John, 2017). Negatively worded items were inverted prior to the computation of personal reliability, normed Guttman errors, personal scalability, standardized log-likelihood, infit and outfit MSE statistics, and individual contribution to the model misfit. For the computation of all other indices, the raw item scoring was used. For all indices, we calculated two versions: First, a t1 version for which only the item responses of the first measurement were used. Second, a t2 version for which only the item responses of the second measurement were used. Because Studies 4 and 5 were designed identically (i.e., identical regarding substantive measures, experimental conditions, layout) except for the type of direct careless responding measure (bogus vs. instructed response items), we calculated all indirect indices in an overall sample (*n* = 962) in which participants in Study 4 and those in Study 5 were included. Whenever the index calculation involved an implicit or explicit comparison with a norm, the participants in both careful responding conditions were used as reference.

## Measures of Response Time

### Average Time Per Item

The Qualtrics survey tool that we used for the Studies 4 and 5 allowed us to extract three response time measures for every survey page that was displayed (and all substantive items were displayed on a single web page). ‘First Click’ measures how many milliseconds the page was visible before the respondents clicked the first time (not including the submit button). ‘Last Click’ measures how many milliseconds the page was visible before the respondents clicked the last time (not including the submit button). ‘Page Submit’ measures how many milliseconds the page was visible before the respondent clicked on the submit button. For our analyses, we calculated an average score of the 60 Page Submit response time measures.

## Measures of Response Invariability

### Maximum Longstring

To obtain the maximum long string, we counted for each respondent the maximum number of identical responses in a row. After rearranging the data from wide to long format, we therefore used the community-contributed Stata function *tsspell* (Cox, 2007) and collapsed (using the max option) the variable _seq for each respondent. If a respondent’s response was missing, the maximum longstring was based on the remaining non-missing responses.

### Intra-Individual Response Variability

To obtain the intra-individual response variability (IRV), we computed the within-person standard deviation over the set of 60 substantive items. We therefore used the Stata function *rowsd*. If a respondent’s response was missing, the IRV was based on the remaining non-missing responses.

## Measure of Within-Person Inconsistency

### Psychometric Synonyms

To obtain the psychometric synonym index, we proceeded as follows. First, we searched the item correlation matrix of the two careful responding groups (*n*_Study4 & Study5_ = 481) at the second measurement for correlation coefficients larger than .60 (Meade & Craig, 2012, p. 442-443). Out of 1,770 potential pairs, we identified 31 pairs meeting the criterion of being correlated larger than .60. Next, we computed the within-person correlation across these 31 pairs for each respondent. If an item pair had a missing value, the within-person correlation was based on the remaining item pairs with no missing values. Because of missing values or lack variance in one of the synonym vectors, however, the synonym index at the second measurement could not be computed for 14 respondents in the fully careless responding group (i.e., Careless 100%) in Study 4 and for 8 respondents in the fully careless responding group (i.e., Careless 100%) in Study 5.

### Psychometric Antonyms

To obtain the psychometric synonym index, we proceeded as follows. First, we searched the item correlation matrix of the two careful responding groups (*n*_Study4 & Study5_ = 481) at the second measurement for correlation coefficients lower than -.60 (Meade & Craig, 2012, p. 442-443). Out of 1,770 potential pairs, we identified 19 pairs meeting the criterion of being correlated lower than -.60. Next, we computed the within-person correlation across these 19 pairs for each respondent. If an item pair had a missing value, the within-person correlation was based on the remaining item pairs with no missing values. Because of missing values or lack variance in one of the antonym vectors, however, the antonym index at the second measurement could not be computed for 14 respondents in the fully careless responding group (i.e., Careless 100%) in Study 4, for 12 respondents in the fully careless responding group (i.e., Careless 100%) in Study 5, and for 1 respondent in the incentivized careful responding group in Study 5.

### Resampled Personal Reliability

We calculated the resampled personal reliability (RPR; Curran, 2016) as follows. Compared to the conventional personal reliability or even-odd consistency, in which just one set of scale halves is used (e.g., halves of even or odd numbered items), RPR is based on the repeated calculation of the personal reliability across several randomly rearranged sets of scale halves. RPR therefore helps to cancel out random error that may occur when using a specific (even weird) set of scale halve pairings for the reliability calculation (Curran, 2016).

To obtain the RPR we proceeded as follows: After transposing the data, we first used the Stata command *splitsample* with the *balance* option to randomly split the items of each of the 15 facets of the BFI-2 into two halves. In this transposed data, we then used the *collapse* command with the *by* option to obtain two scale half scores for each of the 15 facets. Next, we re-transposed the data and transformed the 15 scores of the first halves and the 15 scores of the second halves into a data set in long format. Based on this data set in long format, we then calculated the within-person correlation between the two scale halves and corrected each of these calculated correlation coefficients for decreased length with the Spearman-Brown formula (Brown, 1910; Spearman, 1910). In the rare cases where this corrected coefficient was less than -1, it was replaced with -1, which ensured that all corrected coefficients lay within the boundaries of -1 and 1. This whole process was repeated for 100 random splits (iterations), and the final RPR was obtained by taking the average of the resulting 100 personal reliabilities.

Across the 100 iterations in Studies 4 and 5, the standard deviation of the personal reliabilities within each respondent was on average 0.07 (*SD* = 0.06) at the first measurement and 0.15 (*SD* = 0.16) at the second measurement. This indicated that using different sets of scale half scores resulted in remarkably different personal reliabilities for each respondent, especially at the second measurement, wherehalf of the participants were instructed to respond carelessly. Because of this variability, we also examined how the personal reliability of each iteration performed compared to the RPR in detecting careless respondents. We therefore ran ROC analyses with the combined sample of Studies 4 and 5 (*n* = 962) in which the two careful responding groups were coded with 0 and the two careless responding groups with 1. In 91 of the iterations, personal reliability performed significantly worse than RPR and in only 9 of the iterations, personal reliability performed as well as RPR (area under the curve [AUC] = .90, *SE* = .01). Accordingly, in none of the iterations did personal reliability perform better than RPR. These results clearly illustrate that a more trustworthy and accurate index can be obtained when the calculation of personal reliability is based on several randomly rearranged sets of scale halves.

## Sample Norm-Based Inconsistency

### Mahalanobis Distance and Person-Total Correlation

As suggested by Goldammer et al. (2022), we used subsamples to calculate the Mahalanobis distance (MD; Mahalanobis, 1936) and the *r*_pbis_ (Donlon & Fischer, 1968; Curran, 2016, pp. 12-13). This meant that each respondent in the two careless responding groups was separately merged with the group of careful respondents (i.e., normal and incentivized careful responding groups; *n*_Study4 & Study5_ = 481) for the calculation of the indices. The subsample runs resulted in 481 distance and *r*_pbis_ measures for every careful respondent and one distance and *r*_pbis_ measure for every careless respondent. To obtain the final distance and *r*_pbis_ measures for every careful respondent, the 481 values of subsample runs were averaged. The calculation in subsamples (Goldammer et al., 2022) was chosen because the validity of these indices grounds on the assumption that the majority of responses was given honestly (Curran, 2016, p. 13). Thus, calculating the indices at once for the total sample (*n* = 962) could have produced biased indices in our case, because careless respondents formed 50% (*n* = 481) in the sample of Studies 4 and Study 5.

Because the MD can only be computed if no missing item responses are present, we used the non-parametric imputation method (see Niessen et al., 2016, p. 6) that is available in the *PerFit* package (Tendeiro et al., 2016) in R (R Core Team, 2022) before calculating the distance measure for the first and second measurement. In the 60 substantive items at the first measurement, 95.5% (*n* = 919) completed the questionnaire with no missing responses, 4% (*n* = 38) with one missing response and 0.5% (*n* = 5) with two missing responses. In the 60 substantive items at the second measurement 11.2% (*n* = 108) of the respondents had at least one missing item response; however, the vast majority of them (85%) had only one or two item responses missing. Given this pattern of missingness, we expected the effect of the imputation on the consistency of the response patterns to be minimal for the vast majority of the response protocols. In the case of *r*_pbis_, the remaining non-missing responses were used for calculation if respondents had missing item responses. However, *r*_pbis_ at the second measurement could not be computed for 10 participants in Study 4 and for 5 participants in Study 5 (all of them were assigned to the fully careless responding condition), because their response protocols had no variance. The Stata command *mahascore* with the options *refmeans* and *compute_incov* was used for the computation of MD. Based on a transposed data frame, *r*_pbis_ could be computed with the *alpha* command in Stata and the item-rest correlation coefficients were extracted for further analyses purposes.

To check the utility of the subsample-based index calculations, we compared their performance in detecting careless respondents with the performance of distance/correlation indices that were calculated at once in the total sample. We therefore ran ROC analyses with the combined sample of Studies 4 and 5 (*n* = 962) in which the two careful responding groups were coded with 0 and the two careless responding groups with 1. In the case of MD, the subsample-based version (AUC = .85; *SE* = .01) turned out to perform significantly better (χ^2^[1] = 109.73, *p* < .001) than the version in which MD was calculated at once in the total sample (AUC = .77; *SE* = .02). In the case of the *r*_pbis_, however, the subsample-based version (AUC = .80; *SE* = .01) did not perform better (χ^2^[1] = 3.01, *p* = .08) than the version in which *r*_pbis_ was calculated at once in the total sample (AUC = .80; *SE* = .01). Thus, the additional effort for calculating the subsample-based versions of MD and *r*_pbis_ paid off in terms of higher detection accuracy, at least in the case of MD.

## Model-Based Inconsistency: Nonparametric Person-Fit Indices

### Guttman Error and Scalability Index

To obtain the normed Guttman error index for polytomous items (Gnormed; Emons, 2008; Molenaar, 1991) and the person or transposed scalability index (H_t_; Mokken; 1971; Sijtsma, 1986), we first computed for each of the five domains in BF1-2 domain-specific or scale-specific indices. On this basis, we then calculated a global or multi-test extension of Gnormed and H_t_ (Drasgow et al., 1991; Niessen et al., 2016, p. 6) by averaging the values of the scale-specific indices. The *PerFit* package (Tendeiro et al., 2016) was used to calculate Gnormed, and the *mokken* package (Van der Ark, 2007) was used to calculate H_t_. For calculating Gnormed, we used a data set in which rows represented observations and columns variables/items. For calculating H_t_, in contrast, we used a transposed data set in which rows represented variables/items and columns observations. Because Gnormed and H_t_ cannot be computed if missing item responses are present, we used the parametric and non-parametric imputation method that is available in *PerFit* before calculating Gnormed and H_t_ (see Niessen et al., 2016, p. 6) for the first and second measurement. In the 60 substantive items at the first measurement, 95.5% (*n* = 919) of the respondents completed the questionnaire with no missing responses, 4% (*n* = 38) with one missing response, and 0.5% (*n* = 5) with two missing responses. In the 60 substantive items at the second measurement 11.2% (*n* = 108) of the respondents had at least one missing item response; however, the vast majority of them (85%) had only one or two item responses missing. Given this pattern of missingness, we expected the effect of the imputation on the consistency of the response patterns to be minimal for the vast majority of the response protocols.

## Model-Based Inconsistency: Parametric IRT Based Person-Fit Indices

### Standardized Log-Likelihood and Infit and Outfit MSE

To obtain the standardized log-likelihood for polytomous items (lz; Drasgow et al., 1985), and the Rasch model-based infit and outfit mean square error (MSE) statistics (Wright & Stone, 1979), we first computed for each of the five domains in BF1-2 domain-specific or scale-specific indices. On this basis, we then calculated a global or multi-test extension of the lz and the infit and outfit MSE (Drasgow et al., 1991; Niessen et al., 2016, p. 6) by averaging the values of the scale-specific indices. We estimated graded response models (Samejima, 1969) in the *PerFit* package (Tendeiro et al., 2016) in the case of lz, and Rasch rating scale models (Andrich, 1978) in the *TAM* package (Robitzsch et al., 2020) in the case of infit and outfit MSE statistics. Because lz cannot be computed if missing item responses are present, we used the parametric and non-parametric imputation method that is available in *PerFit* before calculating lz (see Niessen et al., 2016, p. 6) for the first and second measurement. In the 60 substantive items at the first measurement, 95.5% (*n* = 919) of the respondents completed the questionnaire with no missing responses, 4% (*n* = 38) with one missing response and 0.5% (*n* = 5) with two missing responses. In the 60 substantive items at the second measurement 11.2% (*n* = 108) of the respondents had at least one missing item response; however, the vast majority of them (85%) had only one or two item responses missing. Given this pattern of missingness, we expected the effect of the imputation on the consistency of the response patterns to be minimal for the vast majority of the response protocols. When calculating infit and outfit MSE, in contrast, no imputation was necessary, because we used marginal maximum likelihood in the *TAM* package for estimating the Rasch rating scale models, which allowed us to handle missing values trough full information estimation.

## Model-Based Inconsistency: Parametric Covariance-Structure Based Person-Fit Index

### Individual Contribution to Model Misfit

As suggested by Goldammer et al. (2022), we used subsamples to calculate the individual contribution to model misfit (INDCHI; Reise & Widaman, 1999, pp. 7-8). We did so because the second part of formula for calculating the person-specific log-likelihood (i.e., the basis for calculating the INDCHI) is equivalent to the squared Mahalanobis distance (Reise & Widaman, 1999, p. 7), which in turn grounds on the assumption that the majority of responses was given carefully, to be a valid measure for aberrant responding (Curran, 2016, p. 13). Thus, calculating the INDCHI at once for the total sample (*n* = 962), could have produced a biased index in our case, because careless respondents formed 50% (*n* = 481) in the sample of Studies 4 and Study 5.

To obtain scale-specific INDCHI indices (for each of the five BFI-2 domains/scales) for each respondent, we first estimated a saturated model and a one-factor model for each scale and extracted the person-specific log-likelihood values of both models. In the subsample runs, we obtained for every careful respondent 481 person-specific log-likelihood values for the saturated model and 481 person-specific log-likelihood values for the one-factor model. For every careless respondent, one person-specific log-likelihood value for the saturated model and one person-specific log-likelihood value for the one-factor model were obtained from the subsample runs. The final two person-specific log-likelihood values for every careful respondent were obtained by averaging the 481 values of each of the two models. Next, the scale-specific INDCHI values could be computed for each respondent by subtracting the person-specific log-likelihood of the saturated model from the person-specific log-likelihood of the substantive factor model and by multiplying this difference by -2. Eventually, we computed a global or multi-test extension of the INDCHI by averaging the values of the scale-specific indices. All models were estimated with robust maximum likelihood estimation (MLR) in Mplus Version 8.8 (Muthén & Muthén, 1998-2017). Missing values were handled through the implemented default when using MLR (i.e., full information maximum likelihood estimation FIML).

To check the utility of the subsample-based index calculation, we compared its performance in detecting careless respondents with the performance of an INDCHI index that was calculated at once in the total sample. We therefore ran ROC analyses with the combined sample of Studies 4 and 5 (*n* = 962) in which the two careful responding groups were coded with 0 and the two careless responding groups with 1. Contrary to our expectation, however, the subsample-based version of INDCHI (AUC = .54; *SE* = .02) did not perform better (χ^2^[1] = 0.39, *p* = .53) than the version in which the INDCHI was calculated at once in the total sample (AUC = .55; *SE* = .02). Thus, the additional effort for calculating the subsample-based version of INDCHI did not pay off in terms of higher detection accuracy.

# Manipulation Check Study 4

To examine whether the respondents followed our response instructions, we compared the t1-t2 mean differences of the five substantive scales and of three indirect careless response indices (i.e., average response time per item, IRV, RPR) across the four response conditions (see Table S18). In line with our expectations, the t1-t2 mean differences were significantly different across the four response conditions for three of the five substantive scales (i.e., agreeableness, conscientiousness, and open-mindedness). The scores on the agreeableness, conscientiousness, and open-mindedness scales changed only marginally between t1 and t2 in the two careful responding conditions, but those in the two careless responding conditions became biased towards the scale mid-point. As anticipated, the bias towards the scale mid-point turned out to be more pronounced in the fully careless responding condition than in the partially careless responding condition. A similar pattern occurred when we examined the t1-t2 mean differences in average response time per item, IRV, and RPR across the four response conditions. Compared to the participants in the two careful responding conditions, participants in the fully careless responding condition increased their responding speed to a much larger degree and changed from consistent to inconsistent responding between t1 and t2.

In addition, we correlated the response vectors of the first and second measurement within each person and examined the within-person response vector correlation coefficients. The global test for equality indicated a significant inequality between the within-person response vector correlation coefficients of the four response conditions, χ^2^(3) = 832.28, *p* < .001. Bonferroni-corrected pairwise comparisons then revealed the expected pattern: First, the within-person response vector correlation coefficients of the unincentivized (*M*_1_ = .81, *SD*_1_ = .25) and incentivized careful responding group (*M*_2_ = .87, *SD*_2_ = .15) were high and did not differ from each other, *M*_2_-*M*_1_ = 0.05, *z* = 1.98, according to the Bonferroni-corrected critical *z-*value of 2.64. Second, the partially careless responding group (*M*_3_ = .56, *SD*_3_ = .23) and fully careless responding group (*M*_4_ = .03, *SD*_4_ = .29) had significantly lower within-person response vector correlation coefficients than the two careful responding groups (*M*_1_-*M*_3_ = 0.26, *z* = 8.18; *M*_1_-*M*_4_ = 0.78, *z* = 22.19; *M*_2_-*M*_3_ = 0.31, *z* = 12.11; *M*_2_-*M*_4_ = 0.83, *z* = 27.66). Third, the within-person response vector correlation coefficients of the fully careless responding group were significantly lower than those of the partially careless responding group (*M*_3_-*M*_4_ = 0.53, *z* = 15.28). Based on these results, the manipulation was successful, and we therefore proceeded with the main analyses.

# Table S18

*Comparisons of t1-t2 Mean Differences of Substantive Scale Scores and Selected Indirect Careless Responding Indices Across the Conditions in Study 4*

|  | Careful Unincentivized | | | | | |  | Careful Incentivized | | | | | |  | Careless 33% | | | | | |  | Careless 100% | | | | | |  |  |
| --- | --- | --- | --- | --- | --- | --- | --- | --- | --- | --- | --- | --- | --- | --- | --- | --- | --- | --- | --- | --- | --- | --- | --- | --- | --- | --- | --- | --- | --- |
|  | t1 | |  | t2 | |  |  | t1 | |  | t2 | |  |  | t1 | |  | t2 | |  |  | t1 | |  | t2 | |  |  |  |
| Measure | *M* | *SD* |  | *M* | *SD* | Δ*M* |  | *M* | *SD* |  | *M* | *SD* | Δ*M* |  | *M* | *SD* |  | *M* | *SD* | Δ*M* |  | *M* | *SD* |  | *M* | *SD* | Δ*M* |  | χ^2^(3) |
| Substantive Scale |  |  |  |  |  |  |  |  |  |  |  |  |  |  |  |  |  |  |  |  |  |  |  |  |  |  |  |  |  |
| Extraversion | 3.51 | 0.77 |  | 3.53 | 0.81 | 0.02 |  | 3.40 | 0.85 |  | 3.43 | 0.88 | 0.03 |  | 3.42 | 0.85 |  | 3.43 | 0.64 | 0.01 |  | 3.54 | 0.79 |  | 3.57 | 0.41 | 0.03 |  | 0.25 |
| Agreeableness | 4.40 | 0.70 |  | 4.41 | 0.78 | 0.01_a_ |  | 4.42 | 0.64 |  | 4.49 | 0.68 | 0.07_a_ |  | 4.51 | 0.63 |  | 4.14 | 0.69 | -0.37_b_ |  | 4.48 | 0.68 |  | 3.49 | 0.65 | -0.99_c_ |  | 181.18* |
| Conscientiousness | 4.31 | 0.79 |  | 4.34 | 0.80 | 0.03_a_ |  | 4.37 | 0.80 |  | 4.40 | 0.85 | 0.04_a_ |  | 4.39 | 0.83 |  | 4.11 | 0.67 | -0.28_b_ |  | 4.46 | 0.79 |  | 3.58 | 0.73 | -0.88_c_ |  | 107.30* |
| Emotionality | 3.36 | 1.03 |  | 3.28 | 1.04 | -0.09 |  | 3.37 | 1.08 |  | 3.29 | 1.12 | -0.07 |  | 3.39 | 0.91 |  | 3.36 | 0.76 | -0.04 |  | 3.43 | 0.80 |  | 3.43 | 0.51 | -0.01 |  | 1.72 |
| Open-mindedness | 4.21 | 0.79 |  | 4.17 | 0.86 | -0.04_a_ |  | 4.25 | 0.84 |  | 4.22 | 0.92 | -0.03_a_ |  | 4.34 | 0.83 |  | 4.05 | 0.77 | -0.30_b_ |  | 4.31 | 0.88 |  | 3.50 | 0.50 | -0.82_c_ |  | 88.87* |
| Careless Response Indices |  |  |  |  |  |  |  |  |  |  |  |  |  |  |  |  |  |  |  |  |  |  |  |  |  |  |  |  |  |
| Time | 5.88 | 3.54 |  | 4.82 | 2.53 | -1.06_a_ |  | 5.83 | 3.20 |  | 4.79 | 2.44 | -1.05_a_ |  | 5.71 | 3.37 |  | 4.33 | 2.61 | -1.38_a_ |  | 5.73 | 2.67 |  | 2.37 | 2.14 | -3.36_b_ |  | 63.77* |
| IRV | 1.36 | 0.31 |  | 1.36 | 0.34 | 0.00_a_ |  | 1.43 | 0.30 |  | 1.45 | 0.34 | 0.02_a_ |  | 1.42 | 0.33 |  | 1.44 | 0.34 | 0.02_a_ |  | 1.38 | 0.28 |  | 1.18 | 0.58 | -0.21_b_ |  | 16.38* |
| RPR | 0.75 | 0.29 |  | 0.76 | 0.36 | 0.01 |  | 0.82 | 0.14 |  | 0.84 | 0.18 | 0.02 |  | 0.77 | 0.22 |  | 0.42 | 0.41 | -0.35 |  | 0.79 | 0.23 |  | -0.18 | 0.56 | -0.96 |  | 401.26* |

*Note*. Means and standard deviations are based on univariate sample statistics for the unincentivized careful responding condition (*n* = 120), the incentivized careful responding condition (*n* = 121), the partially careless responding (i.e., Careless 33%) condition (*n* = 118), and the fully careless responding (i.e., Careless 100%) condition (*n* = 122); t1 = first measurement, t2 = second measurement; Δ*M* = estimated latent difference between t2 and t1 measurement; reported Delta estimates (i.e., ∆*M*) may not exactly match reported t1 and t2 values due to rounding error. In each row, ∆*M* values with different subscripts are significantly different from each other (i.e., α = .025/36, with the critical z-value of 3.20). χ^2^ = χ^2^ values were obtained by conducting Wald tests of parameter constraints that were based on robust full information maximum likelihood estimation (MLR) in Mplus Version 8.8.

^*^ Larger than the critical χ^2^(3) value of 12.36 (i.e., α = .05/8), which indicates global inequality between the ∆*M* values.

# Table S19

*Descriptive Statistics of the Substantive Measures and Careless Response Indices for the Unincentivized Careful Responding Group in Study 4 at t1*

| Scale | 1. | 2. | 3. | 4. | 5. | 6. | 7. | 8. | 9. | 10. | 11. | 12. | 13. | 14. | 15. | 16. | 17. | 18. | 19. | 20. |
| --- | --- | --- | --- | --- | --- | --- | --- | --- | --- | --- | --- | --- | --- | --- | --- | --- | --- | --- | --- | --- |
| 1. EX | 1 |  |  |  |  |  |  |  |  |  |  |  |  |  |  |  |  |  |  |  |
| 2. AG | .363** | 1 |  |  |  |  |  |  |  |  |  |  |  |  |  |  |  |  |  |  |
| 3. CO | .219* | .409** | 1 |  |  |  |  |  |  |  |  |  |  |  |  |  |  |  |  |  |
| 4. EM | -.368** | -.295** | -.445** | 1 |  |  |  |  |  |  |  |  |  |  |  |  |  |  |  |  |
| 5. OP | .227* | .078 | -.036 | -.023 | 1 |  |  |  |  |  |  |  |  |  |  |  |  |  |  |  |
| 6. Bogus | .019 | -.123 | -.071 | -.010 | -.058 | 1 |  |  |  |  |  |  |  |  |  |  |  |  |  |  |
| 7. Time | .178 | .051 | .086 | -.171 | .107 | .512** | 1 |  |  |  |  |  |  |  |  |  |  |  |  |  |
| 8. Longstring | -.087 | -.256** | -.195* | .157 | -.148 | .805** | .349** | 1 |  |  |  |  |  |  |  |  |  |  |  |  |
| 9. IRV | .296** | .507** | .382** | -.305** | .217* | -.327** | -.014 | -.540** | 1 |  |  |  |  |  |  |  |  |  |  |  |
| 10. Synonyms | .156 | .454** | .473** | -.183* | -.037 | -.459** | -.168 | -.582** | .563** | 1 |  |  |  |  |  |  |  |  |  |  |
| 11. Antonyms | -.056 | -.236** | -.241** | .091 | .114 | .481** | .297** | .502** | -.416** | -.704** | 1 |  |  |  |  |  |  |  |  |  |
| 12. RPR | -.073 | .275** | .236** | -.197* | .053 | -.664** | -.262** | -.771** | .492** | .630** | -.537** | 1 |  |  |  |  |  |  |  |  |
| 13. MD | .051 | -.033 | -.194* | .010 | -.020 | .031 | .045 | -.079 | .457** | -.183* | .155 | -.083 | 1 |  |  |  |  |  |  |  |
| 14. *r*_pbis_ | .166 | .621** | .619** | -.271** | .455** | -.199* | .016 | -.309** | .226* | .355** | -.105 | .303** | -.357** | 1 |  |  |  |  |  |  |
| 15. Gnormed | .208* | .215* | .051 | -.165 | .053 | .074 | .181* | -.079 | .683** | .028 | -.010 | -.023 | .785** | -.200* | 1 |  |  |  |  |  |
| 16. H_t_ | -.237** | -.022 | .019 | .171 | -.030 | -.505** | -.383** | -.429** | .067 | .205* | -.247** | .337** | -.155 | .260** | -.240** | 1 |  |  |  |  |
| 17. lz | -.097 | -.069 | .086 | .033 | -.037 | -.221* | -.205* | -.081 | -.501** | .184* | -.183* | .171 | -.866** | .293** | -.902** | .305** | 1 |  |  |  |
| 18. Infit MSE | .202* | .217* | .044 | -.134 | .122 | .059 | .205* | -.108 | .682** | .005 | .031 | -.021 | .847** | -.139 | .944** | -.218* | -.929** | 1 |  |  |
| 19. Outfit MSE | .183* | .199* | .022 | -.120 | .094 | .078 | .203* | -.095 | .661** | -.006 | .045 | -.036 | .852** | -.167 | .945** | -.229* | -.940** | .992** | 1 |  |
| 20. INDCHI | -.147 | .014 | -.063 | .138 | .096 | .206* | .181* | .173 | -.259** | -.295** | .422** | -.221* | .042 | .191* | .022 | -.042 | -.222* | .130 | .163 | 1 |
| *M* | 3.514 | 4.402 | 4.313 | 3.366 | 4.207 | 1.097 | 5.875 | 3.500 | 1.363 | 0.652 | -0.639 | 0.748 | 56.731 | 0.519 | 0.108 | 0.181 | 0.284 | 0.883 | 0.875 | -0.829 |
| *SD* | 0.769 | 0.704 | 0.792 | 1.028 | 0.794 | 0.310 | 3.542 | 1.815 | 0.307 | 0.200 | 0.221 | 0.286 | 20.795 | 0.209 | 0.055 | 0.086 | 0.816 | 0.370 | 0.353 | 2.097 |

*Note.* The careful responding group included 120 participants. All reported Pearson correlation coefficients and means and standard deviations are based on 120 observations. EX = Extraversion; AG = Agreeableness; CO = Conscientiousness; EM = Negative Emotionality; OP = Open-Mindedness; bogus = average score of the eight bogus items; time = average response time per item; longstring = maximum longstring; IRV = intra-individual response variability; synonyms = psychometric synonyms; antonyms = psychometric antonyms; RPR = resampled personal reliability; MD = Mahalanobis distance measure for which the calculation was based on subsamples; *r*_pbis_ = person-total correlation coefficient for which the calculation was based on subsamples; Gnormed = global (i.e., the average of the scale-specific scores was computed) normed Guttman error index for polytomous items; H_t_ = global (i.e., the average of the scale-specific scores was computed) person scalability index; lz = global (i.e., the average of the scale-specific scores was computed) standardized log-likelihood index for polytomous items; infit MSE = global (i.e., the average of the scale-specific statistics was computed) Rasch model-based infit mean square error; outfit MSE = global (i.e., the average of the scale-specific statistics was computed) Rasch model-based outfit mean square error; INDCHI = global (i.e., the average of the scale-specific scores was computed) individual contribution to model misfit for which the calculation of the two person-specific model log-likelihood values was based on subsamples.

* *p* < .05 (two-tailed).

** *p* < .01 (two-tailed).

# Table S20

*Descriptive Statistics of the Substantive Measures and Careless Response Indices for the Incentivized Careful Responding Group in Study 4 at t1*

| Scale | 1. | 2. | 3. | 4. | 5. | 6. | 7. | 8. | 9. | 10. | 11. | 12. | 13. | 14. | 15. | 16. | 17. | 18. | 19. | 20. |
| --- | --- | --- | --- | --- | --- | --- | --- | --- | --- | --- | --- | --- | --- | --- | --- | --- | --- | --- | --- | --- |
| 1. EX | 1 |  |  |  |  |  |  |  |  |  |  |  |  |  |  |  |  |  |  |  |
| 2. AG | .173 | 1 |  |  |  |  |  |  |  |  |  |  |  |  |  |  |  |  |  |  |
| 3. CO | .343** | .369** | 1 |  |  |  |  |  |  |  |  |  |  |  |  |  |  |  |  |  |
| 4. EM | -.488** | -.329** | -.568** | 1 |  |  |  |  |  |  |  |  |  |  |  |  |  |  |  |  |
| 5. OP | .247** | .357** | .093 | -.178 | 1 |  |  |  |  |  |  |  |  |  |  |  |  |  |  |  |
| 6. Bogus | .062 | -.224* | -.210* | .105 | -.051 | 1 |  |  |  |  |  |  |  |  |  |  |  |  |  |  |
| 7. Time | .065 | .105 | .039 | .034 | .043 | .000 | 1 |  |  |  |  |  |  |  |  |  |  |  |  |  |
| 8. Longstring | -.063 | -.212* | -.147 | .088 | -.132 | .076 | -.037 | 1 |  |  |  |  |  |  |  |  |  |  |  |  |
| 9. IRV | .013 | .277** | .363** | -.137 | .090 | -.021 | -.011 | -.456** | 1 |  |  |  |  |  |  |  |  |  |  |  |
| 10. Synonyms | .138 | .372** | .401** | -.202* | .124 | -.179* | .094 | -.191* | .338** | 1 |  |  |  |  |  |  |  |  |  |  |
| 11. Antonyms | .052 | .058 | -.265** | .068 | .185* | .092 | .038 | .067 | -.232* | -.526** | 1 |  |  |  |  |  |  |  |  |  |
| 12. RPR | -.060 | .312** | .312** | -.247** | .113 | -.183* | .108 | -.226* | .337** | .704** | -.541** | 1 |  |  |  |  |  |  |  |  |
| 13. MD | -.156 | -.257** | -.121 | .147 | -.274** | .221* | -.091 | -.191* | .556** | -.397** | .147 | -.230* | 1 |  |  |  |  |  |  |  |
| 14. *r*_pbis_ | .225* | .712** | .598** | -.461** | .632** | -.261** | .096 | -.049 | -.006 | .282** | .064 | .259** | -.501** | 1 |  |  |  |  |  |  |
| 15. Gnormed | -.102 | -.191* | -.011 | .075 | -.231* | .310** | -.062 | -.203* | .680** | -.183* | -.004 | -.076 | .894** | -.437** | 1 |  |  |  |  |  |
| 16. H_t_ | -.271** | .206* | .006 | .133 | .040 | -.158 | .029 | .033 | .055 | .114 | .102 | .041 | -.209* | .228* | -.199* | 1 |  |  |  |  |
| 17. lz | .145 | .175 | .052 | -.123 | .202* | -.208* | .052 | .212* | -.635** | .289** | -.077 | .138 | -.923** | .410** | -.954** | .153 | 1 |  |  |  |
| 18. Infit MSE | -.075 | -.131 | .043 | .055 | -.188* | .235** | -.075 | -.215* | .708** | -.189* | .037 | -.081 | .906** | -.374** | .981** | -.186* | -.958** | 1 |  |  |
| 19. Outfit MSE | -.071 | -.114 | .055 | .032 | -.190* | .171 | -.083 | -.226* | .707** | -.201* | .040 | -.081 | .912** | -.359** | .967** | -.203* | -.962** | .993** | 1 |  |
| 20. INDCHI | -.078 | .043 | -.016 | .088 | -.027 | -.038 | -.013 | .075 | .011 | -.368** | .263** | -.175 | .341** | .069 | .363** | .013 | -.487** | .413** | .440** | 1 |
| *M* | 3.396 | 4.419 | 4.368 | 3.365 | 4.249 | 1.127 | 5.834 | 3.110 | 1.432 | 0.692 | -0.660 | 0.816 | 59.396 | 0.540 | 0.117 | 0.196 | 0.143 | 0.962 | 0.943 | -0.362 |
| *SD* | 0.847 | 0.641 | 0.803 | 1.076 | 0.844 | 0.396 | 3.203 | 1.031 | 0.302 | 0.161 | 0.206 | 0.140 | 25.313 | 0.220 | 0.071 | 0.068 | 1.080 | 0.503 | 0.464 | 2.363 |

*Note.* The careful responding group included 121 participants. All reported Pearson correlation coefficients and means and standard deviations are based on 121 observations. EX = Extraversion; AG = Agreeableness; CO = Conscientiousness; EM = Negative Emotionality; OP = Open-Mindedness; bogus = average score of the eight bogus items; time = average response time per item; longstring = maximum longstring; IRV = intra-individual response variability; synonyms = psychometric synonyms; antonyms = psychometric antonyms; RPR = resampled personal reliability; MD = Mahalanobis distance measure for which the calculation was based on subsamples; *r*_pbis_ = person-total correlation coefficient for which the calculation was based on subsamples; Gnormed = global (i.e., the average of the scale-specific scores was computed) normed Guttman error index for polytomous items; H_t_ = global (i.e., the average of the scale-specific scores was computed) person scalability index; lz = global (i.e., the average of the scale-specific scores was computed) standardized log-likelihood index for polytomous items; infit MSE = global (i.e., the average of the scale-specific statistics was computed) Rasch model-based infit mean square error; outfit MSE = global (i.e., the average of the scale-specific statistics was computed) Rasch model-based outfit mean square error; INDCHI = global (i.e., the average of the scale-specific scores was computed) individual contribution to model misfit for which the calculation of the two person-specific model log-likelihood values was based on subsamples.

* *p* < .05 (two-tailed).

** *p* < .01 (two-tailed).

# Table S21

*Descriptive Statistics of the Substantive Measures and Careless Response Indices for the Partially Careless Responding Group (i.e., Careless 33%) in Study 4 at t1*

| Scale | 1. | 2. | 3. | 4. | 5. | 6. | 7. | 8. | 9. | 10. | 11. | 12. | 13. | 14. | 15. | 16. | 17. | 18. | 19. | 20. |
| --- | --- | --- | --- | --- | --- | --- | --- | --- | --- | --- | --- | --- | --- | --- | --- | --- | --- | --- | --- | --- |
| 1. EX | 1 |  |  |  |  |  |  |  |  |  |  |  |  |  |  |  |  |  |  |  |
| 2. AG | .214* | 1 |  |  |  |  |  |  |  |  |  |  |  |  |  |  |  |  |  |  |
| 3. CO | .335** | .308** | 1 |  |  |  |  |  |  |  |  |  |  |  |  |  |  |  |  |  |
| 4. EM | -.406** | -.293** | -.499** | 1 |  |  |  |  |  |  |  |  |  |  |  |  |  |  |  |  |
| 5. OP | .378** | .214* | .079 | .010 | 1 |  |  |  |  |  |  |  |  |  |  |  |  |  |  |  |
| 6. Bogus | .186* | .012 | .154 | -.193* | -.097 | 1 |  |  |  |  |  |  |  |  |  |  |  |  |  |  |
| 7. Time | -.006 | .076 | .138 | -.054 | -.054 | .228* | 1 |  |  |  |  |  |  |  |  |  |  |  |  |  |
| 8. Longstring | .023 | -.170 | -.020 | -.024 | -.112 | .265** | .195* | 1 |  |  |  |  |  |  |  |  |  |  |  |  |
| 9. IRV | .119 | .358** | .346** | .012 | .362** | -.069 | .092 | -.317** | 1 |  |  |  |  |  |  |  |  |  |  |  |
| 10. Synonyms | .030 | .369** | .217* | -.182* | .268** | -.158 | -.060 | -.342** | .382** | 1 |  |  |  |  |  |  |  |  |  |  |
| 11. Antonyms | .069 | -.050 | -.093 | .066 | -.173 | .199* | .102 | .261** | -.248** | -.605** | 1 |  |  |  |  |  |  |  |  |  |
| 12. RPR | -.159 | .330** | .209* | -.148 | .149 | -.432** | -.040 | -.365** | .359** | .672** | -.561** | 1 |  |  |  |  |  |  |  |  |
| 13. MD | .008 | -.070 | .103 | .184* | -.074 | .116 | .169 | .056 | .545** | -.359** | .296** | -.291** | 1 |  |  |  |  |  |  |  |
| 14. *r*_pbis_ | .280** | .582** | .584** | -.386** | .484** | -.102 | .001 | -.039 | .068 | .317** | -.144 | .397** | -.390** | 1 |  |  |  |  |  |  |
| 15. Gnormed | .130 | .090 | .211* | .030 | .089 | .130 | .242** | .011 | .723** | -.073 | .065 | -.131 | .841** | -.248** | 1 |  |  |  |  |  |
| 16. H_t_ | -.252** | -.115 | -.212* | .151 | .021 | -.346** | -.092 | -.126 | -.069 | .140 | -.201* | .298** | -.396** | .128 | -.385** | 1 |  |  |  |  |
| 17. lz | -.081 | .023 | -.167 | -.136 | -.043 | -.158 | -.174 | -.026 | -.645** | .202* | -.155 | .238** | -.888** | .309** | -.937** | .407** | 1 |  |  |  |
| 18. Infit MSE | .156 | .093 | .250** | .021 | .106 | .115 | .260** | .048 | .713** | -.101 | .106 | -.145 | .864** | -.209* | .980** | -.385** | -.943** | 1 |  |  |
| 19. Outfit MSE | .120 | .058 | .242** | .059 | .085 | .131 | .231* | .027 | .697** | -.131 | .104 | -.170 | .883** | -.231* | .970** | -.411** | -.967** | .987** | 1 |  |
| 20. INDCHI | -.076 | -.255** | .007 | .165 | -.041 | .006 | -.119 | .214* | -.220* | -.308** | .187* | -.254** | .128 | .048 | .070 | -.153 | -.293** | .123 | .193* | 1 |
| *M* | 3.421 | 4.507 | 4.386 | 3.392 | 4.345 | 1.118 | 5.714 | 3.380 | 1.420 | 0.643 | -0.602 | 0.774 | 60.105 | 0.574 | 0.117 | 0.190 | 0.176 | 0.970 | 0.941 | -0.633 |
| *SD* | 0.852 | 0.626 | 0.835 | 0.907 | 0.833 | 0.280 | 3.366 | 1.161 | 0.333 | 0.163 | 0.212 | 0.217 | 24.632 | 0.194 | 0.074 | 0.076 | 1.042 | 0.544 | 0.477 | 2.173 |

*Note.* The careful responding group included 118 participants. All reported Pearson correlation coefficients and means and standard deviations are based on 118 observations. EX = Extraversion; AG = Agreeableness; CO = Conscientiousness; EM = Negative Emotionality; OP = Open-Mindedness; bogus = average score of the eight bogus items; time = average response time per item; longstring = maximum longstring; IRV = intra-individual response variability; synonyms = psychometric synonyms; antonyms = psychometric antonyms; RPR = resampled personal reliability; MD = Mahalanobis distance measure for which the calculation was based on subsamples; *r*_pbis_ = person-total correlation coefficient for which the calculation was based on subsamples; Gnormed = global (i.e., the average of the scale-specific scores was computed) normed Guttman error index for polytomous items; H_t_ = global (i.e., the average of the scale-specific scores was computed) person scalability index; lz = global (i.e., the average of the scale-specific scores was computed) standardized log-likelihood index for polytomous items; infit MSE = global (i.e., the average of the scale-specific statistics was computed) Rasch model-based infit mean square error; outfit MSE = global (i.e., the average of the scale-specific statistics was computed) Rasch model-based outfit mean square error; INDCHI = global (i.e., the average of the scale-specific scores was computed) individual contribution to model misfit for which the calculation of the two person-specific model log-likelihood values was based on subsamples.

* *p* < .05 (two-tailed).

** *p* < .01 (two-tailed).

# Table S22

*Descriptive Statistics of the Substantive Measures and Careless Response Indices for the Fully Careless Responding Group (i.e., Careless 100%) in Study 4 at t1*

| Scale | 1. | 2. | 3. | 4. | 5. | 6. | 7. | 8. | 9. | 10. | 11. | 12. | 13. | 14. | 15. | 16. | 17. | 18. | 19. | 20. |
| --- | --- | --- | --- | --- | --- | --- | --- | --- | --- | --- | --- | --- | --- | --- | --- | --- | --- | --- | --- | --- |
| 1. EX | 1 |  |  |  |  |  |  |  |  |  |  |  |  |  |  |  |  |  |  |  |
| 2. AG | .119 | 1 |  |  |  |  |  |  |  |  |  |  |  |  |  |  |  |  |  |  |
| 3. CO | .328** | .267** | 1 |  |  |  |  |  |  |  |  |  |  |  |  |  |  |  |  |  |
| 4. EM | -.467** | -.356** | -.442** | 1 |  |  |  |  |  |  |  |  |  |  |  |  |  |  |  |  |
| 5. OP | .535** | .277** | .221* | -.250** | 1 |  |  |  |  |  |  |  |  |  |  |  |  |  |  |  |
| 6. Bogus | .086 | -.033 | -.028 | -.128 | -.010 | 1 |  |  |  |  |  |  |  |  |  |  |  |  |  |  |
| 7. Time | .044 | -.097 | .026 | .030 | -.141 | .228* | 1 |  |  |  |  |  |  |  |  |  |  |  |  |  |
| 8. Longstring | -.122 | -.307** | -.127 | .129 | -.191* | .158 | .165 | 1 |  |  |  |  |  |  |  |  |  |  |  |  |
| 9. IRV | .091 | .415** | .343** | -.126 | .312** | -.129 | .007 | -.417** | 1 |  |  |  |  |  |  |  |  |  |  |  |
| 10. Synonyms | .018 | .290** | .352** | -.257** | .125 | -.320** | -.298** | -.256** | .341** | 1 |  |  |  |  |  |  |  |  |  |  |
| 11. Antonyms | -.003 | -.150 | -.230* | .080 | -.013 | .380** | .152 | .177 | -.191* | -.641** | 1 |  |  |  |  |  |  |  |  |  |
| 12. RPR | -.106 | .213* | .239** | -.253** | .028 | -.362** | -.304** | -.286** | .262** | .700** | -.550** | 1 |  |  |  |  |  |  |  |  |
| 13. MD | -.129 | -.107 | -.245** | .206* | -.145 | .166 | .240** | .038 | .409** | -.477** | .492** | -.428** | 1 |  |  |  |  |  |  |  |
| 14. *r*_pbis_ | .374** | .633** | .661** | -.452** | .609** | -.033 | -.106 | -.142 | .162 | .298** | -.157 | .222* | -.440** | 1 |  |  |  |  |  |  |
| 15. Gnormed | -.015 | .047 | -.030 | .044 | .067 | .011 | .198* | -.026 | .618** | -.186* | .269** | -.187* | .812** | -.226* | 1 |  |  |  |  |  |
| 16. H_t_ | -.291** | .129 | -.020 | .200* | -.117 | -.219* | .011 | -.022 | -.046 | .013 | -.108 | .001 | -.180* | .170 | -.237** | 1 |  |  |  |  |
| 17. lz | .047 | -.029 | .164 | -.090 | .028 | -.046 | -.198* | -.059 | -.535** | .302** | -.375** | .288** | -.896** | .320** | -.922** | .191* | 1 |  |  |  |
| 18. Infit MSE | .026 | .114 | -.050 | .036 | .027 | .022 | .188* | -.019 | .620** | -.240** | .291** | -.260** | .873** | -.217* | .921** | -.193* | -.959** | 1 |  |  |
| 19. Outfit MSE | .029 | .089 | -.063 | .041 | .035 | .025 | .190* | -.016 | .613** | -.249** | .305** | -.265** | .880** | -.231* | .931** | -.204* | -.964** | .997** | 1 |  |
| 20. INDCHI | .161 | .138 | -.012 | -.105 | .127 | -.011 | .072 | .266** | -.115 | -.237** | .295** | -.128 | .220* | .220* | .341** | -.103 | -.402** | .365** | .364** | 1 |
| *M* | 3.536 | 4.480 | 4.459 | 3.435 | 4.312 | 1.097 | 5.728 | 3.250 | 1.384 | 0.665 | -0.633 | 0.779 | 53.234 | 0.575 | 0.103 | 0.182 | 0.387 | 0.846 | 0.838 | -0.777 |
| *SD* | 0.784 | 0.683 | 0.789 | 0.804 | 0.876 | 0.264 | 2.668 | 0.956 | 0.280 | 0.173 | 0.220 | 0.228 | 22.331 | 0.229 | 0.060 | 0.068 | 0.942 | 0.415 | 0.402 | 2.621 |

*Note.* The careful responding group included 122 participants. All reported Pearson correlation coefficients and means and standard deviations are based on 122 observations. EX = Extraversion; AG = Agreeableness; CO = Conscientiousness; EM = Negative Emotionality; OP = Open-Mindedness; bogus = average score of the eight bogus items; time = average response time per item; longstring = maximum longstring; IRV = intra-individual response variability; synonyms = psychometric synonyms; antonyms = psychometric antonyms; RPR = resampled personal reliability; MD = Mahalanobis distance measure for which the calculation was based on subsamples; *r*_pbis_ = person-total correlation coefficient for which the calculation was based on subsamples; Gnormed = global (i.e., the average of the scale-specific scores was computed) normed Guttman error index for polytomous items; H_t_ = global (i.e., the average of the scale-specific scores was computed) person scalability index; lz = global (i.e., the average of the scale-specific scores was computed) standardized log-likelihood index for polytomous items; infit MSE = global (i.e., the average of the scale-specific statistics was computed) Rasch model-based infit mean square error; outfit MSE = global (i.e., the average of the scale-specific statistics was computed) Rasch model-based outfit mean square error; INDCHI = global (i.e., the average of the scale-specific scores was computed) individual contribution to model misfit for which the calculation of the two person-specific model log-likelihood values was based on subsamples.

* *p* < .05 (two-tailed).

** *p* < .01 (two-tailed).

# Table S23

*Descriptive Statistics of the Substantive Measures and Careless Response Indices for the Unincentivized Careful Responding Group in Study 4 at t2*

| Scale | 1. | 2. | 3. | 4. | 5. | 6. | 7. | 8. | 9. | 10. | 11. | 12. | 13. | 14. | 15. | 16. | 17. | 18. | 19. | 20. |
| --- | --- | --- | --- | --- | --- | --- | --- | --- | --- | --- | --- | --- | --- | --- | --- | --- | --- | --- | --- | --- |
| 1. EX | 1 |  |  |  |  |  |  |  |  |  |  |  |  |  |  |  |  |  |  |  |
| 2. AG | .243** | 1 |  |  |  |  |  |  |  |  |  |  |  |  |  |  |  |  |  |  |
| 3. CO | .196* | .447** | 1 |  |  |  |  |  |  |  |  |  |  |  |  |  |  |  |  |  |
| 4. EM | -.420** | -.264** | -.422** | 1 |  |  |  |  |  |  |  |  |  |  |  |  |  |  |  |  |
| 5. OP | .218* | .046 | .002 | -.068 | 1 |  |  |  |  |  |  |  |  |  |  |  |  |  |  |  |
| 6. Bogus | .089 | -.377** | -.333** | -.013 | -.069 | 1 |  |  |  |  |  |  |  |  |  |  |  |  |  |  |
| 7. Time | .148 | .079 | -.040 | -.056 | .147 | -.122 | 1 |  |  |  |  |  |  |  |  |  |  |  |  |  |
| 8. Longstring | .011 | -.338** | -.222* | .050 | -.106 | .467** | .058 | 1 |  |  |  |  |  |  |  |  |  |  |  |  |
| 9. IRV | .379** | .393** | .393** | -.339** | .281** | -.066 | -.013 | -.321** | 1 |  |  |  |  |  |  |  |  |  |  |  |
| 10. Synonyms | .189* | .408** | .529** | -.214* | .123 | -.634** | .096 | -.350** | .500** | 1 |  |  |  |  |  |  |  |  |  |  |
| 11. Antonyms | -.142 | -.283** | -.410** | .150 | -.063 | .542** | .001 | .314** | -.494** | -.836** | 1 |  |  |  |  |  |  |  |  |  |
| 12. RPR | .024 | .308** | .366** | -.152 | .181* | -.581** | .054 | -.522** | .500** | .771** | -.785** | 1 |  |  |  |  |  |  |  |  |
| 13. MD | .080 | -.315** | -.331** | -.004 | -.046 | .723** | -.153 | .151 | .303** | -.495** | .376** | -.353** | 1 |  |  |  |  |  |  |  |
| 14. *r*_pbis_ | .092 | .676** | .628** | -.284** | .443** | -.520** | .078 | -.389** | .242** | .531** | -.371** | .505** | -.533** | 1 |  |  |  |  |  |  |
| 15. Gnormed | .233* | -.218* | -.147 | -.114 | .066 | .700** | -.045 | .297** | .449** | -.240** | .222* | -.276** | .843** | -.399** | 1 |  |  |  |  |  |
| 16. H_t_ | -.044 | .200* | .260** | -.076 | .113 | -.473** | -.122 | -.407** | .018 | .276** | -.243** | .360** | -.391** | .460** | -.427** | 1 |  |  |  |  |
| 17. lz | -.147 | .140 | .182* | .028 | .052 | -.667** | .126 | -.267** | -.299** | .462** | -.416** | .489** | -.825** | .466** | -.842** | .424** | 1 |  |  |  |
| 18. Infit MSE | .259** | -.161 | -.106 | -.138 | .111 | .661** | -.033 | .262** | .499** | -.188* | .187* | -.223* | .821** | -.326** | .991** | -.383** | -.818** | 1 |  |  |
| 19. Outfit MSE | .213* | -.136 | -.123 | -.098 | .065 | .655** | -.078 | .229* | .468** | -.252** | .252** | -.286** | .844** | -.348** | .969** | -.394** | -.899** | .973** | 1 |  |
| 20. INDCHI | -.284** | .003 | -.103 | .227* | -.090 | .067 | -.058 | .123 | -.400** | -.353** | .480** | -.435** | .017 | -.028 | .010 | .054 | -.311** | -.011 | .118 | 1 |
| *M* | 3.533 | 4.414 | 4.342 | 3.275 | 4.168 | 1.219 | 4.815 | 3.500 | 1.359 | 0.686 | -0.672 | 0.758 | 57.209 | 0.504 | 0.118 | 0.152 | 1.001 | 0.635 | 0.615 | -0.590 |
| *SD* | 0.811 | 0.785 | 0.799 | 1.044 | 0.864 | 0.767 | 2.528 | 2.359 | 0.339 | 0.242 | 0.250 | 0.360 | 30.620 | 0.247 | 0.100 | 0.092 | 0.855 | 0.472 | 0.387 | 2.883 |

*Note.* The careful responding group included 120 participants. All reported Pearson correlation coefficients and means and standard deviations are based on 120 observations. EX = Extraversion; AG = Agreeableness; CO = Conscientiousness; EM = Negative Emotionality; OP = Open-Mindedness; bogus = average score of the eight bogus items; time = average response time per item; longstring = maximum longstring; IRV = intra-individual response variability; synonyms = psychometric synonyms; antonyms = psychometric antonyms; RPR = resampled personal reliability; MD = Mahalanobis distance measure for which the calculation was based on subsamples; *r*_pbis_ = person-total correlation coefficient for which the calculation was based on subsamples; Gnormed = global (i.e., the average of the scale-specific scores was computed) normed Guttman error index for polytomous items; H_t_ = global (i.e., the average of the scale-specific scores was computed) person scalability index; lz = global (i.e., the average of the scale-specific scores was computed) standardized log-likelihood index for polytomous items; infit MSE = global (i.e., the average of the scale-specific statistics was computed) Rasch model-based infit mean square error; outfit MSE = global (i.e., the average of the scale-specific statistics was computed) Rasch model-based outfit mean square error; INDCHI = global (i.e., the average of the scale-specific scores was computed) individual contribution to model misfit for which the calculation of the two person-specific model log-likelihood values was based on subsamples.

* *p* < .05 (two-tailed).

** *p* < .01 (two-tailed).

# Table S24

*Descriptive Statistics of the Substantive Measures and Careless Response Indices for the Incentivized Careful Responding Group in Study 4 at t2*

| Scale | 1. | 2. | 3. | 4. | 5. | 6. | 7. | 8. | 9. | 10. | 11. | 12. | 13. | 14. | 15. | 16. | 17. | 18. | 19. | 20. |
| --- | --- | --- | --- | --- | --- | --- | --- | --- | --- | --- | --- | --- | --- | --- | --- | --- | --- | --- | --- | --- |
| 1. EX | 1 |  |  |  |  |  |  |  |  |  |  |  |  |  |  |  |  |  |  |  |
| 2. AG | .266** | 1 |  |  |  |  |  |  |  |  |  |  |  |  |  |  |  |  |  |  |
| 3. CO | .343** | .449** | 1 |  |  |  |  |  |  |  |  |  |  |  |  |  |  |  |  |  |
| 4. EM | -.515** | -.349** | -.529** | 1 |  |  |  |  |  |  |  |  |  |  |  |  |  |  |  |  |
| 5. OP | .274** | .319** | .143 | -.188* | 1 |  |  |  |  |  |  |  |  |  |  |  |  |  |  |  |
| 6. Bogus | .033 | -.098 | -.091 | -.039 | -.066 | 1 |  |  |  |  |  |  |  |  |  |  |  |  |  |  |
| 7. Time | -.044 | -.137 | -.263** | .026 | -.170 | -.054 | 1 |  |  |  |  |  |  |  |  |  |  |  |  |  |
| 8. Longstring | -.182* | -.318** | -.102 | .201* | -.239** | -.067 | -.065 | 1 |  |  |  |  |  |  |  |  |  |  |  |  |
| 9. IRV | .128 | .366** | .339** | -.209* | .162 | -.040 | .114 | -.475** | 1 |  |  |  |  |  |  |  |  |  |  |  |
| 10. Synonyms | .145 | .376** | .404** | -.175 | .188* | -.397** | -.067 | -.205* | .179* | 1 |  |  |  |  |  |  |  |  |  |  |
| 11. Antonyms | .010 | -.147 | -.312** | .058 | -.034 | .402** | -.037 | .171 | -.164 | -.732** | 1 |  |  |  |  |  |  |  |  |  |
| 12. RPR | .033 | .284** | .254** | -.190* | .169 | -.595** | .028 | -.090 | .124 | .704** | -.737** | 1 |  |  |  |  |  |  |  |  |
| 13. MD | -.096 | -.177 | -.209* | .111 | -.195* | .281** | .279** | -.195* | .557** | -.556** | .415** | -.492** | 1 |  |  |  |  |  |  |  |
| 14. *r*_pbis_ | .252** | .684** | .679** | -.468** | .596** | -.174 | -.275** | -.111 | .044 | .415** | -.237** | .365** | -.490** | 1 |  |  |  |  |  |  |
| 15. Gnormed | -.097 | -.105 | -.044 | .027 | -.159 | .115 | .245** | -.161 | .695** | -.369** | .247** | -.274** | .915** | -.377** | 1 |  |  |  |  |  |
| 16. H_t_ | .002 | .182* | .412** | -.121 | .013 | -.155 | -.155 | .078 | -.086 | .161 | -.203* | .187* | -.368** | .441** | -.328** | 1 |  |  |  |  |
| 17. lz | .076 | .083 | .077 | -.049 | .151 | -.165 | -.254** | .231* | -.676** | .420** | -.263** | .321** | -.932** | .393** | -.969** | .350** | 1 |  |  |  |
| 18. Infit MSE | -.044 | .001 | .046 | -.016 | -.103 | .089 | .204* | -.188* | .754** | -.310** | .205* | -.226* | .883** | -.281** | .983** | -.261** | -.956** | 1 |  |  |
| 19. Outfit MSE | -.041 | .000 | .029 | -.031 | -.105 | .106 | .210* | -.220* | .753** | -.326** | .222* | -.245** | .899** | -.296** | .984** | -.301** | -.967** | .995** | 1 |  |
| 20. INDCHI | -.178 | -.095 | -.143 | .082 | -.074 | -.014 | .152 | -.011 | .002 | -.406** | .227* | -.169 | .400** | -.063 | .427** | -.052 | -.476** | .410** | .426** | 1 |
| *M* | 3.426 | 4.491 | 4.404 | 3.292 | 4.219 | 1.119 | 4.786 | 3.030 | 1.447 | 0.741 | -0.698 | 0.839 | 57.900 | 0.534 | 0.125 | 0.178 | 0.930 | 0.700 | 0.680 | -0.388 |
| *SD* | 0.883 | 0.675 | 0.855 | 1.120 | 0.921 | 0.427 | 2.441 | 0.894 | 0.341 | 0.153 | 0.225 | 0.178 | 32.473 | 0.249 | 0.091 | 0.071 | 0.957 | 0.464 | 0.430 | 2.703 |

*Note.* The careful responding group included 121 participants. All reported Pearson correlation coefficients and means and standard deviations are based on 121 observations. EX = Extraversion; AG = Agreeableness; CO = Conscientiousness; EM = Negative Emotionality; OP = Open-Mindedness; bogus = average score of the eight bogus items; time = average response time per item; longstring = maximum longstring; IRV = intra-individual response variability; synonyms = psychometric synonyms; antonyms = psychometric antonyms; RPR = resampled personal reliability; MD = Mahalanobis distance measure for which the calculation was based on subsamples; *r*_pbis_ = person-total correlation coefficient for which the calculation was based on subsamples; Gnormed = global (i.e., the average of the scale-specific scores was computed) normed Guttman error index for polytomous items; H_t_ = global (i.e., the average of the scale-specific scores was computed) person scalability index; lz = global (i.e., the average of the scale-specific scores was computed) standardized log-likelihood index for polytomous items; infit MSE = global (i.e., the average of the scale-specific statistics was computed) Rasch model-based infit mean square error; outfit MSE = global (i.e., the average of the scale-specific statistics was computed) Rasch model-based outfit mean square error; INDCHI = global (i.e., the average of the scale-specific scores was computed) individual contribution to model misfit for which the calculation of the two person-specific model log-likelihood values was based on subsamples.

* *p* < .05 (two-tailed).

** *p* < .01 (two-tailed).

# Table S25

*Descriptive Statistics of the Substantive Measures and Careless Response Indices for the Partially Careless Responding Group (i.e., Careless 33%) in Study 4 at t2*

| Scale | 1. | 2. | 3. | 4. | 5. | 6. | 7. | 8. | 9. | 10. | 11. | 12. | 13. | 14. | 15. | 16. | 17. | 18. | 19. | 20. |
| --- | --- | --- | --- | --- | --- | --- | --- | --- | --- | --- | --- | --- | --- | --- | --- | --- | --- | --- | --- | --- |
| 1. EX | 1 |  |  |  |  |  |  |  |  |  |  |  |  |  |  |  |  |  |  |  |
| 2. AG | .226* | 1 |  |  |  |  |  |  |  |  |  |  |  |  |  |  |  |  |  |  |
| 3. CO | .278** | .360** | 1 |  |  |  |  |  |  |  |  |  |  |  |  |  |  |  |  |  |
| 4. EM | -.412** | -.339** | -.543** | 1 |  |  |  |  |  |  |  |  |  |  |  |  |  |  |  |  |
| 5. OP | .374** | .287** | .324** | -.199* | 1 |  |  |  |  |  |  |  |  |  |  |  |  |  |  |  |
| 6. Bogus | .054 | -.364** | -.185* | .189* | -.200* | 1 |  |  |  |  |  |  |  |  |  |  |  |  |  |  |
| 7. Time | .032 | .182* | .229* | -.123 | .020 | -.073 | 1 |  |  |  |  |  |  |  |  |  |  |  |  |  |
| 8. Longstring | -.049 | -.092 | -.120 | -.010 | -.189* | .119 | -.168 | 1 |  |  |  |  |  |  |  |  |  |  |  |  |
| 9. IRV | .137 | .170 | .372** | -.068 | .294** | .138 | .158 | -.190* | 1 |  |  |  |  |  |  |  |  |  |  |  |
| 10. Synonyms | .020 | .253** | .274** | -.231* | .326** | -.212* | .077 | .003 | .150 | 1 |  |  |  |  |  |  |  |  |  |  |
| 11. Antonyms | .128 | -.188* | -.263** | .223* | -.234* | .171 | -.175 | .004 | -.210* | -.578** | 1 |  |  |  |  |  |  |  |  |  |
| 12. RPR | -.090 | .217* | .280** | -.208* | .274** | -.231* | .072 | .057 | .127 | .678** | -.608** | 1 |  |  |  |  |  |  |  |  |
| 13. MD | .029 | -.158 | .017 | .176 | -.071 | .338** | .038 | -.148 | .670** | -.468** | .280** | -.404** | 1 |  |  |  |  |  |  |  |
| 14. *r*_pbis_ | .249** | .726** | .649** | -.482** | .604** | -.375** | .164 | -.060 | .092 | .395** | -.357** | .432** | -.325** | 1 |  |  |  |  |  |  |
| 15. Gnormed | .124 | -.045 | .140 | .065 | .099 | .227* | .119 | -.089 | .818** | -.208* | .109 | -.214* | .852** | -.215* | 1 |  |  |  |  |  |
| 16. H_t_ | .046 | .112 | .159 | -.264** | .085 | .068 | .026 | .069 | .042 | .220* | -.280** | .151 | -.240** | .319** | -.224* | 1 |  |  |  |  |
| 17. lz | -.078 | .100 | -.044 | -.157 | -.029 | -.264** | -.058 | .124 | -.761** | .326** | -.187* | .329** | -.893** | .288** | -.948** | .275** | 1 |  |  |  |
| 18. Infit MSE | .155 | .044 | .216* | .011 | .172 | .192* | .135 | -.105 | .853** | -.145 | .060 | -.163 | .828** | -.114 | .987** | -.185* | -.925** | 1 |  |  |
| 19. Outfit MSE | .141 | .006 | .185* | .048 | .145 | .220* | .137 | -.136 | .843** | -.184* | .092 | -.205* | .868** | -.157 | .984** | -.238** | -.955** | .988** | 1 |  |
| 20. INDCHI | .111 | .051 | .023 | .000 | .242** | -.105 | .013 | .028 | .046 | -.035 | .091 | -.131 | .039 | .092 | .165 | .012 | -.174 | .195* | .178 | 1 |
| *M* | 3.429 | 4.138 | 4.108 | 3.356 | 4.046 | 1.954 | 4.332 | 3.750 | 1.436 | 0.322 | -0.304 | 0.423 | 117.482 | 0.374 | 0.220 | 0.126 | -0.135 | 1.123 | 1.110 | -0.216 |
| *SD* | 0.636 | 0.691 | 0.678 | 0.761 | 0.766 | 0.771 | 2.607 | 1.282 | 0.342 | 0.262 | 0.283 | 0.413 | 45.493 | 0.216 | 0.122 | 0.078 | 1.180 | 0.602 | 0.566 | 3.379 |

*Note.* The careful responding group included 118 participants. All reported Pearson correlation coefficients and means and standard deviations are based on 118 observations. EX = Extraversion; AG = Agreeableness; CO = Conscientiousness; EM = Negative Emotionality; OP = Open-Mindedness; bogus = average score of the eight bogus items; time = average response time per item; longstring = maximum longstring; IRV = intra-individual response variability; synonyms = psychometric synonyms; antonyms = psychometric antonyms; RPR = resampled personal reliability; MD = Mahalanobis distance measure for which the calculation was based on subsamples; *r*_pbis_ = person-total correlation coefficient for which the calculation was based on subsamples; Gnormed = global (i.e., the average of the scale-specific scores was computed) normed Guttman error index for polytomous items; H_t_ = global (i.e., the average of the scale-specific scores was computed) person scalability index; lz = global (i.e., the average of the scale-specific scores was computed) standardized log-likelihood index for polytomous items; infit MSE = global (i.e., the average of the scale-specific statistics was computed) Rasch model-based infit mean square error; outfit MSE = global (i.e., the average of the scale-specific statistics was computed) Rasch model-based outfit mean square error; INDCHI = global (i.e., the average of the scale-specific scores was computed) individual contribution to model misfit for which the calculation of the two person-specific model log-likelihood values was based on subsamples.

* *p* < .05 (two-tailed).

** *p* < .01 (two-tailed).

# Table S26

*Descriptive Statistics of the Substantive Measures and Careless Response Indices for the Fully Careless Responding Group (i.e., Careless 100%) in Study 4 at t2*

| Scale | 1. | 2. | 3. | 4. | 5. | 6. | 7. | 8. | 9. | 10. | 11. | 12. | 13. | 14. | 15. | 16. | 17. | 18. | 19. | 20. |
| --- | --- | --- | --- | --- | --- | --- | --- | --- | --- | --- | --- | --- | --- | --- | --- | --- | --- | --- | --- | --- |
| 1. EX | 1 |  |  |  |  |  |  |  |  |  |  |  |  |  |  |  |  |  |  |  |
| 2. AG | .163 | 1 |  |  |  |  |  |  |  |  |  |  |  |  |  |  |  |  |  |  |
| 3. CO | .170 | .520** | 1 |  |  |  |  |  |  |  |  |  |  |  |  |  |  |  |  |  |
| 4. EM | -.244** | -.339** | -.273** | 1 |  |  |  |  |  |  |  |  |  |  |  |  |  |  |  |  |
| 5. OP | .373** | .235** | .257** | -.271** | 1 |  |  |  |  |  |  |  |  |  |  |  |  |  |  |  |
| 6. Bogus | -.027 | -.443** | -.409** | .147 | -.086 | 1 |  |  |  |  |  |  |  |  |  |  |  |  |  |  |
| 7. Time | .145 | .082 | .138 | -.125 | .161 | -.303** | 1 |  |  |  |  |  |  |  |  |  |  |  |  |  |
| 8. Longstring | -.069 | -.062 | -.028 | .091 | .011 | .251** | -.062 | 1 |  |  |  |  |  |  |  |  |  |  |  |  |
| 9. IRV | .141 | .011 | .033 | -.233** | .182* | -.104 | .090 | -.688** | 1 |  |  |  |  |  |  |  |  |  |  |  |
| 10. Synonyms | .201* | .272** | .247* | -.347** | .277** | -.393** | .309** | -.166 | .216* | 1 |  |  |  |  |  |  |  |  |  |  |
| 11. Antonyms | -.100 | -.172 | -.120 | .263** | -.152 | .300** | -.237* | .093 | -.178 | -.524** | 1 |  |  |  |  |  |  |  |  |  |
| 12. RPR | .132 | .164 | .106 | -.226* | .153 | -.307** | .329** | .020 | .184* | .569** | -.668** | 1 |  |  |  |  |  |  |  |  |
| 13. MD | -.071 | -.209* | -.180* | .023 | .001 | .278** | -.184* | -.373** | .763** | -.282** | .206* | -.063 | 1 |  |  |  |  |  |  |  |
| 14. *r*_pbis_ | .194* | .759** | .770** | -.348** | .500** | -.493** | .146 | -.032 | -.008 | .345** | -.139 | .156 | -.254** | 1 |  |  |  |  |  |  |
| 15. Gnormed | .040 | -.174 | -.141 | -.058 | .158 | .504** | -.119 | .138 | .381** | -.067 | .092 | -.004 | .703** | -.170 | 1 |  |  |  |  |  |
| 16. H_t_ | -.032 | .158 | .313** | .211* | .036 | .182* | -.099 | .367** | -.487** | -.032 | .192* | -.379** | -.366** | .371** | -.104 | 1 |  |  |  |  |
| 17. lz | .040 | .170 | .135 | -.059 | -.040 | -.494** | .166 | -.111 | -.355** | .198* | -.191* | .042 | -.748** | .188* | -.968** | .098 | 1 |  |  |  |
| 18. Infit MSE | .018 | -.193* | -.121 | -.045 | .167 | .502** | -.113 | .151 | .370** | -.070 | .102 | -.013 | .699** | -.156 | .994** | -.082 | -.962** | 1 |  |  |
| 19. Outfit MSE | .014 | -.202* | -.135 | -.036 | .145 | .504** | -.129 | .150 | .370** | -.077 | .108 | -.016 | .706** | -.175 | .995** | -.094 | -.969** | .998** | 1 |  |
| 20. INDCHI | -.045 | -.097 | -.004 | .151 | .033 | .519** | -.147 | .576** | -.351** | -.039 | .195* | -.133 | .032 | -.012 | .637** | .361** | -.628** | .647** | .648** | 1 |
| *M* | 3.567 | 3.494 | 3.581 | 3.425 | 3.496 | 3.851 | 2.365 | 10.220 | 1.177 | 0.072 | -0.045 | -0.179 | 131.054 | 0.026 | 0.279 | 0.112 | -0.992 | 1.342 | 1.339 | 2.016 |
| *SD* | 0.414 | 0.654 | 0.725 | 0.508 | 0.503 | 1.238 | 2.139 | 16.429 | 0.584 | 0.271 | 0.313 | 0.561 | 62.447 | 0.238 | 0.182 | 0.103 | 2.158 | 0.861 | 0.849 | 9.996 |

*Note.* The careful responding group included 122 participants. All reported Pearson correlation coefficients and means and standard deviations are based on 122 observations except for those that involve the bogus item average score (*n* = 121), the synonym and antonym index (*n* = 108) and the person-total correlation coefficient (*n* = 112). In the case of the bogus item average score, the missing value occurred because one participant was not responding to any of the bogus items. In the case of the synonym and antonym index and the person-total correlation coefficient, the missing values occurred because the response protocols of some participants had little or even no variance. EX = Extraversion; AG = Agreeableness; CO = Conscientiousness; EM = Negative Emotionality; OP = Open-Mindedness; bogus = average score of the three bogus items; time = average response time per item; longstring = maximum longstring; IRV = intra-individual response variability; synonyms = psychometric synonyms; antonyms = psychometric antonyms; RPR = resampled personal reliability; MD = Mahalanobis distance measure for which the calculation was based on subsamples; *r*_pbis_ = person-total correlation coefficient for which the calculation was based on subsamples; Gnormed = global (i.e., the average of the scale-specific scores was computed) normed Guttman error index for polytomous items; H_t_ = global (i.e., the average of the scale-specific scores was computed) person scalability index; lz = global (i.e., the average of the scale-specific scores was computed) standardized log-likelihood index for polytomous items; infit MSE = global (i.e., the average of the scale-specific statistics was computed) Rasch model-based infit mean square error; outfit MSE = global (i.e., the average of the scale-specific statistics was computed) Rasch model-based outfit mean square error; INDCHI = global (i.e., the average of the scale-specific scores was computed) individual contribution to model misfit for which the calculation of the two person-specific model log-likelihood values was based on subsamples.

* *p* < .05 (two-tailed).

** *p* < .01 (two-tailed).

# Table S27

*Ordered Accuracies of the 14 Indirect Indices in Detecting Partially Careless (i.e., Careless 33%)* *Responding and Corresponding Pairwise Comparison Results in Study 4*

| Index | AUC_[95% CI]_ | Sen95 ^a^ | Sen99 ^b^ | 1. | 2. | 3. | 4. | 5. | 6. | 7. | 8. | 9. | 10. | 11. | 12. | 13. |
| --- | --- | --- | --- | --- | --- | --- | --- | --- | --- | --- | --- | --- | --- | --- | --- | --- |
| 1. MD | .88_[.84, .91]_ | .53 | .36 |  |  |  |  |  |  |  |  |  |  |  |  |  |
| 2. Synonyms | .85_[.80, .89]_ | .48 | .33 | 2.00 |  |  |  |  |  |  |  |  |  |  |  |  |
| 3. RPR | .84_[.80, .89]_ | .41 | .07 | 2.65 | 0.09 |  |  |  |  |  |  |  |  |  |  |  |
| 4. Antonyms | .80_[.76, .85]_ | .36 | .31 | 10.14 | 4.86 | 3.56 |  |  |  |  |  |  |  |  |  |  |
| 5. Gnormed | .78_[.74, .83]_ | .33 | .14 | 43.81 | 6.44 | 5.50 | 0.37 |  |  |  |  |  |  |  |  |  |
| 6. *r*_pbis_ | .77_[.73, .81]_ | .20 | .05 | 21.62 | 12.60 | 12.05 | 1.54 | 0.40 |  |  |  |  |  |  |  |  |
| 7. H_t_ | .73_[.68, .79]_ | .11 | .03 | 22.51 | 14.19 | 11.28 | 4.37 | 2.56 | 1.14 |  |  |  |  |  |  |  |
| 8. Time | .69_[.65, .74]_ | .20 | .14 | 42.31 | 24.70 | 25.84 | 11.49 | 7.86 | 6.10 | 1.33 |  |  |  |  |  |  |
| 9. Outfit MSE | .59_[.53, .64]_ | .12 | .02 | 204.86 | 90.24 | 87.79 | 51.46 | 209.75 | 41.23 | 20.63 | 8.01 |  |  |  |  |  |
| 10. lz | .58_[.52, .63]_ | .10 | NA ^c^ | 204.65 | 97.81 | 94.24 | 53.81 | 204.76 | 42.40 | 22.02 | 9.09 | 0.72 |  |  |  |  |
| 11. Infit MSE | .58_[.53, .63]_ | .09 | .03 | 209.24 | 93.85 | 91.71 | 54.92 | 235.78 | 45.58 | 22.08 | 9.22 | 4.61 | 0.00 |  |  |  |
| 12. Longstring | .58_[.52, .64]_ | .10 | .05 | 83.02 | 57.79 | 63.08 | 37.64 | 31.11 | 27.24 | 13.33 | 12.43 | 0.04 | 0.00 | 0.00 |  |  |
| 13. INDCHI | .56_[.49, .63]_ | .14 | .04 | 74.61 | 50.26 | 52.44 | 34.38 | 35.64 | 26.28 | 14.30 | 10.61 | 0.53 | 0.28 | 0.28 | 0.21 |  |
| 14. IRV | .48_[.44, .52]_ | .06 | .03 | 149.28 | 146.42 | 158.55 | 103.79 | 61.39 | 90.93 | 46.94 | 60.57 | 5.81 | 4.89 | 4.95 | 8.87 | 3.83 |

*Note.* The ratings of the first and second measurement of the partially careless responding condition (*n* = 118) were used to determine the classification accuracy. All indices were coded such that higher scores were more indicative of careless responding. Values in the matrix part represent the χ^2^ values for the pairwise comparisons with one degree of freedom. The Bonferroni-corrected critical χ^2^ value for each of the 91 comparisons was 11.94; if this value is exceeded, the two AUCs can be considered significantly different from each other, and the corresponding cells in the matrix part are shown in gray. AUC = area under the receiver operating characteristic curve; time = average response time per item; longstring = maximum longstring; IRV = intra-individual response variability; synonyms = psychometric synonyms; antonyms = psychometric antonyms; RPR = resampled personal reliability; MD = Mahalanobis distance measure for which the calculation was based on subsamples; *r*_pbis_ = person-total correlation coefficient for which the calculation was based on subsamples; Gnormed = global (i.e., the average of the scale-specific scores was computed) normed Guttman error index for polytomous items; H_t_ = global (i.e., the average of the scale-specific scores was computed) person scalability index; lz = global (i.e., the average of the scale-specific scores was computed) standardized log-likelihood index for polytomous items; infit MSE = global (i.e., the average of the scale-specific statistics was computed) Rasch model-based infit mean square error; outfit MSE = global (i.e., the average of the scale-specific statistics was computed) Rasch model-based outfit mean square error; INDCHI = global (i.e., the average of the scale-specific scores was computed) individual contribution to model misfit for which the calculation of the two person-specific model log-likelihood values was based on subsamples.

^a^ Sensitivity of the index at specificity level of 95% (i.e., false-positive rate of 5%). ^b^ Sensitivity of the index at specificity level of 99% (i.e., false-positive rate of 1%). ^c^ Sensitivity could not be computed because of missing values in the matrix (i.e., non-zero sensitivities could only be calculated for false-positive rates that were higher than the 1% level that we aimed for).

# Table S28

*Ordered Accuracies of the 14 Indirect Indices in Detecting Fully Careless (i.e., Careless 100%)* *Responding and Corresponding Pairwise Comparison Results in Study 4*

| Index | AUC_[95% CI]_ | Sen95 ^a^ | Sen99 ^b^ | 1. | 2. | 3. | 4. | 5. | 6. | 7. | 8. | 9. | 10. | 11. | 12. | 13. |
| --- | --- | --- | --- | --- | --- | --- | --- | --- | --- | --- | --- | --- | --- | --- | --- | --- |
| 1. Synonyms | .94_[.91, .98]_ | .87 | .73 |  |  |  |  |  |  |  |  |  |  |  |  |  |
| 2. *r*_pbis_ | .94_[.91, .97]_ | .79 | .18 | 0.03 |  |  |  |  |  |  |  |  |  |  |  |  |
| 3. RPR | .93_[.90, .97]_ | .78 | .53 | 0.78 | 0.09 |  |  |  |  |  |  |  |  |  |  |  |
| 4. Time | .91_[.88, .95]_ | .80 | .75 | 1.78 | 1.26 | 1.10 |  |  |  |  |  |  |  |  |  |  |
| 5. Antonyms | .90_[.84, .96]_ | .69 | .41 | 2.47 | 1.53 | 1.63 | 0.16 |  |  |  |  |  |  |  |  |  |
| 6. MD | .86_[.81, .91]_ | .70 | .57 | 10.23 | 8.03 | 8.66 | 3.88 | 1.43 |  |  |  |  |  |  |  |  |
| 7. Gnormed | .83_[.78, .88]_ | .61 | .30 | 14.67 | 13.21 | 13.14 | 8.42 | 3.76 | 5.15 |  |  |  |  |  |  |  |
| 8. lz | .72_[.65, .78]_ | .38 | .12 | 51.90 | 47.97 | 49.55 | 35.03 | 22.19 | 51.97 | 61.15 |  |  |  |  |  |  |
| 9. H_t_ | .70_[.65, .76]_ | .33 | .27 | 53.30 | 47.50 | 47.52 | 39.56 | 24.19 | 20.81 | 11.99 | 0.11 |  |  |  |  |  |
| 10. Longstring | .70_[.64, .76]_ | .25 | .23 | 55.35 | 55.77 | 54.12 | 37.78 | 23.33 | 12.33 | 7.59 | 0.12 | 0.01 |  |  |  |  |
| 11. Outfit MSE | .69_[.63, .76]_ | .34 | .12 | 54.39 | 52.74 | 52.57 | 40.50 | 25.81 | 53.08 | 71.57 | 6.86 | 0.08 | 0.02 |  |  |  |
| 12. Infit MSE | .69_[.62, .75]_ | .26 | .12 | 56.70 | 54.48 | 55.05 | 42.34 | 26.90 | 55.30 | 74.61 | 8.55 | 0.13 | 0.05 | 3.16 |  |  |
| 13. IRV | .60_[.53, .66]_ | .31 | .23 | 90.61 | 91.56 | 92.25 | 81.82 | 45.94 | 29.15 | 20.38 | 4.52 | 4.71 | 10.57 | 2.67 | 2.48 |  |
| 14. INDCHI | .49_[.42, .56]_ | .25 | .21 | 138.97 | 126.52 | 138.30 | 119.38 | 80.99 | 77.70 | 74.46 | 31.04 | 16.21 | 21.39 | 25.13 | 24.70 | 4.99 |

*Note.* The ratings of the first and second measurement of the fully careless responding condition (*n* = 122) were used to determine the classification accuracy. Because four indices (synonyms, antonyms, *r*_pbis_, average score of bogus items) could not be computed for every participant of the fully careless responding condition, all reported estimates and test statistics are based on 100 multiply imputed data sets. All indices were coded such that higher scores were more indicative of careless responding. Values in the matrix part represent the *F*-values for the pairwise comparisons with one degree of freedom for the numerator and degrees of freedom that ranged from 224.6 to ∞ for the denominator. With the most conservative denominator value of 224.6, the Bonferroni-corrected critical *F*-value for each of the 91 comparisons was 12.29; if this value is exceeded, the two AUCs can be considered significantly different from each other, and the corresponding cells in the matrix part are shown in gray. AUC = area under the receiver operating characteristic curve; time = average response time per item; longstring = maximum longstring; IRV = intra-individual response variability; synonyms = psychometric synonyms; antonyms = psychometric antonyms; RPR = resampled personal reliability; MD = Mahalanobis distance measure for which the calculation was based on subsamples; *r*_pbis_ = person-total correlation coefficient for which the calculation was based on subsamples; Gnormed = global (i.e., the average of the scale-specific scores was computed) normed Guttman error index for polytomous items; H_t_ = global (i.e., the average of the scale-specific scores was computed) person scalability index; lz = global (i.e., the average of the scale-specific scores was computed) standardized log-likelihood index for polytomous items; infit MSE = global (i.e., the average of the scale-specific statistics was computed) Rasch model-based infit mean square error; outfit MSE = global (i.e., the average of the scale-specific statistics was computed) Rasch model-based outfit mean square error; INDCHI = global (i.e., the average of the scale-specific scores was computed) individual contribution to model misfit for which the calculation of the two person-specific model log-likelihood values was based on subsamples.

^a^ Sensitivity of the index at specificity level of 95% (i.e., false-positive rate of 5%). ^b^ Sensitivity of the index at specificity level of 99% (i.e., false-positive rate of 1%). ^c^ Sensitivities could not be computed because of missing values in the matrix (i.e., non-zero sensitivities could only be calculated for false-positive rates that were higher than the 1% level that we aimed for).

# Table S29

*Incremental Validity Beyond Average Score on Bogus Items of Each Indirect Index in Study 4*

|  | Careless 33% ^a^ | |  | Careless 100% ^b^ | |
| --- | --- | --- | --- | --- | --- |
| Index | AUC_[95% CI]_ | χ^2^(1) |  | AUC_[95% CI]_ | χ^2^(1) |
| Bogus (reference) | .89_[.85, .93]_ |  |  | .98 _[.96, .998]_ |  |
| Bogus + MD | .94_[.92, .97]_ | 13.87 |  | .98_[.96, .998]_ | 0.00 |
| Bogus + Synonyms | .93_[.90, .96]_ | 8.69 |  | .97_[.95, .998]_ | 2.16 |
| Bogus + RPR | .92_[.88, .95]_ | 5.39 |  | .97_[.95, .998]_ | 2.54 |
| Bogus + Antonyms | .92_[.89, .96]_ | 9.53 |  | .98_[.96, .998]_ | 0.76 |
| Bogus + Gnormed | .93_[.90, .96]_ | 10.48 |  | .99_[.97, .998]_ | 1.02 |
| Bogus + *r*_pbis_ | .92_[.88, .95]_ | 7.21 |  | .98_[.95, .998]_ | 0.92 |
| Bogus + H_t_ | .92_[.88, .95]_ | 6.47 |  | .98_[.97, .998]_ | 1.20 |
| Bogus + Time | .90_[.86, .94]_ | 1.03 |  | .98_[.96, .998]_ | 0.01 |
| Bogus + Outfit MSE | .91_[.87, .94]_ | 5.90 |  | .98_[.96, .998]_ | 0.09 |
| Bogus + lz | .90_[.87, .94]_ | 4.00 |  | .98_[.97, .998]_ | 1.04 |
| Bogus + Infit MSE | .91_[.87, .94]_ | 5.58 |  | .98_[.96, .998]_ | 0.06 |
| Bogus + Longstring | .89_[.85, .93]_ | 0.20 |  | .98_[.95, .998]_ | 1.35 |
| Bogus + IRV | - | - |  | .97_[.95, .997]_ | 2.29 |

*Note*. Only indirect indices that turned out be effective in detecting partially or fully careless responding were examined. Because four indices (i.e., synonyms, antonyms, *r*_pbis_, average score of bogus items) could not be computed every participant of the fully careless responding condition, we used linear multiple imputation predictions (across 100 imputations) of logit models as input for the ROC analyses in the fully careless responding condition. All indices were coded such that higher scores were more indicative of careless responding. The Bonferroni-corrected critical χ^2^ value for each of the 25 comparisons was 9.55 (i.e., 12 pairwise comparisons in the partially careless responding condition, 13 pairwise comparisons in the fully careless responding condition). If this value is exceeded, the AUCs can be considered significantly different from each other. AUC = area under the receiver operating characteristic curve; time = average response time per item; longstring = maximum longstring; IRV = intra-individual response variability; synonyms = psychometric synonyms; antonyms = psychometric antonyms; RPR = resampled personal reliability; MD = Mahalanobis distance measure for which the calculation was based on subsamples; *r*_pbis_ = person-total correlation coefficient for which the calculation was based on subsamples; Gnormed = global (i.e., the average of the scale-specific scores was computed) normed Guttman error index for polytomous items; H_t_ = global (i.e., the average of the scale-specific scores was computed) person scalability index; lz = global (i.e., the average of the scale-specific scores was computed) standardized log-likelihood index for polytomous items; infit MSE = global (i.e., the average of the scale-specific statistics was computed) Rasch model-based infit mean square error; outfit MSE = global (i.e., the average of the scale-specific statistics was computed) Rasch model-based outfit mean square error.

^a^ The ratings of the first and second measurement of the partially careless responding condition (*n* = 118) were used to determine the classification accuracy. ^b^ The ratings of the first and second measurement of the fully careless responding condition (*n* = 122) were used to determine the classification accuracy.

## Manipulation Check Study 5

To examine whether the respondents followed our response instructions, we compared the t1-t2 mean differences of the five substantive scales and of three indirect careless response indices (i.e., average response time per item, IRV, RPR) across the four response conditions (see Table S30). In line with our expectations, the t1-t2 mean differences were significantly different across the four response conditions for three of the five substantive scales (i.e., agreeableness, conscientiousness, and open-mindedness). The scores of the agreeableness, conscientiousness and open-mindedness scales changed only marginally between t1 and t2 in the two careful responding conditions, but those in the two careless responding conditions became biased towards the scale mid-point. As anticipated, the bias towards the scale mid-point turned out to be more pronounced in the fully careless responding condition than in the partially careless responding condition. A similar pattern occurred when we examined the t1-t2 mean differences in average response time per item, IRV, and RPR across the four response conditions. Compared to the participants in the two careful responding conditions, those in the fully careless responding condition increased their responding speed to a much larger degree and changed from consistent to inconsistent responding between t1 and t2.

In addition, we correlated the response vectors of the first and second measurement within each person and examined the within-person response vector correlation coefficients. The global test for equality indicated a significant inequality between the within-person response vector correlation coefficients of the four conditions, χ^2^(3) = 517.77, *p* < .001. Bonferroni-corrected pairwise comparisons then revealed the expected pattern: First, the within-person response vector correlation coefficients of the unincentivized (*M*_1_ = .79, *SD*_1_ = .33) and incentivized careful responding group (*M*_2_ = .82, *SD*_2_ = .26) were high and did not differ from each other, *M*_2_-*M*_1_ = 0.03, *z* = 0.71, according to the Bonferroni-corrected critical *z-*value of 2.64. Second the partially careless responding group (*M*_3_ = .57, *SD*_3_ = .23) and fully careless responding group (*M*_4_ = .08, *SD*_4_ = .28) had significantly lower within-person response vector correlation coefficients than the two carful responding groups (*M*_1_-*M*_3_ = 0.22, *z* = 5.92; *M*_1_-*M*_4_ = 0.70, *z* = 17.77; *M*_2_-*M*_3_ = 0.25, *z* = 7.71; *M*_2_-*M*_4_ = 0.73, *z* = 20.89). Third, the within-person response vector correlation coefficients of the fully careless responding group were significantly lower than those of the partially careless responding group (*M*_3_-*M*_4_ = 0.49, *z* = 14.69). Based on these results, the manipulation was successful, and we therefore proceeded with the main analyses.

# Table S30

*Comparisons of t1-t2 Mean Differences of Substantive Scale Scores and Selected Indirect Careless Responding Indices Across the Conditions in Study 5*

|  | Careful Unincentivized | | | | | |  | Careful Incentivized | | | | | |  | Careless 33% | | | | | |  | Careless 100% | | | | | |  |  |
| --- | --- | --- | --- | --- | --- | --- | --- | --- | --- | --- | --- | --- | --- | --- | --- | --- | --- | --- | --- | --- | --- | --- | --- | --- | --- | --- | --- | --- | --- |
|  | t1 | |  | t2 | |  |  | t1 | |  | t2 | |  |  | t1 | |  | t2 | |  |  | t1 | |  | t2 | |  |  |  |
| Measure | *M* | *SD* |  | *M* | *SD* | Δ*M* |  | *M* | *SD* |  | *M* | *SD* | Δ*M* |  | *M* | *SD* |  | *M* | *SD* | Δ*M* |  | *M* | *SD* |  | *M* | *SD* | Δ*M* |  | χ^2^(3) |
| Substantive Scale |  |  |  |  |  |  |  |  |  |  |  |  |  |  |  |  |  |  |  |  |  |  |  |  |  |  |  |  |  |
| Extraversion | 3.34 | 0.81 |  | 3.31 | 0.85 | -0.03 |  | 3.43 | 0.87 |  | 3.50 | 0.91 | 0.07 |  | 3.53 | 0.78 |  | 3.48 | 0.68 | -0.05 |  | 3.54 | 0.88 |  | 3.50 | 0.46 | -0.04 |  | 5.63 |
| Agreeableness | 4.53 | 0.74 |  | 4.42 | 0.94 | -0.11_ab_ |  | 4.38 | 0.70 |  | 4.36 | 0.80 | -0.02_b_ |  | 4.37 | 0.68 |  | 4.13 | 0.60 | -0.24_a_ |  | 4.41 | 0.70 |  | 3.59 | 0.55 | -0.82_c_ |  | 96.85* |
| Conscientiousness | 4.43 | 0.80 |  | 4.38 | 0.91 | -0.04_a_ |  | 4.35 | 0.83 |  | 4.35 | 0.91 | -0.01_a_ |  | 4.28 | 0.88 |  | 4.01 | 0.74 | -0.27_b_ |  | 4.32 | 0.84 |  | 3.58 | 0.54 | -0.74_c_ |  | 77.99* |
| Emotionality | 3.27 | 1.05 |  | 3.33 | 1.12 | 0.05 |  | 3.44 | 1.02 |  | 3.35 | 1.05 | -0.09 |  | 3.45 | 0.99 |  | 3.39 | 0.77 | -0.06 |  | 3.36 | 1.06 |  | 3.53 | 0.52 | 0.17 |  | 9.28 |
| Open-mindedness | 4.22 | 0.78 |  | 4.19 | 0.85 | -0.03_a_ |  | 4.37 | 0.87 |  | 4.29 | 0.94 | -0.08_ab_ |  | 4.17 | 0.88 |  | 3.94 | 0.74 | -0.24_b_ |  | 4.36 | 0.77 |  | 3.53 | 0.45 | -0.83_c_ |  | 110.77* |
| Careless Response Indices |  |  |  |  |  |  |  |  |  |  |  |  |  |  |  |  |  |  |  |  |  |  |  |  |  |  |  |  |  |
| Time | 5.87 | 2.87 |  | 4.72 | 3.13 | -1.15_a_ |  | 6.71 | 4.27 |  | 5.06 | 3.17 | -1.65_a_ |  | 5.53 | 3.40 |  | 4.14 | 2.57 | -1.39_a_ |  | 5.90 | 3.81 |  | 2.29 | 1.67 | -3.61_b_ |  | 36.51* |
| IRV | 1.45 | 0.34 |  | 1.46 | 0.37 | 0.01_a_ |  | 1.44 | 0.30 |  | 1.43 | 0.36 | -0.02_a_ |  | 1.38 | 0.32 |  | 1.36 | 0.30 | -0.03_a_ |  | 1.42 | 0.30 |  | 1.16 | 0.47 | -0.26_b_ |  | 28.75* |
| RPR | 0.79 | 0.20 |  | 0.79 | 0.31 | 0.00_a_ |  | 0.78 | 0.19 |  | 0.78 | 0.26 | 0.01_a_ |  | 0.75 | 0.27 |  | 0.43 | 0.43 | -0.32_b_ |  | 0.77 | 0.23 |  | -0.14 | 0.60 | -0.91_c_ |  | 315.45* |

*Note*. Means and standard deviations are based on univariate sample statistics for the unincentivized careful responding condition (*n* = 120), the incentivized careful responding condition (*n* = 120), the partially careless responding (i.e., Careless 33%) condition (*n* = 120), and the fully careless responding (i.e., Careless 100%) condition (*n* = 121); t1 = first measurement, t2 = second measurement; Δ*M* = estimated latent difference between t2 and t1 measurement; reported Delta estimates (i.e., ∆*M*) may not exactly match reported t1 and t2 values due to rounding error. In each row, ∆*M* values with different subscripts are significantly different from each other (i.e., α = .025/36, with the critical z-value of 3.20). χ^2^ = χ^2^ values were obtained by conducting Wald tests of parameter constraints that were based on robust full information maximum likelihood estimation (MLR) in Mplus Version 8.8.

^*^ Larger than the critical χ^2^(3) value of 12.36 (i.e., α = .05/8), which indicates global inequality between the ∆*M* values.

# Table S31

*Descriptive Statistics of the Substantive Measures and Careless Response Indices for the Unincentivized Careful Responding Group in Study 5 at t1*

| Scale | 1. | 2. | 3. | 4. | 5. | 6. | 7. | 8. | 9. | 10. | 11. | 12. | 13. | 14. | 15. | 16. | 17. | 18. | 19. | 20. |
| --- | --- | --- | --- | --- | --- | --- | --- | --- | --- | --- | --- | --- | --- | --- | --- | --- | --- | --- | --- | --- |
| 1. EX | 1 |  |  |  |  |  |  |  |  |  |  |  |  |  |  |  |  |  |  |  |
| 2. AG | .205* | 1 |  |  |  |  |  |  |  |  |  |  |  |  |  |  |  |  |  |  |
| 3. CO | .302** | .356** | 1 |  |  |  |  |  |  |  |  |  |  |  |  |  |  |  |  |  |
| 4. EM | -.457** | -.330** | -.416** | 1 |  |  |  |  |  |  |  |  |  |  |  |  |  |  |  |  |
| 5. OP | .102 | .166 | .077 | -.086 | 1 |  |  |  |  |  |  |  |  |  |  |  |  |  |  |  |
| 6. Ires | .257** | .043 | .164 | -.081 | .034 | 1 |  |  |  |  |  |  |  |  |  |  |  |  |  |  |
| 7. Time | .134 | .172 | -.018 | .008 | -.048 | .191* | 1 |  |  |  |  |  |  |  |  |  |  |  |  |  |
| 8. Longstring | -.285** | -.245** | -.053 | .187* | -.266** | -.107 | -.033 | 1 |  |  |  |  |  |  |  |  |  |  |  |  |
| 9. IRV | .037 | .422** | .428** | -.175 | .214* | .171 | .104 | -.289** | 1 |  |  |  |  |  |  |  |  |  |  |  |
| 10. Synonyms | .080 | .281** | .384** | -.233* | .009 | -.010 | -.017 | -.159 | .309** | 1 |  |  |  |  |  |  |  |  |  |  |
| 11. Antonyms | -.062 | -.148 | -.295** | .221* | .065 | -.005 | .015 | .121 | -.311** | -.806** | 1 |  |  |  |  |  |  |  |  |  |
| 12. RPR | -.063 | .142 | .256** | -.269** | .047 | -.025 | -.094 | -.143 | .172 | .654** | -.686** | 1 |  |  |  |  |  |  |  |  |
| 13. MD | -.106 | -.079 | -.079 | .122 | -.053 | .201* | .128 | -.027 | .592** | -.379** | .243** | -.448** | 1 |  |  |  |  |  |  |  |
| 14. *r*_pbis_ | .132 | .661** | .592** | -.294** | .521** | .071 | .034 | -.169 | .172 | .293** | -.153 | .279** | -.390** | 1 |  |  |  |  |  |  |
| 15. Gnormed | .001 | .150 | .158 | -.060 | -.009 | .191* | .139 | -.044 | .768** | -.102 | .013 | -.222* | .873** | -.207* | 1 |  |  |  |  |  |
| 16. H_t_ | -.331** | .109 | .057 | .107 | .057 | .117 | .029 | .035 | .114 | .088 | -.086 | .189* | -.110 | .298** | -.167 | 1 |  |  |  |  |
| 17. lz | .085 | -.041 | -.028 | -.059 | -.051 | -.194* | -.169 | .018 | -.657** | .272** | -.176 | .340** | -.921** | .248** | -.940** | .168 | 1 |  |  |  |
| 18. Infit MSE | -.003 | .146 | .165 | -.050 | .018 | .216* | .166 | -.045 | .764** | -.139 | .044 | -.254** | .894** | -.181* | .981** | -.146 | -.954** | 1 |  |  |
| 19. Outfit MSE | .009 | .121 | .132 | -.020 | .032 | .217* | .177 | -.054 | .741** | -.175 | .073 | -.290** | .905** | -.202* | .974** | -.173 | -.973** | .991** | 1 |  |
| 20. INDCHI | -.167 | -.010 | -.100 | .154 | .121 | .093 | .178 | .129 | -.031 | -.384** | .305** | -.320** | .305** | .080 | .288** | -.131 | -.475** | .329** | .378** | 1 |
| *M* | 3.341 | 4.531 | 4.427 | 3.274 | 4.223 | 0.005 | 5.872 | 3.190 | 1.445 | 0.672 | -0.657 | 0.790 | 62.683 | 0.545 | 0.125 | 0.183 | 0.076 | 1.029 | 0.998 | -0.686 |
| *SD* | 0.810 | 0.741 | 0.799 | 1.053 | 0.783 | 0.025 | 2.869 | 1.162 | 0.342 | 0.195 | 0.226 | 0.200 | 28.454 | 0.208 | 0.079 | 0.081 | 1.160 | 0.579 | 0.537 | 2.254 |

*Note.* The careful responding group included 120 participants. All reported Pearson correlation coefficients and means and standard deviations are based on 120 observations. EX = Extraversion; AG = Agreeableness; CO = Conscientiousness; EM = Negative Emotionality; OP = Open-Mindedness; Ires = average score of the scored instructed response items; time = average response time per item; longstring = maximum longstring; IRV = intra-individual response variability; synonyms = psychometric synonyms; antonyms = psychometric antonyms; RPR = resampled personal reliability; MD = Mahalanobis distance measure for which the calculation was based on subsamples; *r*_pbis_ = person-total correlation coefficient for which the calculation was based on subsamples; Gnormed = global (i.e., the average of the scale-specific scores was computed) normed Guttman error index for polytomous items; H_t_ = global (i.e., the average of the scale-specific scores was computed) person scalability index; lz = global (i.e., the average of the scale-specific scores was computed) standardized log-likelihood index for polytomous items; infit MSE = global (i.e., the average of the scale-specific statistics was computed) Rasch model-based infit mean square error; outfit MSE = global (i.e., the average of the scale-specific statistics was computed) Rasch model-based outfit mean square error; INDCHI = global (i.e., the average of the scale-specific scores was computed) individual contribution to model misfit for which the calculation of the two person-specific model log-likelihood values was based on subsamples.

* *p* < .05 (two-tailed).

** *p* < .01 (two-tailed).

# Table S32

*Descriptive Statistics of the Substantive Measures and Careless Response Indices for the Incentivized Careful Responding Group in Study 5 at t1*

| Scale | 1. | 2. | 3. | 4. | 5. | 6. | 7. | 8. | 9. | 10. | 11. | 12. | 13. | 14. | 15. | 16. | 17. | 18. | 19. | 20. |
| --- | --- | --- | --- | --- | --- | --- | --- | --- | --- | --- | --- | --- | --- | --- | --- | --- | --- | --- | --- | --- |
| 1. EX | 1 |  |  |  |  |  |  |  |  |  |  |  |  |  |  |  |  |  |  |  |
| 2. AG | .307** | 1 |  |  |  |  |  |  |  |  |  |  |  |  |  |  |  |  |  |  |
| 3. CO | .547** | .360** | 1 |  |  |  |  |  |  |  |  |  |  |  |  |  |  |  |  |  |
| 4. EM | -.592** | -.355** | -.565** | 1 |  |  |  |  |  |  |  |  |  |  |  |  |  |  |  |  |
| 5. OP | .457** | .293** | .367** | -.308** | 1 |  |  |  |  |  |  |  |  |  |  |  |  |  |  |  |
| 6. Ires | .059 | .056 | .035 | -.097 | .001 | 1 |  |  |  |  |  |  |  |  |  |  |  |  |  |  |
| 7. Time | .047 | -.145 | -.053 | .080 | -.094 | -.047 | 1 |  |  |  |  |  |  |  |  |  |  |  |  |  |
| 8. Longstring | -.083 | -.340** | -.163 | .159 | -.195* | -.121 | -.077 | 1 |  |  |  |  |  |  |  |  |  |  |  |  |
| 9. IRV | .212* | .419** | .514** | -.230* | .295** | .246** | -.135 | -.304** | 1 |  |  |  |  |  |  |  |  |  |  |  |
| 10. Synonyms | .163 | .417** | .341** | -.202* | .258** | .115 | -.060 | -.485** | .505** | 1 |  |  |  |  |  |  |  |  |  |  |
| 11. Antonyms | -.038 | -.219* | -.159 | .111 | -.103 | -.084 | .117 | .284** | -.363** | -.718** | 1 |  |  |  |  |  |  |  |  |  |
| 12. RPR | -.042 | .260** | .165 | -.192* | .187* | .032 | -.144 | -.531** | .298** | .696** | -.682** | 1 |  |  |  |  |  |  |  |  |
| 13. MD | -.102 | -.201* | -.029 | .163 | -.259** | .192* | .068 | .254** | .365** | -.379** | .322** | -.476** | 1 |  |  |  |  |  |  |  |
| 14. *r*_pbis_ | .427** | .651** | .658** | -.460** | .664** | -.039 | -.141 | -.251** | .250** | .309** | -.072 | .228* | -.371** | 1 |  |  |  |  |  |  |
| 15. Gnormed | .096 | .057 | .194* | -.054 | -.034 | .256** | -.016 | .154 | .564** | -.086 | .078 | -.239** | .801** | -.146 | 1 |  |  |  |  |  |
| 16. H_t_ | -.374** | .020 | -.194* | .233* | -.148 | -.094 | -.130 | .046 | -.078 | .065 | -.025 | .104 | -.227* | .118 | -.374** | 1 |  |  |  |  |
| 17. lz | .023 | .134 | -.145 | -.049 | .128 | -.220* | -.066 | -.212* | -.474** | .266** | -.265** | .397** | -.911** | .226* | -.790** | .226* | 1 |  |  |  |
| 18. Infit MSE | .135 | .088 | .257** | -.085 | .014 | .268** | -.015 | .144 | .595** | -.088 | .101 | -.268** | .815** | -.087 | .985** | -.394** | -.830** | 1 |  |  |
| 19. Outfit MSE | .041 | -.041 | .215* | -.011 | -.051 | .253** | .037 | .169 | .534** | -.196* | .197* | -.361** | .888** | -.139 | .859** | -.316** | -.960** | .909** | 1 |  |
| 20. INDCHI | .026 | -.057 | .090 | -.063 | .068 | -.033 | .020 | .219* | -.298** | -.314** | .354** | -.214* | .098 | .256** | .073 | -.003 | -.263** | .109 | .233* | 1 |
| *M* | 3.341 | 4.531 | 4.427 | 3.274 | 4.223 | 0.005 | 5.872 | 3.190 | 1.445 | 0.672 | -0.657 | 0.790 | 62.683 | 0.545 | 0.125 | 0.183 | 0.076 | 1.029 | 0.998 | -0.686 |
| *SD* | 0.810 | 0.741 | 0.799 | 1.053 | 0.783 | 0.025 | 2.869 | 1.162 | 0.342 | 0.195 | 0.226 | 0.200 | 28.454 | 0.208 | 0.079 | 0.081 | 1.160 | 0.579 | 0.537 | 2.254 |

*Note.* The careful responding group included 120 participants. All reported Pearson correlation coefficients and means and standard deviations are based on 120 observations. EX = Extraversion; AG = Agreeableness; CO = Conscientiousness; EM = Negative Emotionality; OP = Open-Mindedness; Ires = average score of the scored instructed response items; time = average response time per item; longstring = maximum longstring; IRV = intra-individual response variability; synonyms = psychometric synonyms; antonyms = psychometric antonyms; RPR = resampled personal reliability; MD = Mahalanobis distance measure for which the calculation was based on subsamples; *r*_pbis_ = person-total correlation coefficient for which the calculation was based on subsamples; Gnormed = global (i.e., the average of the scale-specific scores was computed) normed Guttman error index for polytomous items; H_t_ = global (i.e., the average of the scale-specific scores was computed) person scalability index; lz = global (i.e., the average of the scale-specific scores was computed) standardized log-likelihood index for polytomous items; infit MSE = global (i.e., the average of the scale-specific statistics was computed) Rasch model-based infit mean square error; outfit MSE = global (i.e., the average of the scale-specific statistics was computed) Rasch model-based outfit mean square error; INDCHI = global (i.e., the average of the scale-specific scores was computed) individual contribution to model misfit for which the calculation of the two person-specific model log-likelihood values was based on subsamples.

* *p* < .05 (two-tailed).

** *p* < .01 (two-tailed).

# Table S33

*Descriptive Statistics of the Substantive Measures and Careless Response Indices for the Partially Careless Responding Group (i.e., Careless 33%) in Study 5 at t1*

| Scale | 1. | 2. | 3. | 4. | 5. | 6. | 7. | 8. | 9. | 10. | 11. | 12. | 13. | 14. | 15. | 16. | 17. | 18. | 19. | 20. |
| --- | --- | --- | --- | --- | --- | --- | --- | --- | --- | --- | --- | --- | --- | --- | --- | --- | --- | --- | --- | --- |
| 1. EX | 1 |  |  |  |  |  |  |  |  |  |  |  |  |  |  |  |  |  |  |  |
| 2. AG | .037 | 1 |  |  |  |  |  |  |  |  |  |  |  |  |  |  |  |  |  |  |
| 3. CO | .284** | .235** | 1 |  |  |  |  |  |  |  |  |  |  |  |  |  |  |  |  |  |
| 4. EM | -.474** | -.242** | -.513** | 1 |  |  |  |  |  |  |  |  |  |  |  |  |  |  |  |  |
| 5. OP | .276** | .197* | .083 | -.029 | 1 |  |  |  |  |  |  |  |  |  |  |  |  |  |  |  |
| 6. Ires | .030 | -.113 | -.058 | -.120 | -.107 | 1 |  |  |  |  |  |  |  |  |  |  |  |  |  |  |
| 7. Time | -.091 | .156 | -.167 | .008 | .041 | .170 | 1 |  |  |  |  |  |  |  |  |  |  |  |  |  |
| 8. Longstring | -.186* | -.336** | -.134 | .161 | -.232* | .310** | -.054 | 1 |  |  |  |  |  |  |  |  |  |  |  |  |
| 9. IRV | .136 | .416** | .306** | -.031 | .439** | -.157 | .014 | -.415** | 1 |  |  |  |  |  |  |  |  |  |  |  |
| 10. Synonyms | .017 | .347** | .324** | -.224* | .184* | -.299** | -.020 | -.454** | .298** | 1 |  |  |  |  |  |  |  |  |  |  |
| 11. Antonyms | .067 | -.231* | -.176 | .075 | .006 | .289** | .116 | .296** | -.219* | -.690** | 1 |  |  |  |  |  |  |  |  |  |
| 12. RPR | -.092 | .340** | .215* | -.116 | .150 | -.495** | -.160 | -.553** | .347** | .721** | -.608** | 1 |  |  |  |  |  |  |  |  |
| 13. MD | .063 | -.093 | -.092 | .229* | .099 | .036 | -.008 | -.004 | .542** | -.427** | .377** | -.291** | 1 |  |  |  |  |  |  |  |
| 14. *r*_pbis_ | .156 | .624** | .663** | -.434** | .462** | -.137 | -.003 | -.274** | .228* | .347** | -.100 | .331** | -.275** | 1 |  |  |  |  |  |  |
| 15. Gnormed | .102 | .021 | -.003 | .166 | .197* | .033 | .065 | -.039 | .704** | -.217* | .173 | -.188* | .886** | -.207* | 1 |  |  |  |  |  |
| 16. H_t_ | -.294** | .031 | .162 | .018 | -.052 | -.057 | -.180* | .118 | -.019 | -.055 | .102 | .017 | -.099 | .257** | -.177 | 1 |  |  |  |  |
| 17. lz | -.108 | .080 | .037 | -.155 | -.141 | -.083 | -.027 | .031 | -.613** | .319** | -.303** | .263** | -.915** | .247** | -.949** | .154 | 1 |  |  |  |
| 18. Infit MSE | .162 | .025 | .037 | .127 | .223* | .026 | .020 | -.041 | .700** | -.226* | .195* | -.200* | .887** | -.168 | .985** | -.165 | -.943** | 1 |  |  |
| 19. Outfit MSE | .172 | .010 | .016 | .115 | .215* | .035 | .033 | -.058 | .688** | -.230* | .206* | -.210* | .892** | -.188* | .982** | -.208* | -.959** | .993** | 1 |  |
| 20. INDCHI | .127 | -.101 | .071 | -.169 | -.016 | .025 | -.073 | .038 | -.113 | -.228* | .264** | -.208* | .100 | .131 | .164 | -.089 | -.293** | .182* | .215* | 1 |
| *M* | 3.525 | 4.368 | 4.284 | 3.446 | 4.173 | 0.007 | 5.530 | 3.440 | 1.385 | 0.629 | -0.601 | 0.753 | 61.919 | 0.492 | 0.117 | 0.187 | 0.122 | 0.961 | 0.951 | -0.814 |
| *SD* | 0.781 | 0.682 | 0.877 | 0.989 | 0.879 | 0.029 | 3.402 | 1.488 | 0.320 | 0.208 | 0.247 | 0.268 | 27.013 | 0.247 | 0.065 | 0.083 | 1.056 | 0.487 | 0.460 | 2.055 |

*Note.* The careful responding group included 120 participants. All reported Pearson correlation coefficients and means and standard deviations are based on 120 observations. EX = Extraversion; AG = Agreeableness; CO = Conscientiousness; EM = Negative Emotionality; OP = Open-Mindedness; Ires = average score of the scored instructed response items; time = average response time per item; longstring = maximum longstring; IRV = intra-individual response variability; synonyms = psychometric synonyms; antonyms = psychometric antonyms; RPR = resampled personal reliability; MD = Mahalanobis distance measure for which the calculation was based on subsamples; *r*_pbis_ = person-total correlation coefficient for which the calculation was based on subsamples; Gnormed = global (i.e., the average of the scale-specific scores was computed) normed Guttman error index for polytomous items; H_t_ = global (i.e., the average of the scale-specific scores was computed) person scalability index; lz = global (i.e., the average of the scale-specific scores was computed) standardized log-likelihood index for polytomous items; infit MSE = global (i.e., the average of the scale-specific statistics was computed) Rasch model-based infit mean square error; outfit MSE = global (i.e., the average of the scale-specific statistics was computed) Rasch model-based outfit mean square error; INDCHI = global (i.e., the average of the scale-specific scores was computed) individual contribution to model misfit for which the calculation of the two person-specific model log-likelihood values was based on subsamples.

* *p* < .05 (two-tailed).

** *p* < .01 (two-tailed).

# Table S34

*Descriptive Statistics of the Substantive Measures and Careless Response Indices for the Fully Careless Responding Group (i.e., Careless 100%) in Study 5 at t1*

| Scale | 1. | 2. | 3. | 4. | 5. | 6. | 7. | 8. | 9. | 10. | 11. | 12. | 13. | 14. | 15. | 16. | 17. | 18. | 19. | 20. |
| --- | --- | --- | --- | --- | --- | --- | --- | --- | --- | --- | --- | --- | --- | --- | --- | --- | --- | --- | --- | --- |
| 1. EX | 1 |  |  |  |  |  |  |  |  |  |  |  |  |  |  |  |  |  |  |  |
| 2. AG | .238** | 1 |  |  |  |  |  |  |  |  |  |  |  |  |  |  |  |  |  |  |
| 3. CO | .167 | .442** | 1 |  |  |  |  |  |  |  |  |  |  |  |  |  |  |  |  |  |
| 4. EM | -.320** | -.487** | -.491** | 1 |  |  |  |  |  |  |  |  |  |  |  |  |  |  |  |  |
| 5. OP | .192* | .141 | .012 | -.073 | 1 |  |  |  |  |  |  |  |  |  |  |  |  |  |  |  |
| 6. Ires | -.024 | -.068 | -.118 | .064 | -.085 | 1 |  |  |  |  |  |  |  |  |  |  |  |  |  |  |
| 7. Time | .003 | .009 | .120 | -.061 | .012 | .041 | 1 |  |  |  |  |  |  |  |  |  |  |  |  |  |
| 8. Longstring | -.209* | -.238** | -.162 | .095 | -.212* | .287** | .111 | 1 |  |  |  |  |  |  |  |  |  |  |  |  |
| 9. IRV | .108 | .328** | .343** | -.166 | .238** | -.192* | .036 | -.480** | 1 |  |  |  |  |  |  |  |  |  |  |  |
| 10. Synonyms | .137 | .394** | .387** | -.214* | .106 | -.363** | -.249** | -.395** | .489** | 1 |  |  |  |  |  |  |  |  |  |  |
| 11. Antonyms | -.035 | -.142 | -.176 | .067 | -.118 | .325** | .226* | .341** | -.426** | -.743** | 1 |  |  |  |  |  |  |  |  |  |
| 12. RPR | -.036 | .253** | .252** | -.201* | .196* | -.219* | -.271** | -.373** | .407** | .683** | -.637** | 1 |  |  |  |  |  |  |  |  |
| 13. MD | -.121 | -.257** | -.189* | .177 | -.134 | .086 | .274** | -.026 | .414** | -.378** | .277** | -.314** | 1 |  |  |  |  |  |  |  |
| 14. *r*_pbis_ | .096 | .680** | .688** | -.486** | .406** | -.126 | .079 | -.117 | .120 | .341** | -.126 | .353** | -.495** | 1 |  |  |  |  |  |  |
| 15. Gnormed | -.079 | -.102 | -.056 | .034 | -.038 | .001 | .231* | -.099 | .615** | -.158 | .065 | -.161 | .880** | -.351** | 1 |  |  |  |  |  |
| 16. H_t_ | -.295** | -.012 | .035 | .098 | -.118 | -.106 | -.030 | .118 | -.068 | .166 | -.155 | .222* | -.294** | .241** | -.277** | 1 |  |  |  |  |
| 17. lz | .091 | .148 | .094 | -.101 | .088 | -.053 | -.252** | .068 | -.520** | .281** | -.169 | .272** | -.925** | .415** | -.961** | .341** | 1 |  |  |  |
| 18. Infit MSE | -.043 | -.041 | -.031 | .010 | .000 | -.017 | .230* | -.106 | .614** | -.161 | .096 | -.172 | .874** | -.290** | .981** | -.286** | -.949** | 1 |  |  |
| 19. Outfit MSE | -.029 | -.065 | -.055 | .024 | -.007 | -.006 | .218* | -.116 | .605** | -.186* | .103 | -.185* | .893** | -.325** | .977** | -.308** | -.960** | .992** | 1 |  |
| 20. INDCHI | -.096 | .065 | .020 | -.035 | -.001 | .099 | .069 | .196* | -.175 | -.369** | .299** | -.247** | .242** | .119 | .276** | -.233** | -.342** | .318** | .308** | 1 |
| *M* | 3.539 | 4.409 | 4.322 | 3.362 | 4.363 | 0.006 | 5.898 | 3.270 | 1.419 | 0.670 | -0.646 | 0.773 | 58.301 | 0.535 | 0.112 | 0.183 | 0.238 | 0.927 | 0.913 | -0.817 |
| *SD* | 0.884 | 0.704 | 0.839 | 1.062 | 0.774 | 0.032 | 3.810 | 1.072 | 0.302 | 0.189 | 0.226 | 0.234 | 24.561 | 0.231 | 0.064 | 0.079 | 0.997 | 0.458 | 0.428 | 2.133 |

*Note.* The careful responding group included 121 participants. All reported Pearson correlation coefficients and means and standard deviations are based on 121 observations. EX = Extraversion; AG = Agreeableness; CO = Conscientiousness; EM = Negative Emotionality; OP = Open-Mindedness; Ires = average score of the scored instructed response items; time = average response time per item; longstring = maximum longstring; IRV = intra-individual response variability; synonyms = psychometric synonyms; antonyms = psychometric antonyms; RPR = resampled personal reliability; MD = Mahalanobis distance measure for which the calculation was based on subsamples; *r*_pbis_ = person-total correlation coefficient for which the calculation was based on subsamples; Gnormed = global (i.e., the average of the scale-specific scores was computed) normed Guttman error index for polytomous items; H_t_ = global (i.e., the average of the scale-specific scores was computed) person scalability index; lz = global (i.e., the average of the scale-specific scores was computed) standardized log-likelihood index for polytomous items; infit MSE = global (i.e., the average of the scale-specific statistics was computed) Rasch model-based infit mean square error; outfit MSE = global (i.e., the average of the scale-specific statistics was computed) Rasch model-based outfit mean square error; INDCHI = global (i.e., the average of the scale-specific scores was computed) individual contribution to model misfit for which the calculation of the two person-specific model log-likelihood values was based on subsamples.

* *p* < .05 (two-tailed).

** *p* < .01 (two-tailed).

# Table S35

*Descriptive Statistics of the Substantive Measures and Careless Response Indices for the Unincentivized Careful Responding Group in Study 5 at t2*

| Scale | 1. | 2. | 3. | 4. | 5. | 6. | 7. | 8. | 9. | 10. | 11. | 12. | 13. | 14. | 15. | 16. | 17. | 18. | 19. | 20. |
| --- | --- | --- | --- | --- | --- | --- | --- | --- | --- | --- | --- | --- | --- | --- | --- | --- | --- | --- | --- | --- |
| 1. EX | 1 |  |  |  |  |  |  |  |  |  |  |  |  |  |  |  |  |  |  |  |
| 2. AG | .165 | 1 |  |  |  |  |  |  |  |  |  |  |  |  |  |  |  |  |  |  |
| 3. CO | .277** | .520** | 1 |  |  |  |  |  |  |  |  |  |  |  |  |  |  |  |  |  |
| 4. EM | -.475** | -.332** | -.467** | 1 |  |  |  |  |  |  |  |  |  |  |  |  |  |  |  |  |
| 5. OP | .166 | .247** | .199* | -.136 | 1 |  |  |  |  |  |  |  |  |  |  |  |  |  |  |  |
| 6. Ires | .134 | -.069 | -.135 | .005 | -.078 | 1 |  |  |  |  |  |  |  |  |  |  |  |  |  |  |
| 7. Time | .075 | -.033 | -.042 | .014 | -.029 | .043 | 1 |  |  |  |  |  |  |  |  |  |  |  |  |  |
| 8. Longstring | -.216* | -.317** | -.176 | .179 | -.221* | .021 | .291** | 1 |  |  |  |  |  |  |  |  |  |  |  |  |
| 9. IRV | -.008 | .199* | .231* | -.082 | .227* | -.100 | -.048 | -.276** | 1 |  |  |  |  |  |  |  |  |  |  |  |
| 10. Synonyms | -.024 | .389** | .335** | -.185* | .051 | -.100 | -.249** | -.345** | .275** | 1 |  |  |  |  |  |  |  |  |  |  |
| 11. Antonyms | .111 | -.238** | -.267** | .056 | .030 | .069 | .257** | .292** | -.257** | -.841** | 1 |  |  |  |  |  |  |  |  |  |
| 12. RPR | -.136 | .310** | .235** | -.121 | .118 | -.041 | -.420** | -.405** | .300** | .791** | -.723** | 1 |  |  |  |  |  |  |  |  |
| 13. MD | -.085 | -.426** | -.317** | .213* | -.085 | .008 | .166 | .150 | .535** | -.459** | .373** | -.331** | 1 |  |  |  |  |  |  |  |
| 14. *r*_pbis_ | .141 | .785** | .734** | -.373** | .503** | -.259** | -.130 | -.317** | .058 | .390** | -.252** | .381** | -.550** | 1 |  |  |  |  |  |  |
| 15. Gnormed | -.073 | -.239** | -.095 | .105 | .010 | -.053 | .104 | .102 | .731** | -.238** | .184* | -.133 | .889** | -.346** | 1 |  |  |  |  |  |
| 16. H_t_ | .111 | .404** | .509** | -.226* | .105 | .102 | -.023 | -.134 | -.129 | .168 | -.081 | .079 | -.449** | .528** | -.359** | 1 |  |  |  |  |
| 17. lz | .104 | .268** | .162 | -.131 | .009 | .039 | -.122 | -.104 | -.680** | .311** | -.234* | .182* | -.920** | .400** | -.959** | .438** | 1 |  |  |  |
| 18. Infit MSE | -.070 | -.154 | -.025 | .081 | .052 | -.074 | .098 | .059 | .769** | -.197* | .151 | -.101 | .861** | -.259** | .984** | -.280** | -.938** | 1 |  |  |
| 19. Outfit MSE | -.070 | -.155 | -.027 | .085 | .046 | -.072 | .092 | .058 | .758** | -.219* | .163 | -.116 | .870** | -.267** | .983** | -.292** | -.954** | .995** | 1 |  |
| 20. INDCHI | -.121 | .273** | .262** | -.148 | .193* | -.176 | .053 | .089 | -.003 | -.070 | .123 | .098 | -.029 | .385** | .083 | .091 | -.137 | .124 | .141 | 1 |
| *M* | 3.309 | 4.423 | 4.383 | 3.327 | 4.191 | 0.010 | 4.724 | 3.430 | 1.457 | 0.710 | -0.688 | 0.794 | 63.133 | 0.503 | 0.133 | 0.164 | 0.833 | 0.725 | 0.703 | -0.996 |
| *SD* | 0.845 | 0.936 | 0.915 | 1.118 | 0.849 | 0.062 | 3.131 | 1.370 | 0.368 | 0.226 | 0.243 | 0.311 | 36.732 | 0.300 | 0.101 | 0.077 | 1.003 | 0.485 | 0.457 | 2.590 |

*Note.* The careful responding group included 120 participants. All reported Pearson correlation coefficients and means and standard deviations are based on 120 observations. EX = Extraversion; AG = Agreeableness; CO = Conscientiousness; EM = Negative Emotionality; OP = Open-Mindedness; Ires = average score of the scored instructed response items; time = average response time per item; longstring = maximum longstring; IRV = intra-individual response variability; synonyms = psychometric synonyms; antonyms = psychometric antonyms; RPR = resampled personal reliability; MD = Mahalanobis distance measure for which the calculation was based on subsamples; *r*_pbis_ = person-total correlation coefficient for which the calculation was based on subsamples; Gnormed = global (i.e., the average of the scale-specific scores was computed) normed Guttman error index for polytomous items; H_t_ = global (i.e., the average of the scale-specific scores was computed) person scalability index; lz = global (i.e., the average of the scale-specific scores was computed) standardized log-likelihood index for polytomous items; infit MSE = global (i.e., the average of the scale-specific statistics was computed) Rasch model-based infit mean square error; outfit MSE = global (i.e., the average of the scale-specific statistics was computed) Rasch model-based outfit mean square error; INDCHI = global (i.e., the average of the scale-specific scores was computed) individual contribution to model misfit for which the calculation of the two person-specific model log-likelihood values was based on subsamples.

* *p* < .05 (two-tailed).

** *p* < .01 (two-tailed).

# Table S36

*Descriptive Statistics of the Substantive Measures and Careless Response Indices for the Incentivized Careful Responding Group in Study 5 at t2*

| Scale | 1. | 2. | 3. | 4. | 5. | 6. | 7. | 8. | 9. | 10. | 11. | 12. | 13. | 14. | 15. | 16. | 17. | 18. | 19. | 20. |
| --- | --- | --- | --- | --- | --- | --- | --- | --- | --- | --- | --- | --- | --- | --- | --- | --- | --- | --- | --- | --- |
| 1. EX | 1 |  |  |  |  |  |  |  |  |  |  |  |  |  |  |  |  |  |  |  |
| 2. AG | .207* | 1 |  |  |  |  |  |  |  |  |  |  |  |  |  |  |  |  |  |  |
| 3. CO | .455** | .415** | 1 |  |  |  |  |  |  |  |  |  |  |  |  |  |  |  |  |  |
| 4. EM | -.645** | -.325** | -.565** | 1 |  |  |  |  |  |  |  |  |  |  |  |  |  |  |  |  |
| 5. OP | .424** | .321** | .429** | -.374** | 1 |  |  |  |  |  |  |  |  |  |  |  |  |  |  |  |
| 6. Ires | -.012 | -.040 | .138 | -.059 | .091 | 1 |  |  |  |  |  |  |  |  |  |  |  |  |  |  |
| 7. Time | .027 | -.060 | -.049 | .023 | -.028 | .107 | 1 |  |  |  |  |  |  |  |  |  |  |  |  |  |
| 8. Longstring | -.131 | -.364** | -.281** | .229* | -.315** | -.078 | .034 | 1 |  |  |  |  |  |  |  |  |  |  |  |  |
| 9. IRV | .316** | .337** | .473** | -.298** | .303** | .104 | -.086 | -.453** | 1 |  |  |  |  |  |  |  |  |  |  |  |
| 10. Synonyms | .078 | .467** | .410** | -.173 | .290** | -.015 | .011 | -.492** | .439** | 1 |  |  |  |  |  |  |  |  |  |  |
| 11. Antonyms | .000 | -.267** | -.281** | .081 | -.111 | .042 | -.049 | .210* | -.342** | -.784** | 1 |  |  |  |  |  |  |  |  |  |
| 12. RPR | -.020 | .346** | .255** | -.153 | .200* | -.010 | -.021 | -.589** | .375** | .747** | -.764** | 1 |  |  |  |  |  |  |  |  |
| 13. MD | .102 | -.330** | -.154 | .074 | -.257** | .075 | .031 | .133 | .355** | -.504** | .430** | -.414** | 1 |  |  |  |  |  |  |  |
| 14. *r*_pbis_ | .297** | .704** | .698** | -.458** | .666** | .099 | -.044 | -.326** | .188* | .438** | -.215* | .336** | -.492** | 1 |  |  |  |  |  |  |
| 15. Gnormed | .310** | -.104 | .105 | -.130 | -.066 | .095 | -.033 | .057 | .632** | -.143 | .075 | -.168 | .819** | -.286** | 1 |  |  |  |  |  |
| 16. H_t_ | .020 | .210* | .423** | -.124 | .238** | .158 | -.013 | .068 | -.123 | .131 | -.141 | .056 | -.475** | .560** | -.430** | 1 |  |  |  |  |
| 17. lz | -.172 | .232* | -.031 | .027 | .126 | -.156 | -.010 | -.042 | -.567** | .292** | -.217* | .283** | -.908** | .365** | -.882** | .406** | 1 |  |  |  |
| 18. Infit MSE | .295** | -.010 | .195* | -.166 | .024 | .124 | -.014 | .005 | .686** | -.085 | .060 | -.130 | .789** | -.169 | .966** | -.335** | -.870** | 1 |  |  |
| 19. Outfit MSE | .246** | -.129 | .119 | -.117 | -.035 | .151 | .001 | .013 | .647** | -.160 | .118 | -.179 | .858** | -.245** | .932** | -.375** | -.951** | .949** | 1 |  |
| 20. INDCHI | .016 | -.227* | .017 | .043 | -.056 | .095 | .178 | .283** | -.211* | -.252** | .158 | -.272** | .234* | .024 | .174 | .055 | -.276** | .153 | .244** | 1 |
| *M* | 3.498 | 4.361 | 4.348 | 3.351 | 4.289 | 0.004 | 5.059 | 3.330 | 1.426 | 0.676 | -0.674 | 0.782 | 60.776 | 0.494 | 0.129 | 0.159 | 0.949 | 0.701 | 0.664 | -0.884 |
| *SD* | 0.911 | 0.803 | 0.909 | 1.048 | 0.945 | 0.023 | 3.174 | 1.373 | 0.362 | 0.234 | 0.259 | 0.256 | 34.055 | 0.285 | 0.108 | 0.084 | 0.883 | 0.504 | 0.427 | 2.828 |

*Note.* The careful responding group included 120 participants. All reported Pearson correlation coefficients and means and standard deviations are based on 120 observations. EX = Extraversion; AG = Agreeableness; CO = Conscientiousness; EM = Negative Emotionality; OP = Open-Mindedness; Ires = average score of the scored instructed response items; time = average response time per item; longstring = maximum longstring; IRV = intra-individual response variability; synonyms = psychometric synonyms; antonyms = psychometric antonyms; RPR = resampled personal reliability; MD = Mahalanobis distance measure for which the calculation was based on subsamples; *r*_pbis_ = person-total correlation coefficient for which the calculation was based on subsamples; Gnormed = global (i.e., the average of the scale-specific scores was computed) normed Guttman error index for polytomous items; H_t_ = global (i.e., the average of the scale-specific scores was computed) person scalability index; lz = global (i.e., the average of the scale-specific scores was computed) standardized log-likelihood index for polytomous items; infit MSE = global (i.e., the average of the scale-specific statistics was computed) Rasch model-based infit mean square error; outfit MSE = global (i.e., the average of the scale-specific statistics was computed) Rasch model-based outfit mean square error; INDCHI = global (i.e., the average of the scale-specific scores was computed) individual contribution to model misfit for which the calculation of the two person-specific model log-likelihood values was based on subsamples.

* *p* < .05 (two-tailed).

** *p* < .01 (two-tailed).

# Table S37

*Descriptive Statistics of the Substantive Measures and Careless Response Indices for the Partially Careless Responding Group (i.e., Careless 33%) in Study 5 at t2*

| Scale | 1. | 2. | 3. | 4. | 5. | 6. | 7. | 8. | 9. | 10. | 11. | 12. | 13. | 14. | 15. | 16. | 17. | 18. | 19. | 20. |
| --- | --- | --- | --- | --- | --- | --- | --- | --- | --- | --- | --- | --- | --- | --- | --- | --- | --- | --- | --- | --- |
| 1. EX | 1 |  |  |  |  |  |  |  |  |  |  |  |  |  |  |  |  |  |  |  |
| 2. AG | .127 | 1 |  |  |  |  |  |  |  |  |  |  |  |  |  |  |  |  |  |  |
| 3. CO | .328** | .306** | 1 |  |  |  |  |  |  |  |  |  |  |  |  |  |  |  |  |  |
| 4. EM | -.481** | -.219* | -.447** | 1 |  |  |  |  |  |  |  |  |  |  |  |  |  |  |  |  |
| 5. OP | .252** | .217* | .243** | -.084 | 1 |  |  |  |  |  |  |  |  |  |  |  |  |  |  |  |
| 6. Ires | -.050 | -.192* | -.122 | .028 | -.029 | 1 |  |  |  |  |  |  |  |  |  |  |  |  |  |  |
| 7. Time | -.116 | .238** | -.039 | .095 | -.031 | -.064 | 1 |  |  |  |  |  |  |  |  |  |  |  |  |  |
| 8. Longstring | -.142 | -.159 | -.243** | .119 | -.162 | .087 | .006 | 1 |  |  |  |  |  |  |  |  |  |  |  |  |
| 9. IRV | .147 | .439** | .324** | -.060 | .283** | -.050 | .096 | -.219* | 1 |  |  |  |  |  |  |  |  |  |  |  |
| 10. Synonyms | .037 | .361** | .299** | -.189* | .288** | -.084 | .070 | -.229* | .271** | 1 |  |  |  |  |  |  |  |  |  |  |
| 11. Antonyms | -.015 | -.160 | -.201* | .040 | -.220* | .028 | -.134 | .204* | -.101 | -.625** | 1 |  |  |  |  |  |  |  |  |  |
| 12. RPR | -.120 | .358** | .178 | -.098 | .152 | -.013 | .153 | -.237** | .220* | .647** | -.617** | 1 |  |  |  |  |  |  |  |  |
| 13. MD | .064 | .057 | -.021 | .138 | -.127 | .091 | -.017 | .030 | .669** | -.372** | .440** | -.337** | 1 |  |  |  |  |  |  |  |
| 14. *r*_pbis_ | .229* | .654** | .714** | -.405** | .578** | -.176 | .088 | -.192* | .252** | .449** | -.300** | .347** | -.248** | 1 |  |  |  |  |  |  |
| 15. Gnormed | .076 | .092 | .024 | .135 | .036 | .084 | .012 | .086 | .742** | -.183* | .278** | -.263** | .890** | -.170 | 1 |  |  |  |  |  |
| 16. H_t_ | .055 | -.024 | .312** | -.283** | .034 | -.114 | -.100 | .101 | -.108 | .062 | .020 | -.022 | -.188* | .333** | -.260** | 1 |  |  |  |  |
| 17. lz | -.056 | -.057 | .025 | -.150 | .017 | -.080 | -.008 | -.083 | -.694** | .247** | -.325** | .294** | -.902** | .215* | -.965** | .283** | 1 |  |  |  |
| 18. Infit MSE | .107 | .158 | .092 | .089 | .069 | .063 | .013 | .054 | .791** | -.150 | .247** | -.226* | .885** | -.097 | .992** | -.208* | -.950** | 1 |  |  |
| 19. Outfit MSE | .107 | .139 | .087 | .096 | .063 | .072 | .009 | .043 | .782** | -.165 | .255** | -.237** | .896** | -.107 | .991** | -.228* | -.965** | .996** | 1 |  |
| 20. INDCHI | .016 | -.095 | -.051 | .007 | .028 | .118 | -.140 | .131 | .047 | -.027 | .099 | -.209* | .105 | -.068 | .295** | .039 | -.359** | .271** | .298** | 1 |
| *M* | 3.478 | 4.133 | 4.011 | 3.386 | 3.937 | 0.137 | 4.138 | 3.760 | 1.355 | 0.347 | -0.344 | 0.430 | 105.554 | 0.329 | 0.190 | 0.121 | 0.104 | 0.976 | 0.967 | -0.823 |
| *SD* | 0.684 | 0.604 | 0.743 | 0.774 | 0.740 | 0.154 | 2.570 | 1.230 | 0.301 | 0.248 | 0.310 | 0.427 | 40.966 | 0.240 | 0.107 | 0.083 | 1.156 | 0.518 | 0.498 | 3.807 |

*Note.* The careful responding group included 120 participants. All reported Pearson correlation coefficients and means and standard deviations are based on 120 observations. EX = Extraversion; AG = Agreeableness; CO = Conscientiousness; EM = Negative Emotionality; OP = Open-Mindedness; Ires = average score of the scored instructed response items; time = average response time per item; longstring = maximum longstring; IRV = intra-individual response variability; synonyms = psychometric synonyms; antonyms = psychometric antonyms; RPR = resampled personal reliability; MD = Mahalanobis distance measure for which the calculation was based on subsamples; *r*_pbis_ = person-total correlation coefficient for which the calculation was based on subsamples; Gnormed = global (i.e., the average of the scale-specific scores was computed) normed Guttman error index for polytomous items; H_t_ = global (i.e., the average of the scale-specific scores was computed) person scalability index; lz = global (i.e., the average of the scale-specific scores was computed) standardized log-likelihood index for polytomous items; infit MSE = global (i.e., the average of the scale-specific statistics was computed) Rasch model-based infit mean square error; outfit MSE = global (i.e., the average of the scale-specific statistics was computed) Rasch model-based outfit mean square error; INDCHI = global (i.e., the average of the scale-specific scores was computed) individual contribution to model misfit for which the calculation of the two person-specific model log-likelihood values was based on subsamples.

* *p* < .05 (two-tailed).

** *p* < .01 (two-tailed).

# Table S38

*Descriptive Statistics of the Substantive Measures and Careless Response Indices for the Fully Careless Responding Group (i.e., Careless 100%) in Study 5 at t2*

| Scale | 1. | 2. | 3. | 4. | 5. | 6. | 7. | 8. | 9. | 10. | 11. | 12. | 13. | 14. | 15. | 16. | 17. | 18. | 19. | 20. |
| --- | --- | --- | --- | --- | --- | --- | --- | --- | --- | --- | --- | --- | --- | --- | --- | --- | --- | --- | --- | --- |
| 1. EX | 1 |  |  |  |  |  |  |  |  |  |  |  |  |  |  |  |  |  |  |  |
| 2. AG | -.153 | 1 |  |  |  |  |  |  |  |  |  |  |  |  |  |  |  |  |  |  |
| 3. CO | -.087 | .472** | 1 |  |  |  |  |  |  |  |  |  |  |  |  |  |  |  |  |  |
| 4. EM | -.064 | -.330** | -.458** | 1 |  |  |  |  |  |  |  |  |  |  |  |  |  |  |  |  |
| 5. OP | -.179 | .299** | .288** | -.066 | 1 |  |  |  |  |  |  |  |  |  |  |  |  |  |  |  |
| 6. Ires | -.136 | -.219* | -.186* | .068 | -.176 | 1 |  |  |  |  |  |  |  |  |  |  |  |  |  |  |
| 7. Time | .079 | .215* | .222* | -.061 | .056 | -.286** | 1 |  |  |  |  |  |  |  |  |  |  |  |  |  |
| 8. Longstring | -.025 | -.048 | -.052 | .013 | -.012 | .038 | .121 | 1 |  |  |  |  |  |  |  |  |  |  |  |  |
| 9. IRV | .045 | -.062 | -.026 | .091 | -.102 | -.062 | .000 | -.694** | 1 |  |  |  |  |  |  |  |  |  |  |  |
| 10. Synonyms | .221* | .324** | .210* | -.025 | .122 | -.278** | .343** | .084 | .116 | 1 |  |  |  |  |  |  |  |  |  |  |
| 11. Antonyms | -.166 | -.268** | -.213* | .033 | -.265** | .292** | -.210* | .065 | -.165 | -.615** | 1 |  |  |  |  |  |  |  |  |  |
| 12. RPR | .133 | .230* | .143 | -.122 | .098 | -.312** | .312** | -.140 | .326** | .510** | -.495** | 1 |  |  |  |  |  |  |  |  |
| 13. MD | -.094 | -.340** | -.232* | .176 | -.276** | .221* | -.118 | -.199* | .656** | -.350** | .245* | -.095 | 1 |  |  |  |  |  |  |  |
| 14. *r*_pbis_ | -.142 | .746** | .702** | -.368** | .614** | -.314** | .346** | -.043 | -.122 | .373** | -.388** | .334** | -.456** | 1 |  |  |  |  |  |  |
| 15. Gnormed | -.010 | -.269** | -.207* | .143 | -.217* | .189* | .115 | .496** | -.019 | -.115 | .105 | -.222* | .568** | -.387** | 1 |  |  |  |  |  |
| 16. H_t_ | -.064 | .105 | .244** | .041 | .042 | .063 | -.069 | .107 | -.197* | .232* | -.085 | -.160 | -.210* | .278** | -.112 | 1 |  |  |  |  |
| 17. lz | .050 | .234** | .181* | -.088 | .218* | -.206* | -.081 | -.479** | .027 | .214* | -.186 | .256** | -.599** | .367** | -.984** | .130 | 1 |  |  |  |
| 18. Infit MSE | -.017 | -.247** | -.182* | .131 | -.211* | .176 | .139 | .510** | -.030 | -.096 | .093 | -.205* | .553** | -.366** | .998** | -.110 | -.981** | 1 |  |  |
| 19. Outfit MSE | -.023 | -.228* | -.156 | .101 | -.208* | .188* | .130 | .515** | -.047 | -.121 | .107 | -.215* | .555** | -.358** | .995** | -.112 | -.985** | .997** | 1 |  |
| 20. INDCHI | -.031 | -.016 | .012 | .006 | -.005 | .083 | .172 | .728** | -.549** | .051 | .024 | -.277** | -.019 | -.016 | .764** | .102 | -.749** | .774** | .780** | 1 |
| *M* | 3.497 | 3.592 | 3.579 | 3.530 | 3.531 | 0.463 | 2.290 | 8.610 | 1.158 | 0.095 | -0.112 | -0.141 | 125.803 | 0.057 | 0.281 | 0.090 | -1.039 | 1.341 | 1.339 | 3.891 |
| *SD* | 0.462 | 0.549 | 0.542 | 0.523 | 0.451 | 0.397 | 1.666 | 13.482 | 0.470 | 0.300 | 0.279 | 0.598 | 51.213 | 0.230 | 0.177 | 0.118 | 2.025 | 0.836 | 0.829 | 10.728 |

*Note.* The careful responding group included 121 participants. All reported Pearson correlation coefficients and means and standard deviations are based on 121 observations except for those that involve the synonym (*n* = 113) and antonym index (*n* = 109) and the person-total correlation coefficient (*n* = 116). The missing values in these indices occurred because the response protocols of some participants had little or even no variance. EX = Extraversion; AG = Agreeableness; CO = Conscientiousness; EM = Negative Emotionality; OP = Open-Mindedness; Ires = average score of the scored instructed response items; time = average response time per item; longstring = maximum longstring; IRV = intra-individual response variability; synonyms = psychometric synonyms; antonyms = psychometric antonyms; RPR = resampled personal reliability; MD = Mahalanobis distance measure for which the calculation was based on subsamples; *r*_pbis_ = person-total correlation coefficient for which the calculation was based on subsamples; Gnormed = global (i.e., the average of the scale-specific scores was computed) normed Guttman error index for polytomous items; H_t_ = global (i.e., the average of the scale-specific scores was computed) person scalability index; lz = global (i.e., the average of the scale-specific scores was computed) standardized log-likelihood index for polytomous items; infit MSE = global (i.e., the average of the scale-specific statistics was computed) Rasch model-based infit mean square error; outfit MSE = global (i.e., the average of the scale-specific statistics was computed) Rasch model-based outfit mean square error; INDCHI = global (i.e., the average of the scale-specific scores was computed) individual contribution to model misfit for which the calculation of the two person-specific model log-likelihood values was based on subsamples.

* *p* < .05 (two-tailed).

** *p* < .01 (two-tailed).

# Table S39

*Ordered Accuracies of the 14 Indirect Indices in Detecting Partially Careless (i.e., Careless 33%)* *Responding and Corresponding Pairwise Comparison Results in Study 5*

| Index | AUC_[95% CI]_ | Sen95 ^a^ | Sen99 ^b^ | 1. | 2. | 3. | 4. | 5. | 6. | 7. | 8. | 9. | 10. | 11. | 12. | 13. |
| --- | --- | --- | --- | --- | --- | --- | --- | --- | --- | --- | --- | --- | --- | --- | --- | --- |
| 1. Synonyms | .82_[.77, .87]_ | .29 | .02 |  |  |  |  |  |  |  |  |  |  |  |  |  |
| 2. MD | .81_[.77, .86]_ | .40 | .20 | 0.12 |  |  |  |  |  |  |  |  |  |  |  |  |
| 3. RPR | .81_[.76, .85]_ | .28 | .03 | 0.82 | 0.20 |  |  |  |  |  |  |  |  |  |  |  |
| 4. Antonyms | .74_[.70, .80]_ | .30 | .08 | 11.36 | 8.09 | 7.45 |  |  |  |  |  |  |  |  |  |  |
| 5. Gnormed | .72_[.68, .77]_ | .23 | .13 | 22.91 | 59.48 | 16.51 | 0.97 |  |  |  |  |  |  |  |  |  |
| 6. H_t_ | .71_[.66, .77]_ | .21 | .11 | 9.39 | 7.99 | 6.08 | 0.82 | 0.04 |  |  |  |  |  |  |  |  |
| 7. *r*_pbis_ | .70_[.66, .74]_ | .11 | NA ^c^ | 23.52 | 23.91 | 20.70 | 2.89 | 0.88 | 0.20 |  |  |  |  |  |  |  |
| 8. Time | .68_[.64, .72]_ | .26 | .15 | 22.31 | 20.37 | 16.39 | 4.39 | 1.79 | 1.04 | 0.49 |  |  |  |  |  |  |
| 9. Longstring | .59_[.54, .65]_ | .12 | NA ^c^ | 34.22 | 31.85 | 34.63 | 15.82 | 10.27 | 7.64 | 8.46 | 5.91 |  |  |  |  |  |
| 10. IRV | .52_[.48, .56]_ | .05 | .02 | 75.05 | 59.47 | 61.63 | 35.56 | 25.65 | 29.37 | 32.66 | 32.24 | 4.97 |  |  |  |  |
| 11. Infit MSE | .50_[.45, .56]_ | .06 | .02 | 167.37 | 255.47 | 165.61 | 82.75 | 231.25 | 33.22 | 58.07 | 25.71 | 4.64 | 0.17 |  |  |  |
| 12. Outfit MSE | .50_[.45, .56]_ | .06 | .03 | 163.74 | 251.52 | 165.99 | 81.05 | 227.60 | 34.13 | 58.84 | 25.60 | 4.79 | 0.21 | 0.30 |  |  |
| 13. lz | .50_[.44, .55]_ | .06 | .04 | 186.42 | 276.45 | 166.52 | 89.32 | 265.27 | 37.97 | 66.50 | 26.96 | 4.88 | 0.26 | 0.14 | 0.04 |  |
| 14. INDCHI | .46_[.40, .52]_ | .11 | .08 | 85.50 | 86.17 | 91.68 | 52.79 | 55.21 | 36.78 | 44.18 | 34.70 | 11.72 | 2.62 | 1.23 | 1.13 | 1.08 |

*Note.* The ratings of the first and second measurement of the partially careless responding condition (*n* = 120) were used to determine the classification accuracy. All indices were coded such that higher scores were more indicative of careless responding. Values in the matrix part represent the χ^2^ values for the pairwise comparisons with one degree of freedom. The Bonferroni-corrected critical χ^2^ value for each of the 91 comparisons was 11.94; if this value is exceeded, the two AUCs can be considered significantly different from each other, and the corresponding cells in the matrix part are shown in gray. AUC = area under the receiver operating characteristic curve; time = average response time per item; longstring = maximum longstring; IRV = intra-individual response variability; synonyms = psychometric synonyms; antonyms = psychometric antonyms; RPR = resampled personal reliability; MD = Mahalanobis distance measure for which the calculation was based on subsamples; *r*_pbis_ = person-total correlation coefficient for which the calculation was based on subsamples; Gnormed = global (i.e., the average of the scale-specific scores was computed) normed Guttman error index for polytomous items; H_t_ = global (i.e., the average of the scale-specific scores was computed) person scalability index; lz = global (i.e., the average of the scale-specific scores was computed) standardized log-likelihood index for polytomous items; infit MSE = global (i.e., the average of the scale-specific statistics was computed) Rasch model-based infit mean square error; outfit MSE = global (i.e., the average of the scale-specific statistics was computed) Rasch model-based outfit mean square error; INDCHI = global (i.e., the average of the scale-specific scores was computed) individual contribution to model misfit for which the calculation of the two person-specific model log-likelihood values was based on subsamples.

^a^ Sensitivity of the index at specificity level of 95% (i.e., false-positive rate of 5%). ^b^ Sensitivity of the index at specificity level of 99% (i.e., false-positive rate of 1%). ^c^ Sensitivities could not be computed because of missing values in the matrix (i.e., non-zero sensitivities could only be calculated for false-positive rates that were higher than the 1% level that we aimed for).

# Table S40

*Ordered Accuracies of the 14 Indirect Indices in Detecting Fully Careless (i.e., Careless 100%)* *Responding and Corresponding Pairwise Comparison Results in Study 5*

| Index | AUC_[95% CI]_ | Sen95 ^a^ | Sen99 ^b^ | 1. | 2. | 3. | 4. | 5. | 6. | 7. | 8. | 9. | 10. | 11. | 12. | 13. |
| --- | --- | --- | --- | --- | --- | --- | --- | --- | --- | --- | --- | --- | --- | --- | --- | --- |
| 1. RPR | .93_[.90, .96]_ | .79 | .45 |  |  |  |  |  |  |  |  |  |  |  |  |  |
| 2. Time | .92_[.88, .95]_ | .76 | .73 | 0.43 |  |  |  |  |  |  |  |  |  |  |  |  |
| 3. *r*_pbis_ | .91_[.88, .95]_ | .60 | .10 | 1.25 | 0.07 |  |  |  |  |  |  |  |  |  |  |  |
| 4. Synonyms | .90_[.86, .95]_ | .75 | .63 | 1.96 | 0.35 | 0.15 |  |  |  |  |  |  |  |  |  |  |
| 5. Antonyms | .90_[.85, .95]_ | .64 | .25 | 2.03 | 0.44 | 0.23 | 0.02 |  |  |  |  |  |  |  |  |  |
| 6. MD | .88_[.83, .92]_ | .65 | .45 | 5.87 | 2.86 | 2.55 | 1.15 | 0.78 |  |  |  |  |  |  |  |  |
| 7. Gnormed | .84_[.79, .89]_ | .54 | .28 | 12.32 | 8.12 | 8.60 | 4.68 | 3.76 | 6.94 |  |  |  |  |  |  |  |
| 8. H_t_ | .73_[.68, .79]_ | .36 | .16 | 39.97 | 37.57 | 33.39 | 27.40 | 21.65 | 18.49 | 8.46 |  |  |  |  |  |  |
| 9. lz | .71_[.65, .79]_ | .40 | .10 | 55.23 | 41.66 | 50.52 | 32.19 | 29.80 | 59.56 | 72.97 | 0.42 |  |  |  |  |  |
| 10. Longstring | .69_[.63, .75]_ | .27 | .21 | 55.90 | 42.99 | 38.86 | 30.12 | 30.36 | 20.06 | 11.42 | 1.03 | 0.16 |  |  |  |  |
| 11. Outfit MSE | .67_[.60, .74]_ | .33 | .10 | 62.09 | 50.20 | 55.45 | 39.04 | 37.61 | 71.59 | 103.68 | 2.38 | 16.03 | 0.17 |  |  |  |
| 12. Infit MSE | .66_[.60, .73]_ | .25 | .08 | 64.52 | 51.45 | 57.37 | 40.19 | 38.76 | 74.05 | 111.10 | 2.66 | 17.74 | 0.25 | 2.13 |  |  |
| 13. IRV | .66_[.60, .72]_ | .29 | .14 | 66.17 | 47.35 | 42.28 | 36.59 | 36.50 | 22.96 | 12.91 | 2.46 | 0.70 | 0.64 | 0.01 | 0.00 |  |
| 14. INDCHI | .64_[.57, .71]_ | .42 | .23 | 58.73 | 51.35 | 45.16 | 37.89 | 37.14 | 34.29 | 26.17 | 4.26 | 2.73 | 1.70 | 0.51 | 0.38 | 0.31 |

*Note.* The ratings of the first and second measurement of the fully careless responding condition (*n* = 121) were used to determine the classification accuracy. Because four indices (i.e., synonyms, antonyms, *r*_pbis_) could not be computed for every participant of the fully careless responding condition, all reported estimates and test statistics are based on 100 multiply imputed data sets. All indices were coded such that higher scores were more indicative of careless responding. Values in the matrix part represent the *F*-values for the pairwise comparisons with one degree of freedom for the numerator and degrees of freedom that ranged from 409.5 to ∞ for the denominator. With the most conservative denominator value of 409.5, the Bonferroni-corrected critical *F*-value for each of the 91 comparisons was 12.13; if this value is exceeded, the two AUCs can be considered significantly different from each other, and the corresponding cells in the matrix part are shown in gray. AUC = area under the receiver operating characteristic curve; time = average response time per item; longstring = maximum longstring; IRV = intra-individual response variability; synonyms = psychometric synonyms; antonyms = psychometric antonyms; RPR = resampled personal reliability; MD = Mahalanobis distance measure for which the calculation was based on subsamples; *r*_pbis_ = person-total correlation coefficient for which the calculation was based on subsamples; Gnormed = global (i.e., the average of the scale-specific scores was computed) normed Guttman error index for polytomous items; H_t_ = global (i.e., the average of the scale-specific scores was computed) person scalability index; lz = global (i.e., the average of the scale-specific scores was computed) standardized log-likelihood index for polytomous items; infit MSE = global (i.e., the average of the scale-specific statistics was computed) Rasch model-based infit mean square error; outfit MSE = global (i.e., the average of the scale-specific statistics was computed) Rasch model-based outfit mean square error; INDCHI = global (i.e., the average of the scale-specific scores was computed) individual contribution to model misfit for which the calculation of the two person-specific model log-likelihood values was based on subsamples.

^a^ Sensitivity of the index at specificity level of 95% (i.e., false-positive rate of 5%). ^b^ Sensitivity of the index at specificity level of 99% (i.e., false-positive rate of 1%). ^c^ Sensitivities could not be computed because of missing values in the matrix (i.e., non-zero sensitivities could only be calculated for false-positive rates that were higher than the 1% level that we aimed for).

# Table S41

*Incremental Validity Beyond Average Score of Instructed Response Items of Each Indirect Index in Study 5*

|  | Careless 33% ^a^ | |  | Careless 100% ^b^ | |
| --- | --- | --- | --- | --- | --- |
| Index | AUC_[95% CI]_ | χ^2^(1) |  | AUC_[95% CI]_ | χ^2^(1) |
| Instructed Responses (reference) | .75_[.71, .80]_ |  |  | .84 _[.79, .88]_ |  |
| IRES + Synonyms | .88_[.84, .92]_ | 36.90 |  | .94_[.90, .97]_ | 22.25 |
| IRES + MD | .88_[.84, .92]_ | 36.46 |  | .92_[.89, .96]_ | 20.05 |
| IRES + RPR | .87_[.83, .91]_ | 30.77 |  | .95_[.92, .98]_ | 29.64 |
| IRES + Antonyms | .85_[.80, .89]_ | 23.10 |  | .94_[.91, .97]_ | 30.21 |
| IRES + Gnormed | .84_[.79, .88]_ | 20.01 |  | .92_[.88, .95]_ | 16.26 |
| IRES + H_t_ | .84_[.79, .88]_ | 20.59 |  | .92_[.89, .96]_ | 21.90 |
| IRES + *r*_pbis_ | .82_[.78, .86]_ | 14.98 |  | .94_[.92, .97]_ | 29.21 |
| IRES + Time | .83_[.78, .88]_ | 16.68 |  | .94_[.92, .97]_ | 28.19 |
| IRES + Longstring | .77_[.72, .82]_ | 1.45 |  | .90_[.86, .93]_ | 13.05 |
| IRES + IRV | - | - |  | .87_[.82, .92]_ | 3.40 |
| IRES + Infit MSE | - | - |  | .86_[.81, .91]_ | 1.26 |
| IRES + Outfit MSE | - | - |  | .86_[.81, .91]_ | 1.39 |
| IRES + lz | - | - |  | .87_[.82, .92]_ | 3.08 |
| IRES + INDCHI | - | - |  | .90_[.85, .94]_ | 9.13 |

*Note*. Only indirect indices that turned out be effective in detecting partially or fully careless responding were examined. Because three indices (i.e., synonyms, antonyms, *r*_pbis_) could not be computed every participant of the fully careless responding condition, we used linear multiple imputation predictions (across 100 imputations) of logit models as input for the ROC analyses in the fully careless responding condition. All indices were coded such that higher scores were more indicative of careless responding. The Bonferroni-corrected critical χ^2^ value for each of the 22 comparisons was 9.32 (i.e., 9 pairwise comparisons in the partially careless responding condition, 13 pairwise comparisons in the fully careless responding condition). If this value is exceeded, the AUCs can be considered significantly different from each other. AUC = area under the receiver operating characteristic curve; IRES = average score of instructed response items; time = average response time per item; longstring = maximum longstring; IRV = intra-individual response variability; synonyms = psychometric synonyms; antonyms = psychometric antonyms; RPR = resampled personal reliability; MD = Mahalanobis distance measure for which the calculation was based on subsamples; *r*_pbis_ = person-total correlation coefficient for which the calculation was based on subsamples; Gnormed = global (i.e., the average of the scale-specific scores was computed) normed Guttman error index for polytomous items; H_t_ = global (i.e., the average of the scale-specific scores was computed) person scalability index; lz = global (i.e., the average of the scale-specific scores was computed) standardized log-likelihood index for polytomous items; infit MSE = global (i.e., the average of the scale-specific statistics was computed) Rasch model-based infit mean square error; outfit MSE = global (i.e., the average of the scale-specific statistics was computed) Rasch model-based outfit mean square error; INDCHI = global (i.e., the average of the scale-specific scores was computed) individual contribution to model misfit for which the calculation of the two person-specific model log-likelihood values was based on subsamples.

^a^ The ratings of the first and second measurement of the partially careless responding condition (*n* = 120) were used to determine the classification accuracy. ^b^ The ratings of the first and second measurement of the fully careless responding condition (*n* = 121) were used to determine the classification accuracy.

# Table S42

*Accuracy of Careless Responding Indices in Study 4 and 5 When Using a Within-Subject and Between-Subject Design for the Analysis*

|  | Study 4 | | | | |  | Study 5 | | | | |
| --- | --- | --- | --- | --- | --- | --- | --- | --- | --- | --- | --- |
|  | Detecting partially CR | |  | Detecting fully CR | |  | Detecting partially CR | |  | Detecting fully CR | |
|  | WS ^a^ | BS ^b^ |  | WS ^c^ | BS ^d^ |  | WS ^e^ | BS ^f^ |  | WS ^g^ | BS ^h^ |
| Index | AUC_[95% CI]_ | AUC_[95% CI]_ |  | AUC_[95% CI]_ | AUC_[95% CI]_ |  | AUC_[95% CI]_ | AUC_[95% CI]_ |  | AUC_[95% CI]_ | AUC_[95% CI]_ |
| Indirect indices |  |  |  |  |  |  |  |  |  |  |  |
| RPR | .84_[.80, .89]_ | .88_[.85, .92]_ |  | .93_[.90, .97]_ | .94_[.90, .97]_ |  | .81_[.76, .85]_ | .85_[.81, .89]_ |  | .93_[.90, .96]_ | .93_[.90, .96]_ |
| Synonyms | .85_[.80, .89]_ | .89_[.86, .93]_ |  | .94_[.91, .98]_ | .94_[.91, .97]_ |  | .82_[.77, .87]_ | .86_[.82, .90]_ |  | .90_[.86, .95]_ | .90_[.86, .95]_ |
| Antonyms | .80_[.76, .85]_ | .87_[.83, .91]_ |  | .90_[.84, .96]_ | .89_[.83, .95]_ |  | .74_[.70, .80]_ | .82_[.78, .87]_ |  | .90_[.85, .95]_ | .90_[.86, .95]_ |
| MD | .88_[.84, .91]_ | .88_[.85, .92]_ |  | .86_[.81, .91]_ | .84_[.79, .89]_ |  | .81_[.77, .86]_ | .81_[.77, .86]_ |  | .88_[.83, .92]_ | .85_[.80, .90]_ |
| *r*_pbis_ | .77_[.73, .81]_ | .70_[.65, .76]_ |  | .94_[.91, .97]_ | .91_[.88, .95]_ |  | .70_[.66, .74]_ | .71_[.66, .77]_ |  | .91_[.88, .95]_ | .87_[.83, .91]_ |
| Gnormed | .78_[.74, .83]_ | .78_[.73, .83]_ |  | .83_[.78, .88]_ | .79_[.74, .85]_ |  | .72_[.68, .77]_ | .70_[.65, .76]_ |  | .84_[.79, .89]_ | .80_[.75, .85]_ |
| H_t_ | .73_[.68, .79]_ | .66_[.60, .72]_ |  | .70_[.65, .76]_ | .66_[.60, .72]_ |  | .71_[.66, .77]_ | .65_[.59, .71]_ |  | .73_[.68, .79]_ | .69_[.63, .75]_ |
| Time | .69_[.65, .74]_ | .60_[.53, .66]_ |  | .91_[.88, .95]_ | .87_[.82, .91]_ |  | .68_[.64, .72]_ | .61_[.55, .68]_ |  | .92_[.88, .95]_ | .88_[.83, .92]_ |
| lz | .58_[.52, .63]_ | .79_[.75, .84]_ |  | .72_[.65, .78]_ | .81_[.76, .86]_ |  | .50_[.44, .55]_ | .72_[.67, .77]_ |  | .71_[.65, .79]_ | .83_[.78, .87]_ |
| Outfit MSE | .59_[.53, .64]_ | .77_[.72, .82]_ |  | .69_[.63, .76]_ | .77_[.72, .83]_ |  | .50_[.45, .56]_ | .69_[.63, .75]_ |  | .67_[.60, .74]_ | .78_[.73, .83]_ |
| Infit MSE | .58_[.53, .63]_ | .76_[.71, .81]_ |  | .69_[.62, .75]_ | .77_[.71, .82]_ |  | .50_[.45, .56]_ | .68_[.62, .73]_ |  | .66_[.60, .73]_ | .77_[.72, .82]_ |
| INDCHI | .56_[.49, .63]_ | .56_[.49, .62]_ |  | .49_[.42, .56]_ | .48_[.41, .55]_ |  | .46_[.40, .52]_ | .48_[.42, .55]_ |  | .64_[.57, .71]_ | .64_[.58, .71]_ |
| Longstring | .58_[.52, .64]_ | .63_[.58, .69]_ |  | .70_[.64, .76]_ | .72_[.66, .77]_ |  | .59_[.54, .65]_ | .60_[.54, .66]_ |  | .69_[.63, .75]_ | .68_[.62, .74]_ |
| IRV | .48_[.44, .52]_ | .47_[.41, .53]_ |  | .60_[.53, .66]_ | .61_[.54, .67]_ |  | .52_[.48, .56]_ | .57_[.51, .63]_ |  | .66_[.60, .72]_ | .67_[.61, .73]_ |
| Direct indices |  |  |  |  |  |  |  |  |  |  |  |
| Bogus | .89_[.85, .93]_ | .88_[.84, .92]_ |  | .98_[.96, .998]_ | .96_[.94, .99]_ |  | - | - |  | - | - |
| Ires | - | - |  | - | - |  | .75_[.71, .80]_ | .76_[.71, .80]_ |  | .84_[.79, .88]_ | .84_[.80, .88]_ |

*Note*. Because some indices could not be computed for every participant of the fully careless responding conditions, all reported estimates and test statistics for the fully careless responding conditions are based on 100 multiply imputed data sets. All indices were coded such that higher scores were more indicative of careless responding.CR = careless responding; AUC = area under the receiver operating characteristic curve; time = average response time per item; longstring = maximum longstring; IRV = intra-individual response variability; synonyms = psychometric synonyms; antonyms = psychometric antonyms; RPR = resampled personal reliability; MD = Mahalanobis distance measure for which the calculation was based on subsamples; *r*_pbis_ = person-total correlation coefficient for which the calculation was based on subsamples; Gnormed = global (i.e., the average of the scale-specific scores was computed) normed Guttman error index for polytomous items; H_t_ = global (i.e., the average of the scale-specific scores was computed) person scalability index; lz = global (i.e., the average of the scale-specific scores was computed) standardized log-likelihood index for polytomous items; infit MSE = global (i.e., the average of the scale-specific statistics was computed) Rasch model-based infit mean square error; outfit MSE = global (i.e., the average of the scale-specific statistics was computed) Rasch model-based outfit mean square error; INDCHI = global (i.e., the average of the scale-specific scores was computed) individual contribution to model misfit for which the calculation of the two person-specific model log-likelihood values was based on subsamples; bogus = average of eight bogus items; Ires = average score of eight instructed response items.

^a^ The ratings of the first and second measurement of the partially careless responding condition (*n* = 118) of Study 4 were used to determine the classification accuracy. ^b^ The ratings of the partially careless responding condition (*n* = 118) and the two careful responding conditions of the second measurement (*n* = 441 [*n*_Careful unincentivized_ = 120; *n*_Careful incentivized_ = 121]) of Study 4 were used to determine the classification accuracy. ^c^ The ratings of the first and second measurement of the fully careless responding condition (*n* = 122) of Study 4 were used to determine the classification accuracy. ^d^ The ratings of the fully careless responding condition (*n* = 122) and the two careful responding conditions of the second measurement (*n* = 441 [*n*_Careful unincentivized_ = 120; *n*_Careful incentivized_ = 121]) of Study 4 were used to determine the classification accuracy. ^e^ The ratings of the first and second measurement of the partially careless responding condition (*n* = 120) of Study 5 were used to determine the classification accuracy. ^f^ The ratings of the partially careless responding condition (*n* = 120) and the two careful responding conditions of the second measurement (*n* = 440 [*n*_Careful unincentivized_ = 120; *n*_Careful incentivized_ = 120]) of Study 5 were used to determine the classification accuracy. ^g^ The ratings of the first and second measurement of the fully careless responding condition (*n* = 121) of Study 5 were used to determine the classification accuracy. ^h^ The ratings of the fully careless responding condition (*n* = 121) and the two careful responding conditions of the second measurement (*n* = 440 [*n*_Careful unincentivized_ = 120; *n*_Careful incentivized_ = 120]) of Study 5 were used to determine the classification accuracy.

# References

Andrich, D. (1978). A rating formulation for ordered response categories. *Psychometrika, 43*, 561-573.

Brown, W. (1910). Some experimental results in the correlation of mental abilities. *British Journal of Psychology, 3*, 296-322.

Cox, N. J. (2007). Speaking Stata: Identifying spells. *Stata Journal, 7*, 249-265.

Curran, P. G. (2016). Methods for the detection of carelessly invalid responses in survey data. *Journal of Experimental Social Psychology, 66*, 4-19.

Drasgow, F., Levine, M. V., & McLaughlin, M. E. (1991). Appropriateness measurement for some multidimensional test batteries. *Applied Psychological Measurement, 15*, 171-191.

Drasgow, F., Levine, M. V., & Williams, E. A. (1985). Appropriateness measurement with polychotomous item response models and standardized indices. *British Journal of Mathematical and Statistical Psychology, 38*, 67-86.

Emons, W. H. (2008). Nonparametric person-fit analysis of polytomous item scores. *Applied Psychological Measurement*, 32, 224-247.

Goldammer, P., Annen, H., Stöckli, P. L., & Jonas, K. (2020). Careless responding in questionnaire measures: Detection, impact, and remedies. *The Leadership Quarterly, 31*, 101384. https://doi.org/10.1016/j.leaqua.2020.101384

Goldammer, P., Stöckli, P. L., Escher, Y. A., Annen, H., & Jonas, K. (2023). On the utility of indirect methods for detecting faking. *Educational and Psychological Measurement*, Advance online publication. https://doi.org/10.1177/00131644231209520

Goldberg, L. R., Johnson, J. A., Eber, H. W., Hogan, R., Ashton, M. C., Cloninger, C. R., & Gough, H. C. (2006). The International Personality Item Pool and the future of public-domain personality measures. *Journal of Research in Personality, 40*, 84-96.

Huang, J. L., Liu, M., & Bowling, N. A. (2015). Insufficient effort responding: Examining an insidious confound in survey data. *Journal of Applied Psychology, 100*(3), 828–845. https://doi.org/10.1037/a0038510

Iller, M. -L., Grunder, M., & Schreiber, M. (2020). *Handbuch Fragebogen zur Erfassung der Persönlichkeit* *(IPIP-5F30F-R1)* [Personality Assessment Questionnaire Manual (IPIP-5F30F-R1)]. Institute of Applied Psychology, Zurich University of Applied Sciences.

McArdle, J. J., & Hamagami, F. (2001). Latent difference score structural models for linear dynamic analyses with incomplete longitudinal data. In L. M. Collins & A. G. Sayer (Eds.), *New methods for the analysis of change. Decade of behavior* (pp. 139-175). Washington, DC: American Psychological Association.

Meade, A. W., & Craig, S. B. (2012). Identifying careless responses in survey data. *Psychological Methods, 17*, 437-455.

Molenaar, I. W. (1991). A weighted Loevinger H-coefficient extending Mokken scaling to multicategory items. *Kwantitatieve Methoden, 12*, 97-117.

Muthén, L. K., & Muthén, B. O. (1998-2017). *Mplus user’s guide* (8th ed.). Muthén & Muthén.

Newsom, J. T. (2015). *Longitudinal structural equation modeling: A comprehensive introduction*. New York, NY: Routledge.

Niessen, A. S. M., Meijer, R. R., & Tendeiro, J. N. (2016). Detecting careless respondents in web-based questionnaires: Which method to use? *Journal of Research in Personality, 63*, 1-11.

R Core Team. (2022). *R: A language and environment for statistical computing*. R Foundation for Statistical Computing.

Reise, S. P., & Widaman, K. F. (1999). Assessing the fit of measurement models at the individual level: A comparison of item response theory and covariance structure approaches. *Psychological Methods, 4*, 3-21.

Robitzsch A, Kiefer T, Wu M (2022). *TAM: Test Analysis Modules*. R package version 4.1-4, https://CRAN.R-project.org/package=TAM.

Samejima, F. (1969). *Estimation of latent ability using a response pattern of graded scores* (Psychometric Monograph No. 17). Psychometric Society.

Soto, C. J., & John, O. P. (2017). The next Big Five Inventory (BFI-2): Developing and assessing a hierarchical model with 15 facets to enhance bandwidth, fidelity, and predictive power. *Journal of Personality and Social Psychology, 113*, 117-143.

Spearman, C. (1910). Correlation calculated from faulty data. *British Journal of Psychology, 3*, 271-295.

StataCorp. (2021). *Stata Statistical Software: Release 17*. StataCorp LLC.

Tendeiro, J. N., Meijer, R. R., & Niessen, A. S. M. (2016). PerFit: An R package for person-fit analysis in IRT. *Journal of Statistical Software, 74*, 1-27.

Van der Ark, L. A. (2007). Mokken scale analysis in R. *Journal of Statistical Software, 20*, 1-19.

Wright, B. D., & Stone, M. H. (1979). *Best test design*. MESA Press.
